# Structural and Functional Diversity in Rigid Thiosemicarbazones with Extended Aromatic Frameworks: Microwave-Assisted Synthesis and Structural Investigations

**DOI:** 10.1021/acsomega.2c08157

**Published:** 2023-04-25

**Authors:** Fernando Cortezon-Tamarit, Kexin Song, Navaratnarajah Kuganathan, Rory L. Arrowsmith, Sara Raquel Mota Merelo de Aguiar, Philip A. Waghorn, Adam Brookfield, Muralidharan Shanmugam, David Collison, Haobo Ge, Gabriele Kociok-Köhn, Charareh Pourzand, Jonathan Robin Dilworth, Sofia Ioana Pascu

**Affiliations:** □ Department of Chemistry, University of Bath, Claverton Down, Bath, BA2 7AY, United Kingdom; ■ Department of Materials, Imperial College London, Royal School of Mines, Exhibition Road, London SW7 2AZ, U.K.; § Department of Chemistry, Chemistry Research Laboratory, University of Oxford, Mansfield Road, Oxford, OX1 3TA, United Kingdom; ∥ Department of Chemistry, and Photon Science Institute, The University of Manchester, Oxford Road, Manchester M13 9PL, United Kingdom; ⊥ Department of Life Sciences, University of Bath, Bath BA2 7AY, U.K.; # Centre of Therapeutic Innovation, University of Bath, Bath BA2 7AY, U.K.

## Abstract

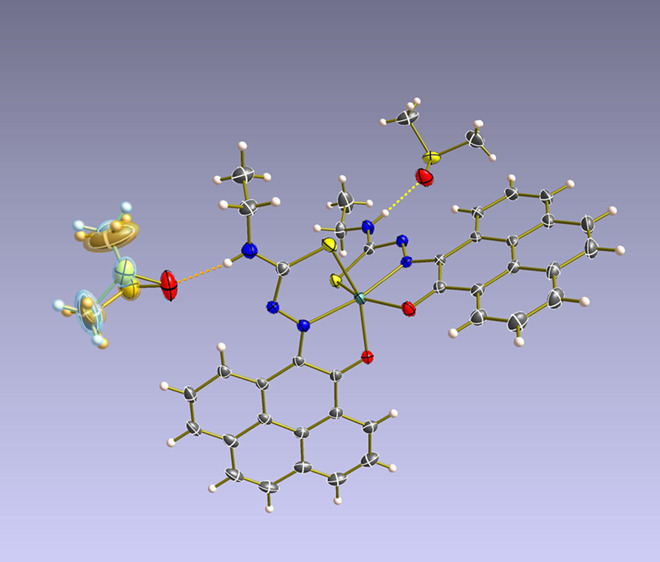

The long-standing interest in thiosemicarbazones (TSCs) has been largely driven by their potential toward theranostic applications including cellular imaging assays and multimodality imaging. We focus herein on the results of our new investigations into: (a) the structural chemistry of a family of rigid mono(thiosemicarbazone) ligands characterized by extended and aromatic backbones and (b) the formation of their corresponding thiosemicarbazonato Zn(II) and Cu(II) metal complexes. The synthesis of new ligands and their Zn(II) complexes was performed using a rapid, efficient and straightforward microwave-assisted method which superseded their preparation by conventional heating. We describe hereby new microwave irradiation protocols that are suitable for both imine bond formation reactions in the thiosemicabazone ligand synthesis and for Zn(II) metalation reactions. The new thiosemicarbazone ligands, denoted HL, mono(4-*R*-3-thiosemicarbazone)quinone, and their corresponding Zn(II) complexes, denoted ZnL_2_, mono(4-*R*-3-thiosemicarbazone)quinone, where R = H, Me, Ethyl, Allyl, and Phenyl, quinone = acenapthnenequinone (AN), aceanthrenequinone (AA), phenanthrenequinone (PH), and pyrene-4,5-dione (PY) were isolated and fully characterized spectroscopically and by mass spectrometry. A plethora of single crystal X-ray diffraction structures were obtained and analyzed and the geometries were also validated by DFT calculations. The Zn(II) complexes presented either distorted octahedral geometry or tetrahedral arrangements of the O/N/S donors around the metal center. The modification of the thiosemicarbazide moiety at the exocyclic N atoms with a range of organic linkers was also explored, opening the way to bioconjugation protocols for these compounds. The radiolabeling of these thiosemicarbazones with ^64^Cu was achieved under mild conditions for the first time: this cyclotron-available radioisotope of copper (*t*_1/2_ = 12.7 h; β+ 17.8%; β– 38.4%) is well-known for its proficiency in positron emission tomography (PET) imaging and for its theranostic potential, on the basis of the preclinical and clinical cancer research of established bis(thiosemicarbazones), such as the hypoxia tracer ^64^Cu-labeled copper(diacetyl-bis(*N*4-methylthiosemicarbazone)], [^64^Cu]Cu(ATSM). Our labeling reactions proceeded in high radiochemical incorporation (>80% for the most sterically unencumbered ligands) showing promise of these species as building blocks for theranostics and synthetic scaffolds for multimodality imaging probes. The corresponding “cold” Cu(II) metalations were also performed under the mild conditions mimicking the radiolabeling protocols. Interestingly, room temperature or mild heating led to Cu(II) incorporation in the 1:1, as well as 1:2 metal: ligand ratios in the new complexes, as evident from extensive mass spectrometry investigations backed by EPR measurements, and the formation of Cu(L)_2_-type species prevails, especially for the **AN-Ph** thiosemicarbazone ligand (L^–^). The cytotoxicity levels of a selection of ligands and Zn(II) complexes in this class were further tested in commonly used human cancer cell lines (HeLa, human cervical cancer cells, and PC-3, human prostate cancer cells). Tests showed that their IC_50_ levels are comparable to that of the clinical drug cis-platin, evaluated under similar conditions. The cellular internalizations of the selected ZnL_2_-type compounds Zn(**AN-Allyl**)_2_, Zn(**AA-Allyl**)_2_, Zn(**PH-Allyl**)_2_, and Zn(**PY-Allyl**)_2_ were evaluated in living PC-3 cells using laser confocal fluorescent spectroscopy and these experiments showed exclusively cytoplasmic distributions.

## Introduction

Thiosemicarbazones (TSCs) and corresponding d-block or p-block thiosemicarbazonato metal complexes (MTSCs) have attracted wide research interest due to their antifungal,^1,2^ antiviral,^3,4^ or antineoplastic^5,6^ properties. Their applications in molecular imaging for Positron Emission Tomography have been explored since the observation of hypoxia selectivity in aliphatic derivative ^64^Cu-labeled copper(diacetyl-bis(*N*4-methylthiosemicarbazone)], [^64^Cu]Cu(ATSM), and several other Cu-based radiotracers (cyclic, or acyclic) have been investigated preclinically as theranostics.^7,8^ The occurrence of hypoxia in tumors inversely affects the prognosis and treatment progression in therapy-resistant cases: radiotherapy is generally associated with the production of reactive oxygen species (ROS) and the local pO_2_ level.^9^ The hypoxia-selectivity of metallo-drugs structurally similar to Cu(ATSM) remains a matter of lively investigations, and it is likely that after decomplexation *in vivo*, the free ions follow the copper metabolism.^10,11^

The research interest in the chemistry of thiosemicarbazones has been sustained over the past two decades, and examples of intrinsically fluorescent derivatives (with higher kinetic stability compared to that observed for other members of the TSCs compounds family, especially those incorporating flexible and aliphatic frameworks) have been reported.^12,13^ The inclusion of an intrinsically fluorescent aromatic backbones opens the way for versatile multimodal imaging agents based on thiosemicarbazonato metal complexes.^14^ Interestingly, a certain degree of hypoxia selectivity has been previously observed in ^68^Gallium-radiolabeled bis(thiosemicarbazonato) complexes with general formula ^68^[Ga]Ga(BTSC), anchored on ligands with naphthyl groups which provide a rigid, flat and aromatic backbone to the BTSC ligand frameworks.^15^ The hypothesis of the reduction of the metal, generally accepted when explaining the mode of action of Cu(ATSM) in hypoxic microenvironments can no longer be applied directly to the case of Ga-substituted BTSCs, as the reduction potentials of Ga(III) are outside of the biological range: as such, its observed trapping in hypoxic cells has been attributed to the targeting of iron species in these cancer cells.^15^ This apparent hypoxic behavior *in vitro*, along with the ability to chelate a wide range of metals, in a plethora of conformations and coordination modes, boosted our interest in thiosemicarbazones as synthetic building blocks for theranostic applications.

A very small number of mono(substituted) aromatic thiosemicarbazones prepared from precursors that include rigid aromatic frameworks have been reported.^12−18^ We recently reported the ^68^Ga(III) incorporation and cellular uptake behavior of a range of mono(thiosemicarbazones).^15b^ Additionally, the biological activity of tridentate N/N/S derivatives as antiproliferative agents, such as triapine (3-aminopyridine-2-carboxaldehyde thiosemicarbazone) has been reported, and assigned to their ability to generate ROS in the presence of iron, thus further enhancing the interest of mono(thiosemicarbazonato) ligands for theranostic applications.^16−18^

Thiosemicarbazones exhibit a number of coordination modes in binding to metal ions, acting as either bidentate, or tridentate ligands when their structure includes an additional donor atom. Thiosemicarbazones coordinate to the metal ion not only as bidentate and tridentate ligands but also as monodentate ligands.^1,2^ In the case of hybrid donors such as O/N/S species, the tridentate mode generally prevails and TSCs have been shown to form highly kinetically stable complexes in a 1:2 (ML_2_) fashion when M = Zn(II) or Ga(III), which present optical as well as coordination isomerism.^15,19^ Of particular interest so far have been the mono(thiosemicarbazonato) complexes of M = Zn(II), Ni(II), Cu(II), and Fe(II) comprising the acenaphthenequinone backbone (denoted AN), and a small number of such MTSCs complexes have been previously reported.^20^ A relatively small number of mono(thiosemicarbazone) ligands having the phenanthrenequinone (PH) backbone have been shown to form complexes for applications in catalysis: these were the ruthenium,^21^ nickel,^22^ or palladium^23^ complexes, and the ligands involved were used as agents for water analysis^24−26^ or for precious metals recovery.^27^ To the best of our knowledge, the published reports on simple phenanthrenequinone-based mono(thiosemicarbazones) are even scarcer, especially in the context of their uses as antiproliferative^28−30^ or antibacterial agents.^31^

So far the application of rigid aromatic thiosemicarbazonato-based complexes for molecular imaging purposes has generally been limited to bis(substituted) acenaphthenequinone compounds, in a 1:1 metal to ligand fashion, where the bis(thiosemicarbazonato) ligands are coordinating to the metal through their N and S atoms, in a symmetric or asymmetric tetradentate manner. The resulting geometry of the complex changes with the nature of the metal and the occupation of the fifth coordination position is possible for Zn(II), Ga(III), and In(III) giving rise to generally square pyramidal structures, whereas for Cu(II) and Ni(II) analogous complexes, the geometry exhibited by bis(thiosemicarbazones) was square planar.^7^

Interestingly, an *in vivo* imaging study reported a 9,10-phenanthrenequinone (PH) thiosemicarbazone labeled with ^61^Cu (a positron emitter with *t*_1/2_ = 3.33 h, β+: 62%, E.C: 38%) as a radiolabeled anticancer compound for malignant tissue imaging studies.^32^ The activity of octahedral Zn(II) mono(thiosemicarbazonato) complexes as antineoplastic compounds was evaluated.^33^ It was speculated that mechanism of action of these Zn(II) complexes in a number of different cancer cell lines involved the complex uptake followed by a transmetalation of the Zn(II) metal ions by Cu(II) ions present in lysosomes. The resulted Cu(II) complexes, formed intracellularly, then entered in a redox cycle that produced reactive oxygen species (ROS) and induced cellular apoptosis.^34^ The rarity of these reports on TSCs, along with the applications for radioactive labeling of phenanthrenequinone-based ligands encouraged us to explore other extended aromatic backbones, and to apply microwave technologies in the synthesis of the ligands as well as in the metal complexation protocols.

**Figure 1:**
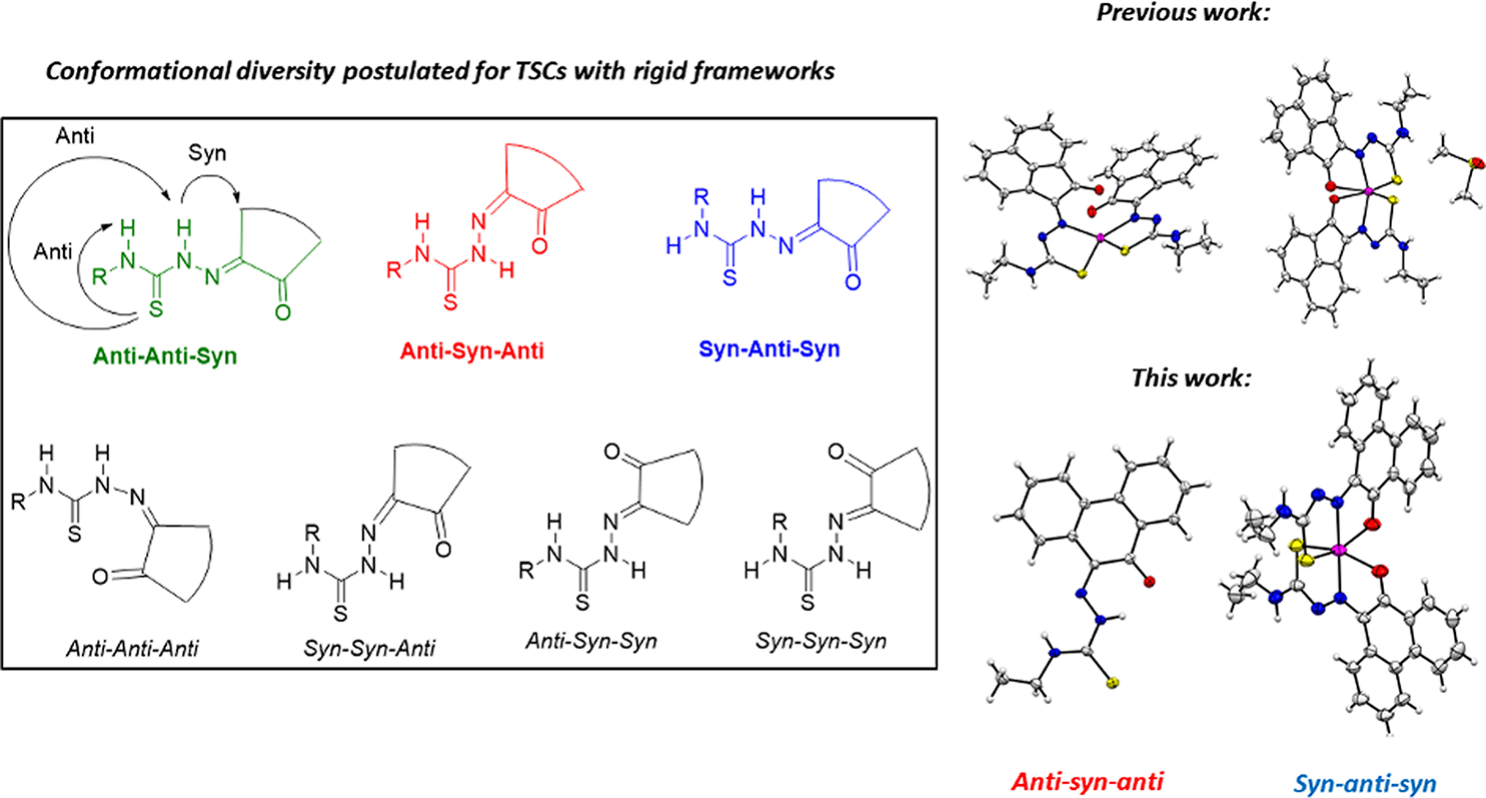
Overview of structural investigations in this, and previous work,^15b^ on monothiosemicarbazones with extended aromatic backbones (TSCs) highlighting the diversity of conformations identifiable in the solid state for this class of ligands and metal complexes.

Investigations into a new library of aromatic mono(thiosemicarbazone) ligands including those with aromatic backbones derived from aceanthrenequinone (denoted AA) and pyrene-4,5-dione (denoted PY) are the focus of this work. Here, a new series of ligands and corresponding metal complexes were prepared by a rapid and efficient microwave heating method that allowed us to reduce dramatically the reaction time, whist giving rise to the desired products in superior, or comparable, yields with the cases when conventional heating methods were used. We report hereby on optimized, sustainable, synthetic methods and functionalization protocols for the exocyclic N atom of the TSCs derived from the AA and PY quinones, and the more widely investigated acenaphthenequinone (AN) and phenanthrenequinone (PH), and closely investigate the diverse range of conformations for the TSC framework found in the ligands and complexes (Figure 1).

## Results and Discussion

### Microwave-Assisted Synthesis and Spectroscopic Characterization

**Scheme 1:**
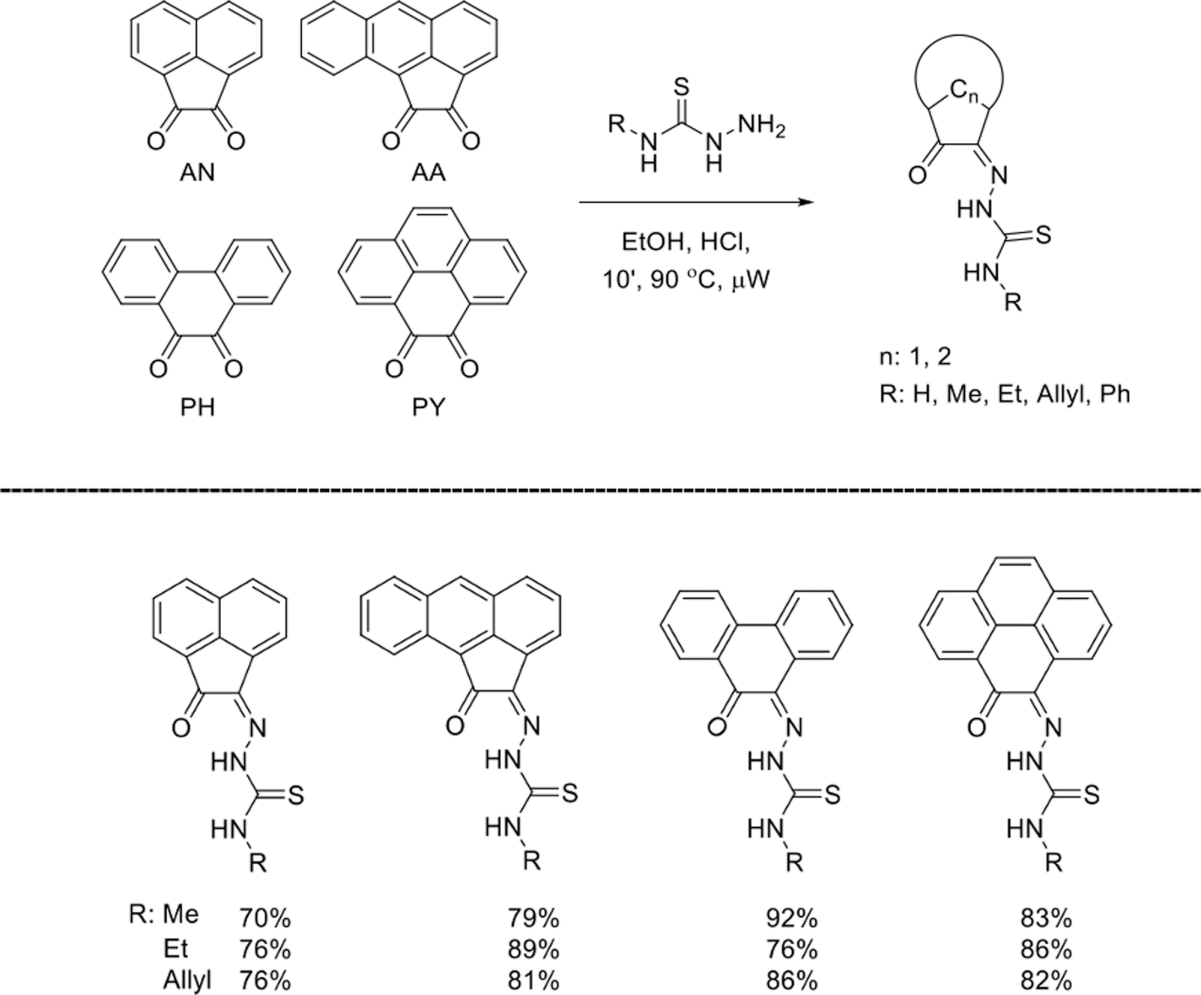
General Representation of a Library of Mono(Thiosemicarbazones) from Aromatic 1,2-Diketones and a Synthetic Method for These Ligands of Type HL Incorporating Extended Quinone-Based Backbones (Denoted AN, AA, PH, PY) The versatile incorporation of substituents at the exocyclic N’s was facilitated by the microwave-assisted irradiation, which proceeded under the general conditions: EtOH, cat. HCl, 10-20′, 90 °C.

The synthesis of a family of mono(thiosemicarbazones) ligands from corresponding diketone precursors having four different aromatic backbones, denoted AA, PH, PY, and including the ubiquitous acenaphthenequinone (AN), was performed and protocols were optimized by analogy with our previously developed methodology.^15^Scheme 1 outlines the general protocols carried out by microwave-assisted synthesis,^15^ and these were benchmarked against well-established conventional heating methods (Supporting Information). The vast majority of the synthetic methods employed for these metal complexes involved the conventional heating the ligands with acetates or chlorides of the metal ion of interest (Zn(II), Ni(II), Cu(II)) in a hydrophilic organic solvent (EtOH, MeOH, DMF, THF) for periods of min. 4–8 h.^20^ Optimized reactions yields were obtained for R = Me, Et, and Allyl substituted thiosemicarbazides (TSCs), whereby more than three reactions repeats were carried out; estimated yields for TSCs with R = H, Ph are also above 70% and details are given in Experimental Section and Supporting Information.

**Figure 2:**
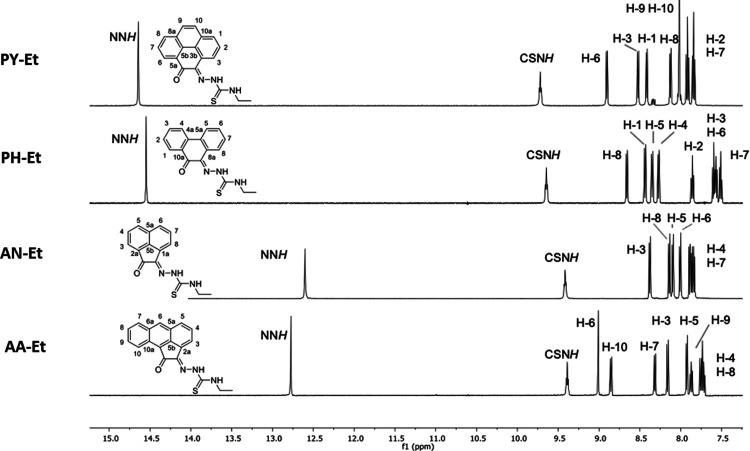
^1^H NMR (400 MHz, d^6^-DMSO) spectra showing the aromatic region of a selection of mono(4-ethyl-3-thiosemicarbazones) recorded at the room temperature.

The mono(thiosemicarbazone) ligands were characterized by standard analytical techniques, such as ^1^H NMR, ^13^C{^1^H} NMR, and mass spectrometry (detailed in Experimental Section and SI). Where available, these results were compared directly with the analysis available for the same compound synthesized from conventional heating and, in each case, the identity of the compound was confirmed by analogy with other compounds in this class, for the AN backbone and R = Me, Et, Ph, and Allyl.^14,15^ For the case of ^1^H NMR spectroscopic analysis recorded in d^6^-DMSO, the aromatic region for the ethyl derivatives with all four aromatic backbones showed comparable features to those reported for their acenaphthenequinone (AN)^13,15^ substituted TSC analogue (Figure 2). For example, the aromatic resonances are characteristic of each backbone and appear between ca. 7.50 and 9.00 ppm. The resonances assignable to the thiosemicarbazone groups are also comparable for all members of this ligand series, the amino proton appears as a triplet at ca. 9.50 ppm while the hydrazinic proton resonances were found further downfield. There is a considerable difference in chemical shift for the hydrazinic protons between the acenaphthenequinone and aceanthrenequinone derivatives (ca. 12.75 ppm) and pyrene-4,5-dione and phenanthrenequinone derivatives (ca. 14.5 ppm).

**Scheme 2:**
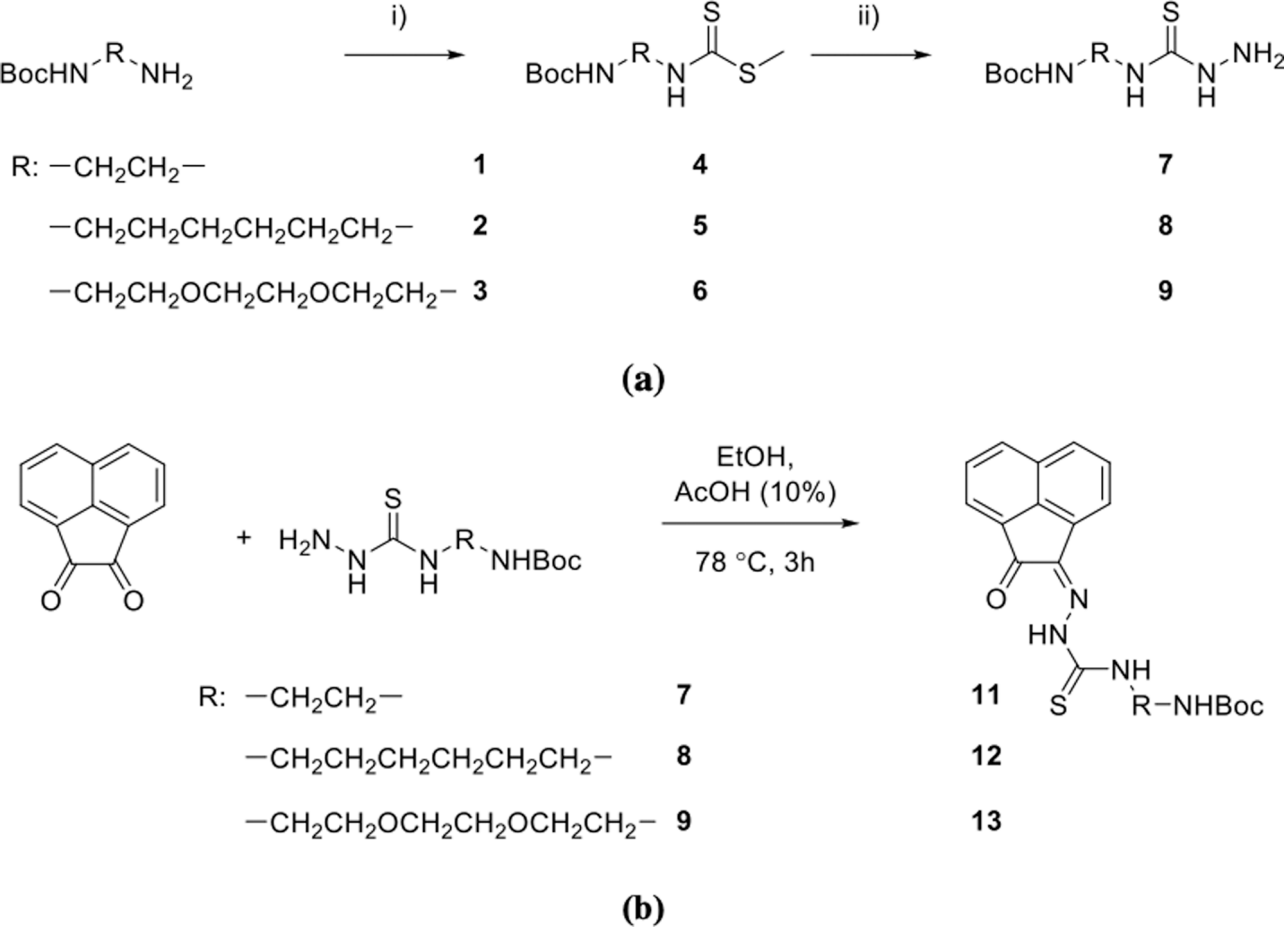
(a) Stepwise Representation of the Synthetic Route for the Preparation of Protected Thiosemicarbazides with AN Backbones Conditions were: (i) Et_3_N, CS_2_, CH_3_I, EtOH, 25 °C, 3 h; (ii) H_2_NNH_2_, EtOH, 78 °C, 3 h. (b) Synthesis of mono(thiosemicarbazone) acenaphthenequinone ligands with retention of the protecting amino group. Conditions: conventional heating (AcOH 10% v/v, cat., reflux, EtOH, 3.5 h, 58% yield). Compound **AN-10** (not shown) is the ^t^Boc-deprotected derivative of **AN-11**.

Furthermore, to explore the functional synthetic chemistry and the potential for further bioconjugation for these ligands, three different thiosemicarbazide precursors containing a terminal NH^t^Boc group were prepared from the corresponding protected diamines, as described in Scheme 2. Ligand modifications with linkers and protected amine groups were explored using these building blocks and several new thiosemicarbazides containing a terminal NHBoc group and the AN backbone were prepared using the microwave-assisted methodology under optimized conditions. Our previously reported procedure involving ethylenediamine conjugates of aliphatic thiosemicarbazones^15b^ was simplified and generalized to result in the formation of derivatives with the AN backbone, denoted **AN-11**, **AN-12**, and **AN-13** (Scheme 2). The synthetic protocol started with the corresponding protected diamines **1**–**3** and followed adapted strategies (see Experimental Section), where the reaction proceeded through the condensation of the amine with carbon disulfide in a basic ethanolic medium. The addition of methyl iodide to the reaction mixture formed the corresponding thiocarbamate intermediates that were then isolated on milligram scale (see Experimental Section and SI). The reaction protocol continued with the hydrazinolysis of the intermediate by reflux in ethanol to obtain the desired thiosemicarbazides **7**–**9** in moderate yields. In these reactions, the main challenge was posed by the hydrazinolysis step, which often led to formation of complex mixtures that required extensive recrystallization and/or chromatography separation, likely due to the occurrence of the well-known cyclization of thiosemicarbazone as highlighted previously for other thiosemicarbazone derivatives.^14,15,37^

The optimization of the purification conditions was necessary to obtain the desired product, especially by tuning the nature of the acid catalyst used. The deprotection of the amine by the removal of the ^t^Boc group necessitated just a few drops of conc. HCl, unlike the case of previously studied 2,3-butanedione thiosemicarbazones.^8^ Deprotection was confirmed by ^1^H NMR spectroscopy which showed the significant diminishing of the characteristic singlet at ca. 1.38 ppm (integrating for 9 protons) characteristic for the ^t^Boc group, and formation of a mixture of products. In the case where the removal of the catalytic HCl was attempted, in our hands, the treatment of the reaction mixture with basic solution (conc. NaOH) led to formation of traces of a urea-type derivative, which were isolated as traces of a pale-yellow crystalline byproduct (<5% yield). These single crystals were mechanically separated and characterized only by single crystal X-ray diffraction (CCDC 2218629 and SI).

Therefore, the use of a weak acid as a catalyst for the reaction to avoid the deprotection of the Boc group for the formation of desired product **AN-11** was employed. This optimized reaction was repeated several times, for the functional thiosemicarbazones depicted in Scheme 2, including the hexyl, **8** or 2,2′-(ethylenedioxyl) **9** using acetic acid (in a 10% v/v concentration in ethanol) as the acid catalyst. In this case, the desired products compounds **AN-11**, **AN-12**, and **AN**-**13** were obtained, in good yields (see Experimental Section), whereby the deprotection of the amino group did not occur. The presence of the hexyl chain or a PEG unit as a spacer in the last two mono(thiosemicarbazone) examples significantly changed the physical properties of the product. These linkers and functional groups enhanced the (notoriously limited) solubility of this class of acenaphthenequinone-based compounds in standard organic solvents. The derivatives **AN-12** and **AN-13** were initially obtained as oils and were separated from the starting materials by column chromatography in CH_2_Cl_2_/MeOH, which led to some hydrolysis, or precipitated as a yellow-colored solid by stirring in pentane overnight, and drying on standing at room temperature.

**Figure 3:**
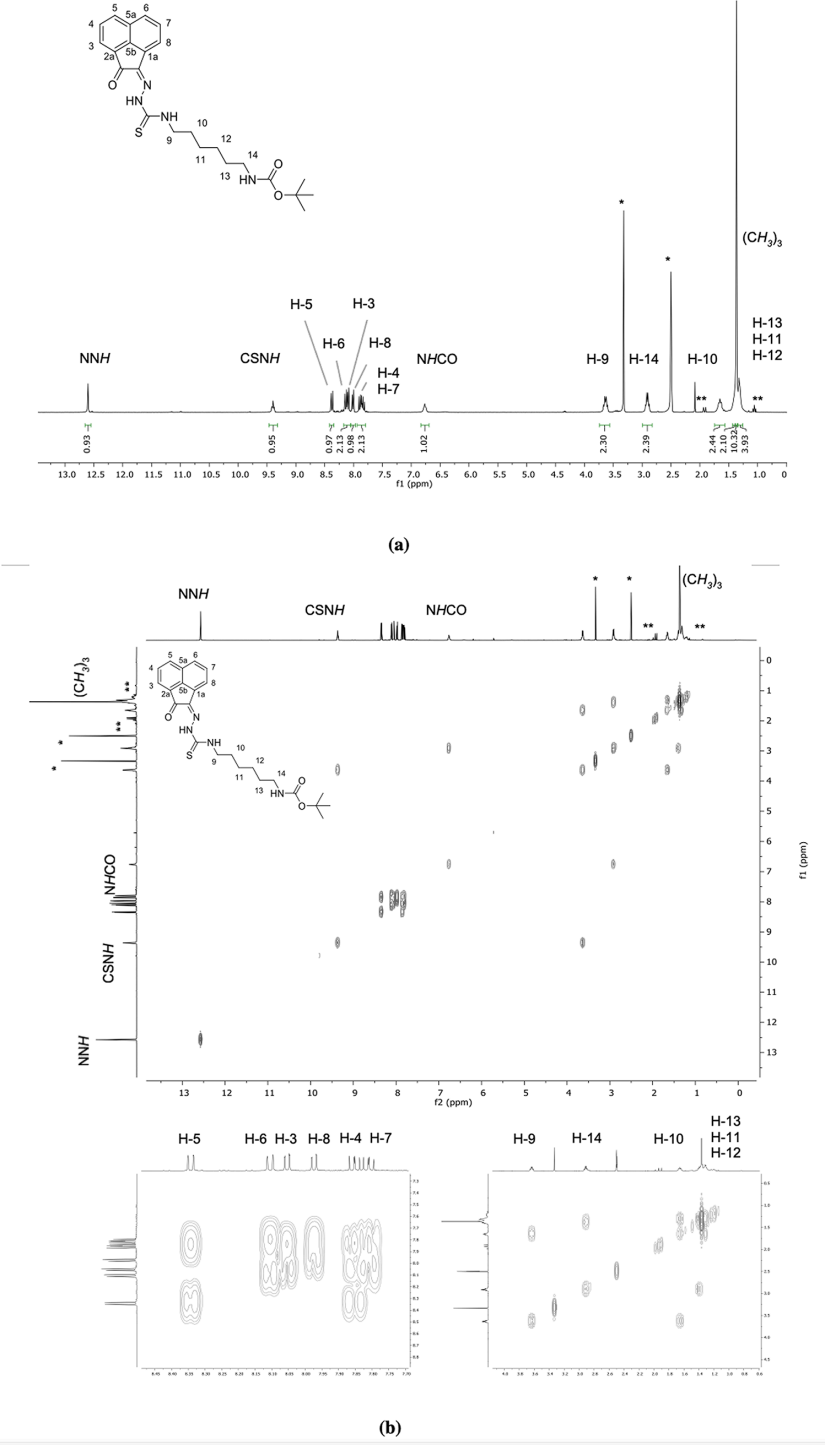
(a) ^1^H NMR spectrum (400 MHz, d^6^-DMSO) of compound **AN-12**. (b) ^1^H–^1^H COSY NMR spectrum (d^6^-DMSO) of compound **AN-12**. [*Residual deuterated solvent signals and water; **residual solvents and impurities traces.]

The linker-functionalized monothiosemicarbazones **AN-11**, **AN-12**, and **AN-13** (and a range of related TSCs with the derivatized backbones aceanthrenequinone (AA), phenanthrenequinone (PH) and pyrene-4,5-dione (PY)) were characterized by ^1^H and ^13^C NMR spectroscopy and HR ESI mass spectrometry (see Experimental Section). The ^1^H NMR spectra of these compounds in the aromatic region were comparable with those of the alkylic or arylic TSCs described above and also consistent with the previously reported compounds with AN backbones.^6,10^ Specifically, the first characteristic hydrazinic proton resonance appears at 12–13 ppm while the second characteristic amino proton appears upfield with respect to it, at 9–10 ppm. This last amino group in the organic chain typically appears as a triplet resonance. The substituent resonances are in the 1–4 ppm region with the presence of the characteristic t-butyl resonances of the protecting group at 1.31 ppm. The ^1^H NMR spectrum of compound **AN-12** is shown in Figure 3a. Assignment was carried out using ^1^H–^1^H COSY experiment (Figure 3b). The NH resonances, notoriously elusive, could be differentiated hereby because the amino proton showed a correlation peak with an alkylic proton (H-9). In addition, in the case of **AN-12**, crystals suitable for single-crystal X-ray crystallography were obtained by the vapor diffusion method, by dissolving the ligands in THF and layering with hexane and the structural features identified are described below.

### Incorporation of Bio-orthogonal Substrates in Functional TSCs

We were interested in the prospect of developing these TSCs as new synthetic scaffolds for the incorporation of peptides of relevance to theranostic applications *via* the construction of an amide bond within the framework.^19^ There is a limited number of TSCs based bioconjugates reported thus far, and these particularly focused on the introduction of a carboxylic acid moiety into a thiosemicarbazonato complex of a 2,3-butanedione moiety to include a benzoic acid in the backbone, which is amenable to then couple the ligand to peptides.^19c^

**Scheme 3:**
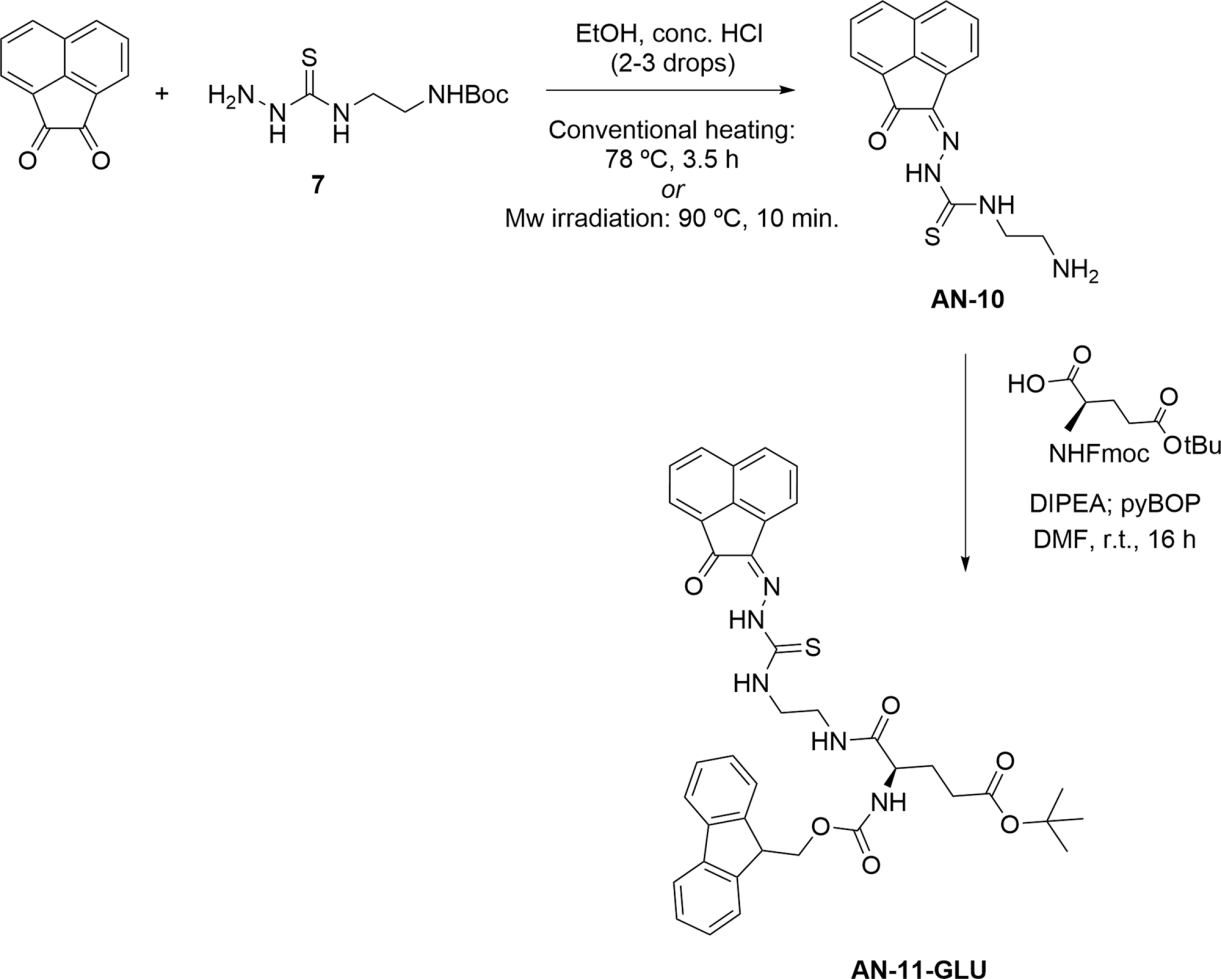
Protocol Applied at the Coupling of a Deprotected and Amino-Functionalized Thiosemicarbazone with l-Fmoc-Glu(O)O^t^Bu General conditions applied for the synthesis of the deprotected **AN-10** were: conventional heating (HCl cat., reflux, EtOH, 3.5h, 58% yield) or microwave-assisted irradiation (HCl cat., 90 °C, 20 min, both in ca. 60% yields). Note: Use of AcOH as a catalyst (10% v/v) in this step led to retention of ^t^Boc group and isolation of **AN-11** in 38% yield. The final product **AN**-**11-GLU** was obtained from the known compound **AN-10** as a yellow/orange solid in ca. 50% yield after purification, as described in the Experimental Section.

To explore the generality of our functionalization methods described above, we adapted the design elements to include a carboxylic acid in the thiosemicarbazonato compound within the AN-backbone functionalized and amine-terminated compounds described in Scheme 2. Our synthetic strategy involved the incorporation of the desired carboxyl functionality by a coupling reaction with a protected glutamine derivative through formation of an amide bond. Additionally, two protected side groups were maintained through a biorthogonal linker with the capability to be subsequently selectively deprotected. The coupling of the protected glutamine derivative was therefore evaluated as a proof of principle hereby using the l-Fmoc-Glu(OtBu)-OH a commercially available protected amino acid derivative (Aldrich). The experimental procedure consisted in the activation of the carboxylic acid group with pyBOP for 2 h at room temperature, followed by the addition of the deprotected amino-functionalized thiosemicarbazone derivative (**AN-10**), using our standard protocol^15b^ as shown in Scheme 3, Experimental Section, and SI. The success of the coupling with the protected amino acid expands the scope of the functional TSCs reactivity and opens a route for the attachment to targeting biomolecules and emergence of new bioconjugates.

### Structural Highlights in Thiosemicarbazones with Flat, Aromatic, and Extended Backbones

**Figure 4:**
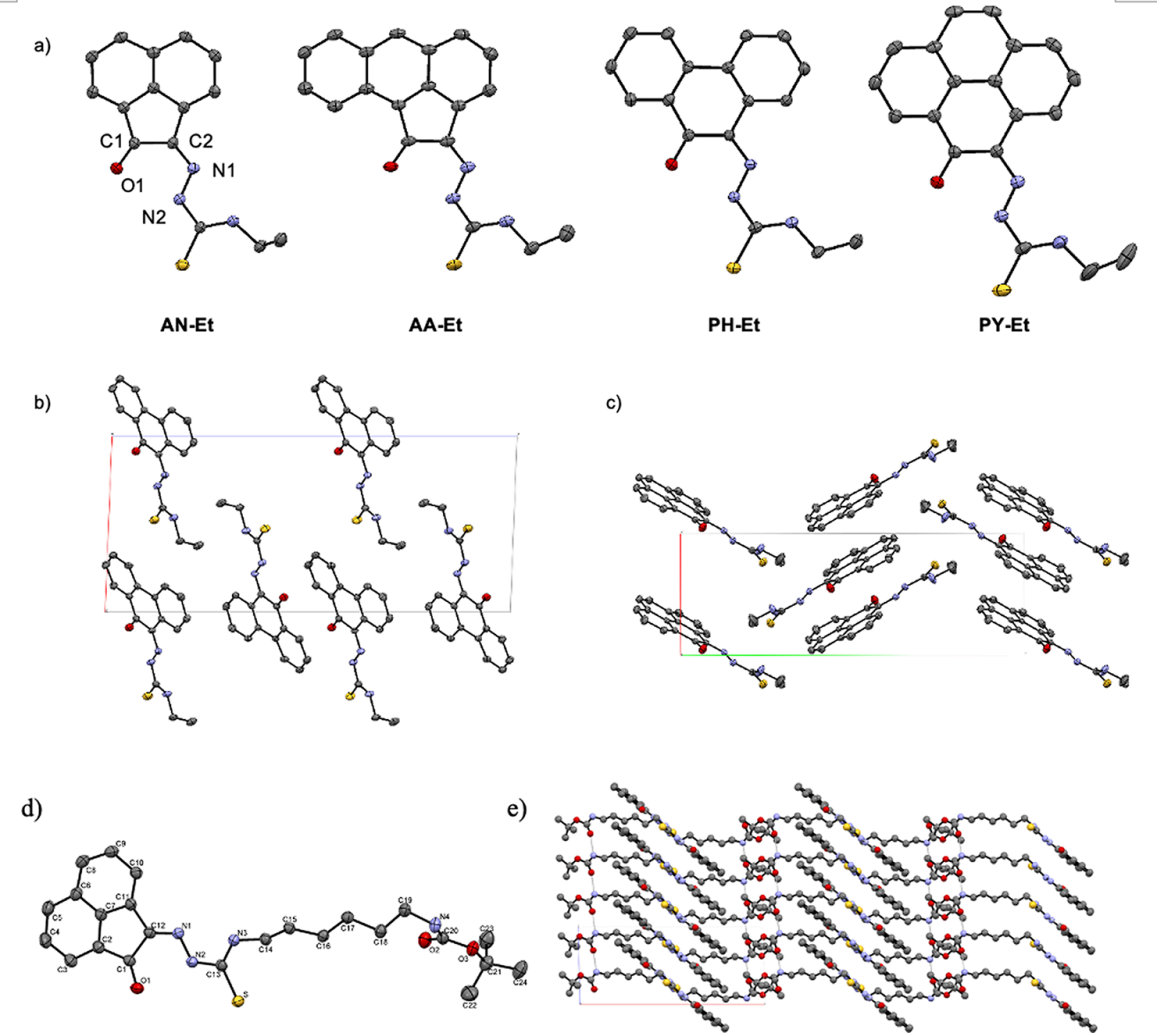
Single crystal X-ray analysis: (a) ORTEP representation with thermal ellipsoids represented at 50% probability of a range of aromatic mono(4-ethyl-3-thiosemicarbazone) ligands in the ethyl-substituted TSC series. Hydrogen atoms have been omitted for clarity. (b) Packing diagram of **PH-Et**. View along *b* axis. (c) Packing diagram of **PY-Et**. View along *c* axis. (d) ORTEP representation of the molecular structure of the free-base TSC ligand **AN-12** and (e) packing diagram of **AN-12** showing the unit cell along the *b* axis. Hydrogen bonds are showed as light gray lines. Atoms color: N: blue, S: yellow, O: red; C: gray. The .cif file of our previously reported^15b^ compound, denoted **AN-Et**, was downloaded from CSD (2131107). The image of the molecular structure of **AN-Et** is based on ref (15b). Copyright 2022 American Chemical Society.

**Figure 5:**
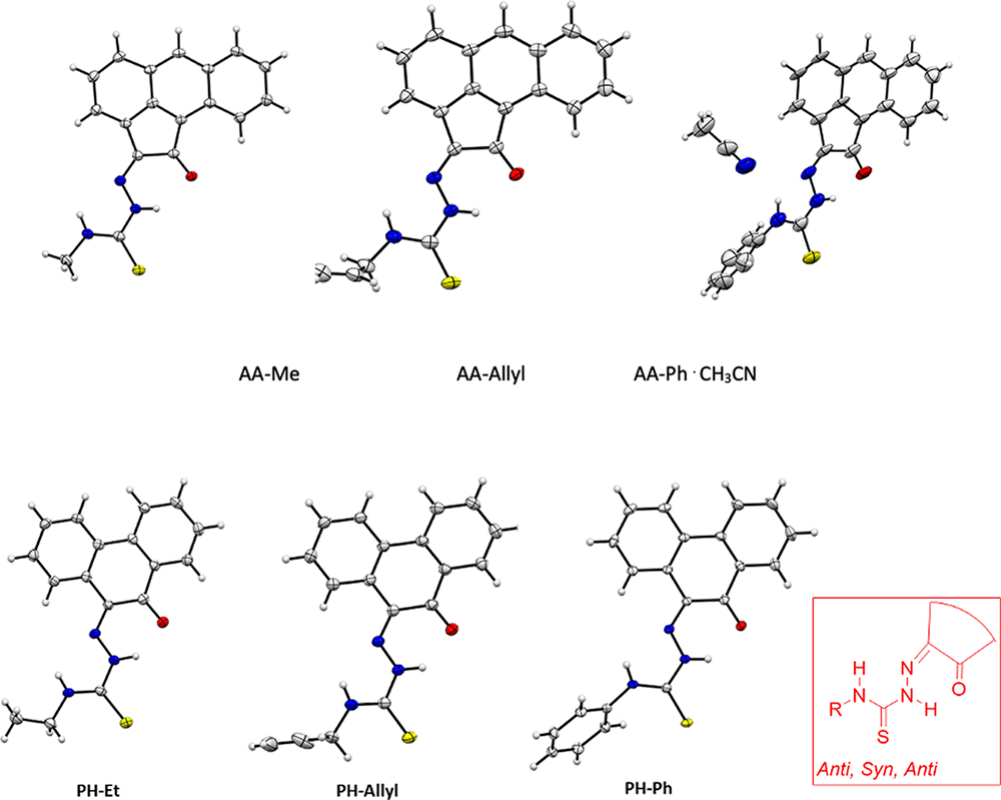
Single crystal X-ray analysis: ORTEP representation of a range of monothiosemicarbazones with AA and PH backbones all featuring *anti*–*syn*–*anti* conformations. Atoms color: N: blue, S: yellow, O: red; C: gray; H: white.

**Figure 6:**
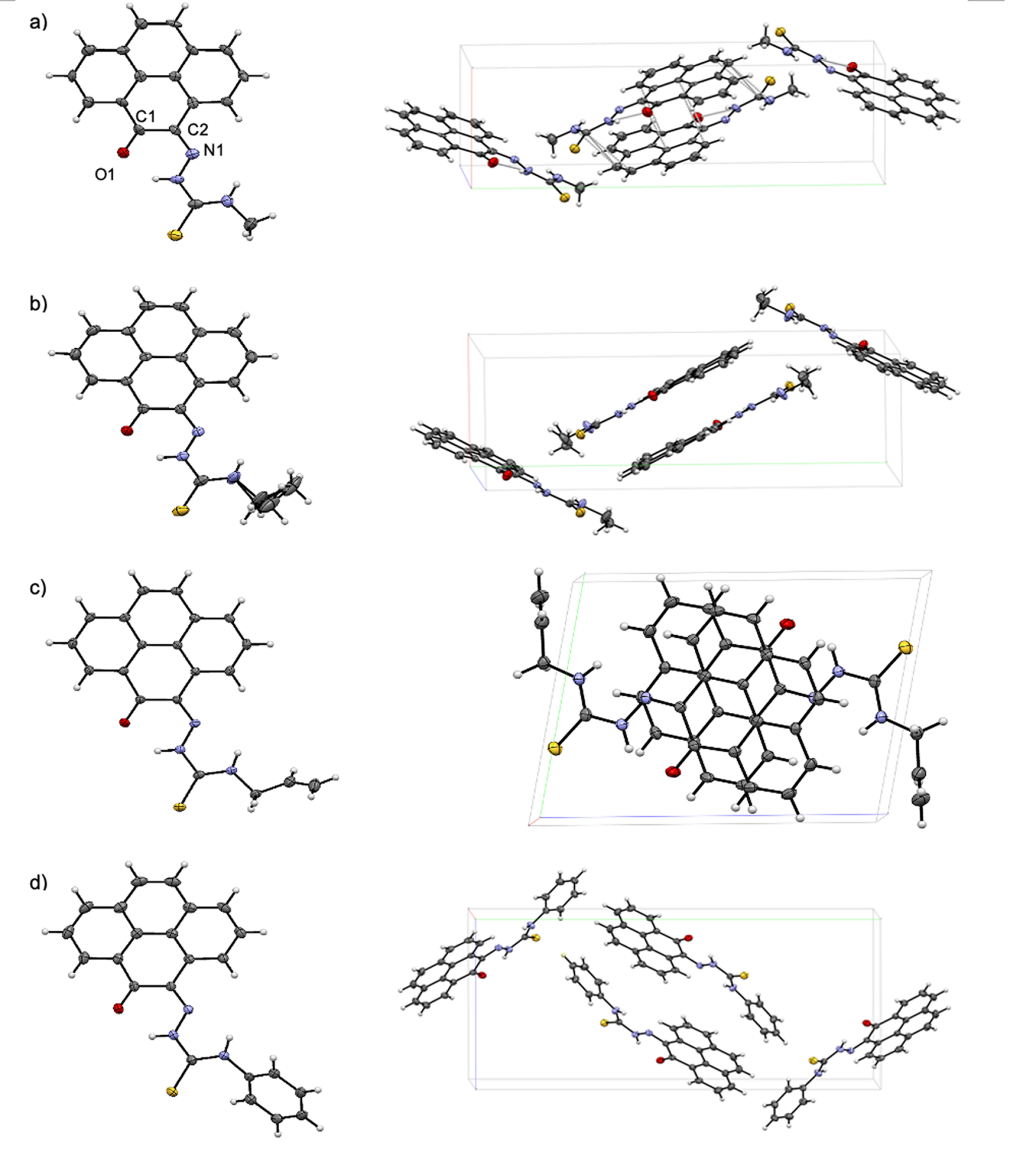
ORTEP representations of pyrene-4,5-dione mono(thiosemicarbazones) (left) and molecular structures showing the unit cells (right). The substituents at the exocyclic N’s are denoted as follows: (a) Methyl derivative, **PY-Me**, (b) ethyl derivative, **PY-Et** (where the exocyclic N’s Et group is disordered over two positions), (c) allyl derivative, **PY-Allyl**, (d) phenyl derivative, **PY-Ph**. Panels a and b show views along the *c* axis; panels c and d show views along the corresponding *a* axis. Atoms color: N: blue, S: yellow, O: red; C: gray.

Single crystals suitable for X-ray diffraction were obtained for the ligands by the slow diffusion of hexane into THF solutions of the ligands or from deuterated DMSO or CD_3_CN in NMR tubes. Generally, all TSCs frameworks show highly planar geometries and extensive networks of intramolecular hydrogen bonds are present in their 3D networks (Figures 4–6).

A close inspection of the selected molecular parameters for a range of compounds featuring ethyl groups at the exocyclic N atoms (Table 1) highlighted a subtle trend in key structural parameters, according to their collection in two groups: ligands featuring a 5-membered fused ring to the aromatic rings (TSCs with aceanthrenequinone and acenaphthenequinone backbones, denoted AA and AN, respectively), and those containing a 6-membered fused ring to the aromatic rings, i.e., with phenanthrenequinone (PH) and pyrene-4,5-dione (PY) backbones. The structural analysis structures of these ligands shows that generally the O1–C1 and C2–N1 distances are shorter in the first group (with the acenaphthenequinone AN and aceanthrenequinone AA backbones) while the corresponding C1–C2 and O1–N2 distances are larger. The shorter O1–N2 distance in the AN and AA-based derivatives (ca. 2.76 Å) with respect to the values found in phenanthrenequinone and pyrene-4,5-dione (ca. 2.56 Å) is most probably the reason of the deshielding of the hydrazinic proton as observed in the ^1^H NMR spectra shown in Figure 1. The values for O–C1–C2 and C1–C2–N1 angles are larger in the case of those compounds incorporating either the AN or the AA backbone.

**Table 1:** Structural Parameters for Mono(4-Ethyl-3-thiosemicarbazone) Aromatic Derivatives with Varying Backbones^a^

distance (Å)/angle (deg)	**AN-Et**	**AA-Et**	**PH-Et**	**PY-Et**
O1–C1	1.223(2)	1.2264(18)	1.239(4)	1.2373(19)
C1–C2	1.515(2)	1.520(2)	1.484(4)	1.487(2)
C2–N1	1.294(2)	1.2945(19)	1.316(3)	1.311(2)
O1–N2	2.771(2)	2.755(2)	2.555(3)	2.567(2)
O1–C1–C2	125.63(17)	124.68(12)	120.9(3)	120.99(14)
C1–C2–N1	128.14(17)	128.41(13)	124.3(3)	123.50(14)
O1–C1–C2-N1	3.5(3)	1.5(2)	4.8(4)	2.4(2)

a Note: The .cif file for the compound denoted **AN-Et** was downloaded from CSD (CCDC: 2131107) and included here for the structural comparison.^15b^

A selection of parameters of the new thiosemicarbazones with expanded frameworks are compared in Tables 1–3. The C1–C2 distance is of an average of ca. 1.48–1.49 Å across the series, and no significant differences were observed; the slight increase appears to be related to the bulkiness of the substituent in the R group of the thiosemicarbazone. As expected for sp^2^ carbon’s hybridization atom, the O–C1–C2 angles have a value in the region of 121° for all the compounds. The complementary angle between the C1–C2–N1 atoms have a value of ca. 124° and a trend to increase as seen for the C1–C2 distance, above. These molecules are all highly planar with the thiosemicarbazone, the exocyclic N substituent and the backbone all within the same plane with negligible deviations from planarity. The exception is the phenyl derivative where the **PY-Ph** aromatic ring is out of the plane formed by the backbone and the thiosemicarbazone unit by an angle of 56.62°. The distance between planes in the solid packing is close to 3.2 Å except for the **PY-Ph** derivative where this distance is larger, ca. 3.56 Å, due to the displacement of the Ph ring out of the plane. For the allyl-substituted compounds, the bond distances and angles are within the expected range (Table 3) and compare well with the crystallographic data for the mono(4-allyl-3-thiosemicarbazone) butane-2,3-dione^20^ and with our previously reported structures of ****AN-Ethyl****, **AN-Allyl**, and **AN-Phenyl**.^15b^ The O–C1–C2 and C1–C2–N1 angles are, however, smaller for mono(4-allyl-3-thiosemicarbazone) butane-2,3-dione than in any of the aromatic analogues investigated hereby. This can be attributed to the relative *E* configuration of the keto and imino groups along the C1–C2 bond, as the free rotation is allowed in the butane-2,3-dione derivative and rigidified in this series of mono(thiosemicarbazones). Overall, the O–C1 and N1–C2 distances are larger in species presenting the six-membered ring while the C1–C2 distance is shorter compared to the five-membered containing compounds. Furthermore, the O–C1–C2 and C1–C2–N1 angles are larger in derivatives presenting the six-membered cyclic ring fused to the aromatic group. The crystal structures for the functional derivative denoted **AN-12** was also obtained (Figure 4). The disposition of the thiosemicarbazone substituent with respect to the backbone is analogous to that of the ethyl-functionalized compounds, above, and the intramolecular hydrogen bond also features between O1 and N2 as in the entire range of monoTSCs derivatives. The nature of the R group does not seem to affect significantly the structural parameters in this series, and the observed values are all highly comparable to other acenaphthenequinone derivatives previously investigated.^15^ The presence of an intramolecular hydrogen bond between the oxygen and nitrogen (N1) atoms can also be observed in **AN-12**. The bond distances are in the same range as those highlighted above compounds and especially close for **AN-12** and its known **AN-Et** analogue.^15^

**Table 2:** Selected Bond Distances and Angles for Pyrene-4,5-dione Thiosemicarbazones Derivatives

distance (Å)/angle (deg)	**PY-Me**	**PY-Et**	**PY-Allyl**	**PY-Ph**
O–C1	1.2537(3)	1.2373(1)	1.2355(17)	1.2328(15)
C1–C2	1.4806(4)	1.487(2)	1.4899(18)	1.4968(16)
N1–C2	1.3172(3)	1.3113(1)	1.3078(17)	1.3043(16)
O–C1–C2	121.0(6)	120.99(14)	120.74(12)	120.63(11)
C1–C2–N1	123.1(6)	123.50(14)	123.65(12)	124.04(11)

**Table 3:** Comparison of Selected Crystallographic Parameters for Mono(4-Allyl-3-thiosemicarbazones) with Different Backbones

distance (Å)/angle(deg)	**AA-Allyl**	**PH-Allyl**	**PY-Allyl**
O–C1	1.227(2)	1.238(2)	1.2355(17)
C1–C2	1.518(2)	1.491(2)	1.4899(18)
N1–C2	1.293(2)	1.308(2)	1.3078(17)
O–C1–C2	124.6(1)	121.0(1)	120.74(12)
C1–C2–N1	128.2(1)	123.9(1)	123.65(12)

A close inspection of the unit cell fragments and corresponding 3D packing diagrams (as highlighted for some representative examples depicted in Figures 4e and 6), all the ligands are arranged in the solid state in zigzag orientations and present interactions with molecules in the planes above and below the aromatic core. The solid-state packing of these compounds revealed the presence of short contact interactions between the different molecules. The distance between planes and the aromatic character of these compounds point to the presence of π–π interactions and observing the structures, the character could be attributed to parallel displaced π–π interactions except for the phenyl derivative with the PY-backbone, that also presents perpendicular *y*-shaped interactions, analogous to those found for other pyrene-based derivatives of interest to targeting cell nucleus and acting as intercalators in DNA.^21−23^ The packing diagram of **AN**-**12** showed that the CO groups in the acenaphthenequinone units are facing each other in a zigzag disposition with short contacts between the sulfur and C2, and there are close, and extended, intermolecular hydrogen bonds between the CO group of the Boc group and the NH of the NH^t^Boc group of a “neighboring” molecule in the unit cell.

### Microwave-Assisted Metalation Reactions with Zn(II) Acetate

The formation of a small number of mono(thiosemicarbazone) complexes of d-block metals has been reported to proceed under conventional heating, often involving prolonged reflux conditions, as highlighted in the Introduction.^20−22^ Furthermore, we showed the preferential formation of acenaphthenequinone mono(thiosemicarbazonato) complexes of **AN-Et**, **AN-Allyl**, and **AN-Ph** in a 2:1 ligand: metal fashion for M = Zn(II) and Ga(III), where the ligand coordinated to the metal in a tridentate manner arranged in a distorted *mer–mer* configuration. Those Gallium(III) compounds have been described by us in the context of “cold” and “hot” gallium complexes formation, i.e., under thermodynamic *vs* kinetic control, respectively, and some of their analogous Zn(II) complexes were characterized structurally.^15b^

**Scheme 4:**
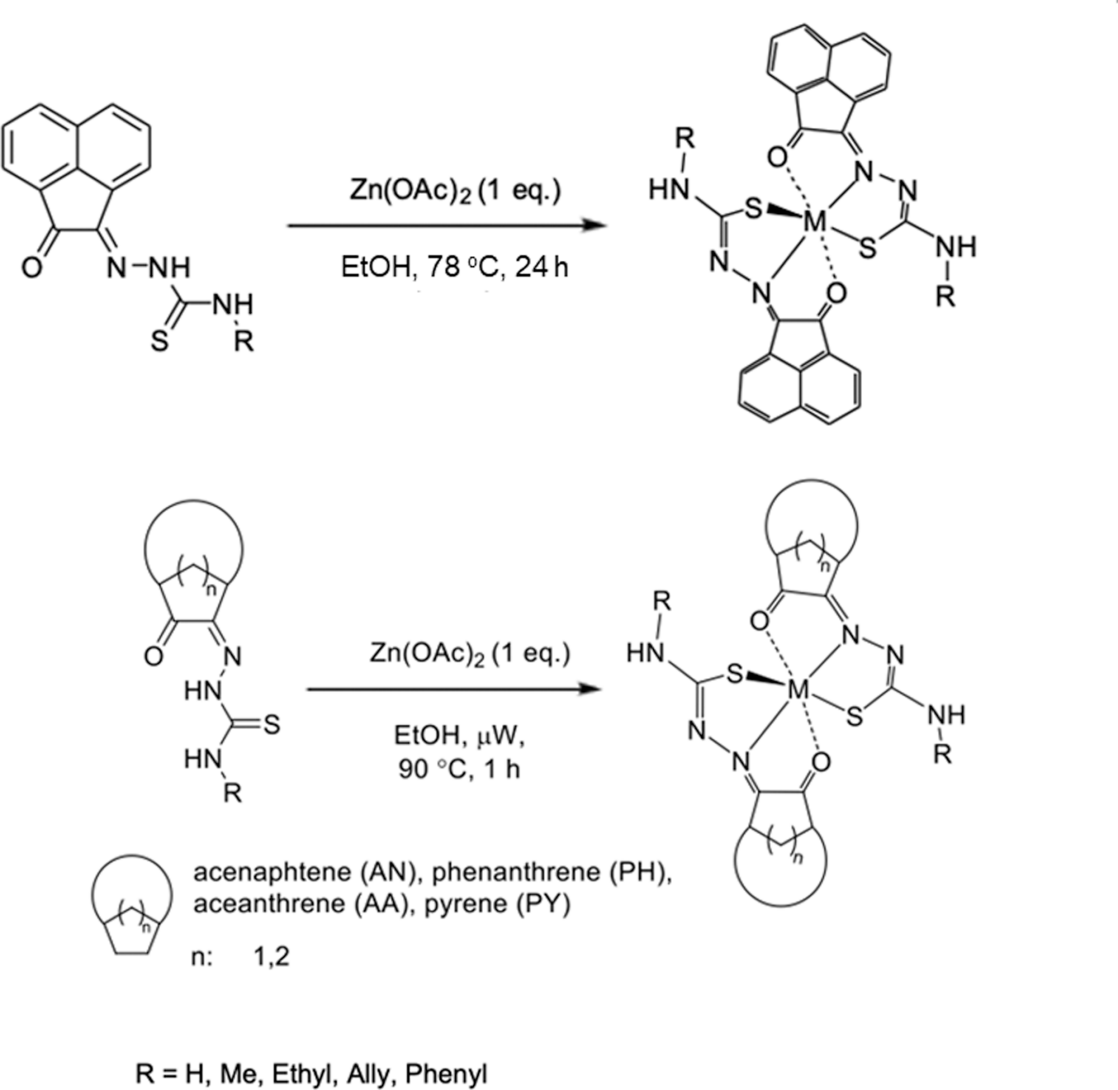
Generalization of the Metallation Reactions Approaches to Zn(II) Monothiosemicarbazones: Prolonged Conventional Heating *vs* Microwave-Assisted Synthesis of Zn(II) Mono(Thiosemicarbazonato) Complexes Further details are given in Experimental Section.

**Figure 7:**
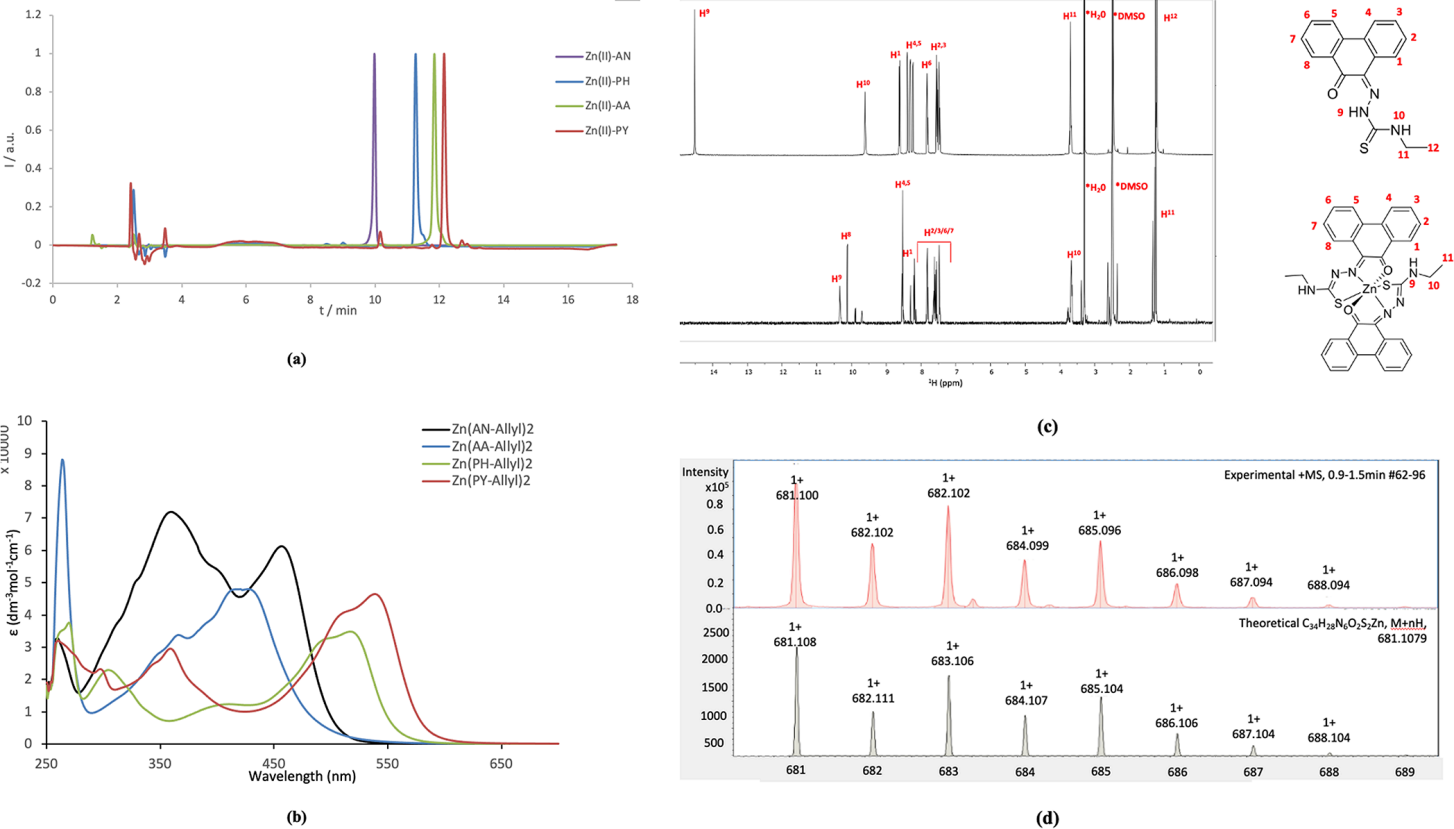
(a) A comparison of the reverse phase HPLC chromatography of Zn(II) mono(4-allyl-3-thiosemicarbazonato) complexes (UV–vis detection, 280 nm, samples injected from DMSO:H_2_O mixtures). (b) UV–vis spectra of Zn(II) mono(4-allyl-3-thiosemicarbazonato) complexes, recorded in DMSO (100 μM conc.). (c) ^1^H NMR (400 MHz, d^6^-DMSO) of the phenanthrenequinone (PH) substituted mono(4-ethyl-3-thiosemicarbazones (**PH-Et**) and of the corresponding Zn(II) mono(4-ethyl-3-thiosemicarbazonato) complex Zn(**PH-Et**)_2_. (d) ESI^+^ mass spectrometry of the Zn(II) mono(4-ethyl-3-thiosemicarbazonato) complex Zn(**PH-Et**)_2_.

We adopted new metalation strategies based on conventional as well as microwave-assisted irradiation protocols for the formation of a range of new thiosemicarbazonato complexes of Zn(II) for the new ligands featuring rigid and extended aromatic backbones. The microwave-assisted metalation was successfully applied, and optimized with respect to our earlier studies,^15b^ to yield the thiosemicarbazone ligand featuring H as the substituent of the exocyclic N’s as additionally to the ethyl and allyl mono(thiosemicarbazones) ligands incorporating the extended backbones denoted AA, PH, or PY. For selected ligands (with R = H, Me, Et, and Allyl), the corresponding Zn(II) complexes were also obtained by applying both conventional heating and microwave irradiation methods side-by-side, and in a range of ligand: metal ratios, to optimize the Zn(OAc)_2_ metalation as shown in the Experimental Section and SI. The use of microwave irradiation for the Zn(II) metalation reactions technique reduced considerably the reaction time needed in the preparation of these derivatives, and the ZnL_2_ species emerged preferentially after some extremely straightforward protocol. For the **AA-Ph**, **PH-Ph** and **PY-Ph**, the low solubility of the resulting metal complexes in common organic solvents prevented the spectrochemical characterization and unequivocal identification, and further studies are in progress in our laboratories. For the case of the TSC ligands featuring AN, AA, PH, and PY backbones and substituted with R = H, Ethyl, and Allyl, the Zn(II) complexes obtained after the microwave reaction emerged generally as orange to red colored solids in yields, ranging from 50–95%, with minimum purification being necessary (see Experimental Section). We found that mild and highly reproducible synthetic routes developed here led to a new class of Zn(II) complexes in ca. 90–95% purity by HPLC. A color change was observed during the synthetic process and HPLC analysis (with UV detection at 280 nm, as well as 450 nm), as well as UV–vis spectroscopy, were used to monitor the complex formation (Scheme 4 and Figure 7a–b). The final products were fully characterized by ESI^+^ mass spectrometry and ^1^H NMR. Figure 7d shows a comparison of the ^1^H NMR spectroscopy and free ligand spectroscopy of **PH-Et** given for this ligand of type HL and its corresponding Zn(II) complex (of type ZnL_2_), and Figure 7e depicts the mass spectrometry assignment for the new Zn(**PH-Et**)_2_ complex, a representative member of this new family of Zn(II) mono(thiosemicarbazones). In line with our observations reported earlier for gallium(III) metalation,^15b^ reactions using either 1:1 or 1:2 Zn(II) to ligand ratio generally led to formation of the ML_2_ species, and no [ML(OAc)] or related species could be isolated and fully characterized for M = Zn(II).

For the ^1^H NMR spectroscopy conducted upon treatment of the ligands with Zn(OAc)_2_, in all cases, the disappearance of the NH proton from the hydrazine group was observed, indicating metal coordination. The aromatic region showed several overlapping multiplets for the resonances assignable to the H’s in the ligand backbones. The interpretation of ^1^H NMR spectra, however, often proved challenging: for AN backbone spectra showed the inequivalence of the two ligand units by NMR in d^6^-DMSO, which we assigned to optical isomerism in previous studies.^15b^ As previously described for Ga(III) and Zn(II) compounds, formation of coordination isomers for Zn(II) in C.N. Four with a distorted tetrahedral geometry (in the N/S/N/S environment), as well as in a pseudo-octahedral environment (O/N/S/O/N/S or O/N/S/S/N/O), are also possible; however, HPLC did not indicate any differences in solution for any of the Zn(II) complexes investigated, and only one single dominant species was found, which we assigned to the ZnL_2_ type derivatives. The general low solubility of this class of compounds (due to aggregation behavior in common organic solvents) prevented detailed NMR investigations and hampered full ^13^C{^1^H} NMR assignments, especially for the quaternary carbon resonances (see Experimental Section and Supporting Information).

The UV–vis and fluorescence spectroscopy in highly diluted solutions were performed to evaluate their potential as optical imaging agents (Figure 7). The summary of the UV–vis and fluorescence properties such as maximum absorption wavelength, maximum emission wavelength, Stokes shift (Δλ), and quantum yields (Φ), are given in the SI (Tables S2–S3 and Figures S10–S18). All the complexes with the exception of the Zn(**AN-Allyl**)_2_, which was reported earlier,^15^ and included hereby for a comparison, show absorption wavelength maxima in the visible region. The emission wavelengths are in the visible region at ca. 600 nm except for the complex having the pyrene-4,5-dione backbone, denoted Zn(**PY-Allyl**)_2_, which is at 542 nm. The Stokes shifts are large for all the complexes. As expected, the quantum yields are low for all the mono(thiosemicarbazonato) complexes with respect to other organic or inorganic fluorophores.^15d,15e^

### Structural Investigations of Zn(II) Complexes of Thiosemicarbazides with Extended Backbones

**Figure 8:**
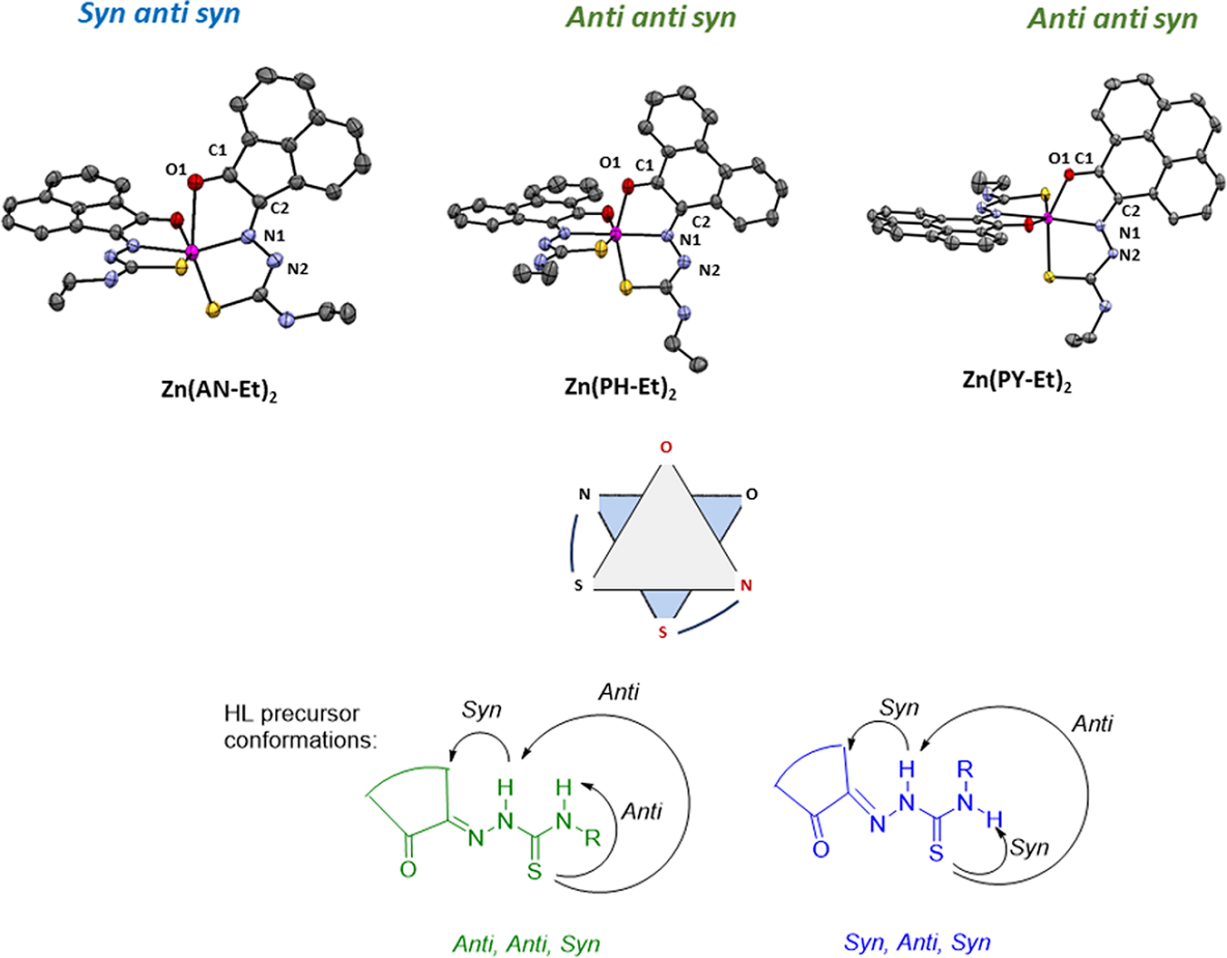
ORTEP representations for the single crystal X-ray diffraction structures of the Zn(II) complexes of ethyl-substituted TSCs showing distorted octahedral environment at the metal center. N: blue, S: yellow, O: red; Zn: magenta; C: gray. A schematic representation of their relative conformations is shown, and the structure of the octahedral isomer of Zn(**AN-Et**)_2_^15b^ (with the structure redrawn from the CSD-available cif file, 2130502) is given for comparison. The image of the molecular structure of Zn(**AN-Et**)_2_ is based on ref (15b). Copyright 2022 American Chemical Society.

Crystals suitable for X-ray diffraction for the Zn(II) mono(4-ethyl-3-thiosemicarbazonato) acenaphthenequinone and phenanthrenequinone complexes were obtained by slow diffusion of pentane in a THF/DMSO solution of the complexes, or from concentrated d^6^-DMSO solutions. For the analysis of Zn(**AN-H**)_2_ complex, crystallography studies indicated that the two structural isomers were present in the same asymmetric unit. These presented two different coordination geometries around the zinc center. The crystal structure of this zinc complex showed the expected planar geometry for each mono(thiosemicarbazone) ligand unit **AN-H** and a heavily distorted tetrahedral geometry (i.e., with Zn(II) in a N/S/N/S environment) *vs* the corresponding pseudo-octahedral geometry (where Zn(II) ion was found in the *mer–mer* O/N/S/S/N/O environment), as shown in Figures 8–Figure 10. DFT calculations (*vide infra*, and Supporting Information) showed that the optimized, equilibrated structure for the Zn(II) in the environment of two ligands (L^–^) displays an octahedral environment. Our previous studies for the structure determination on Zn(**AN-Et**)_2_ indicated that in the solid state, both a tetrahedral environment and an octahedral environment at the metal centre occurred for Zn(II) complexes,^15b^ and the occurrence of a distorted tetrahedral environment at the Zn(II) center was confirmed hereby for Zn(**AN-H**)_2_. The possibility of both tetrahedral and octahedral donor arrangement around the Zn(II) center here is similar to the case of the two isomers of the previously reported Zn(**AN-Et**)_2_ complex,^15b^ which were showing the *syn–anti–syn* orientations. This is due to the versatility of the zinc(II) ion in showing a range of coordination geometries with coordination numbers ranging between 4 and 6 in the presence of these rigid tridentate ligands featuring the hard, intermediate and soft donors, O, N, and S, respectively. DFT calculations were performed for **AN-H**, as well as **AN-Ph** ligands (of type HL) as well as for the corresponding complexes of type ML_2_ (for M = Zn(II), where L^–^ = monodeprotonated **AN-H** and **AN-Ph** thiosemicarbazonato ligands). These DFT-calculated geometries were in good agreement with solid state data for the complexes exhibiting octahedral geometry and the relevant molecular parameters are given in Supporting Information (Tables S5–S19).

**Figure 9:**
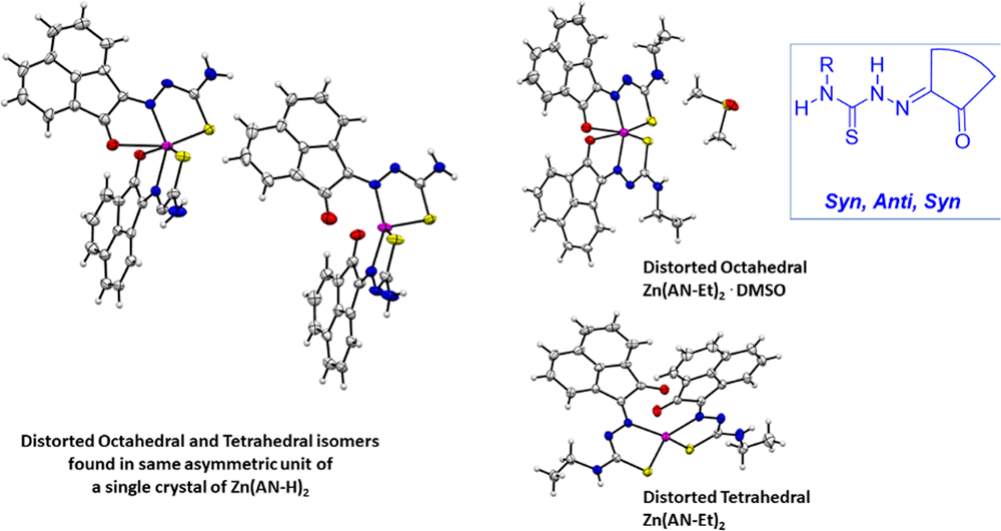
ORTEP representation of selected Zn(II) metal complexes. The main content asymmetric unit of Zn(**AN-H**)_2_ shows the distorted octahedral *vs* tetrahedral environments for Zn(II) in two different molecules found in the same asymmetric unit of this single crystal analyzed. The disordered solvent molecules present in the asymmetric unit were omitted. Thermal ellipsoids represented at 50% probability. N: blue, S: yellow, O: red; Zn: magenta; C: gray; H: white. Hydrogen atoms have been omitted for clarity. The previously determined structures of the pseudo-octahedral isomer of Zn(**AN-Et**)_2_ (with the structure redrawn from the CSD-available cif file, 2130502) and of the pseudotetrahedral isomer of Zn(**AN-Et**)_2_ (with the structure redrawn from the CSD-available cif file, 2130501) are given for the structural comparison. The images of the molecular structures of Zn(**AN-Et**)_2_ are based on ref (15b). Copyright 2022 American Chemical Society.

**Figure 10:**
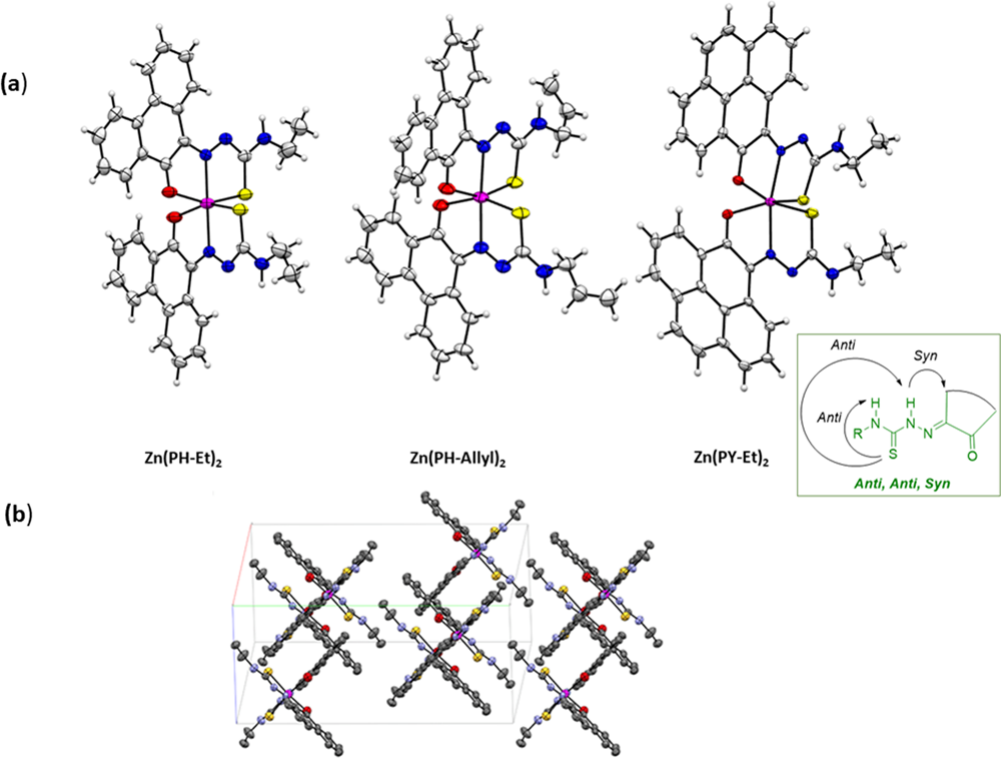
(a) X-ray diffraction molecular structures of a range of new Zn(II) complexes of TSCs ligands with extended backbones (ORTEP representations). Geometries show the pseudo-octahedral environments at the zinc centers for Zn(**PH-Et**)_2_, Zn(**PH-Allyl**)_2_, and Zn(**PY-Et**)_2_ complexes and the *anti-anti-syn* orientations for the TSCs substituents. Hydrogen atoms have been omitted for clarity. (b) Packing diagram of Zn(**PH-Et**)_2_, view along *a* axis. Atoms color: N: blue, S: yellow, O: red; Zn: magenta; C: gray; H: white.

The structures of a range of Zn(II) complexes are depicted in Figure 10 along with a fragment of the unit cell showing the 3D-packing arrangement for Zn(**PH-Et**)_2_ in the solid state (Figure 10b). Generally, the observed geometry corresponds to a distorted octahedral disposition of the O/N/S donor atoms with the Zn(II) metal in the center in the expected mer-mer geometry. This is similar to the octahedral isomer found for the synthesis of Zn(**AN-Et**)_2_ and no evidence of a tetrahedral isomer was found hereby by crystallography for any of the species analyzed and where the extended backbone was AA, PH, or PY-type. An overview of the structural parameters indicated that the estimated angle between the ligands’ mean planes is close to 90° (e.g., 89.9° for Zn(**PH-Et**)_2_), more so than the ca. 85.6° found in the previously reported complex Zn(**AN-Et**)_2_ which showed a heavily distorted octahedral geometryf around the metal center, as shown in the corresponding X-ray structure.^15b^

### Optical Spectroscopy and Cellular Imaging with Zn(II) Complexes

**Figure 11:**
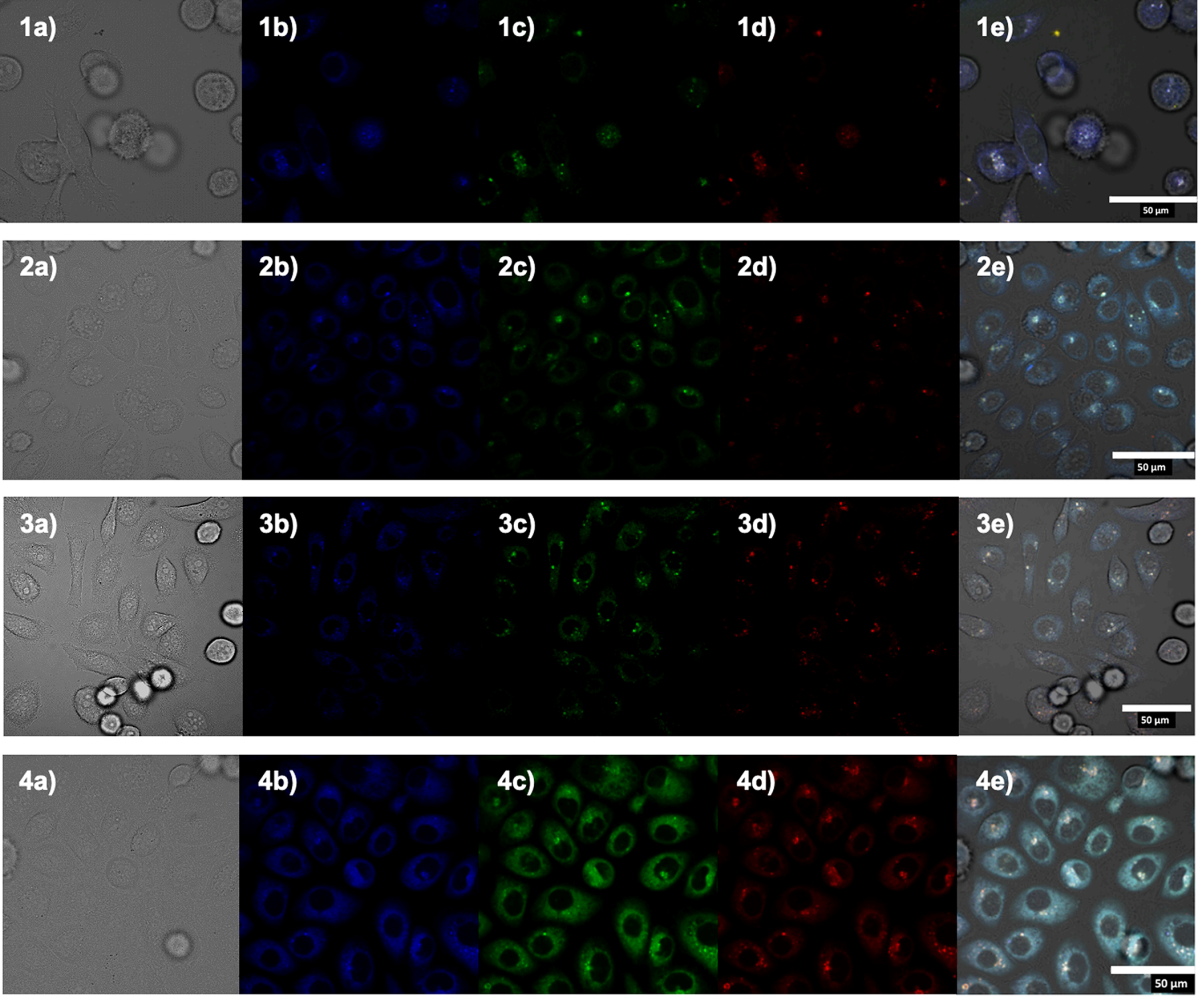
Single photon confocal microscopy images of compounds Zn(**AN-Allyl**)_2_ (1), Zn(**AA-Allyl**)_2_ (2), Zn(**PH-Allyl**)_2_ (3), and Zn(**PY-Allyl**)_2_ (4) in PC-3 cells after 20 min incubation at 37 °C, 100 μM in serum free medium (1% DMSO) λ_ex_ 488 nm, where (a) DIC channel, (b) blue channel (λ_em_ 420–480 nm nm), (c) green channel (λ_em_ 516–530 nm), (d) red channel (λ_em_ 615–650), and (e) overlay. (a–d) Scale bar: 50 μm.

The cellular uptake and cytotoxicity were evaluated for several ligands and Zn(II) complexes in two commonly used, cancer cells lines, HeLa and PC-3 cells. These are well established, commercially available from ATCC as obtained from human cervical cancer and human metastatic prostate cancer, respectively, and routinely used for cancer pathological mechanism studies and drug testing, including in our own previous investigation on related thiosemicarbazones.^15^ These tests were carried out for a subset of compounds which showed intrinsic fluorescence and most promising solubility in aqueous media (with 1% DMSO), aiming to ascertain their relevance for bioimaging assays using laser scanning confocal microscopy (Figure 11) and MTT assays following our standard protocols.^13−15^

The Zn(II) compounds investigated were Zn(**AN-Allyl**)_2_, Zn(**AA-Allyl**)_2_, Zn(**PH-Allyl**)_2_, and Zn(**PY-Allyl**)_2,_ (e.g., each incorporating the allyl substituent at the exocycic N’s and different aromatic backbones. In each case, it was observed that at 100 μM concentration (1% DMSO) these showed cellular uptake and fluorescent emissions were visible in the cells’ cytoplasm. The highest fluorescence intensity emission was obtained in the green channel (λ_em_ 516–530 nm), for excitation wavelengths of 405 or 488 nm (Figure 11). For the Zn(II) compounds with R = Et or Ph substituents at the exocyclic N’s, the relatively high concentrations needed to achieve sufficiently observable fluorescent emission led to considerable cellular damage, as well as precipitation. None of the free ligands show sufficient intrinsic fluorescence in cellular media at comparable concentrations. Meanwhile, the fluorescence intensity in the series Zn(**AN-Allyl**)_2_, Zn(**AA-Allyl**)_2_, Zn(**PH-Allyl**)_2_, and Zn(**PY-Allyl**)_2_ increased with the increase of the number of aromatic rings in the backbone. A certain degree of precipitation of the complex was still observed upon addition in serum-free and phenol-free RPMI medium, and therefore PBS washing protocols needed to be employed. This challenge could be potentially solved by enhancing the solubility by introducing the functional groups at the exocyclic N’s, which will be pursued in further investigations in our laboratories.

Furthermore, for a subset of free ligands with AN-backbone and several representative Zn(II) complexes, the 48 h MTT assay was performed in PC-3 and HeLa cell lines to evaluate the IC_50_ values and to compare these with the well-established cytotoxic behavior, previously reported for related bis(thiosemicarbazonato) complexes and the clinical drug cis-platin.^13,14^ The IC_50_ values of the ligands featuring the AN-backbone and simple substituents as well as those of the cis-platin treatment groups were obtained following incubation for 48 h (Table S4, Figure S70). The Zn(II) compounds investigated generally showed lower cytotoxicity than the free ligands with the IC_50_ value of ca. 50 μM (Figures S72–S73). For [Zn(**AN-Et**)_2_] the IC_50_ value is (61.58 ± 5.38) μM, whereas for [Zn(**AA-Et**)_2_] the IC_50_ value was (44.11 ± 2.08) μM. The corresponding [Cu(**AN-Et**)_2_] (synthesized via the microwave protocol described in Experimental Section, and discussed below) showed a IC_50_ value of (2.25 ± 0.01) μM, highly comparable to that seen in the free ligands. The IC_50_ values of the cis-platin treatment groups were (31.28 ± 9.38) μM in HeLa cells, and (30.64 ± 3.26) μM in PC-3 cells. Therefore this subset of compounds, analyzed for proof-of-concept (whether free ligands or Zn(II) or Cu(II) complexes), showed a significant cytotoxicity in line with previous observations of related TSCs,^15^ and further, more detailed biological investigations are underway in our laboratories.

### Metalation Reactions with Cu(OAc)_2_ and Investigations by EPR Spectroscopy

To generalize the synthetic approach to other d-block metal ion incorporation into these TCSs, room temperature, as well as analogous microwave-assisted conditions, were applied to the reactions of the AN-backboned TSCs with Cu(OAc)_2_ in a variety of organic solvents (DMSO, MeOH or THF). However, the reaction outcome was not as straightforward as seen with Zn(II), as discussed below. A color change toward red-brown was observed in all cases upon mixing the starting materials and the products were observed by mass spectrometry but the reaction mixture contained decomposition products observed as dark residues in the product and several peaks in the mass spectrometry (see Supporting Information and below). This result would indicate that the synthetic method is metal-dependent, and it is clear that the Zn(II) complexation is significantly more thermodynamically and kinetically favorable. In this work, reactions of selected ligands (of relevance for the ^64^Cu-radiolabeling experiments, *vide infra*) were carried out with anhydrous Cu(OAc)_2_ and were explored at the room temperature, under the microwave irradiation or using mild conventional heating.

**Scheme 5:**
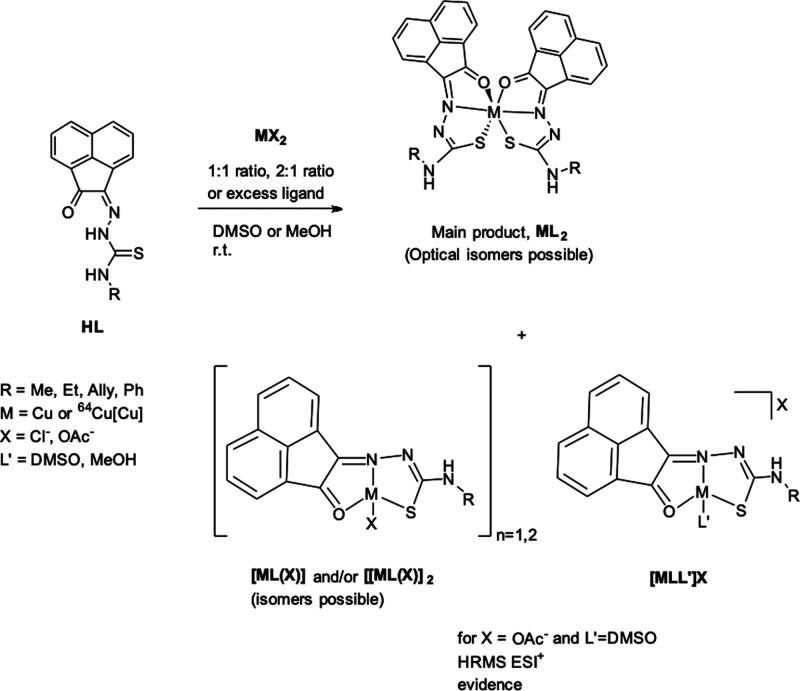
Overview of the Reactions Involving AN-Backboned Ligand and Proposed Main Species Formed As Evidenced from HR ESI^+^ MS Additionally, the presence of tetrahedral, as well as octahedral, environment Cu(II) centers, in addition to optical isomerism in the octahedral *mer–mer* complexes of type CuL_2_ cannot be discounted.

**Figure 12:**
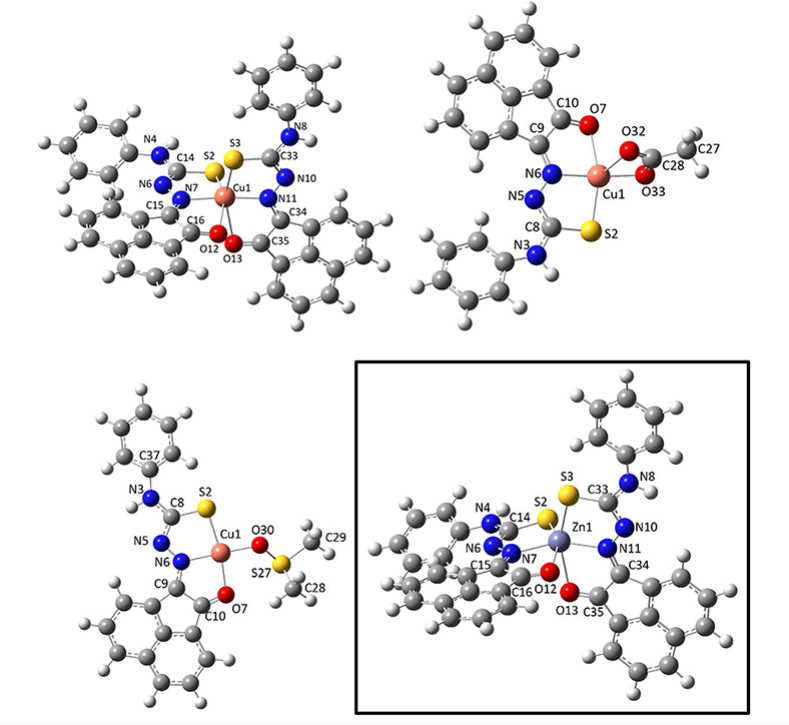
Overview of the DFT-optimized model structures for proposed Cu(II) complexes of the **AN-Ph** ligand and corresponding to main fragments found in HRMS ESI^+^. Inset: Optimized (relaxed) structure of Zn(**AN-Ph**)_2_ model structure. Atoms color: N: blue, S: yellow, O: red; Cu: pink; Zn: purple; C: gray.

Several reaction setups were explored, using either 1:1 or 1:2 molar ratios of metal to ligand, in order to obtain Cu(II) complexes from Cu(OAc)_2_, i.e., in processes carried out under thermodynamic control. The reaction mixtures and purified compounds were monitored by HPLC and extensive mass spectrometry. Copper complexes formed seem to have significantly lower kinetic stability in solution and with respect to acidic environment with respect to their Zn(II) counterparts. In the absence of X-ray diffraction, mass spectrometry was especially instrumental in pointing out the possibility of complexes showing 1:1, as well as 1:2, metal: ligand ratios, and this was consistent with observations from radio-HPLC investigations at the formation of new ^64^Cu complexes (under kinetic control). Scheme 5 gives a representation of our postulated formation of Cu(II) compounds (and corresponding isomers) under mild conditions, and Figure 12 shows the “relaxed” gas-phase calculations for the DFT calculated geometries for the **AN-Ph** ligand.

The postulated structures of the three main species formed, likely in equilibrium in solution additionally to the free ligands, as shown in Scheme 5, and evidence for the presence of these species was found by extensive high resolution ESI^+^ mass spectrometry investigations. The gas-phase DFT optimizations of the proposed monometallic Cu(II) species identified by HRMS ESI^+^ were carried out to identify whether or not such species would show thermodynamic stability in gas phase. Extensive molecular parameters data for the optimized geometries, their energies, and the corresponding HOMO–LUMO levels and corresponding Cu and Zn complexes of the **AN-Ph** ligand and the simplified **AN-H** variant are given in the Supporting Information. We postulate that the major component for reactions carried out under thermodynamic control is the CuL_2_-type species, while under kinetic control, the equilibrium between 1:1 and 1:2 metal:ligand species cannot be discounted (*vide infra*), additionally to the formation of CuL_2_-type species (and corresponding isomers/stereoisomers).

While the possibility of isomerism in CuL_2_ species is expected by analogy with Zn(II) structures discussed above, where the octahedral *vs* tetrahedral geometries are possible in the solid state, the structures of the Cu(II) complexes formed in these reactions could not be determined unequivocally in the absence of X-ray structure determinations for the compounds synthesized. Therefore, extensive EPR spectroscopy was used to shed light into the nature of these species in solution, as well as in the solid state, with an aim to probe for the possibility of the coexistence of multiple Cu(II) centers. The products of the reactions carried out under mild conditions between Cu(OAc)_2_ and HL ligands (in a 1:2 ratio, for AN backbone, and R = Me, Et, Allyl, Ph), as well as those emerging from the reaction conducted in a 1:1 ratio of Cu(OAc)_2_:ligand HL (where HL was **AN-Et**, **AN-Ph**) were analyzed, and corresponding EPR parameters (Figures 12 and S57, Supporting Information) and magnetic susceptibility behavior were evaluated (Figure S58, Supporting Information).

The species of interest for analysis by detailed EPR spectroscopy emerged from reactions carried out at the room temperature using either 1:1 or 1:2 metal:ligand ratios, as described in the Experimental Section. Samples analyzed by EPR were denoted: (A) Cu-**AN-Me**, the product from the 1:1 reaction Cu(OAc)_2_: **AN-Me** ligand, (B) Cu-**AN-Et**-a, the product from 1:1 reaction Cu(OAc)_2_:**AN-Et** ligand), (C) Cu-**AN-Et**-b, the product from 1:2 reaction Cu(OAc)_2_:**AN-Et** ligand; (D) Cu-**AN-Allyl**, the product from 1:1 reaction Cu(OAc)_2_:**AN-Allyl** ligand; (E) Cu-**AN-Ph**-a, the product from 1:1 reaction Cu(OAc)_2_:**AN-Ph** ligand, and (F) Cu-**AN-Ph**-b, the product from 1:2 reaction Cu(OAc)_2_: **AN-Ph** ligand. EPR spectra were first obtained for the powdered solids (A–F) listed above, as well as in fluid and frozen solutions, as described below. Spectra in neat DMSO as solvent showed no Cu hyperfine splitting when frozen. All samples above gave poorly resolved fluid solution spectra, which may derive from the inclusion of the acenaphthenequinone backbone of these ligands, and which could serve to slow the tumbling rate of the molecule in the relatively viscous DMSO solvent and cause a broadening of the spectrum.

We assigned this to the possibility that in solution an equilibrium between a number of Cu(II)-ligand species can occur, with the proposed geometries shown in Scheme 5. Additionally, a 7:1 *v*/*v* EtOH/DMSO mixture was used to produce well-resolved frozen glass spectra. These spectra exhibited three of four lower field, low intensity Cu-hyperfine resonances and more intense higher field features with no obvious Cu-hyperfine structure, which is consistent with a tetragonally elongated electronic structure for the Cu(II) center in each complex. The “parallel” region seems qualitatively diagnostic. The polycrystalline powder spectra do not provide much additional information. The absence of ^63,65^Cu hyperfine splitting indicates that the samples are magnetically concentrated.

**Figure 13:**
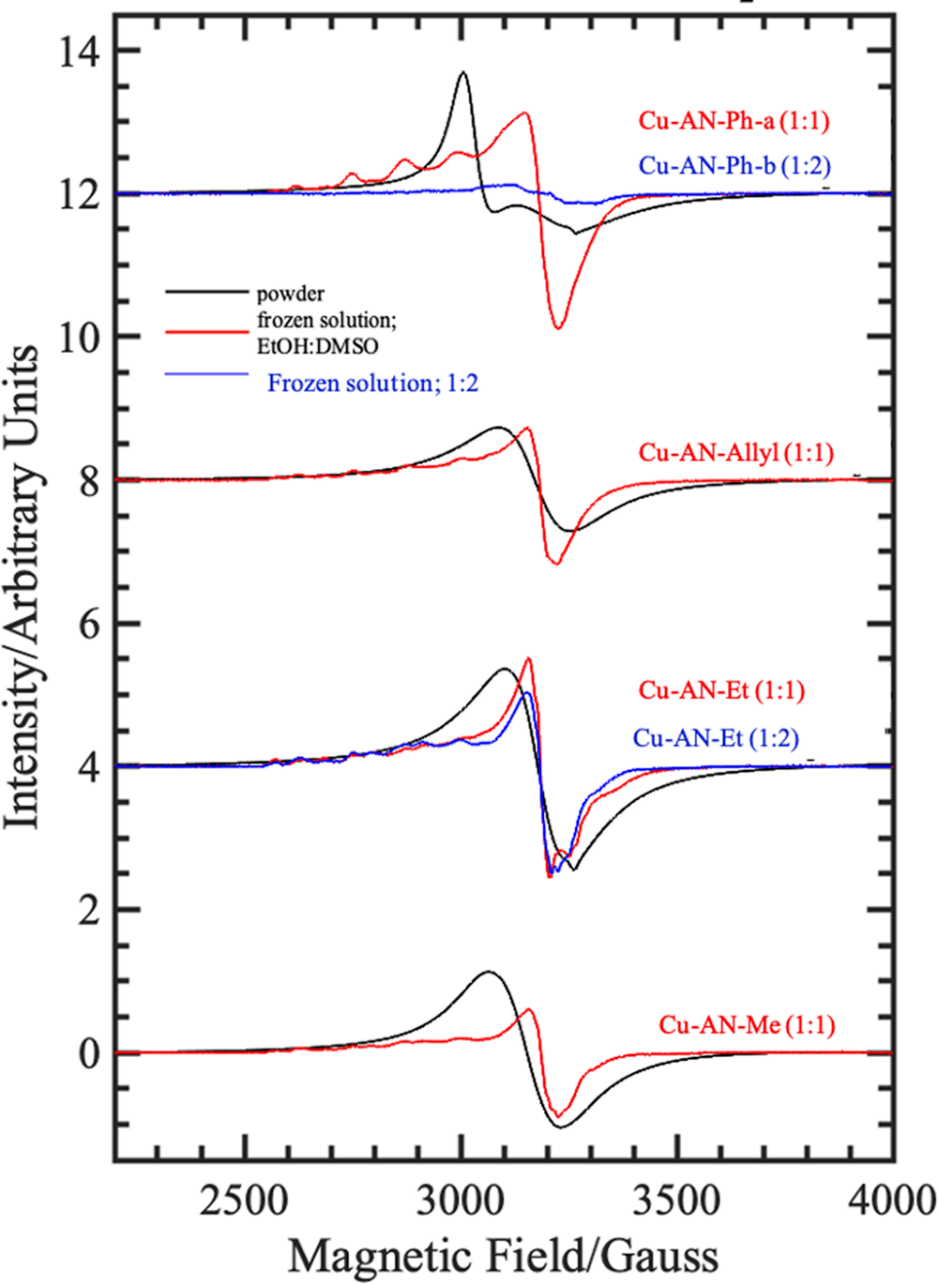
Cw X-band EPR spectra of: (A) Cu-**AN-Me**, (B) Cu-**AN-Et**-a, (C) Cu-**AN-Et**-b, (D) Cu-**AN-Allyl**, (E) Cu-**AN-Ph**-a, and (F) Cu-**AN-Ph**-b in frozen solutions (7:1 *v*/*v* EtOH:DMSO, red line) and as a polycrystalline powder (black line) at 20 K. Blue traces are products from 1:2 Cu: thiosemicarbazone ligand reactions, which are dominated by the CuL_2_-type species.

The spectra of frozen solution and polycrystalline powder spectra of samples (A)-(F), denoted Cu-**AN-Me**, Cu-**AN-Et**-a, Cu-**AN-Et**-b, Cu-**AN-Allyl**, Cu-**AN-Ph**-a, and Cu-**AN-Ph**-b, as shown in Figure 13. All the frozen glass spectra are of the tetragonally distorted type, and all except the 1:2 Cu:ligand complex of **AN-Ph** ligand which seemed to suggest that at least two species are present, based on the patterns in the A_∥_ region. Furthermore, Table 4 shows that the numbers of copper(II)-containing species simultaneously present as indicated by extensive simulations are as follows: (A) Cu-**AN-Me** (from 1:1 reaction) has three; (B) Cu-**AN-Et**-a (from 1:1 reaction) has three (C) Cu-**AN-Et**-b (from 1:2 Cu(II): ligand reaction) has three (D) Cu-**AN-Allyl** has two (E) Cu-**AN-Ph**-a (from 1:1 reaction) has two, and (F) Cu-**AN-Ph**-b (from 1:2 Cu(II): ligand reaction) has one (overwhelmingly) major species. The extracted spin-Hamiltonian parameters for the Cu(II) complexes are in agreement with the previously reported values. There is no well-defined ^14^N superhyperfine splitting. The powder CW EPR spectra of Cu-**AN-Me**, Cu-**AN-Et**-a, Cu-**AN-Et**-b, Cu-**AN-Allyl**, Cu-**AN-Ph**-a display a single broad line that is centered near the middle of the equivalent frozen glass spectrum, and this is consistent with a magnetically broadened spectrum. The powder spectrum of Cu-**AN-Ph**-a is resolved into two *g*-value components, with the more intense *g*_⊥_ at lower field than *g*_∥_, which might imply a reversal of *g*-values for the isolated Cu sites, which would be consistent with a (3d_*z*^2^_)^1^ ground state configuration being trapped in the solid lattice, which then relaxes to the more common (3d_*x*^2^__–y^2^_)^1^ configuration on dissolution.

**Table 4:** Spin-Hamiltonian Parameters Used for the Simulations of the EPR Spectra of the Cu(II) Species Formed in Samples A–F^a^

samples/metal: ligand reactants ratio	*g*-matrix^b^/population^c^	*A*-matrix in MHz^b^^,^^d^	width in mT^e^	*H*-strain in MHz^e^
(A) Cu-**AN-Me1:1**	[2.036 2.085 2.204]/0.5	|[8 37 496]|	[0.72 2.94]	[6 1 160]
	[2.073 2.087 2.403]/0.3	|[10 23 388]|	[1.14 1.08]	[29 9 45]
	[2.047 2.076 2.345]/0.6	|[10 23 408]|	[1.11 1.41]	[29 9 45]
(B) Cu-**AN-Et**-a **1:1**	[2.00 2.074 2.204]/0.5	|[8 37 496]|	[0.67 2.51]	[8 1 175]
	[2.079 2.080 2.403]/0.3	|[10 23 388]|	[1.71 0.94]	[28 10 46]
	[2.039 2.078 2.345]/0.6	|[10 23 408]|	[0.65 1.11]	[29 9 45]
(C) Cu-**AN-Et**-b **1:2**	[2.022 2.098 2.204]/0.5	|[8 37 496]|	[0.73 2.64]	[6 1 160]
	[2.074 2.087 2.403]/0.3	|[10 23 388]|	[1.61 0.85]	[29 9 45]
	[2.047 2.079 2.345]/0.6	|[10 23 408]|	[1.14 1.41]	[29 9 45]
(D) Cu-**AN-Allyl1:1**	[2.032 2.071 2.345]/0.2	|[51 21 409]|	[0.88 1.8]	[240 11 95]
	[2.075 2.090 2.400]/0.1	|[10 30 398]|	[0.93 1.84]	[30 30 27]
(E) Cu-**AN-Ph**-a **1:1**	[2.038 2.071 2.345]/0.3	|[51 21 409]|	[0.88 1.8]	[240 25 95]
	[2.075 2.090 2.400]/0.1	|[10 30 398]|	[0.93 1.84]	[30 30 27]
(F) Cu-**AN-Ph**-b **1:2**	[2.020 2.106 2.194]/0.3	|[80 40 409]|	[2.0 1.74]	[0 0 160]

a Denoted Cu-**AN-Me**, Cu-**AN-Et**-a, Cu-**AN-Et**-b, Cu-**AN-Allyl**, Cu-**AN-Ph**-a, and Cu-**AN-Ph**-b.^35^

b Accurate determination of the *g*_*x*_, *g*_*y*_, |*A*_*x*_|, and |*A*_*y*_| values was not possible owing to the second-order nature of the perpendicular region, although it was noted that satisfactory simulation could only be achieved with the particular set of values reported in the simulation. Furthermore, it was noted that the superhyperfine splitting due to ^14^N/^1^H-nuclei along the *g*_*x*_, *g*_*y*_, regions was poorly resolved/not clearly visible to the naked eye; however, given the ambiguity in the number of ^14^N/^1^H nuclei coupled to the electron spin, these were not included in the simulations for selective cases to remove the overparameterization. Simulations, which included the ^63,65^Cu-hyperfine matrix is given in the table above.

c For a selected experimental spectrum, the simulation involves inclusion of two/three EPR-active species, whose population is provided next to the *g*-matrix values.

d The sign of the hyperfine coupling is not determined, so absolute values are given.

e The line shape of the spectra was reproduced by considering an isotropic Voigtian line shape and an anisotropic broadening(H-Strain) respectively.

These observations are consistent with the ESI^+^ mass spectrometry results, which indicated that in all cases, fragments consistent with [LCu(OAc) + H^+^] and [LCu(DMSO)]^+^ (where L^–^ corresponds to the deprotonated ligand, i.e., **AN-Me**, **AN-Et**, or **AN-Ph**) can be identified additionally to the [CuL_2_]^+^. The DFT calculations for this simple system indicated that formation of such 1:1 Cu: L^–^ species is plausible, additionally to the expected [CuL_2_] species, which dominates the ESI^+^ mass spectra for all investigated Cu(II) compounds irrespective of the reactants ratios used. We already suggested in earlier studies that these versatile tridentate ONS ligands could adopt *mer*-geometry in the 1:2 Cu: ligand complexes of type ML_2_; however, if a distorted octahedral geometry is adopted in the solid state, the arrangement of the exocyclic substituents could also give rise to several geometric isomers additionally to the presence of optical isomerism, which has been proposed for similar molecules, and supported by DFT calculations.^15b^

The crystallography of Zn(II) complexes of the type ZnL_2_ (with R = H, Et, and backbones AN, PH, and PY) indicated the possibility of tetrahedral (N/S/N/S), as well as highly distorted trigonal pyramidal and octahedral arrangements of the ligands around the metal center (O/N/S/O/N/S for R = Et), as shown above, and in previous reports.^15^ Furthermore, formation of dimeric copper(II) complexes derived from the related monothiosemicarbazone anchored on 2-formylpyridine, HFoPyTSC seems ubiquitous whereby tridentate NNS and the fourth basal positions are occupied by acetate oxygen that are strongly coordinated (Cu–O bonds of ca. 1.95 Å). Related structures with centrosymmetric dimers with more weakly bound, axial placed acetate dimers are also possible (and with distances of Cu–O 2.42 Å) more closely resembling monomeric species which are weakly associated in the solid state.^36^

We also obtained the temperature and field dependent magnetization measurements for the samples A–F immobilized in eicosane (Figure S57, Supporting Information), all of which behave as paramagnets, as expected. Samples E, Cu-**AN-Ph**-a, and F, Cu-**AN-Ph**-b, showed nearly horizontal lines, however one would expect better correspondence than observed hereby if each of the samples investigated were to be considered as simple paramagnets, and the plots for Cu-**AN-Et**-a and especially Cu-**AN-Me** seem to be indicative of strong antiferromagnets.

The powder CW EPR spectra of Cu-**AN-Me**, Cu-**AN-Et**-a, Cu-**AN-Et**-b, Cu-**AN-Allyl**, Cu-**AN-Ph**-a, and Cu-**AN-Ph**-b all displayed the characteristic single broad line that is centered near the middle of the equivalent frozen glass spectrum, consistent with a magnetically broadened spectrum. Deconvolution of CW EPR spectrum of Cu(**AN-Me**) (i.e., emerging from the 1:2 reaction of Cu(OAc)_2_ with the HL-type ligand **AN-Me**) indicate the presence of 3 different copper(II) environments in this sample. If the structures we propose all show a strong component from species exhibiting distorted octahedral environments for the Cu(II) in all these samples, whether emerging from 1:1 reactions or 1:2, there must either be some very strong intermolecular interaction, or maybe the compounds are coupled ligand radicals in the solid state. For the low T magnetization, for a simple *s* = 1/2 paramagnet the value of molar magnetization expected when the curve plateaus at high field is *g* × *S*, which would be ca. 1.05 μ_B_ (assuming *g*_av_ = 2.1). Magnetisation data (Supporting Information, Figure S58) show all four compounds tend to reach saturation at high field. Since the EPR measurements also indicated a number of species present, possibly in equilibrium, the correction for diamagnetism was not deemed feasible: the decrease in magnetic moment with temperature might imply that the solid state structures resemble the supramolecular aggregation analogous to that already seen in Zn(II) complexes of type ML_2_, in that they stack extensively in the solid state and as such these Cu(II) samples would display overall antiferromagnetic interactions.

From all analytical and spectroscopy data, taken together, we speculate that the possibility of Cu(II) dimers, linked by one or even two acetate ligands in a bridging mode, similarly to the case of the literature-reported, related monothiosemicarbazone anchored on 2-formylpyridine, HFoPyTSC seems plausible, e.g., whereby tridentate NNS and the fourth basal positions are occupied by acetate oxygen that are strongly coordinated could not be discounted. However, the frozen solution EPR spectra, where it could be resolved, are all consistent with the occurrence of monometallic species being present, rather than dimers. If something like a paddlewheel dimer structure could be found then there would be seven hyperfine lines, but there are only four observed hereby, and the simulation account for all the features in the spectra. The powder EPR spectra cannot determine the exact nature of these species, although the breadth suggests there is a copper component, and on dissolution a typical Cu pattern is present: we eliminated the possibility of production of organic radical species under the mild conditions in which the reactions were conducted. As stated above the presence of a minor (inseparable) component Cu: L 1:1 additionally to the Cu: L_2_ dominant component cannot be discounted, and the extensive mass spectrometry investigations carried out (ESI) pinpoints to a range of species being feasibly present in solution, possibly in equilibrium as shown in Scheme 5, and so do the frozen solution EPR spectra. Furthermore acetate-bridged dimers with the general formula [(TSC)Cu(OAc)_2_Cu(TSC)] have been reportedly isolated for other thiosemicarbazone complexes of Cu(II), however we did not see evidence for such dimers in mass spectrometry of the species analyzed hereby. The EPR determinations in such dimeric compounds have been scarce and a direct comparison of this work, with previously investigated TSCs has not thus far been possible.^36^

### Radiochemistry Assays for the ^64^Cu Incorporation under Mild Conditions

**Figure 14:**
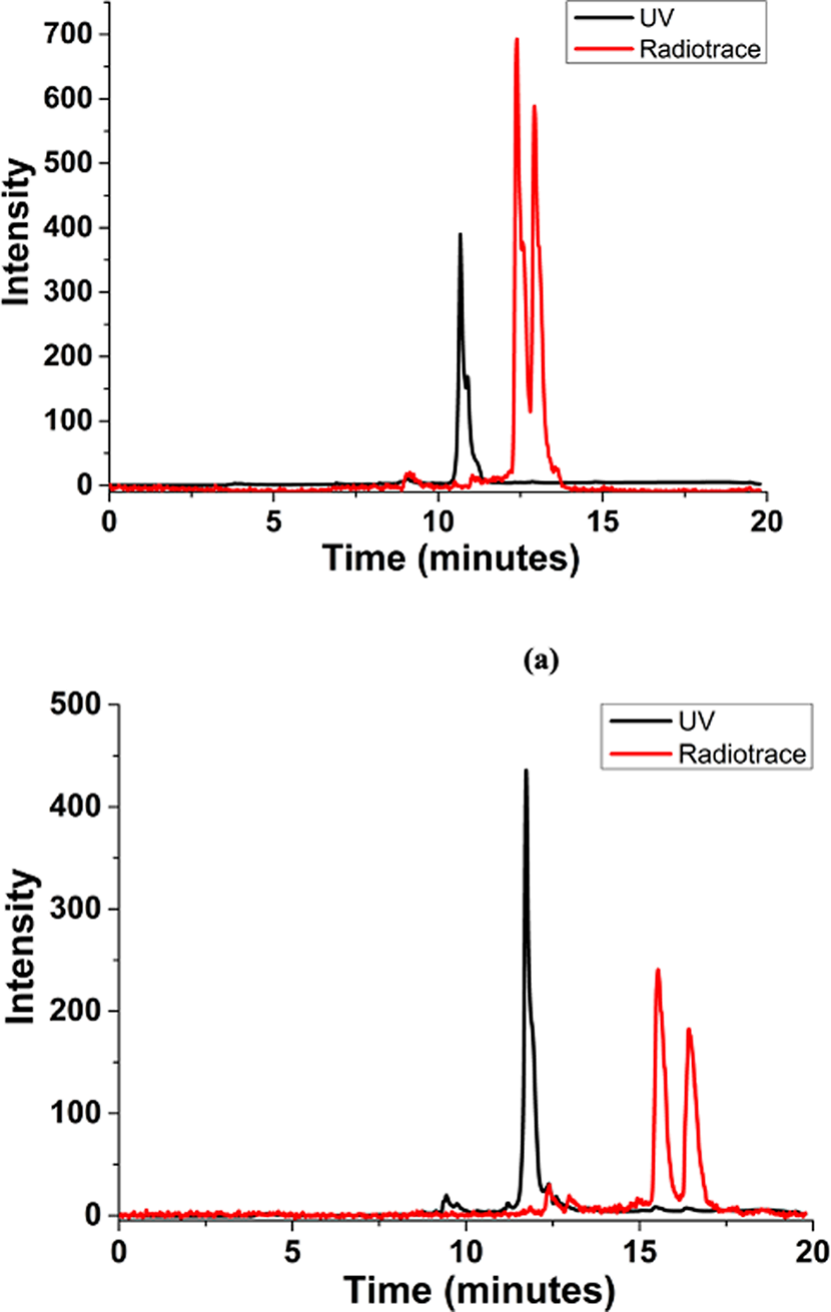
HPLC traces obtained for the optimized incorporation of ^64^Cu into **AN-Me** and **AN-Ph** ligands using ^64^Cu(OAc) under mild conditions. Normalized radioHPLC traces are overlaid with the corresponding UV-detected trace (280 nm, intensity in a.u.). Further details and additional traces are given in Supporting Information.

Metalation reactions under kinetic control were carried out using ^64^Cu(OAc)_2_, as described in the Experimental Section and in Supporting Information. Overall room temperature radiolabeling carried out at pH 5.5 generally proceeded with ca. 50% incorporation yield, whereas moderate heating for ca. 30–90 min in a range of solvents (MeOH, DMSO, on a standard heating block) led to near-quantitative ^64^Cu radiochemical incorporation (Figures 14 and S59–S69, Supporting Information). The UV detection HPLC traces of the corresponding “cold” Cu(II) complexes were difficult to assign as the degradation of the complex to free ligand occurs in the presence of TFA, and precipitation also occurs at the concentrations needed to record these HPLCs. The radioHPLCs of the ^64^Cu complexes indicate consistent behavior at the formation of copper-64 species in solution in all compounds studied. Furthermore, an increase in ^64^Cu activity used at the start of the radiolabeling experiments (from 10 mBq to 100 MBq activity in starting materials samples for the radioreaction, see Supporting Information) showed that it is possible to resolve the ^64^Cu species present, and up to three distinguishable peaks occur within 90 min experiment time under conventional heating (i.e., for the optimizations performed at the ^64^Cu labeling of **AN-Et** and **AN-Ph**).

The radioHPLC traces obtained for the reactions at room temperature pointed out the presence of two different ^64^Cu(II) species in solution, similar with the case of the analogous ^68^Ga chemistry, reported by us earlier.^15b^ Analogous to the gallium radiochemistry assays carried out under similar conditions, we also observed hereby the formation of two main ^64^Cu-based species by radio-HPLC, that cannot be separated. Reactions carried out overnight seem to lead to one major species by radioHPLC however broadening of this peak is also observed. This unusual feature is currently assigned to differences regarding the synthesis protocol under thermodynamic *vs* kinetic control, or decomposition of the CuL_2_ species to a Cu(II)LX species under radiosynthetic conditions where excess of NaOAc and other ligands may also be present in aqueous environment (X = OAc^–^, Cl^–^, or OH^–^ and L = monoanionic mono(thiosemicarbazide) ligand). Through optimized radiolabeling experiments sought to shed light into the nature of these compounds, which we assigned either to isomerism in the octahedral *vs* tetrahedral compounds, optical isomerism in the isomer with octahedral geometry and/or the simultaneous formation of a 1:1 M:L species, possibly in exchange in diluted solutions with the ML_2_-type compounds. Under kinetic control formation of species of type [CuL(DMSO]^+^ and [CuL(OAc)] could not be ruled out in the presence of competing ligands, such as (OAc)^−^ and coordinating solvents (DMSO).

Although radiochemical yields were moderate for the ligands with extended backbones, such as PH and AA (which we assigned to steric hindering and extensive aromatic stacking of the free ligands involved), the radioincorporation yield (estimated by integrating radioHPLC) was well above >90% for the AN derivatives. By similarity with what was reported for a ^61^Cu radiolabeling of a PH-backbone TSC (monitored by iTLC^41^) and our own observations from the ^68^Ga radiolabeling assays reported earlier,^15b^ we propose that the generally consistent occurrence of two different ^64^Cu species in reactions carried out at the room temperature may also be assignable to the presence of optical isomers for ^64^CuL_2_ additionally to the presence of ^64^CuLX species, that these species occur simultaneously under kinetic control, and they are generally detectable at ca. 1 min difference by radioHPLC in fresh solutions, yet not fully separable at more than two half-lives for the radio-reaction. Collection and reinjection of samples consistently led to formation of these “twin” peaks, and employment of high radioactivity starting material ^64^Cu, and slightly harsher conditions (90 min with heating in DMSO) led to observation that 3 different peaks occur, which can be distinguished within 1–2 min r.t. in reverse phase HPLC.

The addition of a base (e.g., NH_4_OH or LiOH) to deprotonate the ligand prior to ^64^Cu radiolabeling did not appear to have a significant effect in the radiolabeling yield or number of species detected by radioHPLC. Validating the nature of the species emerging from the ^64^Cu radiolabeling of monothiosemicarbazones of this family of compounds (using ^64^Cu(OAc)_2_ as the precursor of choice) proved challenging, yet in many respects analogous to the behavior observed for the analogous ^68^Ga radiochemistry. The occurrence of the “twin” peaks features are consistent with our observations from ^68^Ga incorporation in TSCs,^15b^ and we also assign this either to the simultaneous 1:1 and 1:2 Cu:Ligand association, or to isomerism in Cu(L)_2_-type complexes.

As indicated above, the analogous Cu(II) coordination chemistry, whereby reactions between the ligands investigated (**AN-H**, **AN-Me**, **AN-Et**, **AN-Allyl**, and **AN-Ph**) and Cu(OAc)_2_ were carried out under thermodynamic control (in ratio of ligands to metal of either 1:1 or 2:1) did not appear to proceed efficiently. We suggested that these resulted in complex mixtures of species that undergo equilibrium reactions in solution and seem to revert to the free ligand under the HPLC conditions in the presence TFA. Thus, the “cold” standards for analytical chemistry comparisons were unavailable for ^64^Cu(II) radiochemistry. In the case of the Zn(II) complexation reactions, these reactions invariably lead to the preferential formation of ML_2_-type derivatives. The HPLCs of the “cold” Cu(II) compounds isolated and purified from thermodynamically controlled reactions seem to indicate loss of ligand in the presence of TFA which was used under the standard HPLC conditions successfully applied for their Zn(II) analogues. Further details and corresponding data is given in Supporting Information.

We suggest that in each case the two broad signals correspond to at least two distinct complexes of copper(II) in different coordination environments and which we hypothesize to feature an earlier eluting time, presumably due to an MLX_2_ type compound together with the ML_2_ complex, and which equilibrate with time supporting our postulated structures from Scheme 5 and Figure 12. These findings are also consistent with our observations from EPR. All compounds radiolabeled rapidly with ^64^Cu(OAc)_2_ at room temperature or with moderate heating, although some challenges remain to be addressed, in line with our earlier observations from ^68^Ga chemistry: dual or triple peaks with rather close retention times were identified by radioHPLC for all these copper derivatives. These may be assignable to isomerism in ML_2_ species, or monosubstituted MLX/ML(DMSO)^+^ type thiosemicarbazones: the precise identity of all the species present in solution and their behavior at varying pH remain to be investigated in future studies in our laboratories.

## Conclusions

In summary, we developed an efficient general method for the synthesis of novel highly planar and rigid mono(thiosemicarbazone) ligands with extended aromatic backbones reliant on microwave-assisted irradiation. A new family of aromatic mono(thiosemicarbazones) was obtained by varying their exocyclic N group R substituents, including aliphatic or aromatic groups from in-house prepared or commercially available thiosemicarbazides.

The modification of the thiosemicarbazide functional groups was explored, opening new routes for the future applications of these compounds, such as true-theranostic probes for dual imaging and sensing applications. For example, the functionalization of the R group in the thiosemicarbazide opens up the possibility to further bioconjugation with targeting biomolecules. Selected ligands were used in the preparation of Zn complexes also applying a microwave method that reduced the reported procedure through conventional methods by several hours. All new compounds were fully characterized by spectroscopic techniques (^1^H and ^13^C{^1^H} NMR, IR, UV–vis, and fluorescence spectroscopies, also EPR spectroscopy in the case of the Cu(II) compounds synthesized). Their structures were demonstrated by single crystal X-ray diffraction, and a variety of conformations was highlighted for the free ligands (denoted HL) or the corresponding Zn(II) complexes of the monodeprotonated monothiosemicarbazones studied, denoted Zn(L)_2_. A selection of ligands and metal complexes was also carried forward to perform cytotoxicity assays in standard cancer cells, and for radiolabeling experiments to incorporate ^64^Cu. The biological evaluation by MTT cytotoxicity assays was pursued by *in vitro* cellular imaging experiments by laser confocal microscopy in two commonly used human cancer cell lines, HeLa (human cervical cancer cells) and PC-3 (human prostate cancer cells), and the compounds analyzed show consistently higher cytotoxic activity by comparison with cis-platin, which is in line with our previous investigations into related species.^12−15^ The potential of these mono(thiosemicarbazones) to act as synthetic scaffolds for new molecular imaging agents was explored by performing ^64^Cu radiolabeling assays analogous to those developed for [^64^Cu]Cu(ATSM) and related bis(thiosemicarbazones).^10,11,19^ Our experiments gave rise to new, longer-lived radiotracer analogues with respect to our previously investigated ^68^Ga and ^18^F labeled mono(thiosemicarbazones) in this family. We suggest that these new (^nat^Cu or ^64^Cu-labeled) copper(II) compounds, while very interesting structurally, are less kinetically stable than their Ga(III) mono- or bis(thiosemicarbazonato) complexes in aqueous, acidic solutions, especially under acidic conditions, whereas the corresponding Zn(II) compounds, which were used for optical imaging in living cells, are the most kinetically robust in this series of metal complexes. Their cytotoxicity, fluorescent emissive properties and their radiolabeling versatility with several different radioisotopes renders these mono(thiosemicarbazones) as versatile synthetic scaffolds for future theranostic agents. These findings pave the way for their more in-depth testing *in vitro* and *in vivo*: this class of compounds could be of relevance in the design and synthesis of new tracers with theranostic potential for preclinical and clinical biomedical research.

## Experimental Section

All chemicals and solvents were reagent grade and used as received (Sigma, Aldrich) unless otherwise specified. High-purity or HPLC grade solvents were obtained from Aldrich Chemical Co. (Gillingham, UK) and/or VWR (Radnor, PA, USA). Milli-Q water was obtained from a Millipore Milli-Q purification system and anhydrous solvents were obtained from a PS-400-7 Innovative technologies SPS drying system. The deuterated solvents were purchased from Aldrich and dried over 4 Å molecular sieves.

Microwave reactions were conducted in a Biotage (Uppsala, Sweden) Initiator 2.5 reactor (0–450 W depending on T) in 20 mL glass capped vials. The reaction mixture was prestirred for 30 s and then heated for the selected time. Generally, if the irradiation power is not set, it reaches its maximum (300 W from magnetron at 2.45 GHz) at the start of the reaction until the target temperature is reached, decreasing to lower values afterward.

### General Procedure A for the Synthesis of Aromatic Mono(Thiosemicarbazone) Ligands by Microwave-Assisted Heating

Aromatic diketone (1 equiv) and thiosemicarbazide (0.9–1 equiv) were charged in a microwave vial in ethanol (5–10 mL). The mixture was sonicated for 3 min to homogenize the dispersion and 3 drops of concentrated hydrochloric acid added. The vial was capped and heated under microwave irradiation at 90 °C for 10 min. The solid was filtered while hot, washed with ethanol and diethyl ether, and dried under vacuum.

### General Procedure B for the Synthesis of Zn(II) Mono(thiosemicarbazonato) Ligands by Microwave-Assisted Heating

The corresponding ligand (1 equiv, exact quantities given in each case, below) and anhydrous zinc acetate (1 equiv, exact quantities given in each case, below) were suspended in ethanol (5 mL) and the mixture was homogenized by ultrasonication. The reaction mixture was heated for 1 h at 90 °C under microwave irradiation and subsequently filtered while hot. The resulting solid was washed with ethanol, then CH_2_Cl_2_ and dried under vacuum. Further details are given below and in SI.

HPLC method A was performed in a Dionex Ultimate 3000 HPLC instrument with a UV–vis diode array detector measuring at eight wavelengths between 200 and 800 nm. MeCN/H_2_O containing 0.1% TFA were used as mobile phases at a flow rate of 1 mL/min with the following conditions: 0–1 min 5% MeCN; 1–6 min 5–95% MeCN, 6–13 min 95% MeCN, 13–16 min 95–5% MeCN; 16–20 min 5% MeCN. HPLC method B was carried out using a Dionex C18 Acclaim column (5 μm, 4.6 × 150 mm) with UV/visible detection measured at obs = 254 nm. MeCN/H_2_O containing 0.1% TFA were used as mobile phases at a flow rate of 1 mL/min with the following conditions: 0.00–0.65 min 15% MeCN; 0.65–4.10 min 15–95% MeCN; 4.10–10.70 min 95% MeCN, 10.70–12.05 min 95–15% MeCN, 12.05–15.00 min 15% MeCN.

NMR spectroscopy was performed using a Bruker (Banner Lane, UK) Advance NMR spectrometer and/or a 500 MHz Agilent automated system. Spectra were acquired at 500 MHz for ^1^H NMR, at 125 MHz for ^13^C{^1^H}NMR at 298 K, unless otherwise stated. Chemical shifts δ are reported in ppm and coupling constants (*J*) are reported in Hertz (Hz) with a possible discrepancy ≥0.2 Hz. Chemical shifts of solvent residues were identified as follows: CDCl_3_: ^1^H, δ = 7.26, ^13^C, δ = 77.0; d^6^-DMSO 1H, δ = 2.50; ^13^C, δ = 39.5; D_2_O: ^1^H, δ = 4.79). Peak multiplicities in the assignments hereby are as follows: s, singlet; d, doublet; t, triplet; q, quartet; m, multiplet; brs, broad signal.

Accurate Mass Spectrometry was carried out at the EPSRC National Mass Spectrometry Centre of Swansea University, UK, using MALDI, ESI and EI modes, also Atmospheric solids analysis probe (ASAP) using API ionization method.

The IR spectra were recorded on a PerkinElmer (Waltham, Massachusetts) Frontier FTIR spectrometer, in the range between 650 and 4000 cm^–1^ with a resolution of 4 cm^–1^. UV–visible spectra were obtained using a Lamda 650 PerkinElmer Spectrometer in DMSO and processed using UV Winlab 3 software. The orientation of the 1.00 cm quartz cuvette was the same for each experiment for consistency. Fluorescence spectra and excitation–emission maps were measured in a LS55 PerkinElmer luminescence spectrophotometer using a 1.00 cm quartz cuvette. A scan from 250–750 nm with increments of 50 nm was initially carried out to discover excitation wavelength of maximum emission (λ_ex-max_).

### Synthesis of Mono(3-Thiosemicarbazone) Acenaphthenequinone (**AN-H**)

A suspension of acenaphthenequinone (0.240 g, 1.32 mmol) and thiosemicarbazide (0.12 g, 1.32 mmol) in ethanol (10 mL) were sonicated for 3 min in a microwave vial to generate a homogeneous suspension before adding 3 drops of concentrated hydrochloric acid. The reaction mixture was heated for 10 min at 90 °C under microwave irradiation. The solid was filtered while hot, washed with ethanol, diethyl ether and dried under vacuum. The product was obtained as a yellow solid. Yield: 46% (0.156 g).
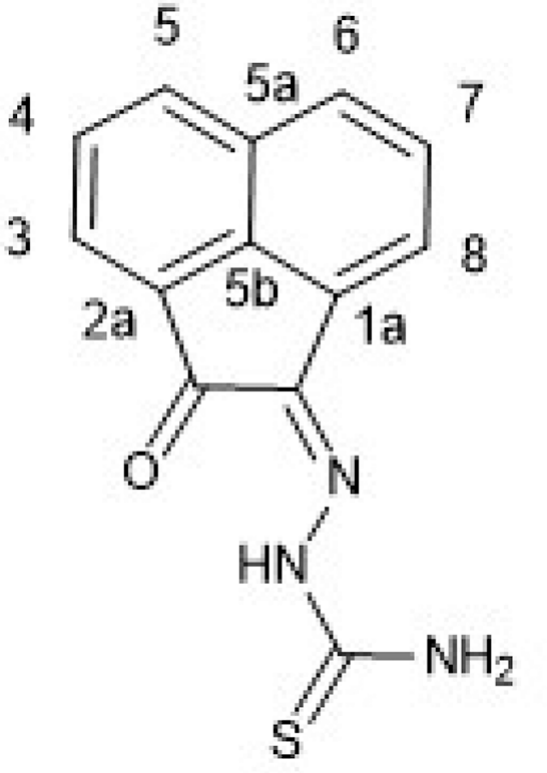


^1^H NMR (500 MHz, d^6^-DMSO, 25 °C): δ 12.52 (s, 1H, NN*H*), 9.11 (s, 1H, N*H*_*2*_), 8.82 (s, 1H, N*H*_*2*_), 8.38 (dd, *J* = 8.3, 0.7 Hz, 1H, H-5), 8.14 (dd, *J* = 8.4, 0.7 Hz, 1H, H-6), 8.10 (dd, *J* = 7.1, 0.7 Hz, 1H, H-3), 8.01 (d, *J* = 6.8 Hz, 1H, H-8), 7.88 (dd, *J* = 8.2, 7.1 Hz, 1H, H-4), 7.83 (dd, *J* = 8.4, 7.0 Hz, 1H, H-7). ^13^C{^1^H} NMR (125 MHz, d^6^-DMSO, 25 °C): δ 188.6, 178.9, 139.2, 137.4, 132.8, 130.5, 130.1, 129.9, 128.9, 128.6, 127.1, 122.4, 118.4. Mass spectrum: NSI-MS calc. for C_13_H_10_N_3_OS^+^ [M + H]^+^: 256.0539; found: 256.0542.

### Synthesis of Mono(4-Methyl-3-thiosemicarbazone) Acenaphthenequinone (**AN-Me**)

A suspension of acenaphthenequinone (0.200 g, 1.10 mmol), 4-methyl-3-thiosemicarbazide (0.108 g, 1.10 mmol) in ethanol (10 mL) were sonicated for 3 min in a microwave vial to generate a homogeneous suspension before adding 3 drops of concentrated hydrochloric acid. The reaction mixture was heated for 10 min at 90 °C under microwave irradiation. The solid was filtered while hot, washed with ethanol and diethyl ether and dried under vacuum. The product was obtained as a yellow solid. Yield: 70% (0.215 g).
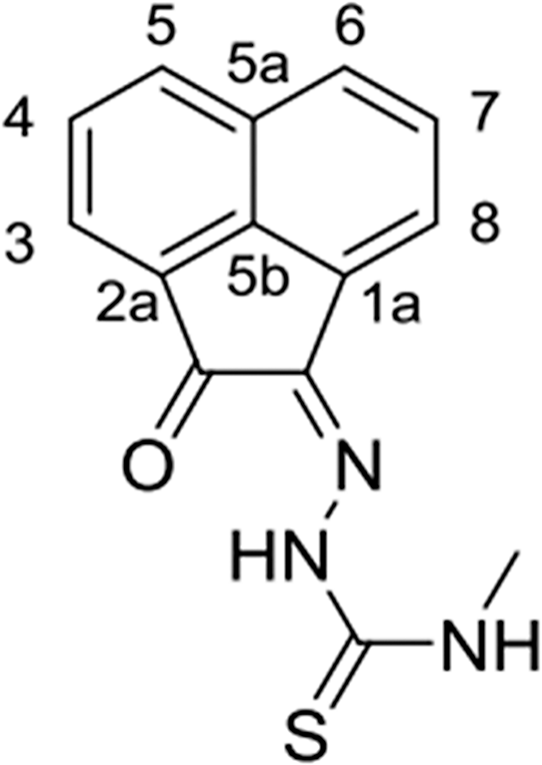


^1^H NMR (500 MHz, d^6^-DMSO, 25 °C): δ 12.64 (s, 1H, NN*H*), 9.38 (q, *J* = 4.5 Hz, 1H, N*H*CH_3_), 8.37 (d, *J* = 8.1 Hz, 1H, H-5), 8.13 (d, *J* = 8.3 Hz, 1H, H-6), 8.09 (d, *J* = 7.0 Hz, 1H, H-3), 7.97 (d, *J* = 7.0 Hz, 1H, H-8), 7.88 (dd, *J* = 8.1, 7.0 Hz, 1H, H-4), 7.84 (dd, *J* = 8.3, 7.0 Hz, 1H, H-7), 3.11 (d, *J* = 4.5 Hz, 3H, CH_3_). ^13^C{^1^H} NMR (125 MHz, d^6^-DMSO, 25 °C): δ 188.5, 177.9, 139.0, 137.1, 132.8, 130.4, 130.1, 129.9, 128.9, 128.6, 127.0, 122.4, 118.1, 31.4. Mass spectrum: ESI-MS calc. for C_14_H_11_N_3_OS [M + H]^+^: 270.0701; found 270.0700. IR (solid): ν (cm^–1^) 3219, 1689, 1540, 1475, 1055, 1027. HPLC (Method A): Rt (min) 9.53.

### Synthesis of Mono(4-(*N*-(2-(2-(2-Aminoethoxy)ethoxy)ethyl))-3-thiosemicarbazone) Acenaphthenequinone

Acenaphthenequinone (0.014 g, 0.071 mmol) and 4-(*N*-(2-(2-(2-aminoethoxy)ethoxy)ethyl))-3-thiosemicarbazide (**9**) (0.023 g, 0.071 mmol) were suspended in ethanol and homogenized by sonication for 3 min. Concentrated HCl (3 drops) was added and the reaction mixture heated to 90 °C for 10 min. The solvent was removed under vacuum, the residue resuspended in CH_2_Cl_2_ and passed through a silica plug. The product was eluted with CH_2_Cl_2_/MeOH (9:1). The solvent was removed under vacuum and the product obtained as a yellow solid. Yield: 37% (0.010 g).
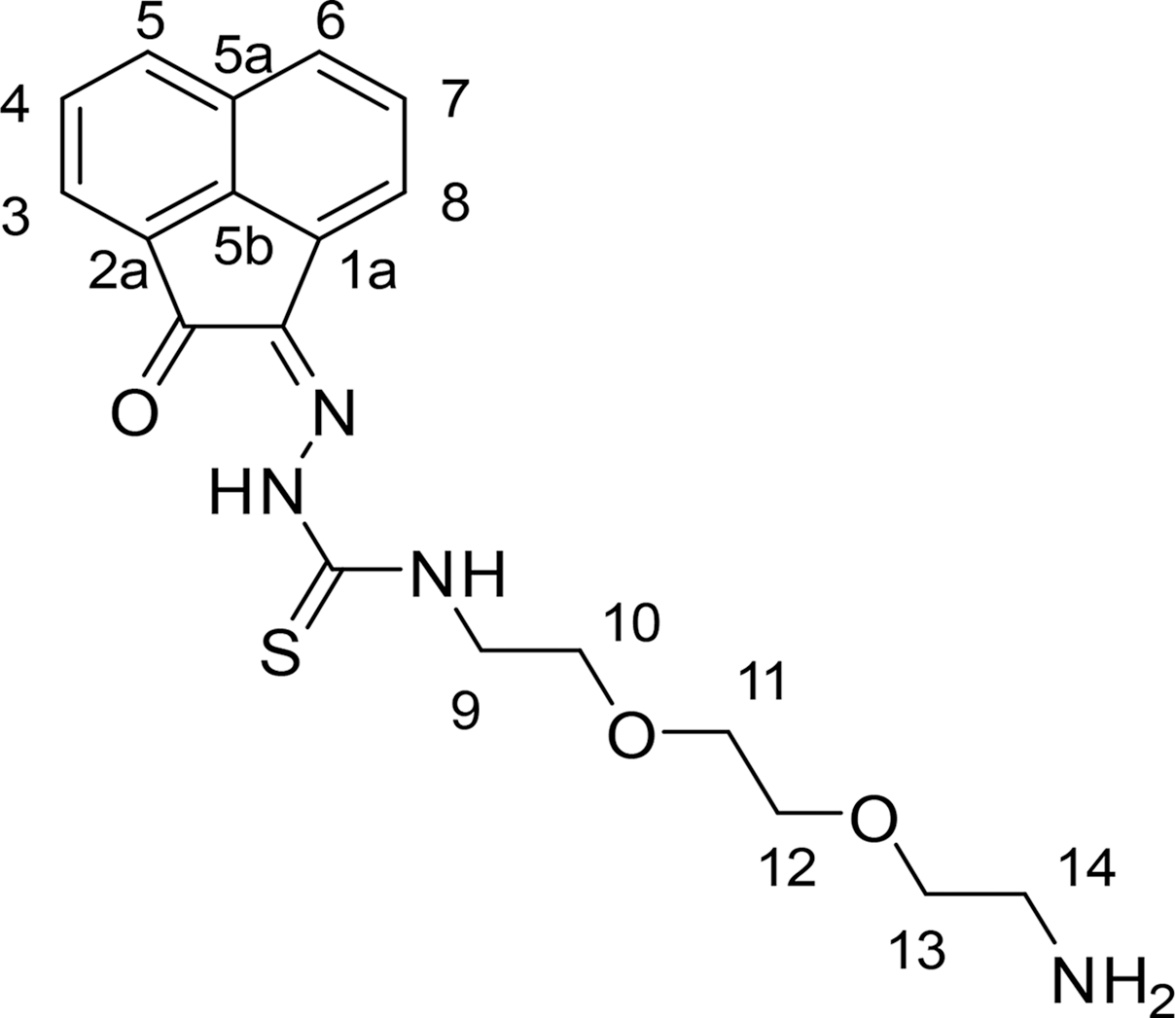


^1^H NMR (500 MHz, d^6^-DMSO, 25 °C): δ 12.66 (s, 1H, NN*H*), 9.42 (t, *J* = 5.8 Hz, 1H, N*H*CH_2_), 8.39 (d, *J* = 8.1 Hz, 1H, H-5), 8.15 (d, *J* = 8.3 Hz, 1H, H-6), 8.10 (d, *J* = 7.0 Hz, 1H, H-3), 8.05 (s, 2H, N*H*_2_), 8.04 (d, *J* = 6.9 Hz, 2H, H-8), 7.93–7.86 (m, 1H, H-4), 7.85 (dd, *J* = 8.3, 7.0 Hz, 1H, H-7), 3.83 (q, *J* = 6.0 Hz, 2H, H-9), 3.70 (t, *J* = 6.1 Hz, 2H, H-10), 3.66–3.58 (m, 6H, H-11, H-12, H-13), 2.94 (q, *J* = 5.5 Hz, 2H, H-14). ^13^C{^1^H} NMR (125 MHz, d^6^-DMSO, 25 °C): δ 188.5, 177.5, 139.2, 137.5, 132.8, 130.4, 130.0, 129.9, 128.9, 128.6, 127.1, 122.5, 118.4, 69.7, 69.5, 68.0, 66.6, 43.8, 38.5. Mass spectrum: ESI-MS calc. for C_19_H_23_N_4_O_3_S [M + H]^+^: 387.1491; found: 387.1561. IR (solid): ν (cm^–1^) 3300, 1659, 1496, 1388, 1097, 1066. HPLC (Method A): Rt (min) 11.84.

### Synthesis of Mono(4-Boc-diethylamine-3-thiosemicarbazone) Acenaphthenequinone (**AN-enBoc** or **AN-11**)

A microwave tube was filled with acenaphthenequinone (0.500 g, 2.74 mmol), 4-Boc-diethylamine thiosemicarbazide (0.640 g, 2.74 mmol), and 15 mL of acetic acid. The mixture was reacted at 90 °C in the microwave for 20 min. The slurry was then allowed to cool, filtered, and washed with diethyl ether. The precipitate was collected to afford 0.956 g of the desired compound in a yellow color with 88% yield.

^1^H NMR (300 MHz, d^6^-DMSO, 25 °C): δ 12.62 (s, 1H), 9.45 (t, 1H, *J* = 5.3 Hz), 8.38 (d, 1H, *J* = 8.2 Hz), 8.13 (overlapping d, 1H), 8.11 (overlapping d, 1H), 8.01 (d, 1H, *J* = 7.1 Hz), 7.86 (overlapping t, 2H), 7.11 (t, 1H, *J* = 5.5 Hz), 3.66 (q, 2H, *J* = 6.2 Hz), 3.66 (q, 2H, *J* = 5.8 Hz), 1.39 (s, 9H). ^13^C{^1^H} NMR (125 MHz, d^6^-DMSO, 25 °C): δ 188.6, 177.6, 156.2, 139.2, 137.3, 132.9, 130.5, 130.0, 129.9, 128.9, 128.7, 127.2, 122.6, 118.3, 78.0, 78.0, 28.2. Mass Spectrometry: ASAP, calc. for C_20_H_22_N_4_O_3_S [M + H]^+^: 399.1485; found 399.1483. IR (solid): ν (cm^–1^) 3383, 3326, 2946, 1669, 1509, 1482, 1246, 1067, 1026.

### Synthesis of Mono(3-Thiosemicarbazone) Aceanthrenequinone (**AA-H**)

**AA-H** was prepared following the general procedure A. Aceanthrenequinone (0.076 g, 1.32 mmol), thiosemicarbazide (0.030 g, 1.32 mmol) and conc. HCl (3 drops) in ethanol (10 mL) were heated for 10 min at 90 °C under microwave irradiation. The product was obtained as an orange solid. Yield: 84% (0.084 g).
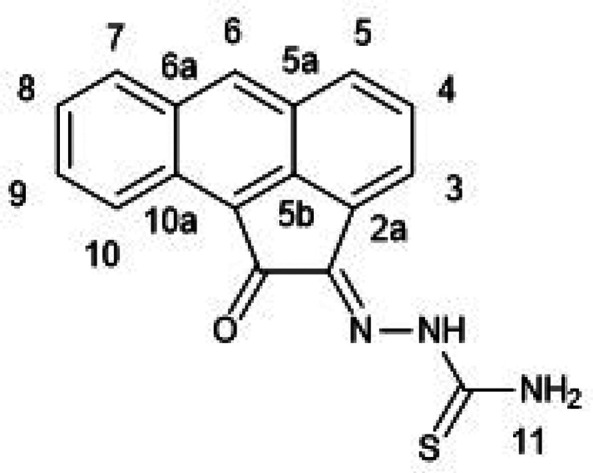


^1^H NMR (500 MHz, d^6^-DMSO, 25 °C): δ 12.74 (s, 1H, NNH), 9.13 (s, 1H, N*H*_*2*_), 9.06 (s, 1H, N*H*_*2*_), 8.90 (dd, *J* = 8.5, 1.1 Hz, 1H, H-6), 8.83 (s, 1H, H-10), 8.37–8.32 (m, 1H, H-7), 8.20 (d, *J* = 8.5 Hz, 1H, H-5), 7.97 (d, *J* = 6.6 Hz, 1H, H-3), 7.90 (ddd, *J* = 8.3, 6.6, 1.2 Hz, 1H, H-9), 7.80–7.70 (m, 2H, H-4, H-8). ^13^C{^1^H} NMR (125 MHz, d^6^-DMSO, 25 °C): δ 188.4, 179.0, 141.0, 137.4, 134.7, 132.7, 130.5, 130.1, 129.4, 128.0, 127.5, 127.3, 127.2, 126.9, 123.5, 123.3, 118.4. Mass spectrum: ESI^+^ calc. for C_17_H_12_N_3_OS [M + H]^+^: 306.0696; found: 306.0698.

### Synthesis of Mono(4-Methyl-3-thiosemicarbazone) Aceanthrenequinone (**AA-Me**)

**AA-Me** was prepared following the general procedure A. Aceanthrenequinone (0.208 g, 0.86 mmol), 4-methyl-3-thiosemicarbazide (0.086 g, 0.82 mmol), and conc. HCl (3 drops) in ethanol (10 mL) were heated for 10 min at 90 °C under microwave irradiation. The product was obtained as a red-brown solid. Yield: 79% (0.207 g).
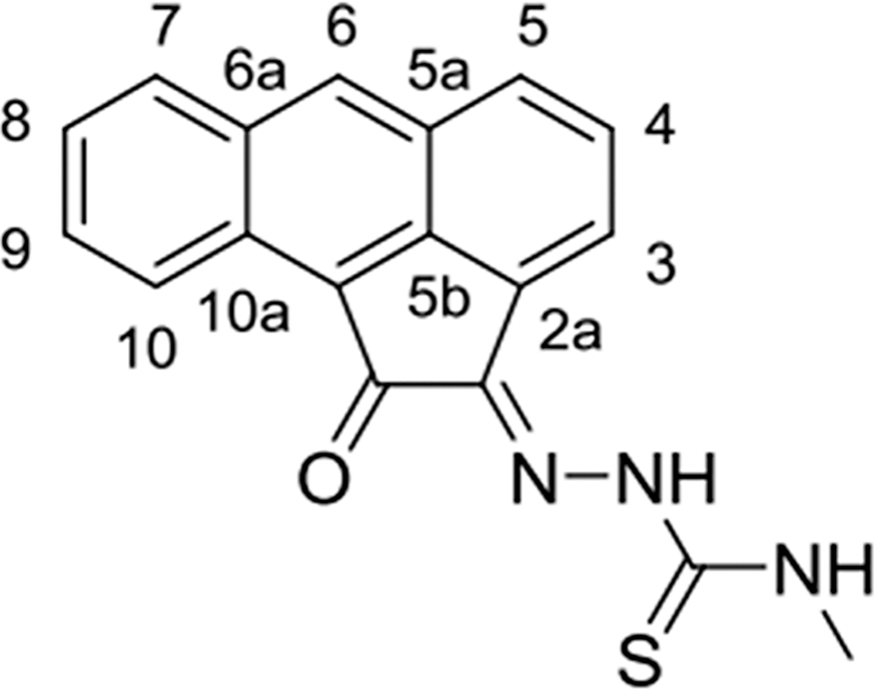


^1^H NMR (500 MHz, d^6^-DMSO, 25 °C): δ 12.89 (s, 1H, NN*H*), 9.41 (q, *J* = 4.0 Hz, 1H, N*H*CH_3_), 9.11 (s, 1H, H-6), 8.95 (d, *J* = 8.5 Hz, 1H, H-10), 8.38 (d, *J* = 8.4 Hz, 1H, H-7), 8.23 (d, *J* = 8.6 Hz, 1H, H-5), 7.97 (d, *J* = 6.6 Hz, 1H, H-3), 7.95–7.89 (m, 1H, H-9), 7.81 (dd, *J* = 8.6, 6.6 Hz, 1H, H-4), 7.78–7.72 (m, 1H, H-8), 3.15 (d, *J* = 4.6 Hz, 3H, C*H*_3_). ^13^C{^1^H} NMR (125 MHz, d^6^-DMSO, 25 °C): δ 188.5, 178.0, 140.9, 137.1, 134.7, 132.8, 130.5, 130.2, 129.5, 128.0, 127.5, 127.4, 127.2, 126.9, 123.6, 123.3, 118.1, 31.4. Mass spectrum: ESI-MS calc. for C_18_H_13_N_3_NaOS [M + Na]^+^: 342.0677, found: 342.0658. IR (solid): ν (cm^–1^) 3373, 3250, 1661, 1542, 1479, 1048. HPLC (Method A): Rt (min) 11.08.

### Synthesis of Mono(4-Ethyl-3-thiosemicarbazone) Aceanthrenequinone (**AA-Et**)

Compound **AA-Et** was prepared following the general procedure A. Aceanthrenequinone (0.208 g, 0.86 mmol), 4-ethyl-3-thiosemicarbazide (0.098 g, 0.82 mmol) and conc. HCl (3 drops) were heated for 10 min at 90 °C under microwave irradiation. The product was obtained as an orange solid. Yield: 89% (0.243 g).
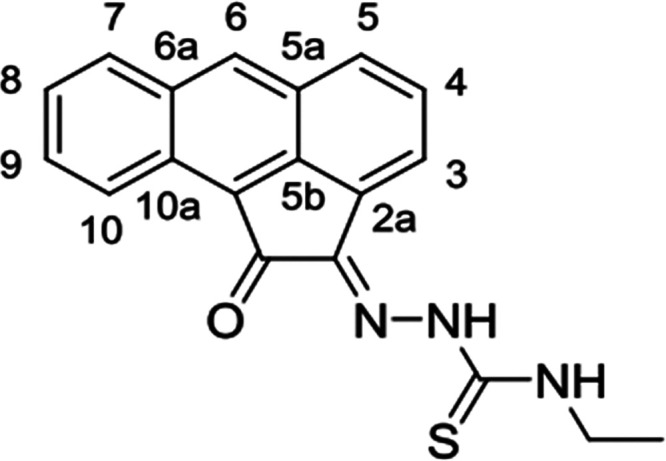


^1^H NMR (500 MHz, d^6^-DMSO, 25 °C): δ 12.81 (s, 1H, NN*H*), 9.41 (t, *J* = 5.6 Hz, 1H, N*H*CH_2_), 9.06 (s, 1H, H-6), 8.90 (d, *J* = 8.6 Hz, 1H, H-10), 8.35 (d, *J* = 8.5 Hz, 1H, H-7), 8.20 (d, *J* = 8.6 Hz, 1H, H-5), 7.96 (d, *J* = 6.7 Hz, 1H, H-3), 7.93–7.86 (m, 1H, H-9), 7.78 (dd, *J* = 8.6, 6.7 Hz, 1H, H-4), 7.76–7.70 (m, 1H, H-8), 3.80–3.63 (m, 2H, C*H*_2_), 1.25 (t, *J* = 7.1 Hz, 3H, C*H*_3_). ^13^C{^1^H} NMR (125 MHz, d^6^-DMSO, 25 °C): δ 188.4, 179.0, 141.0, 137.4, 134.7, 132.7, 130.5, 130.1, 129.4, 128.0, 127.5, 127.3, 127.2, 126.9, 123.5, 123.3, 118.4. Mass spectrum: ESI-MS calc. for C_19_H_15_N_3_NaOS [M + Na]^+^: 356.0833; found: 356.0801. IR (solid): ν (cm^–1^) 3373, 3220, 2967, 1665, 1527, 1482, 1193, 1147, 1072. HPLC (Method A): Rt (min) 11.57.

### Synthesis of Mono(4-Allyl-3-thiosemicarbazone) Aceanthrenequinone (**AA-Allyl**)

Compound **AA-Allyl** was prepared following the general procedure A. Aceanthrenequinone (0.208 g, 0.86 mmol), 4-allyl-3-thiosemicarbazide (0.107 g, 0.82 mmol), and conc. HCl (3 drops) were heated for 10 min at 90 °C under microwave irradiation. The product was obtained as an orange solid. Yield: 81% (0.229 g).
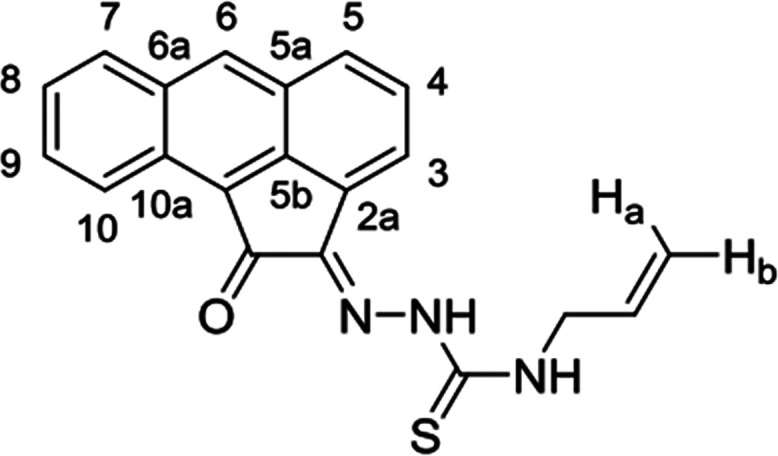


^1^H NMR (500 MHz, d^6^-DMSO, 25 °C): δ 12.89 (s, 1H, NN*H*), 9.59 (t, *J* = 5.9 Hz, 1H, N*H*CH_2_), 9.09 (s, 1H, H-6), 8.93 (d, *J* = 8.8 Hz, 1H, H-10), 8.37 (d, *J* = 8.5 Hz, 1H, H-7), 8.22 (d, *J* = 8.7 Hz, 1H, H-5), 8.00 (d, *J* = 6.4 Hz, 1H, H-3), 7.91 (ddd, *J* = 8.4, 6.7, 1.3 Hz, 1H, H-9), 7.79 (dd, *J* = 8.1, 6.7 Hz, 1H, H-4), 7.75 (ddd, *J* = 8.3, 6.7, 1.3 Hz, 1H, H-8), 6.07–5.84 (m, 1H, 1H, CH), 5.26 (d, *J*_trans_ = 17.1 Hz, 1H, Ha), 5.19 (d, *J*_cis_ = 10.2 Hz, 1H, Hb), 4.33 (brs, 2H, NHC*H*_2_). ^13^C{^1^H} NMR (125 MHz, d^6^-DMSO, 25 °C): δ 188.4, 177.6, 140.9, 137.3, 134.7, 134.0, 132.8, 130.5, 130.2, 129.4, 127.9, 127.5, 127.3, 127.2, 126.9, 123.6, 123.3, 118.3, 116.3, 46.4. Mass spectrum: ESI-MS calc. for C_20_H_15_N_3_NaOS [M + Na]^+^: 368.0833; found: 368.0824. IR (solid): ν (cm^–1^) 3360, 3217, 3066, 1664, 1526, 1485, 1190, 1149, 1075. HPLC (Method A): Rt (min) 11.59.

### Alternative Procedure for the Synthesis of Mono(4-Allyl-3-thiosemicarbazone) Aceanthrenequinone (**AA-Allyl**) by Conventional Heating

Aceanthrenequinone (0.100 g, 0.431 mmol) and 4-ally-3-thiosemicarbazone (0.068 g, 0.517 mmol) were reacted together at a ratio of 1:1.2, respectively. The compounds were added to 40 mL of ethanol and refluxed for 4 h. Once the maximum temperature had been reached of 100 °C, a few drops of glacial acetic acid were added. The isolation of the orange/red solid was obtained by evaporation of the solvent and filtration with diethyl ether. Washing with diethyl ether removed impurities. Yield = 72% (0.107 g).
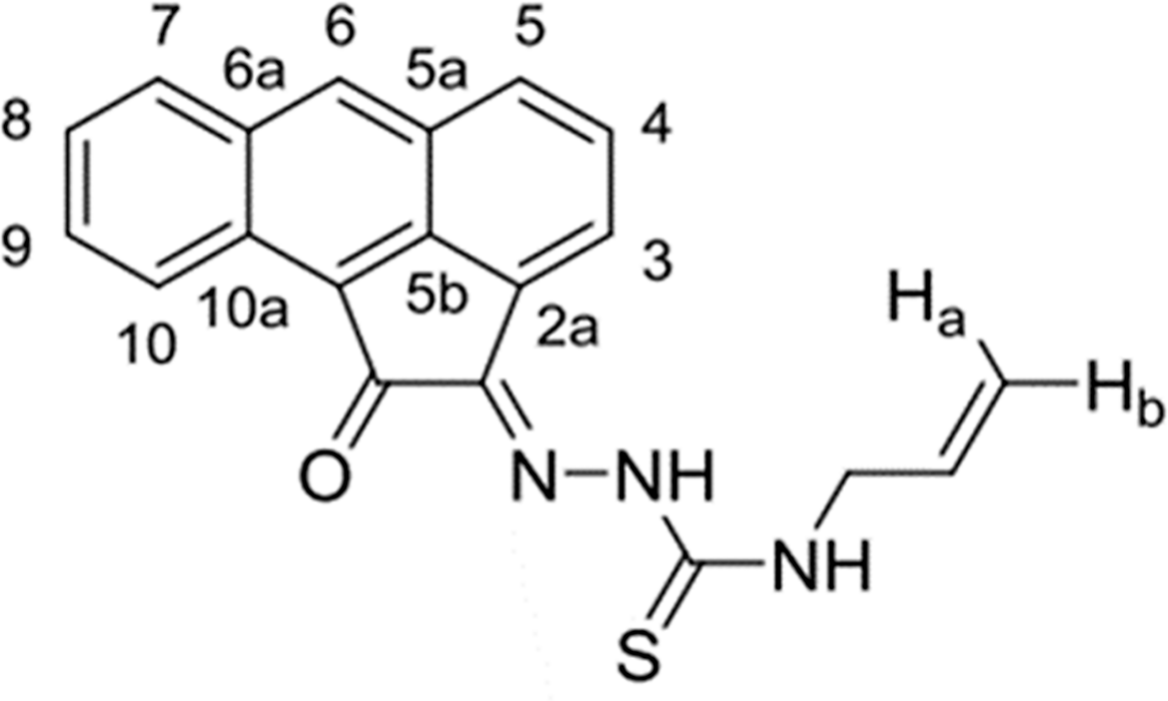


^1^H NMR (300 MHz, d^6^-DMSO, 25 °C) δ 12.97 (s, 1H, NN*H*), 9.60 (d, 1H, N*H*CH_2_), 9.2 (s, 1H, H-6), 8.5 (dd, 1H, H-10), 8.4 (dd, 1H, H-7), 8.3 (t, 1H, H-5), 7.85 (m, 2H, H-3, H-9) 7.7 (m, 2H, H-4, H-8), 6.0 (m, 1H, C*H*), 5.2 (m, 2H, Ha, Hb), 4.2 (dd, 2H, NHC*H*_2_).

### Synthesis of Mono(4-Phenyl-3-thiosemicarbazone) Aceanthrenequinone (**AA-Ph**)

**AA-Ph** was prepared following the general procedure A. Aceanthrenequinone (0.208 g, 0.86 mmol), 4-phenyl-3-thiosemicarbazide (0.137 g, 0.82 mmol), and conc. HCl (3 drops) in ethanol (10 mL) were heated for 10 min at 90 °C under microwave irradiation. The product was obtained as an orange solid. Yield: 82% (0.256 g).
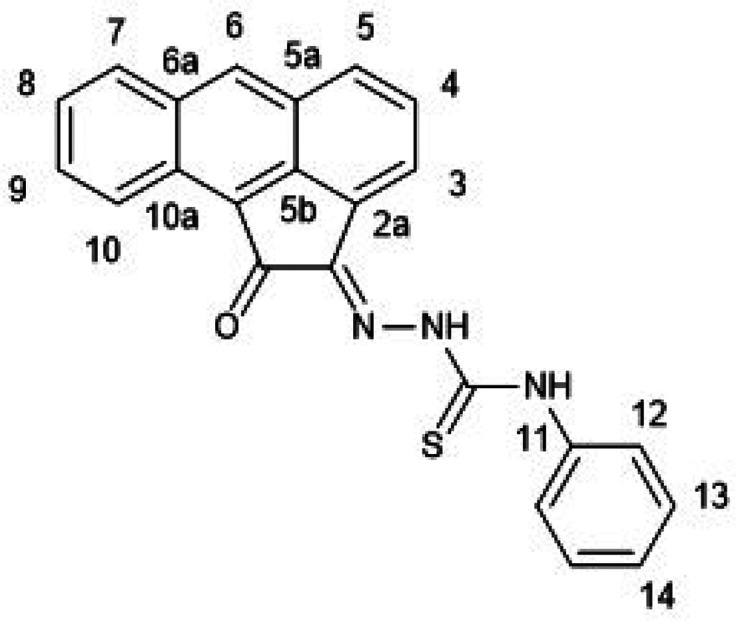


^1^H NMR (500 MHz, d^6^-DMSO, 25 °C): δ 13.05 (s, 1H, NN*H*), 10.96 (s, 1H, CSN*H*), 9.10 (s, 1H, H-6), 8.93 (d, *J* = 8.7 Hz, 1H, H-10), 8.37 (d, *J* = 8.6 Hz, 1H, H-7), 8.23 (d, *J* = 8.6 Hz, 1H, H-5), 8.10 (d, *J* = 6.7 Hz, 1H, H-3), 7.98–7.87 (m, 1H, H-9), 7.85–7.78 (m, 1H, H-4), 7.79–7.70 (m, 1H, H-8), 7.67 (d, *J* = 7.8 Hz, 2H, H-12), 7.51–7.42 (m, 2H, H-13), 7.35–7.28 (m, 1H, H-14). ^13^C{^1^H} NMR (125 MHz, CDCl_3_, 25 °C): δ 137.86, 134.40, 130.53, 129.84, 129.08, 127.81, 127.61, 127.26, 126.51, 124.63, 124.20, 118.00, 58.64, 18.60. FTIR (solid): ν (cm^–1^): 3303, 3201, 3032, 1663, 1592, 1476, 1249, 1158, 1071.

### Synthesis of Mono(4-(*N*-(2-(2-(2-Aminoethoxy)ethoxy)ethyl))-3-thiosemicarbazone) Aceanthrenequinone

Aceanthrenequinone (0.104 g, 0.86 mmol) and corresponding (0.132 g, 0.41 mmol) were suspended in ethanol (5 mL) and homogenized by sonication for 3 min. Concentrated HCl (3 drops) was added and the reaction mixture heated to 90 °C for 10 min by microwave irradiation. The solvent was removed under vacuum and the product resuspended in CH_2_Cl_2_ and passed through a silica plug. After washing with CH_2_Cl_2_, the product was eluted with CH_2_Cl_2_/MeOH (9:1). The solvent was removed under vacuum and the product obtained as an orange solid. Yield: 53% (0.094 g).
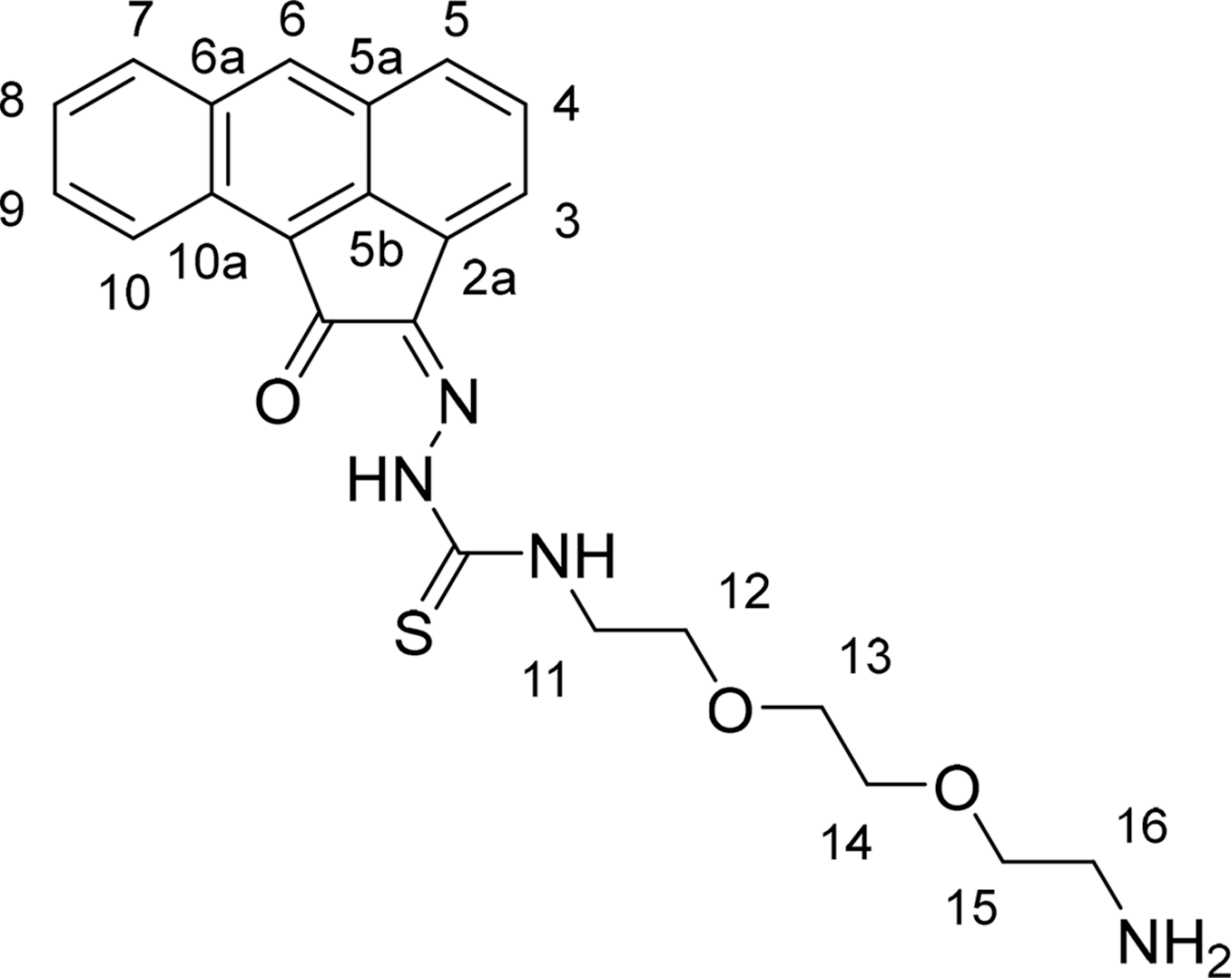


^1^H NMR (500 MHz, d^6^-DMSO, 25 °C): δ 12.86 (s, 1H, NN*H*), 9.40 (t, *J* = 5.8 Hz, 1H, N*H*CH_2_), 9.06 (s, 1H, H-6), 8.88 (dt, *J* = 8.5, 1.0 Hz, 1H, H-10), 8.35 (dd, *J* = 8.3, 1.0 Hz, 1H, H-7), 8.20 (d, *J* = 8.6 Hz, 1H, H-5), 8.00 (brs, 2H, N*H*_*2*_), 7.96 (dd, *J* = 6.7, 0.7 Hz, 1H, H-3), 7.89 (ddd, *J* = 8.4, 6.7, 1.2 Hz, 1H, H-9), 7.78 (dd, *J* = 8.6, 6.7 Hz, 1H, H-4), 7.74 (ddd, *J* = 8.1, 6.6, 1.2 Hz, 1H, H-8), 3.86 (q, *J* = 6.0 Hz, 2H, H-11), 3.73 (t, *J* = 6.0 Hz, 2H, H-12), 3.68–3.61 (m, 6H, H-13, H-14, H-15), 2.96 (q, *J* = 5.4 Hz, 2H, H-16). Mass spectrum: ESI-MS calc. for C_23_H_23_N_4_O_3_S [M + H]^+^: 435.1491; found: 435.1453. IR (solid): ν (cm^–1^): 3367, 3324, 2870, 1630, 1598, 1538, 1485, 1446. HPLC (Method A): Rt (min) 11.84.

### Synthesis of Mono(3-Thiosemicarbazone) phenanthrenequinone (**PH-H**)

**PH-H** was prepared following the general procedure A. Phenanthrenequinone (0.274 g, 1.32 mmol), thiosemicarbazide (0.120 g, 1.32 mmol) and conc. HCl (3 drops) in ethanol (10 mL) were heated for 10 min at 90 °C under microwave irradiation. The product was obtained as an orange solid. Yield: 84% (0.312 g).
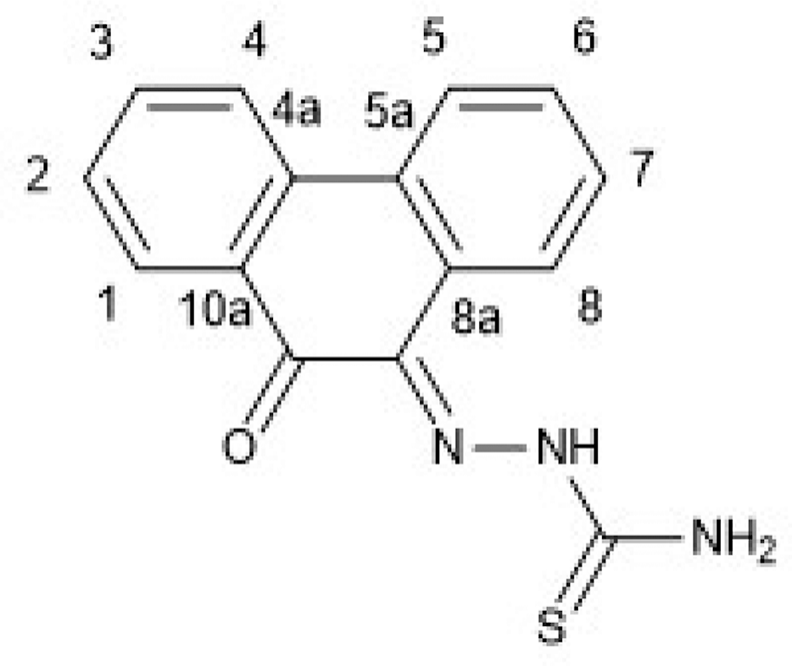


^1^H NMR (500 MHz, d^6^-DMSO, 25 °C): δ 14.41 (s, 1H, NN*H*), 9.36 (s, 1H, N*H*_*2*_), 9.08 (s, 1H, N*H*_*2*_), 8.70 (dd, *J* = 8.1, 1.4 Hz, 1H, H-8), 8.44 (d, *J* = 8.1 Hz, 1H, H-4), 8.35 (dd, *J* = 8.1, 1.2 Hz, 1H, H-5), 8.28 (dd, *J* = 7.9, 1.4 Hz, 1H, H-1), 7.86 (ddd, *J* = 8.4, 7.2, 1.5 Hz, 1H, H-3), 7.63–7.53 (m, 2H, H-2, H-6), 7.47 (ddd, *J* = 8.2, 7.1, 1.2 Hz, 1H, H-7). ^13^C{^1^H} NMR (125 MHz, d^6^-DMSO, 25 °C): δ 181.5, 179.5, 136.1, 135.4, 130.5, 130.1, 129.9, 129.5, 129.0, 128.9, 128.4, 128.3, 125.4, 123.8, 123.7. Mass spectrum: NSI-MS calc. for C_15_H_12_N_3_OS [M + H]^+^: 282.0696; found: 282.0697.

### Synthesis of Mono(4-Methyl-3-thiosemicarbazone) Phenanthrenequinone (**PH-Me**)

Compound **PH-Me** was prepared following the general procedure A. Phenanthrenequinone (0.210 g, 0.96 mmol), 4-methyl-3-thiosemicarbazide (0.096 g, 0.91 mmol) and conc. HCl (3 drops) were heated for 10 min at 90 °C under microwave irradiation. The product was obtained as a yellow solid. Yield: 92% (0.249 g).
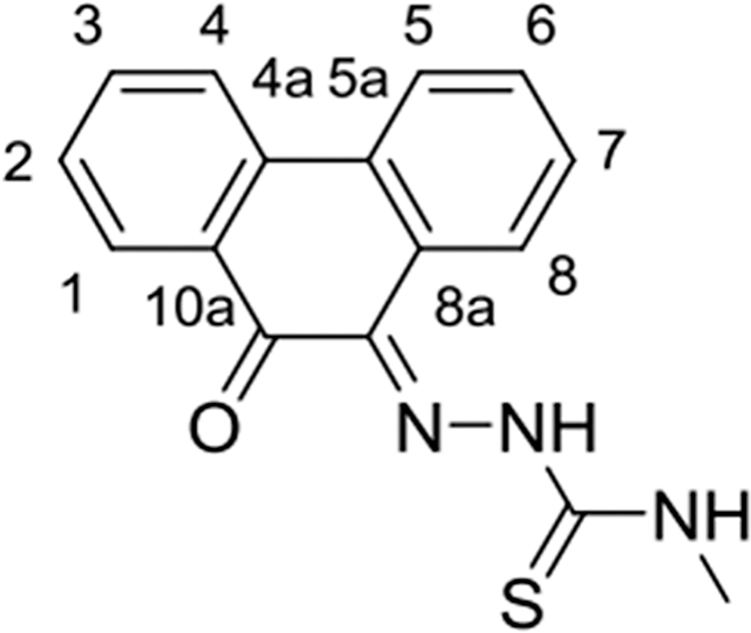


^1^H NMR (500 MHz, d^6^-DMSO, 25 °C): δ 14.59 (s, 1H, NN*H*) 9.56 (q, *J* = 4.5 Hz, 1H, N*H*CH_3_), 8.66 (dd, *J* = 8.0, 1.3 Hz, 1H, H-8), 8.43 (d, *J* = 8.0 Hz, 1H, H-4), 8.34 (dd, *J* = 7.9, 0.5 Hz, 1H, H-5), 8.26 (dd, *J* = 7.9, 1.4 Hz, 1H, H-1), 7.85 (ddd, *J* = 8.0, 7.2, 1.4 Hz, 1H, H-3), 7.60–7.57 (m, 1H, H-2), 7.57–7.54 (m, 1H, H-6), 7.49 (ddd, *J* = 8.0, 7.1, 1.2 Hz, 1H, H-7), 3.16 (d, *J* = 4.5 Hz, 3H, *CH*_*3*_). ^13^C{^1^H} NMR (125 MHz, d^6^-DMSO, 25 °C): δ 181.2, 178.1, 136.0, 135.3, 130.5, 129.8, 129.7, 129.4, 128.8, 128.8, 128.4, 128.2, 125.0, 123.8, 123.7, 31.8. Mass spectrum: ESI-MS calc. for C_16_H_13_N_3_NaOS [M + Na]^+^: 318.0677; found: 318.0718. IR (solid): ν (cm^–1^) 3324, 2976, 1638, 1595, 1552, 1448, 1040. HPLC (Method A): Rt (min) 8.83.

### Synthesis of Mono(4-ethyl-3-thiosemicarbazone) Phenanthrenequinone (**PH-Et**)

Compound **PH-Et** was prepared following the general procedure A. Phenanthrenequinone (0.210 g, 0.96 mmol), 4-ethyl-3-thiosemicarbazide (0.109 g, 0.91 mmol), and conc. HCl (3 drops) were heated for 10 min at 90 °C under microwave irradiation. The product was obtained as red solid. Yield: 76% (0.214 g).
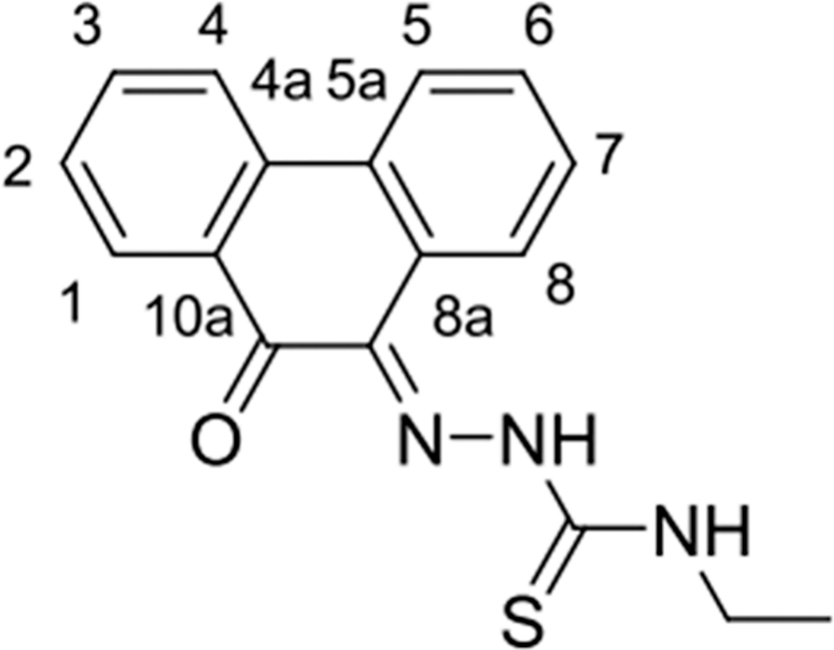


^1^H NMR (500 MHz, d^6^-DMSO, 25 °C): δ 14.55 (s, 1H, NN*H*), 9.65 (t, *J* = 5.8 Hz, 1H, N*H*CH_2_), 8.66 (dd, *J* = 8.0, 1.3 Hz, 1H, H-8), 8.44 (d, *J* = 8.1 Hz, 1H, H-4), 8.35 (d, *J* = 7.3 Hz, 1H, H-5), 8.27 (dd, *J* = 7.9, 1.4 Hz, 1H, H-1), 7.86 (ddd, *J* = 8.1, 7.3, 1.5 Hz, 1H, H-3), 7.61–7.58 (m, 1H, H-2), 7.58–7.55 (m, 1H, H-6), 7.51 (ddd, *J* = 8.0, 7.1, 1.3 Hz, 1H, H-7), 3.77–3.67 (m, 2H, C*H*_2_), 1.24 (t, *J* = 7.1 Hz, 3H, CC*H*_3_). ^13^C{^1^H} NMR (125 MHz, d^6^-DMSO, 25 °C): δ 181.2, 177.2, 136.0, 135.3, 130.5, 129.8, 129.4, 128.9, 128.8, 128.4, 128.3, 125.1, 123.8, 123.7, 39.0, 13.8. Mass spectrum: ESI-MS calc. for C_17_H_15_N_3_NaOS [M + Na]^+^: 332.0833; found: 332.0822. IR (solid): ν (cm^–1^) 3339, 2977, 1682, 1600, 1595, 1482. HPLC (Method A): Rt (min) 8.46.

### Synthesis of Mono(4-Allyl-3-thiosemicarbazone) Phenanthrenequinone (****PH-Allyl****)

Compound **PH-Allyl** was prepared following the general procedure A. Phenanthrenequinone (0.210 g, 0.96 mmol), 4-allyl-3-thiosemicarbazide (0.120 g, 0.91 mmol), and conc. HCl (3 drops) were heated for 10 min at 90 °C under microwave irradiation. The product was obtained as a yellow solid. Yield: 86% (0.253 g).
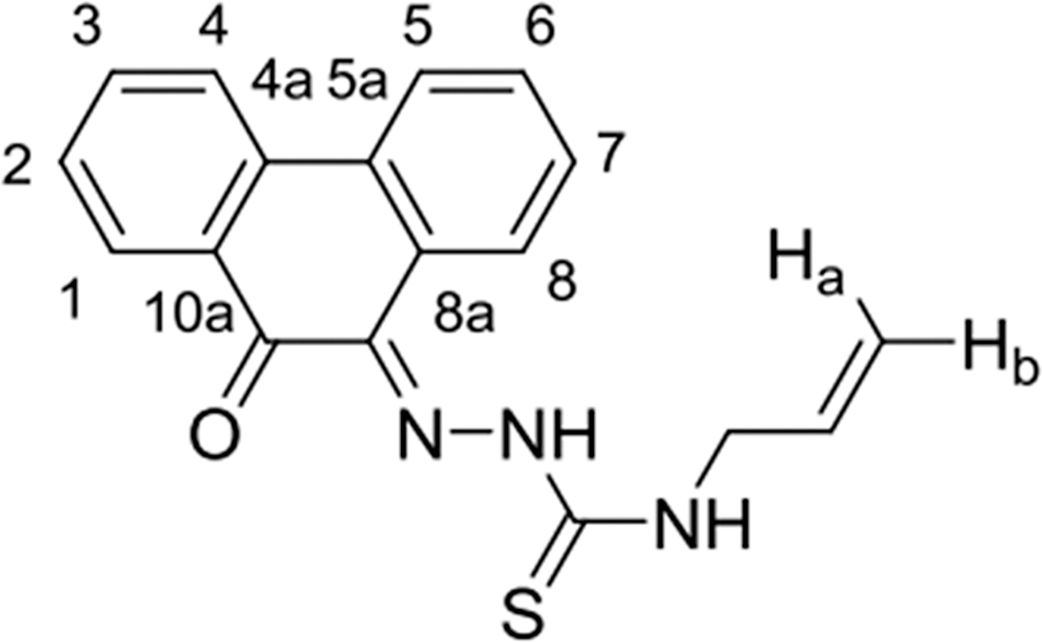


^1^H NMR (500 MHz, d^6^-DMSO, 25 °C): δ 14.59 (s, 1H, NN*H*), 9.79 (t, *J* = 5.8 Hz, 1H, N*H*CH_2_), 8.67 (dd, *J* = 8.0, 1.4 Hz, 1H, H-8), 8.42 (d, *J* = 8.1 Hz, 1H, H-4), 8.33 (d, *J* = 8.0 Hz, 1H, H-5), 8.26 (dd, *J* = 7.9, 1.3 Hz, 1H, H-1), 7.87–7.82 (m, 1H, H-3), 7.61–7.57 (m, 1H, H-2), 7.57–7.54 (m, 1H, H-6), 7.52–7.47 (m, 1H, H-7), 6.02–5.92 (m, 1H, CH_2_C*H*), 5.24 (dd, *J*_trans_ = 17.2, 1.2 Hz, 1H, H-a), 5.17 (dd, *J*_cis_ = 10.3, 1.1 Hz, 1H, H-b), 4.33 (t, J = 5.6 Hz, 2H, C*H*_2_) ^13^C{^1^H} NMR (125 MHz, d^6^-DMSO, 25 °C): δ 181.3, 177.9, 136.0, 135.3, 133.7, 130.4, 130.0, 129.8, 129.4, 128.9, 128.8, 128.4, 128.3, 125.2, 123.8, 123.7, 116.3, 46.6. Mass spectrum: ESI-MS calc. for C_18_H_15_N_3_NaOS [M + Na]^+^: 344.0833; found: 344.0815. IR (solid): ν (cm^–1^) 3311, 2977, 1645, 1599, 1588, 1449. HPLC (Method A): Rt (min) 11.02.

### Alternative Procedure for the Synthesis of Mono(4-Allyl-3-thiosemicarbazone) Phenanthrenequinone by Conventional Heating (**PH-Allyl**)

9,10-Phenanthrenequinone (0.500 g, 2.15 mmol) was suspended in acetic acid at 70 °C and heated to 120 °C. Then, 4-allyl-3-thiosemicarbazide (2.540 g, 19.4 mmol) and calcium chloride (0.720 g, 6.5 mmol) were added and the reaction mixture refluxed under nitrogen atmosphere for 4 h. More acetic acid was added (10 mL), and the red solid formed isolated by filtration and dried under vacuum. After drying, the product is obtained as an orange solid. Yield: 85% (0.588 g).
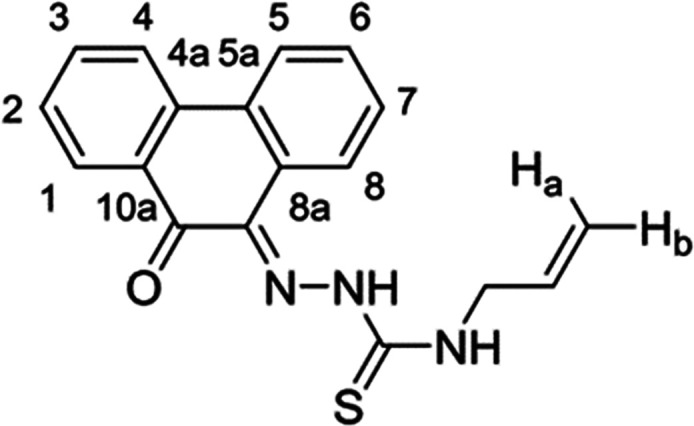


^1^H NMR (400 MHz, *d*^6^-DMSO, 25 ^*◦*^C): δ 12.92 (s, 1H, NN*H*), 9.62 (t, *J* = 5.9 Hz, 1H, N*H*CH_2_), 9.13 (s, 1H, H-8), 8.97 (d, *J* = 8.6 Hz, 1H, H-4), 8.40 (d, *J* = 8.6 Hz, 1H, H-5), 8.25 (d, *J* = 8.6 Hz, 1H, H-1), 8.03 (d, *J* = 6.8 Hz, 1H, H-3), 7.97–7.91 (m, 1H, H-2), 7.84–7.74 (m, 2H, H-6, H-7), 5.97 (ddt, *J* = 17.3, 10.6, 5.4 Hz, 1H, CH_2_C*H*), 5.25 (dd, *J*_trans_ = 17.2, 1.6 Hz, 1H, H-a), 5.18 (dd, *J*_cis_ = 10.3, 1.5 Hz, 1H, H-b), 4.32 (t, *J* = 5.6 Hz, 2H, C*H*_2_). ^13^C{^1^H} NMR (75 MHz, *d*^6^-DMSO) δ 188.48, 177.62, 140.95, 137.34, 134.73, 134.03, 132.79, 130.19, 129.46, 127.52, 127.35, 127.22, 126.94, 123.59, 123.32, 118.38, 116.30, 46.45. Mass spectrum: ESI-MS calculated for C_18_H_15_N_3_NaOS [M + Na]^+^ 344.0833, found 344.0846.

### Synthesis of Mono(4-Phenyl-3-thiosemicarbazone) Phenanthrenequinone (**PH-Ph**)

**PH-Ph** was prepared following the general procedure A. Phenanthrenequinone (0.200 g, 0.91 mmol), 4-phenyl-3-thiosemicarbazide (0.152 g, 0.91 mmol), and conc. HCl (3 drops) in ethanol (10 mL) were heated for 10 min at 90 °C under microwave irradiation. The product was obtained as an orange solid. Yield: 63% (0.204 g).
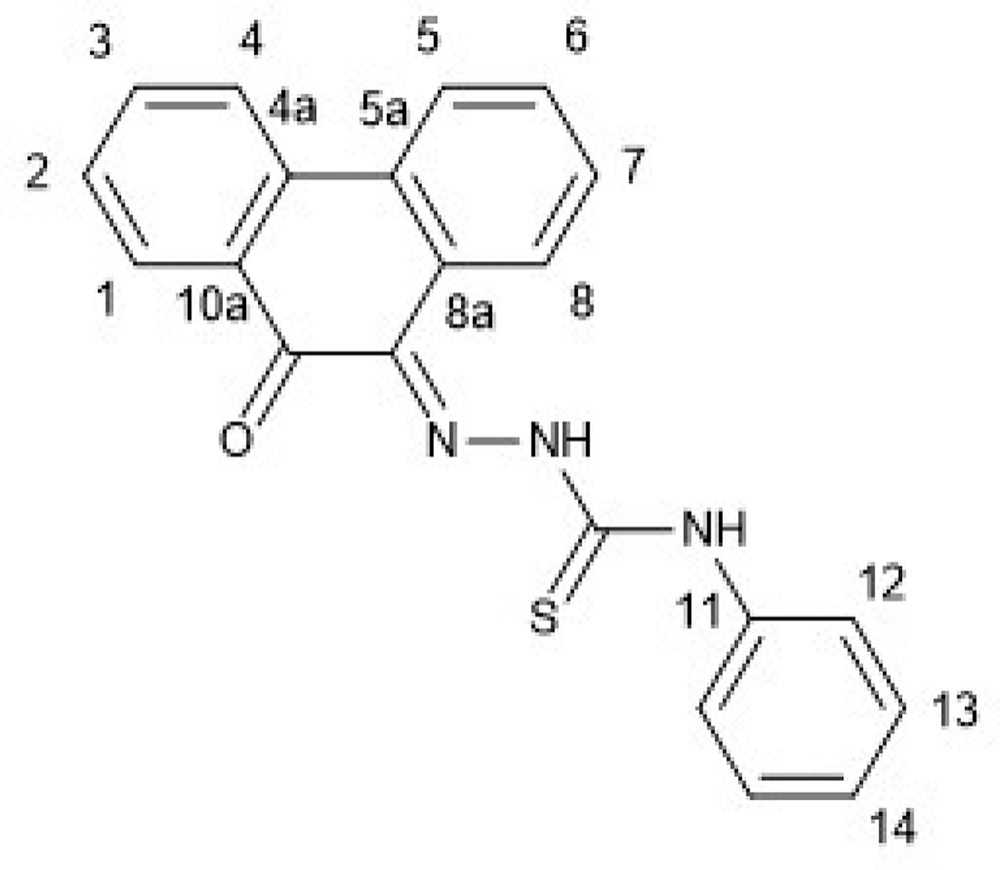


^1^H NMR (500 MHz, d^6^-DMSO, 25 °C): δ 14.79 (s, 1H, NN*H*), 11.08 (s, 1H, CSN*H*), 8.78 (d, *J* = 8.4 Hz, 1H, H-8), 8.43 (dd, *J* = 8.1, 1.0 Hz, 1H, H-4), 8.34 (dd, *J* = 8.3, 1.2 Hz, 1H, H-5), 8.28 (dd, *J* = 7.9, 1.5 Hz, 1H, H-1), 7.86 (ddd, *J* = 8.3, 7.2, 1.5 Hz, 1H, H-3), 7.62–7.59 (m, 2H, H-2, H-6), 7.59–7.56 (m, 1H, H-7), 7.56–7.53 (m, 1H, H-12), 7.52–7.43 (m, 3H, H-12′, H-13), 7.35–7.30 (m, 1H, H-14). ^13^C{^1^H} NMR (125 MHz, d^6^-DMSO, 25 °C): δ 181.5, 177.2, 138.5, 136.1, 135.5, 130.4, 130.3, 129.8, 129.6, 129.0, 128.8, 128.5, 128.4, 128.3, 126.5, 126.2, 125.6, 123.8, 123.7. FTIR (solid): ν (cm^–1^) 3294, 3072, 1631, 1543, 1491, 1413, 1276, 1171, 1114, 1017.

### Synthesis of Mono(4-(*N*-(2-(2-(2-Aminoethoxy)ethoxy)ethyl))-3-thiosemicarbazone) Phenanthrenequinone

Phenanthrenequinone (0.105 g, 0.48 mmol) and the corresponding thiosemicarbazide (0.147 g, 0.46 mmol) were suspended in ethanol (5 mL) and homogenized by sonication for 3 min. Concentrated HCl (3 drops) was added and the reaction mixture heated to 90 °C for 10 min by microwave irradiation. The solvent was removed under vacuum and the product resuspended in CH_2_Cl_2_ and passed through a silica plug. After washing with CH_2_Cl_2_, the product was eluted with CH_2_Cl_2_/MeOH (9:1). The solvent was removed under vacuum and the product obtained as an orange solid. Yield: 58% (0.109 g).
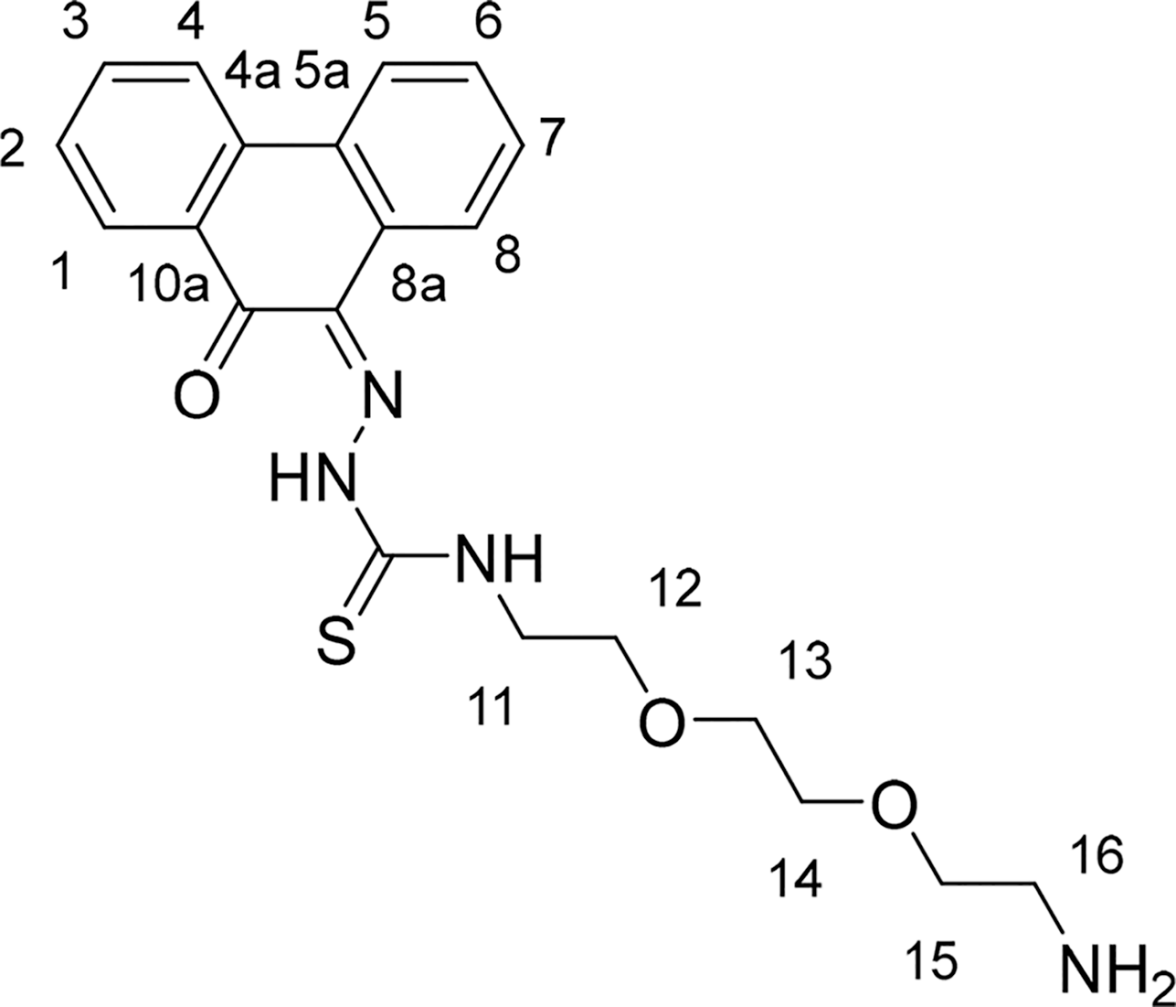


^1^H NMR (500 MHz, d^6^-DMSO, 25 °C): δ 14.57 (s, 1H, NN*H*), 9.60 (t, *J* = 5.9 Hz, 1H, N*H*CH_2_), 8.64 (dd, *J* = 8.1, 1.4 Hz, 1H, H-8), 8.43 (d, *J* = 7.8 Hz, 1H, H-4), 8.34 (dd, *J* = 8.2, 1.2 Hz, 1H, H-5), 8.26 (dd, *J* = 7.9, 1.5 Hz, 1H, H-1), 7.97 (brs, 2H, N*H*_*2*_), 7.85 (ddd, *J* = 8.4, 7.2, 1.5 Hz, 1H, H-3), 7.60–7.54 (m, 2H, H-3, H-2, H-6), 7.50 (ddd, *J* = 8.2, 7.1, 1.2 Hz, 1H, H-7), 3.86 (q, *J* = 6.1 Hz, 2H, H-11), 3.70 (t, *J* = 6.2 Hz, 2H, H-12), 3.64–3.56 (m, 6H, H-13, H-14, H-15), 2.92 (q, *J* = 5.4 Hz, 2H, H-16). ^13^C{^1^H} NMR (125 MHz, d^6^-DMSO, 25 °C): δ 181.4, 177.9, 136.0, 135.4, 130.4, 130.1, 129.8, 129.5, 129.0, 128.5, 128.3, 125.1, 123.8, 69.7, 69.5, 67.8, 66.7, 44.0, 38.5. Mass spectrum: ESI-MS calc. for C_21_H_25_N_4_NaO_3_S [M + Na]^+^: 435.1467; found: 435.1542. IR (solid): ν (cm^–1^) 3300, 1659, 1496, 1388, 1097, 1066. HPLC (Method A): Rt (min) 11.84

### Synthesis of 4,5-Pyrenedione

Sodium metaperiodate (22.050 g, 0.10 mol) and RuCl_3_·*x*H_2_O (0.480 g, 2.32 mmol) were added to pyrene (4.780 g, 23.2 mmol) in CH_2_Cl_2_ (150 mL), THF (150 mL) and H_2_O (200 mL). The reaction mixture was stirred at room temperature for 3 h. The reaction mixture was poured in 1 L of water and the phases separated. The aqueous phase was extracted with CH_2_Cl_2_ (3 × 150 mL). The collected organic phases were washed with water (3 × 150 mL) and dried over MgSO_4_, and the solvent removed under vacuum. The product was purified by flash column chromatography using CH_2_Cl_2_ as eluent. The product was obtained as an orange solid after recrystallization from CH_2_Cl_2_/hexane. Yield: 29% (1.538 g).
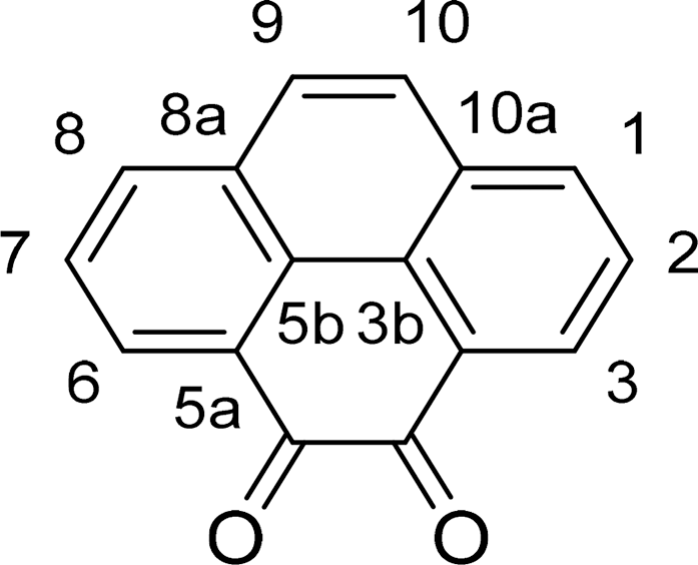


^1^H NMR (300 MHz, CDCl_3_, 25 °C): δ 8.45 (dd, ^*3,4*^*J* = 7.4, 1.1 Hz, 2H, H-3), 8.15 (dd, ^*3,4*^*J* = 8.0, 1.1 Hz, 2H, H-1), 7.82 (s, 2H, H-9), 7.73 (appt, ^*3*^*J* = 7.5 Hz, 2H, H-2). ^13^C{^1^H} NMR (75 MHz, CDCl_3_, 25 °C): δ 180.6 (*C*O), 135.9 (C-1), 132.1 (C-8a), 130.3 (C-3), 130.2 (C-3b), 128.5 (C-2), 128.1 (C-3a)), 127.4 (C-9). Mass spectrum: ESI-MS calc. for C_16_H_9_O_2_ [M + H]^+^: 233.0602; found: 233.0601. IR (solid): ν (cm^–1^) 3048, 2892, 1667, 1614, 1336, 1089. HPLC (Method A): Rt (min) 9.18.

### Synthesis of Mono(3-Thiosemicarbazone) Pyrene-4,5-dione (**PY-H**)

Compound **PY-H** was prepared following the general procedure A. Pyrene-4,5-dione (0.200 g, 0.85 mmol), thiosemicarbazide (0.086 g, 0.82 mmol), and conc. HCl (3 drops) were heated for 10 min at 90 °C under microwave irradiation. The product was obtained as a red solid in quantitative yield.
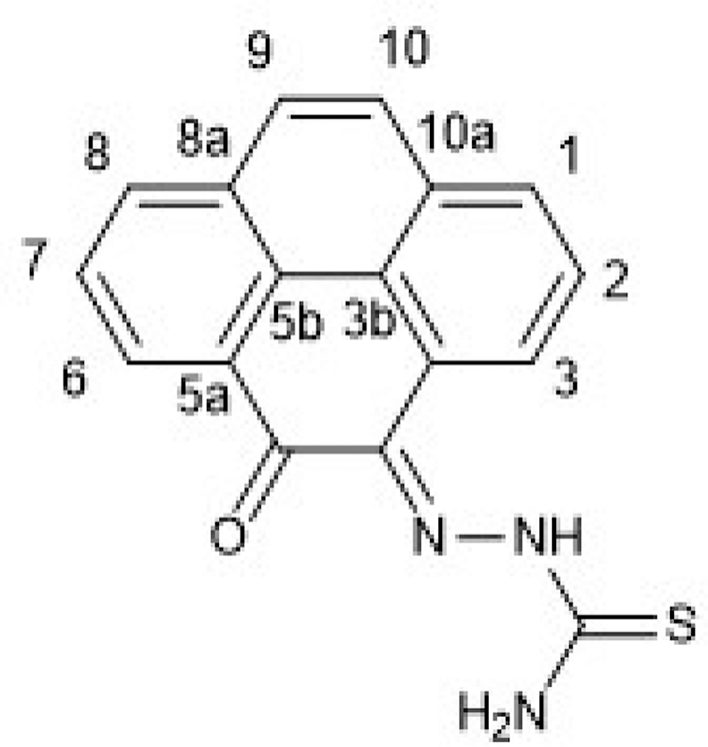


^1^H NMR (500 MHz, d^6^-DMSO, 25 °C): δ 14.49 (s, 1H, N*NH*), 9.40 (s, 1H, *NH*_*2*_), 9.14 (s, 1H, *NH*_*2*_), 8.92 (dd, *J* = 7.8, 1.1 Hz, 1H, H-3), 8.49 (dd, *J* = 7.6, 1.3 Hz, 1H, H-6), 8.39 (dd, *J* = 7.9, 1.3 Hz, 1H, H-8), 8.10 (dd, *J* = 7.9, 1.2 Hz, 1H, H-1), 7.99 (d, *J* = 1.3 Hz, 2H, H-9, H-10), 7.90 (t, *J* = 7.7 Hz, 1H, H-7), 7.79 (t, *J* = 7.8 Hz, 1H, H-2). ^13^C{^1^H} NMR (125 MHz, d^6^-DMSO, 25 °C): δ 181.9, 179.5, 134.4, 131.2, 131.0, 130.6, 129.7, 128.9, 128.6, 128.0, 127.7, 127.6, 127.3, 127.0, 126.3, 123.4, 123.1. Mass spectrum: ESI-MS^+^ calc. for C_17_H_12_N_3_OS [M + H]^+^: 306.0696; found: 306.0698.

### Synthesis of Mono(4-Methyl-3-thiosemicarbazone) Pyrene-4,5-dione (**PY-Me**)

Compound **PY-Me** was prepared following the general procedure A. Pyrene-4,5-dione (0.200 g, 0.86 mmol), 4-methyl-3-thiosemicarbazide (0.086 g, 0.82 mmol) and conc. HCl (3 drops) were heated for 10 min at 90 °C under microwave irradiation. The product was obtained as a red solid. Yield: 83% (0.217 g).
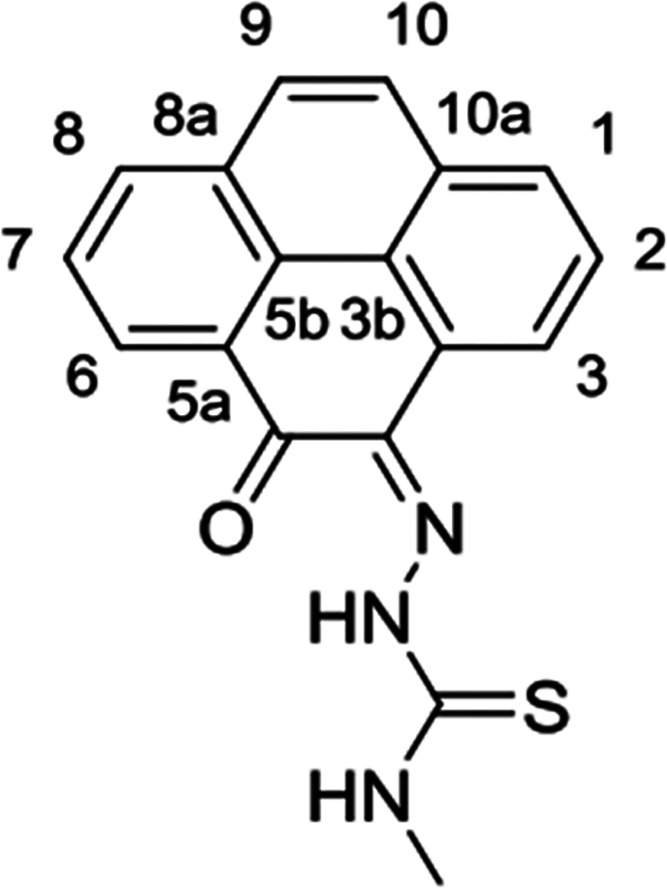


^1^H NMR (500 MHz, d^6^-DMSO, 25 °C): 14.70 (s, 1H, NN*H*), 9.65 (q, *J* = 4.4 Hz, 1H, N*H*CH_3_), 8.93 (dd, *J* = 7.7, 1.2 Hz, 1H, H-3), 8.54 (dd, *J* = 7.5, 1.3 Hz, 1H, H-6), 8.42 (dd, *J* = 7.8, 1.3 Hz, 1H, H-8), 8.13 (dd, *J* = 7.8, 1.1 Hz, 1H, H-1), 8.02 (d, *J* = 1.2 Hz, 2H, H-9, H-10), 7.92 (t, *J* = 7.7 Hz, 1H, H-7), 7.84 (t, *J* = 7.8 Hz, 1H, H-2), 3.19 (d, *J* = 4.6 Hz, 3H, C*H*_3_) ^13^C{^1^H} NMR (125 MHz, d^6^-DMSO, 25 °C): δ 181.9, 178.1, 134.9, 134.4, 131.2, 131.0, 129.8, 129.0, 128.6, 128.5, 128.1, 127.9, 127.6, 127.3, 127.1, 126.4, 123.1, 31.8. Mass spectrum: ESI-MS calc. for C_18_H_13_N_3_NaOS [M + Na]^+^: 342.0677; found: 342.0658. IR (solid): ν (cm^–1^) 3299, 3053, 2932, 1667, 1616, 1543, 1481, 1174. HPLC (Method A): Rt (min) 11.15.

### Synthesis of Mono(4-Ethyl-3-thiosemicarbazone) Pyrene-4,5-dione (**PY-Et**)

Compound **PY-Et** was prepared following the general procedure A. Pyrene-4,5-dione (0.200 g, 0.86 mmol), 4-ethyl-3-thiosemicarbazide (0.098 g, 0.82 mmol), and conc. HCl (3 drops) were heated for 10 min at 90 °C under microwave irradiation. The product was obtained as a red solid in quantitative yield. Yield: 86% (0.236 g).
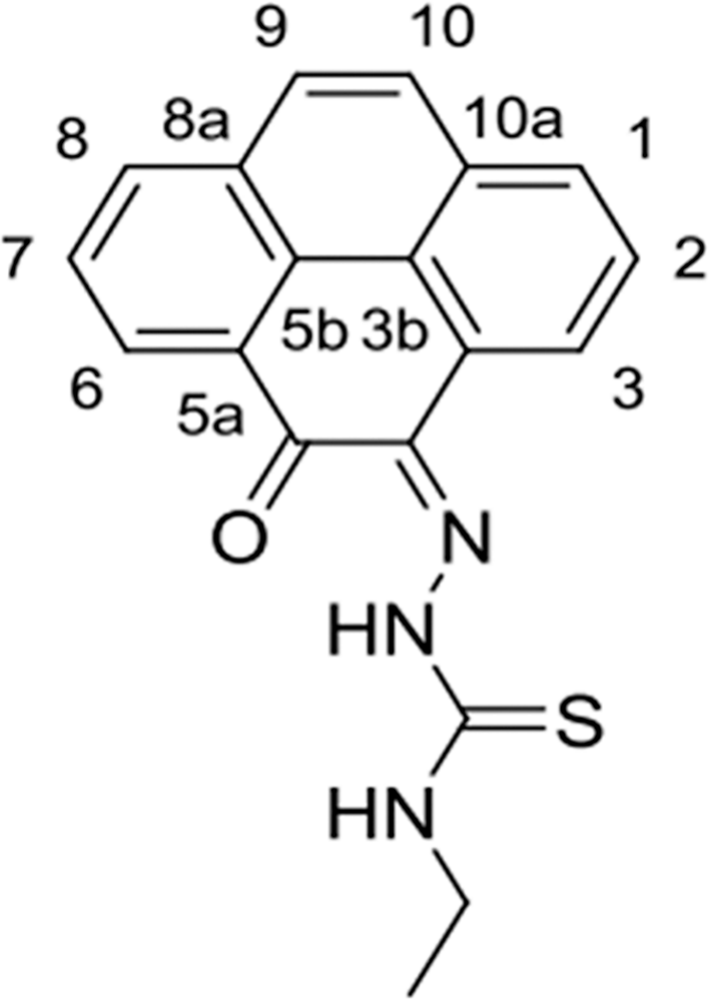


^1^H NMR (500 MHz, d^6^-DMSO, 25 °C): δ 14.64 (s, 1H, NN*H*), 9.72 (t, J = 5.8 Hz, 1H, N*H*CH_2_), 8.90 (dd, J = 7.7, 1.1 Hz, 1H, H-3), 8.52 (dd, J = 7.5, 1.2 Hz, 1H, H-6), 8.42 (dd, J = 7.8, 1.2 Hz, 1H, H-8), 8.13 (dd, J = 7.8, 1.1 Hz, 1H, H-1), 8.01 (s, 2H, H-9, H-10), 7.92 (t, J = 7.7 Hz, 1H, H-7), 7.84 (t, J = 7.8 Hz, 1H, H-2), 3.76 (m, 2H, C*H*_2_), 1.27 (t, J = 7.1 Hz, 3H, C*H*_3_). ^13^C{^1^H} NMR (125 MHz, d^6^-DMSO, 25 °C): δ 181.8, 177.2, 134.4, 131.2, 131.1, 130.5, 129.7, 129.0, 128.6, 128.1, 127.7, 127.3, 127.1, 126.4, 123.1, 39.02, 13.93. Mass spectrum: ESI-MS calc. for C_19_H_15_N_3_NaOS [M + Na]^+^: 356.0834; found: 356.0833. IR (solid): ν (cm^–1^) 3338, 2976, 1670, 1619, 1485, 1416, 1037. HPLC (Method A): Rt (min) 12.00.

### Synthesis of Mono(4-Allyl-3-thiosemicarbazone) Pyrene-4,5-dione (**PY-Allyl**)

Compound **PY-Allyl** was prepared following the general procedure A. Pyrene-4,5-dione (0.200 g, 0.86 mmol), 4-ethyl-3-thiosemicarbazide (0.107 g, 0.82 mmol), and conc. HCl (3 drops) were heated for 10 min at 90 °C under microwave irradiation. The product was obtained as a red solid. Yield: 82% (0.233 g).
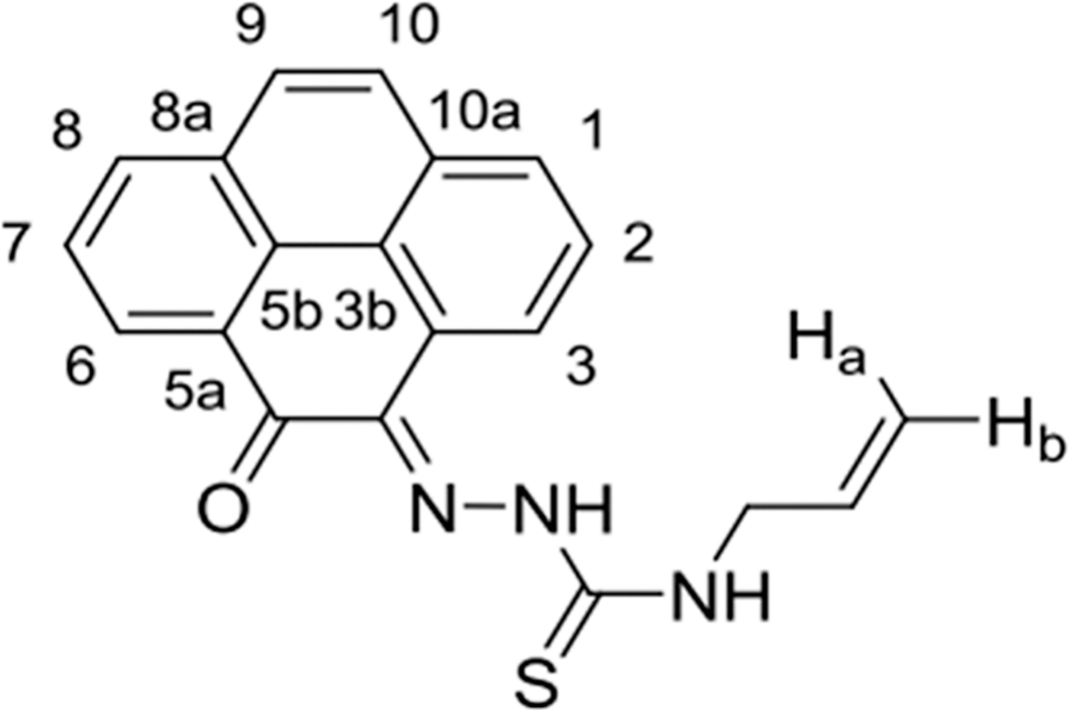


^1^H NMR (500 MHz, d^6^-DMSO, 25 °C): δ 14.72 (s, 1H, NN*H*), 9.89 (t, *J* = 6.0 Hz, 1H, N*H*CH_2_), 8.95 (dd, *J* = 7.8, 1.1 Hz, 1H, H-3), 8.55 (dd, *J* = 7.5, 1.3 Hz, 1H, H-6), 8.43 (dd, *J* = 7.8, 1.3 Hz, 1H, H-8), 8.14 (dd, *J* = 7.8, 1.1 Hz, 1H, H-1), 8.03 (d, *J* = 1.1 Hz, 2H, H-9, H-10), 7.93 (t, *J* = 7.7 Hz, 1H, H-7), 7.85 (t, *J* = 7.8 Hz, 1H, H-2), 5.99 (m, 1H, C*H*), 5.27 (dd, *J*_trans_ = 17.2, 1.7 Hz, 1H, Ha), 5.19 (dd, *J*_cis_ = 10.3, 1.7 Hz, 1H, Hb), 4.47 (m, 2H, C*H*_2_). ^13^C{^1^H} NMR (125 MHz, d^6^-DMSO, 25 °C): δ 181.5, 177.8, 134.2, 133.8, 131.0, 130.9, 130.3, 129.5, 128.7, 128.4, 127.8, 127.5, 127.4, 127.1, 126.2, 123.0, 116.3, 46.7. Mass spectrum: ESI-MS calc. for C_20_H_15_N_3_NaOS [M + Na]^+^: 368.0834; found: 368.0829. IR (solid): ν (cm^–1^) 3354, 1618, 1532, 1484, 1035. HPLC (Method A): Rt (min) 11.31.

### Synthesis of Mono(4-Phenyl-3-thiosemicarbazone) Pyrene-4,5-dione (**PY-Ph**)

Compound **PY-Ph** was prepared following the general procedure A. Pyrene-4,5-dione (0.200 g, 0.86 mmol), 4-phenyl-3-thiosemicarbazide (0.432 g, 2.58 mmol), and conc. HCl (3 drops) were heated for 10 min at 90 °C under microwave irradiation. The product was obtained as a red solid. Yield: 83% (0.274 g).
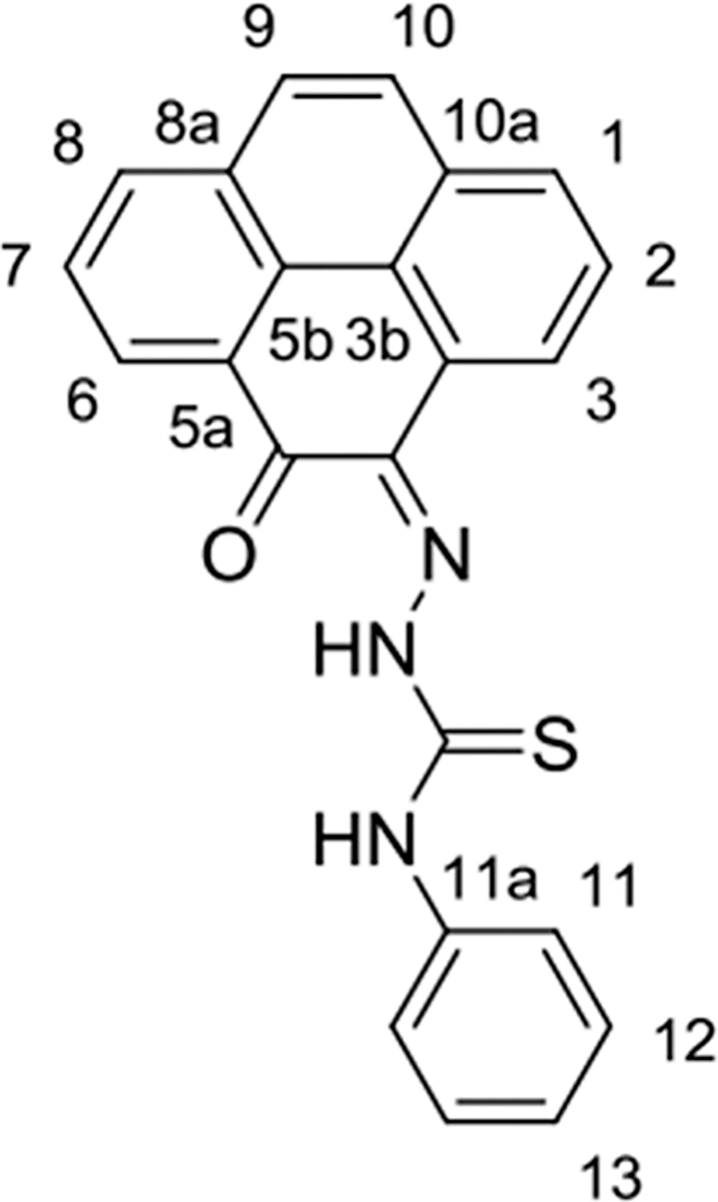


^1^H NMR (500 MHz, d^6^-DMSO, 25 °C): δ 14.82 (s, 1H, NN*H*) 11.12 (s, 1H, CSN*H*), 8.97 (d, *J* = 7.8 Hz, 1H, H-3), 8.48 (dd, *J* = 7.5, 1.3 Hz, 1H, H-6), 8.38 (dd, *J* = 7.8, 1.3 Hz, 1H, H-8), 8.08 (dd, *J* = 7.8, 1.2 Hz, 1H, H-1), 7.98 (s, 2H, H-9, H-10), 7.89 (t, *J* = 7.7 Hz, 1H, H-7), 7.77 (t, *J* = 7.7 Hz, 1H, H-2), 7.65 (dd, *J* = 8.5, 1.3 Hz, 2H, H-11), 7.52–7.46 (m, 2H, H-12), 7.37–7.32 (m, 1H, H-13). ^13^C{^1^H} NMR (125 MHz, d^6^-DMSO, 25 °C): δ 181.9, 177.2, 138.6, 134.5, 131.2, 131.0, 130.9, 129.5, 128.8, 128.7, 128.5, 127.6, 127.3, 127.0, 126.5, 126.3, 126.2, 123.7. Mass spectrum: ESI-MS calc. for C_23_H_15_N_3_NaOS [M + Na]^+^: 404.0833; found: 404.0830. IR (solid): ν (cm^–1^) 3295, 3053, 1638, 1619, 1499, 1130. HPLC (Method A): Rt (min) 12.61.

### Synthesis of Mono(4-*N*(2-(2-(2-Aminoethoxy)ethoxy)ethyl))-3-thiosemicarbazone Pyrene-4,5-dione

Pyrene-4,5-dione (0.100 g, 0.43 mmol) and corresponding thiosemicarbazide (0.132 g, 0.41 mmol) were suspended in ethanol (5 mL), homogenized by sonication for 3 min and heated 10 min at 90 °C in the microwave reactor. The solvent was removed under vacuum and the residue resuspended in CH_2_Cl_2_ and passed through a silica plug washing with CH_2_Cl_2_ and eluting with CH_2_Cl_2_/MeOH (9:1). The solvent was removed under vacuum and the product was obtained as a red solid. Yield: 68% (0.121 g).
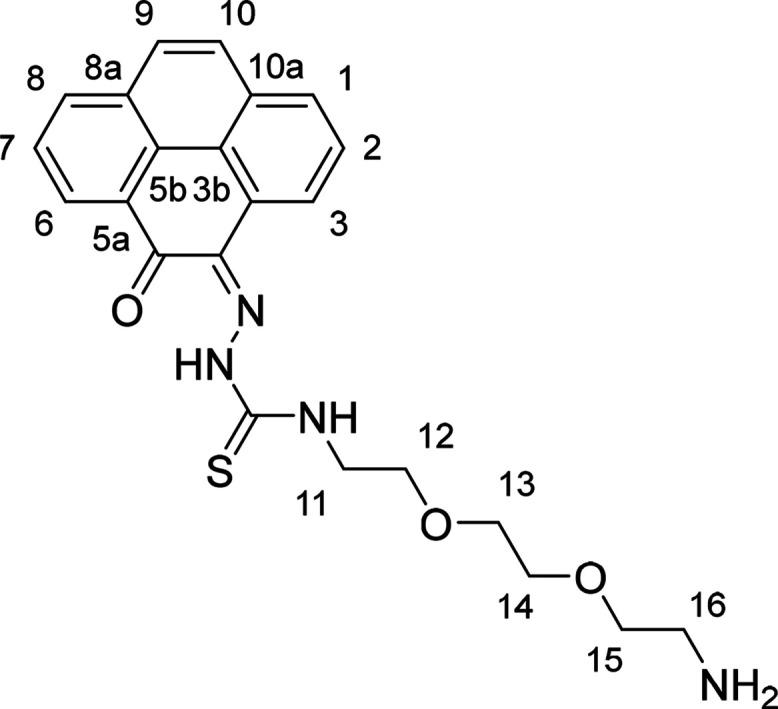


^1^H NMR (400 MHz, CDCl_3_, 25 °C): δ 14.67 (s, 1H, NN*H*), 9.68 (t, *J* = 5.9 Hz, 1H, N*H*CH_2_), 8.92 (dd, *J* = 7.7, 1.1 Hz, 1H, H-3), 8.54 (dd, *J* = 7.5, 1.3 Hz, 1H, H-6), 8.44 (dd, *J* = 7.9, 1.3 Hz, 1H, H-8), 8.15 (dd, *J* = 7.9, 1.1 Hz, 1H, H-1), 8.04 (d, *J* = 0.8 Hz, 2H, H-9, H-10), 7.93 (t, *J* = 7.7 Hz, 1H, H-7), 7.89 (brs, 2H, N*H*_*2*_), 7.85 (t, *J* = 7.8 Hz, 1H, H-2), 3.91 (q, *J* = 6.1 Hz, 2H, H-11), 3.75 (t, *J* = 6.2 Hz, 2H, H-12), 3.68–3.60 (m, 6H, H-13, H-14, H-15), 2.98–2.91 (m, 2H, H-16). ^13^C{^1^H} NMR (125 MHz, CDCl_3_, 25 °C): δ 182.0, 178.0, 134.6, 131.3, 131.1, 131.0, 129.7, 129.0, 128.7, 128.1, 127.7, 127.4, 127.2, 126.4, 123.2, 69.7, 69.6, 67.9, 66.7, 44.1, 38.6. Mass spectrum: ESI-MS calc. for C_23_H_25_N_4_O_3_S [M + H]^+^: 437.1647; found: 437.1656. IR (solid): ν (cm^–1^) 3300, 1659, 1496, 1388, 1097, 1066. HPLC (Method A): Rt (min) 11.84.

### Synthesis of Mono(4-*tert*-Butyl-4-((((9*H*-fluoren-9-yl)methoxy)carbonyl)amino)-5-oxo-5-(propylamino)pentanoyl)-3-thiosemicarbazone) Acenaphthenequinone

A solution of Fmoc-Glu(O^t^Bu)-OH (0.214 g, 0.50 mmol), pyBOP (0.262 g, 0.50 mmol), and DIPEA (0.11 mL, 0.63 mmol) were stirred for 2 h at room temperature in DMF (5 mL). Mono(thiosemicarbazone) **10** (0.125 g, 0.42 mmol) was added to the reaction mixture dissolved in DMF (5 mL) and the reaction mixture stirred at room temperature for 20 h. The solvent was removed under vacuum; the crude was redissolved in CH_2_Cl_2_ and purified by column chromatography using CH_2_Cl_2_/MeOH (0–10%) as solvent system. The solvent was concentrated under vacuum to yield a yellow solid. Yield: 49% (0.147 g).
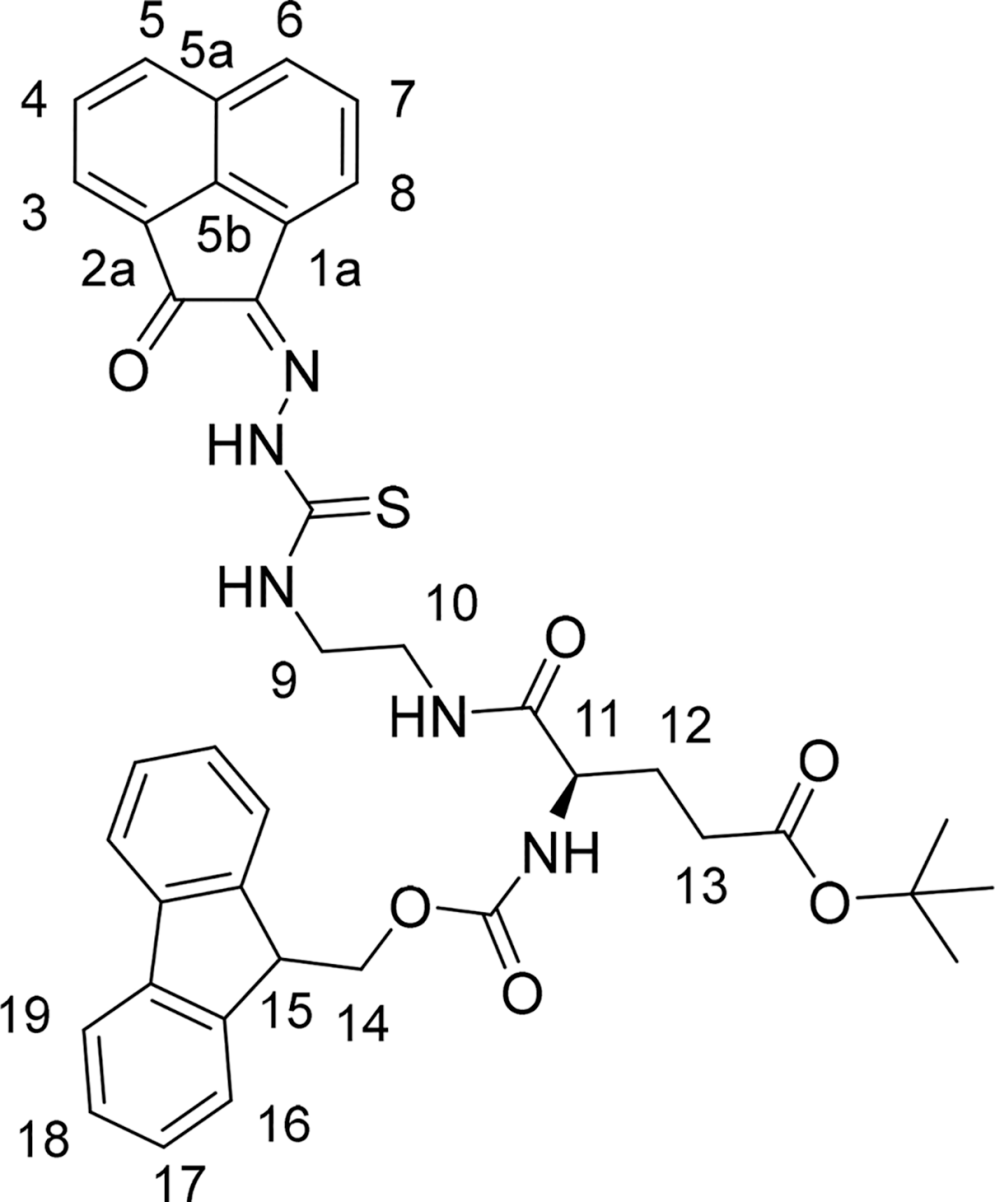


^1^H NMR (500 MHz, d^6^-DMSO, 25 °C): δ 12.61 (s, 1H, NN*H*), 9.36 (t, *J* = 5.4 Hz, 1H, CSN*H*), 8.35 (d, *J* = 8.2 Hz, 1H, H-5), 8.20 (t, *J* = 6.0 Hz, 1H, C-10-N*H*), 8.10 (d, *J* = 8.5 Hz, 1H, H-6), 8.07 (d, *J* = 7.1 Hz, 2H, H-3), 8.02 (d, *J* = 7.0 Hz, 1H, H-8), 7.90–7.86 (m, 1H, H-4), 7.86–7.81 (m, 2H, H-19, H-19’), 7.78 (dd, *J* = 8.9, 7.0, 2H, H-7), 7.70 (d, *J* = 7.5 Hz, 2H, H-16, H-16’), 7.66 (d, *J* = 7.7 Hz, 1H, H-17, H-17’), 7.54 (d, *J* = 7.4 Hz, 1H, C-11-N*H*), 7.38 (t, *J* = 7.5 Hz, 2H, H-18, H-18’), 7.29 (t, *J* = 7.5 Hz, 2H, H-17, H-17’), 4.36–4.11 (m, 3H, H-14, H-16), 4.09–3.94 (m, 2H, H-11), 3.77–3.64 (m, 2H, H-9), 3.51–3.38 (m, 2H, H-10), 2.23 (t, ^*3*^*J* = 7.7 Hz, 3H, H-13), 2.05–1.87 (m, 1H, H-12), 1.87–1.70 (m, 2H, H-12). ^13^C{^1^H} NMR (125 MHz, d^6^-DMSO, 25 °C): δ 188.9, 178.1, 172.6, 172.0, 156.4, 144.3, 144.1, 141.1, 139.6, 137.6, 133.3, 130.8, 130.4, 130.3, 129.3, 129.1, 129.0, 128.0, 127.5, 127.4, 125.7, 122.9, 120.5, 118.8, 66.1, 54.5, 47.1, 44.9, 38.1, 31.8, 28.1, 27.7. Mass spectrum: ESI-MS calc. for C_39_H_39_N_5_NaO_6_S [M + Na]^+^: 728.2518; found: 728.2580. HPLC (Method A): Rt (min) 10.96.

### General Microwave-Assisted Synthesis of Zn(II) Mono(Thiosemicarbazonato) Complexes

Mono (substituted) thiosemicarbazone acenaphthenequinone (2.74 mmol) and 0.5–1 equiv of anhydrous zinc acetate (0.5450 g, 2.74 mmol) were suspended in 5 mL of ethanol and sonicated for 2–3 mind. The mixture was reacted at 90 °C under microwave irradiation for 60 min. The slurry was then filtrated and washed with diethyl ether. The precipitate was collected, further washed with Et_2_O to afford the desired compounds, incorporating one Zn center ligated to two monoanionic ligands for either the 1:1 or 2:1 ligand: Zn reactions (as powders of orange-yellow to red colors) with good yields (generally above 50%). Analytical HPLC data indicated that no further purification was necessary after the Et_2_O wash; however, further details are given below for individual compounds.

### Synthesis of Zinc Mono(4-Allyl-3-thiosemicarbazone) Aceanthrenequinone Zn(**AA-Allyl**)_2_

The ligand ****AA-Allyl**** (0.048 g, 0.14 mmol) and anhydrous zinc acetate (0.027 g, 0.14 mmol) were heated in ethanol for 1 h at 90 °C under microwave irradiation. Resuspension of the solid in diethyl ether yielded a red colored compound that was further washed with Et_2_O to remove impurities and dried under reduced pressure. The product was obtained as a red solid in quantitative yield.

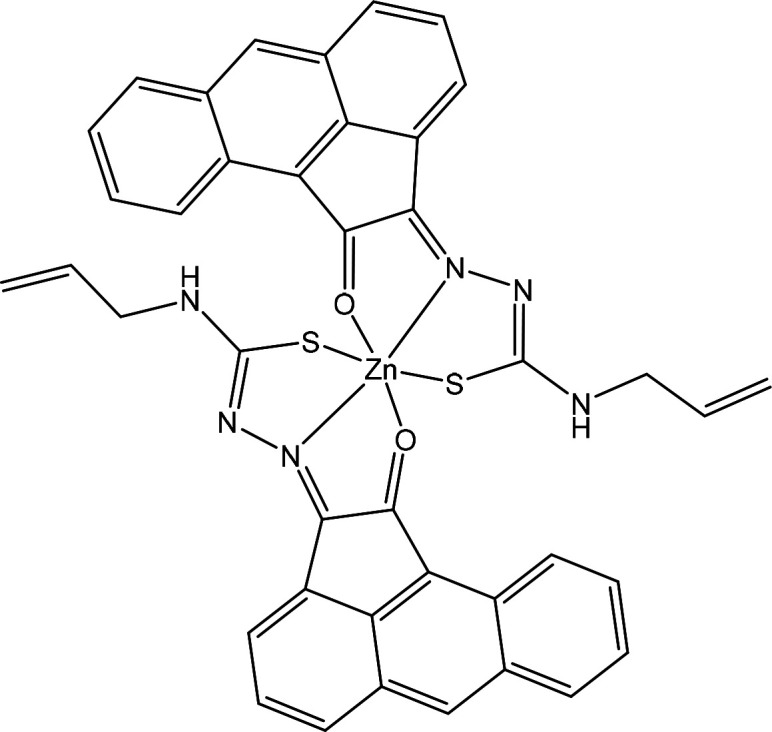



Mass spectrum: nanoESI-MS calc. for C_40_H_29_N_6_O_2_Zn [M + H]^+^: 753.1085; found: 753.1079. IR (solid): ν (cm^–1^) 3300, 1659, 1496, 1388, 1097, 1066. HPLC (Method A): Rt (min) 11.84.

### Alternative Procedure for the Synthesis of Zinc Mono(4-Allyl-3-thiosemicarbazone) Aceanthrenequinone Zn(**AA-Allyl**)_2_ by Conventional Heating

Compound **AA-Allyl** (0.025 g, 0.072 mmol) and zinc acetate (0.016 g, 0.072 mmol) were added together in a 1:1 ratio with 20 mL of THF as the solvent. The reaction proceeded for 3 h at room temperature, then the solvent was removed by evaporation. Resuspension of the solid in diethyl ether yielded a red colored compound that was further washed with Et_2_O to remove impurities and dried under reduced pressure. (0.049 g, 79%)

Mass spectrum: ESI-MS calc. for C_40_H_28_N_6_O_2_S_2_Zn [M + H]^+^: 753.1085; found: 753.1077.

### Synthesis of Zinc Mono(4-Allyl-3-thiosemicarbazone) 9,10-Pheanthrenequinone [Zn(**PH-Allyl**)_2_]

The compound **PH-Allyl** (0.051 g, 0.16 mmol) and anhydrous zinc acetate (0.028 g, 0.16 mmol) were heated together in ethanol for 1 h at 90 °C under microwave irradiation. Resuspension of the solid in diethyl ether yielded a red colored compound that was further washed with Et_2_O to remove impurities and dried under reduced pressure. The product was obtained as a red solid. Yield: 84% (0.046 g).
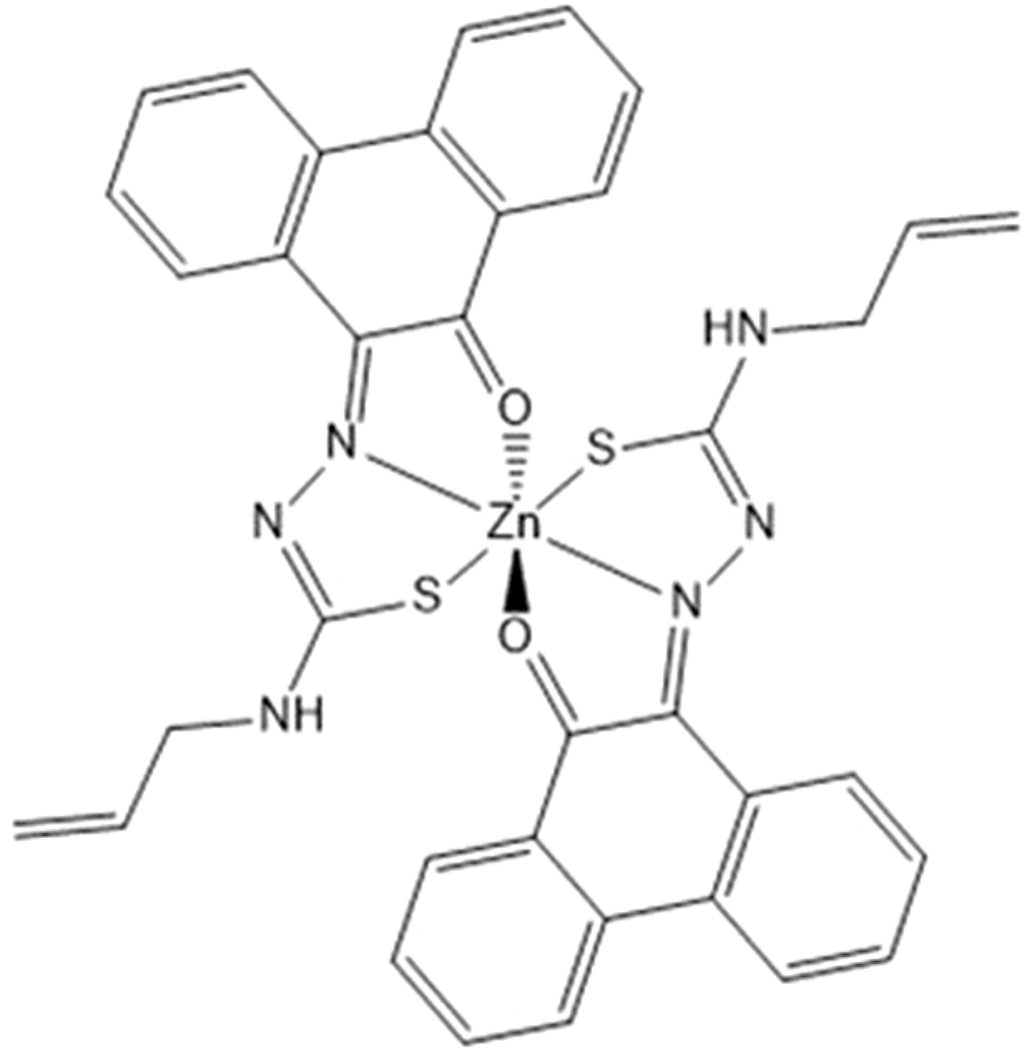


Mass spectrum: ESI-MS calc. for C_36_H_29_N_6_O_2_S_2_Zn [M + H]^+^: 705.1085; found: 705.1200. IR (solid): ν (cm^–1^) 3313, 2961, 2925, 1486, 1078, 1014. HPLC (Method A): Rt (min) 11.26.

### Alternative Procedure for the Synthesis of Zinc Mono(4-Allyl-3-thiosemicarbazone) 9,10-Pheanthrenequinone [Zn(**PH-Allyl**)_2_] by Conventional Heating

Compound **PH-Allyl** (0.025 g, 0.078 mmol) was reacted with zinc acetate (0.017 g, 0.078 mmol). Approximately 20 mL of THF was added as the solvent, stirring the solution for 3 h. Then, following filtration, washing with diethyl ether and removal of volatiles, a dark orange powder was isolated. Yield: 88% (0.048 g).

Mass spectrum: ESI-MS calc. for C_36_H_28_N_6_O_2_S_2_Zn [M + H]^+^: 705.1085; found: 705.1100.

### Synthesis of Zn Mono(4-allyl-3-thiosemicarbazonato) Pyrene-4,5-dione (ML_2_)

Compound **PY-Allyl** (0.050 g, 0.14 mmol) and anhydrous zinc acetate (0.026 g, 0.14 mmol) were heated in ethanol for 1 h at 90 °C under microwave irradiation, according to the general procedure, above. The product was obtained as a dark red solid, in quantitative yield.
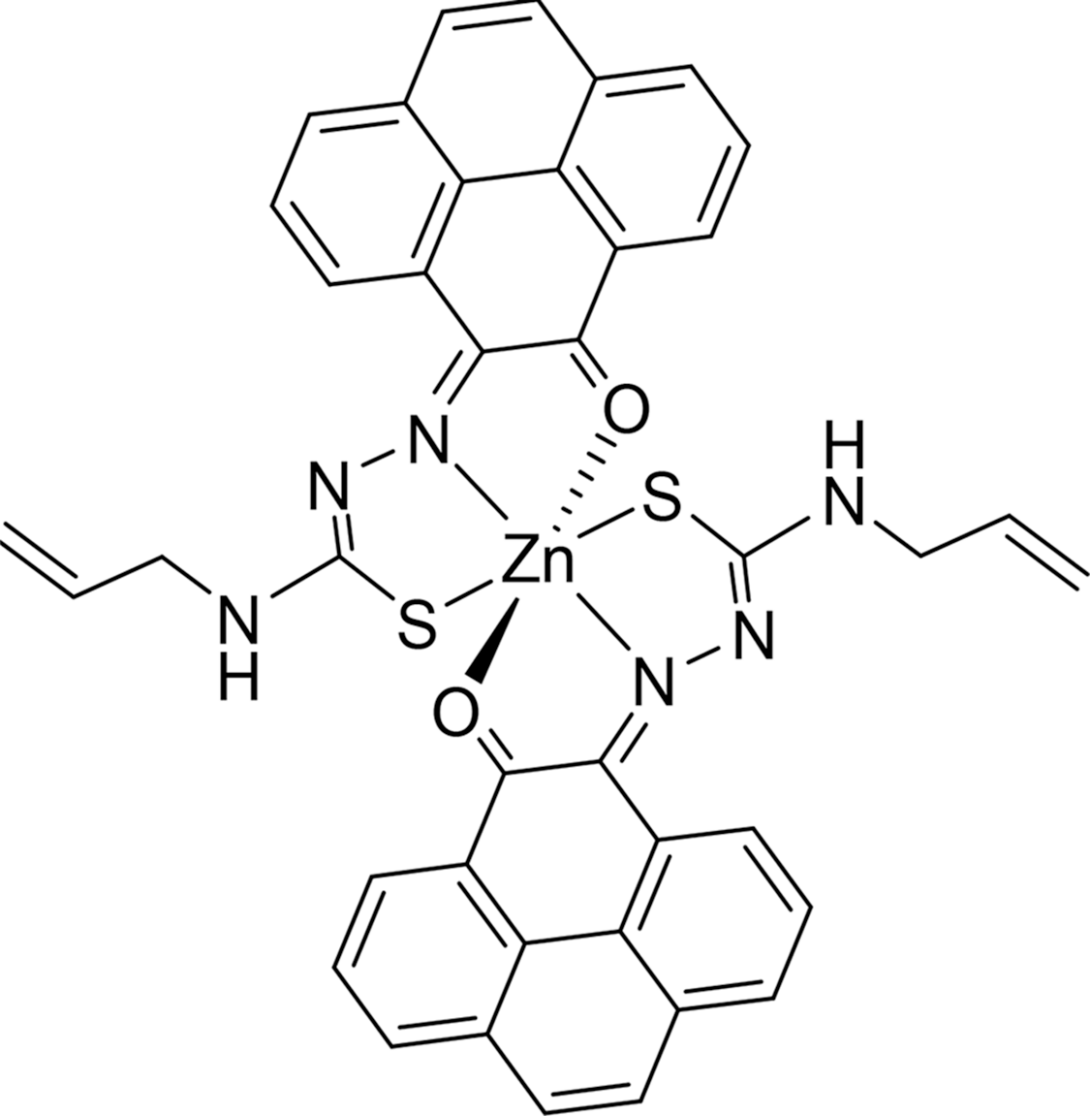


Mass spectrum: ESI-MS calc. for C_40_H_29_N_6_O_2_S_2_Zn [M + H]^+^: 753.1085; found: 753.1186. IR (solid): ν (cm^–1^) 3355, 2918, 1679, 1484, 1075, 1026. HPLC (Method A): Rt (min) 12.15.

### Zn(II)(Mono(3-Thiosemicarbazonato) Acenaphthenequinone)_2_ [Zn(**AN-H**)_2_]

The ligand ****AN-H**** (0.035 g, 0.14 mmol) and anhydrous zinc acetate (0.027 g, 0.14 mmol) were heated in ethanol for 1 h at 90 °C under microwave irradiation. The mixture was filtered; then, it was resuspended in diethyl ether and filtered. This yielded a red colored compound that was further washed with Et_2_O to remove impurities and dried under reduced pressure. The product was obtained as a red solid after the concentration of volatiles and washing with Et_2_O in quantitative yield.

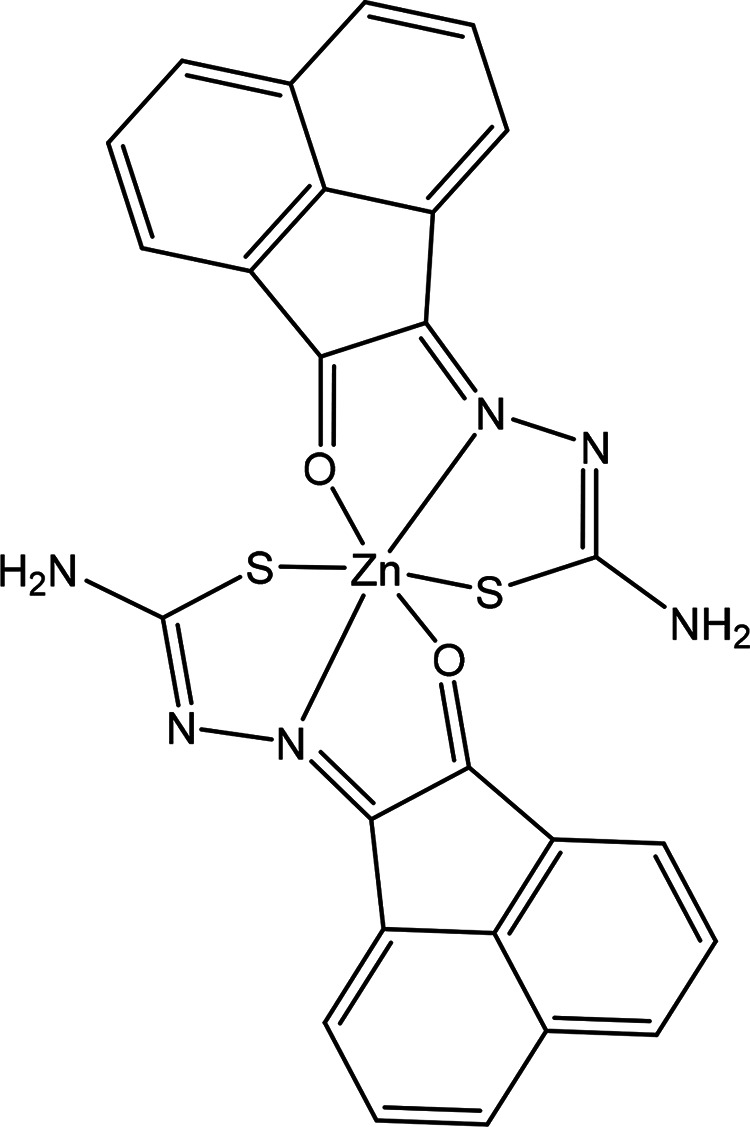



Mass spectrum: NSI-MS calc. for C_26_H_17_N_6_O_2_S_2_Zn [M + H]^+^: 573.0140; found: 573.0135.

### Zn(II)(Mono(3-Thiosemicarbazonato) Aceanthrenequinone)_2_ [Zn(AA-H)_2_]

The ligand **AA-H** (0.043 g, 0.14 mmol) and anhydrous zinc acetate (0.027 g, 0.14 mmol) were heated in ethanol for 1 h at 90 °C under microwave irradiation. The mixture was filtered, then resuspended in diethyl ether and filtered. This yielded a red colored compound that was further washed with Et_2_O to remove impurities and dried under reduced pressure. The desired product was obtained in quantitative yield.
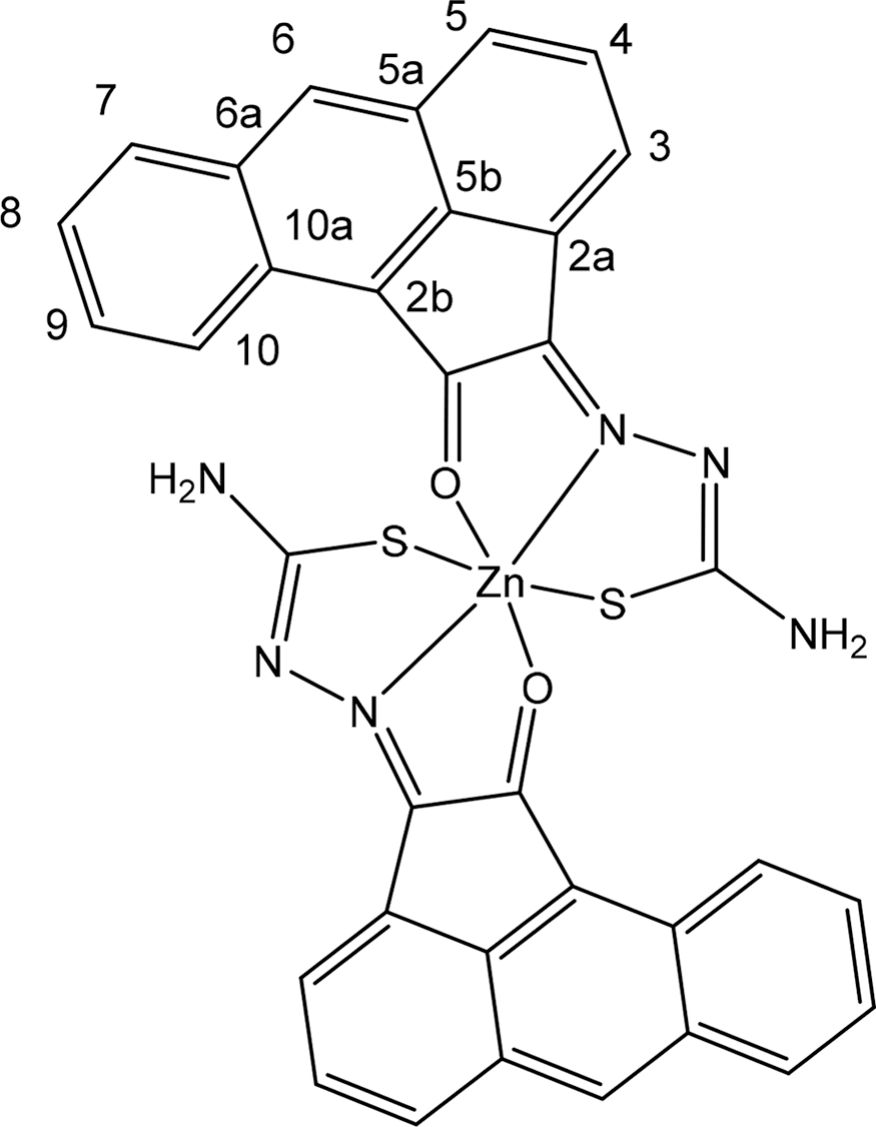


^1^H NMR (400 MHz, d^6^-DMSO, 25 °C): δ 9.07 (s, 2H, N*H*_*2*_), 8.72 (d, *J* = 6.7 Hz, 2H, N*H*_*2*_), 8.70 (m, 6H, H-6, H-7, H-10), 8.31 (d, *J* = 8.4 Hz, 2H, H-5), 8.25 (d, *J* = 8.7 Hz, 2H, H-3), 7.86 (dd, *J* = 8.8, 6.7 Hz, 2H, H-9), 7.72–7.67 (m, 2H, H-4), 7.63 (m, 2H, H-8). Mass spectrum: NSI-MS calc. for C_34_H_21_N_6_O_2_S_2_Zn [M + H]^+^: 673.0453; found: 673.0449.

### Zn(II)(Mono(4-Ethyl-3-thiosemicarbazonato) Aceanthrenequinone)_2_ [Zn(**AA-Et**)_2_]

The ligand **AA-Ethyl** (0.047 g, 0.14 mmol) and anhydrous zinc acetate (0.027 g, 0.14 mmol) were heated in ethanol for 1 h at 90 °C under microwave irradiation, according to the general procedure, given above. The mixture was filtered, then resuspended in diethyl ether and filtered. This yielded a red colored compound that was further washed with Et_2_O to remove impurities and dried under reduced pressure. The product was obtained as a red solid (0.030 g, 63%).

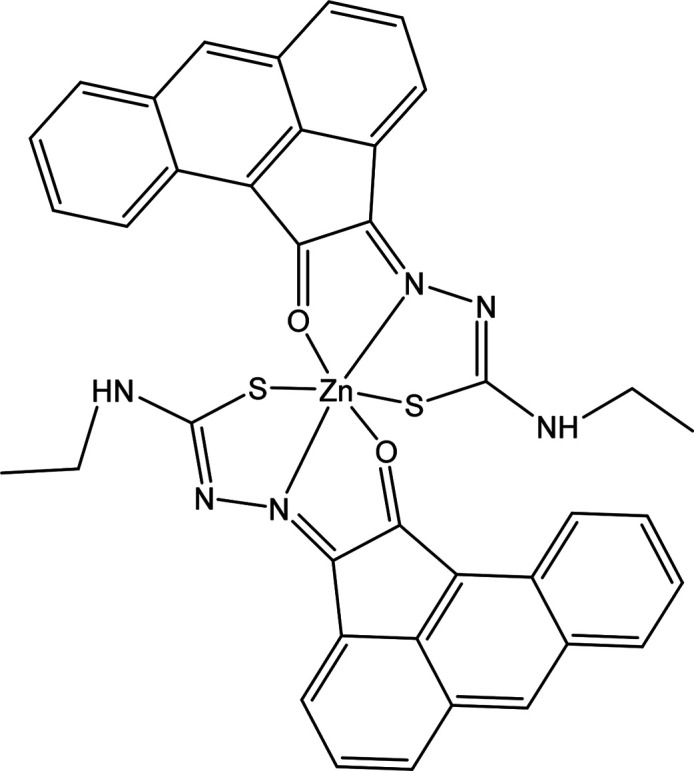



Mass spectrum: ASAP-MS calc. for C_38_H_29_N_6_O_2_S_2_Zn [M + H]^+^: 729.1085; found: 729.1081.

### Zn(II)(Mono(3-Thiosemicarbazonato) Phenanthrenequinone)_2_ [Zn(**PH-H**)_2_]

The compound **PH-H** (0.045 g, 0.16 mmol) and anhydrous zinc acetate (0.028 g, 0.16 mmol) were heated together in ethanol for 1 h at 90 °C under microwave irradiation following the general procedure given above. Resuspension of the solid in diethyl ether yielded a red colored compound that was further washed with Et_2_O to remove impurities and dried under reduced pressure. The product was obtained as an orange solid in quantitative yield.
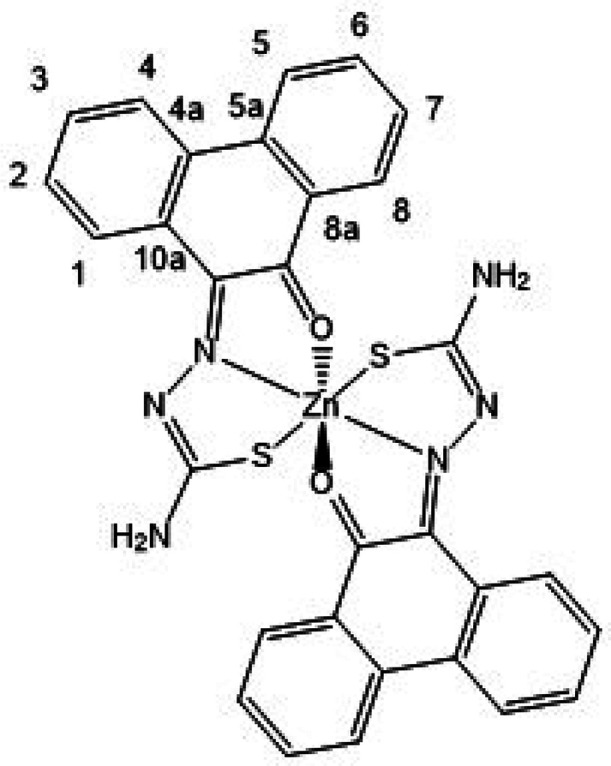


^1^H NMR (400 MHz, d^6^-DMSO, 25 °C): δ 10.16 (dd, *J* = 7.7, 1.9 Hz, 1H, H-8), 9.99 (d, *J* = 5.7, 1H, H-8), 9.74–9.43 (m, 4H, N*H*_*2*_), 8.58–8.48 (m, 4H, H-4, H-5), 8.36 (d, *J* = 7.9 Hz, 1H, H-1), 8.18 (dd, *J* = 8.0, 1.5 Hz, 1H, H-1), 7.89 (t, *J* = 7.8 Hz, 1H, H-3), 7.86–7.80 (m, 1H, H-3), 7.65–7.48 (m, 6H, H-2, H-6, H-7). Mass spectrum: NSI-MS calc. for C_30_H_21_N_6_O_2_S_2_Zn [M + H]^+^: 625.0453; found 625.0447.

### Zn(II)(Mono(4-Ethyl-3-thiosemicarbazonato) Phenanthrenequinone)_2_ [Zn(**PH-Et**)_2_]

The compounds **PH-Et** (0.051 g, 0.16 mmol) and anhydrous zinc acetate (0.028 g, 0.16 mmol) were suspended in 5 mL of ethanol and sonicated (2–3 min.) Note that for the reaction carried out in a 1:0.5 molar ratio, the compounds **PH-Et** (0.051 g, 0.16 mmol) and anhydrous zinc acetate (0.015g, 0.08 mmol) were charged to a 10 mL microwave tube and sonicated 2–3 min. In both cases, the reagents were heated together for 1 h at 90 °C under microwave irradiation (according to the general procedure, above) and filtered under reduced pressure. Resuspension of the solid in diethyl ether yielded a red colored compound that was further washed with Et_2_O to remove impurities and dried under reduced pressure. In both 1:1 and 1:0.5 Ligand:Zn(II) ratio reactants, the same product was obtained as an orange solid. The product from 1:1 reaction was obtained in 36% yield (0.0185 g).
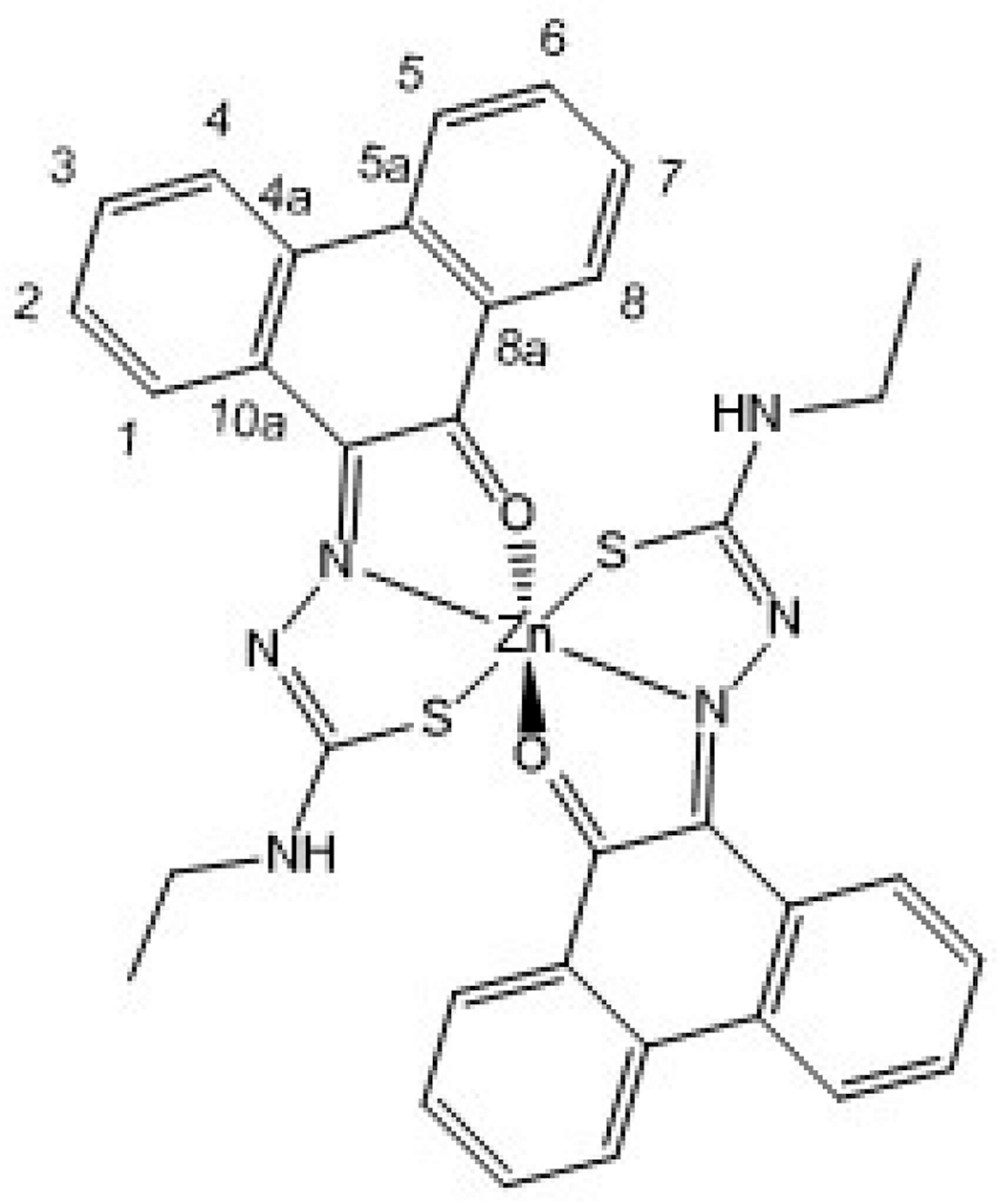


^1^H NMR (500 MHz, d^6^-DMSO, 25 °C): δ 10.34 (t, *J* = 5.8 Hz, 2H, N*H*CH_2_), 10.13 (dd, *J* = 8.2, 1.4 Hz, 2H, H-8), 8.55 (dd, *J* = 8.2, 1.9 Hz, 4H, H-4, H-5), 8.20 (dd, *J* = 8.1, 1.5 Hz, 2H, H-1), 7.83 (ddd, *J* = 8.4, 7.2, 1.6 Hz, 2H, H-3), 7.62 (ddd, *J* = 8.3, 7.1, 1.5 Hz, 2H, H-2), 7.57 (ddd, *J* = 8.6, 7.2, 1.4 Hz, 2H, H-6), 7.48 (ddd, *J* = 8.1, 7.5, 1.9 Hz, 2H, H-7), 3.72–3.64 (m, 4H, C*H*_2_), 1.26 (td, *J* = 7.2, 1.7 Hz, 6H, C*H*_3_). Mass spectrum: NSI-MS calc. for C_34_H_29_N_6_O_2_S_2_Zn [M + H]^+^: 681.1079; found: 681.1083. FTIR (solid): ν (cm^–1^) 3188, 3068, 1649, 1536, 1386, 1266, 1154, 1021.

### Zn(II)(Mono(3-Thiosemicarbazonato) Pyrene-4,5-dione)_2_ [Zn(**PY-H**)_2_]

The compound **PY-H** (0.043 g, 0.14 mmol) and anhydrous zinc acetate (0.026 g, 0.14 mmol) were heated in ethanol for 1 h at 90 °C under microwave irradiation, according to the general procedure, above. The product was obtained as a dark red solid, in quantitative yield.

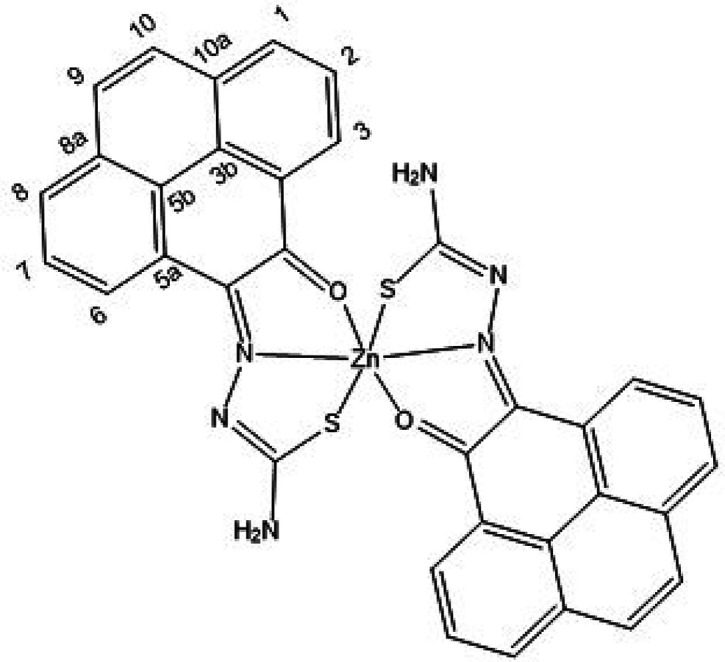



^1^H NMR (400 MHz, d^6^-DMSO, 25 °C): δ 10.47 (d, *J* = 7.9 Hz, 1H, *NH*_*2*_), 10.27 (d, *J* = 8.0 Hz, 1H, *NH*_*2*_), 9.80–9.58 (m, 4H, *NH*_*2*_, H-3), 8.71 (d, *J* = 7.7 Hz, 1H, H-6), 8.56–8.49 (m, 2H, H-8), 8.46 (d, *J* = 8.0 Hz, 1H, H-6), 8.21–8.08 (m, 6H, H-1, H-9, H-10), 7.99 (m, 2H, H-7), 7.92 (t, *J* = 7.9 Hz, 1H, H-2), 7.84 (t, *J* = 7.7 Hz, 1H, H-2). Mass spectrum: NSI-MS calc. for C_34_H_21_N_6_O_2_S_2_Zn [M + H]^+^: 673.0453; found: 673.0449.

### Zn(II)(Mono(4-Ethyl-3-thiosemicarbazonato) Pyrene-4,5-dione)_2_ [Zn(**PY-Et**)_2_]

Compound **PY-Et** (0.040 g, 0.12 mmol) and anhydrous zinc acetate (0.022 g, 0.12 mmol) were heated in ethanol for 1 h at 90 °C under microwave irradiation, according to the general procedure, above. After washing with Et_2_O, the product was obtained as a dark red solid in quantitative yield.
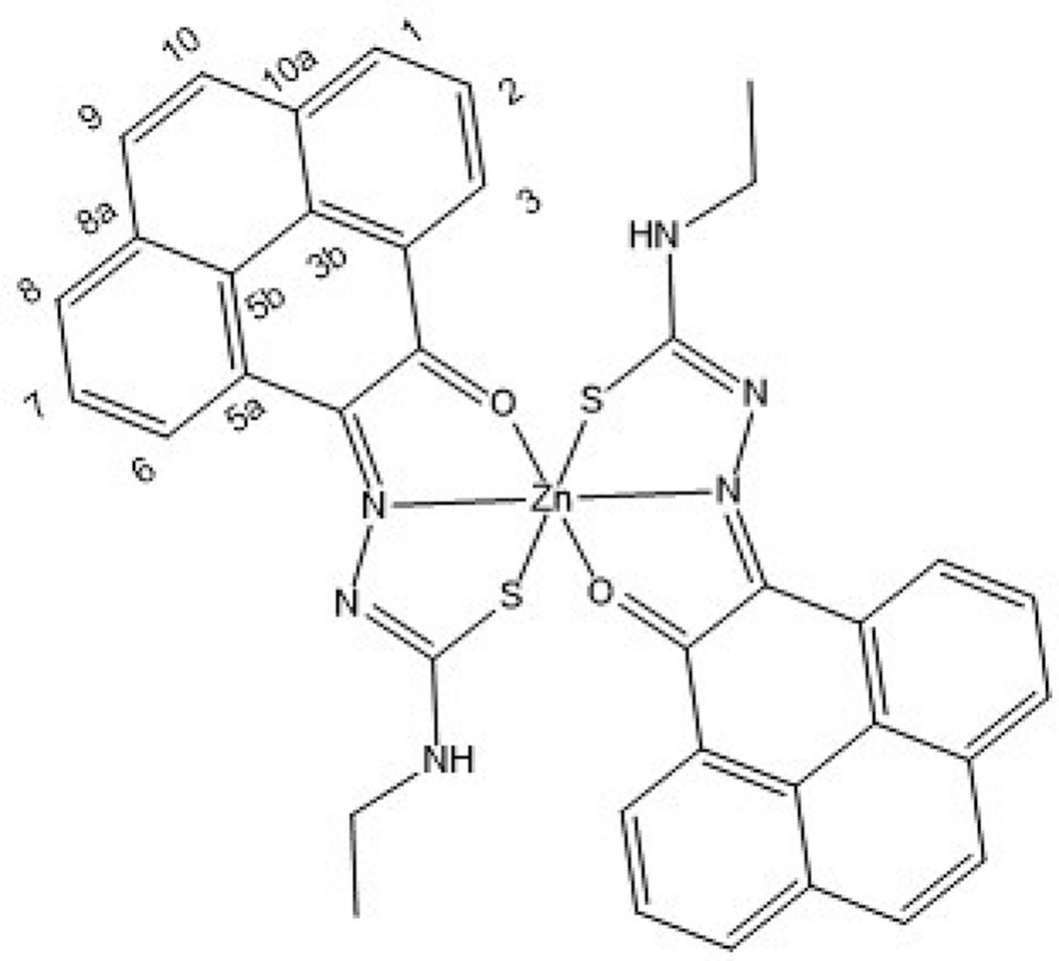


^1^H NMR (400 MHz, d^6^-DMSO, 25 °C): δ 10.49 (m, 2H, *NH*CH_2_), 10.44 (d, *J* = 7.9 Hz, 1H, H-3), 10.23 (d, *J* = 7.8 Hz, 1H, H-3), 8.73 (d, *J* = 7.4 Hz, 1H, H-8), 8.53 (dd, *J* = 16.1, 7.6 Hz, 2H, H-6), 8.45 (d, *J* = 7.6 Hz, 1H, H-8), 8.12 (m, 6H, H-1, H-9, H-10), 8.00 (td, *J* = 7.8, 2.8 Hz, 2H, H-7), 7.93 (t, *J* = 8.0 Hz, 1H, H-2), 7.85 (t, *J* = 7.5 Hz, 1H, H-2), 3.81 (m, 2H, C*H*_2_), 3.72 (m, 2H, C*H*_2_), 1.34 (t, *J* = 7.1 Hz, 3H, C*H*_3_), 1.29 (t, *J* = 7.3 Hz, 3H, C*H*_3_). Mass spectrum: NSI-MS calc. for C_38_H_29_N_6_O_2_S_2_Zn [M + H]^+^: 729.1079; found: 729.1088.

### Synthesis of Copper Mono(4-Methyl-3-thiosemicarbazone) Acenaphthenequinone [Cu(**AN-Me**)_2_]

One equivalent of compound ****AN-Me**** (0.027 g, 0.100 mmol) and one equivalent of copper acetate (0.018 g, 0.100 mmol) were suspended in 20 mL of MeOH and stirred for 3 h at room temperature. The solvent was concentrated removed using a rotavapor, and then, to the resulting slurry, diethyl ether was added dropwise until a precipitate formed. This was then filtered and washed with diethyl ether and dried under vacuum. The final crude product was obtained as a black color solid.

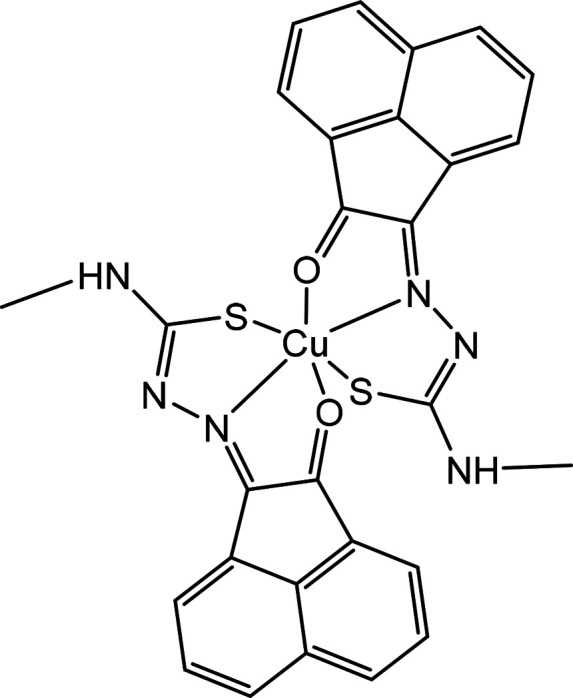



Mass spectrum: ESI-MS calc. for C_28_H_20_N_6_O_2_S_2_Cu [M + H]^+^: 600.0458; found: 600.0485.

### Alternative Procedure for the Synthesis of Copper Mono(4-Methyl-3-thiosemicarbazone) Acenaphthenequinone [Cu(AN-Me)_2_]

Two equivalents of compound **AN-Me** (0.054 g, 0.200 mmol) and one equivalent of copper acetate (0.018 g, 0.100 mmol) were suspended in 20 mL of MeOH and stirred for 3 h at room temperature. The majority of the solvent was removed using the rotavapor, then, to the resulting slurry, diethyl ether was added dropwise until a precipitate formed, which was filtered and washed with diethyl ether, then dried under reduced pressure. The final product was obtained as a dark-brown solid, which was investigated by EPR.

Mass spectrum: ESI-MS calc. for C_28_H_20_N_6_O_2_S_2_Cu [M + H]^+^: 600.0458; found: 600.0492.

### Synthesis of Copper Mono(4-Ethyl-3-thiosemicarbazone) Acenaphthenequinone [Cu(**AN-Et**)_2_]

A microwave vial was filled with the ligand **AN-Et** (0.0565, 0.20 mMol), anydrous copper acetate (0.036 g, 0.20 mMol) and 10 mL of EtOH. Immediatelly after the addition of EtOH, the reaction mixture turned red-brown and a dark brown precipitate started to form after ca. 2. minutes under mild sonication. Then the sample was subjected to microwave irradiation (according to the general procedure) at 90 °C for 1 h. The slurry was left to cool down to room temperature filtered under air, washed with Et_2_O and dried under reducced pressure. The desired product, denoted Cu(**AN-Et**)_2_ was obtained in quantitative yield. (Note that in an alternative methods, the small amount of brown precipitate formed within 2–3 min of the reaction was isolated, washed with Et_2_O and analyzed without proceeding with microwave irradiation. Mass spectrometry for both reaction protocols showed near-identical spectra).

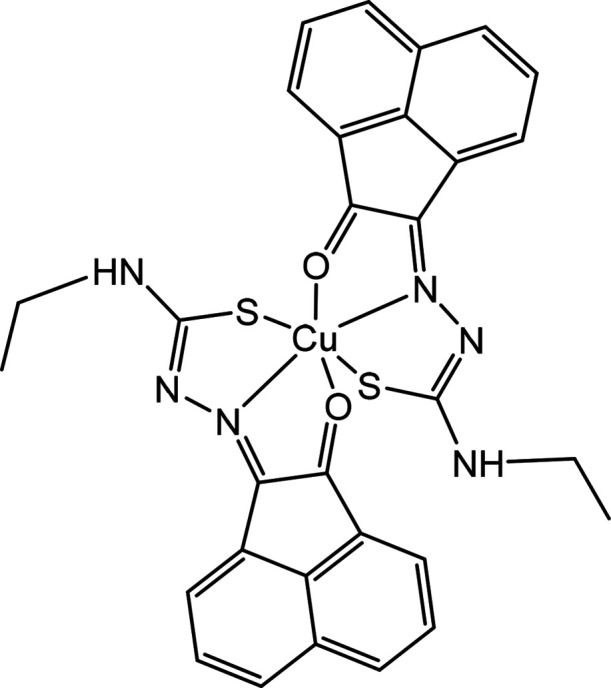



Mass spectrum: ESI-MS calc. for C_30_H_24_N_6_O_2_S_2_Cu [M + H]^+^: 628.0771; found: 628.0809.

### Alternative Method for the Synthesis of Copper Mono(4-Ethyl-3-thiosemicarbazone) Acenaphthenequinone [Cu(**AN-Et**)_2_]

One equivalent of compound ****AN-Et**** (0.028 g, 0.100 mmol) and one equivalent of copper acetate (0.018 g, 0.10 mmol) were suspended in 20 mL of MeOH and stirred for 3 h at room temperature. The solvent was concentrated using the rotavapor, then dropwise added diethyl ether until a precipitate formed, which was filtered and washed with diethyl ether, then dried under reduced pressure. The desired product, denoted Cu(**AN-Et**)_2_ was obtained in quantitative yield as a dark-red solid, which was investigated by EPR.

Mass spectrum: ESI-MS calc. for C_30_H_24_N_6_O_2_S_2_Cu [M + H]^+^: 628.0771; found: 628.0807.

### Alternative Procedure for the Synthesis of Copper Mono(4-Ethyl-3-thiosemicarbazone) acenaphthenequinone [Cu(**AN-Et**)_2_]

Two equivalents of compound **AN-Ethyl** (0.057 g, 0.200 mmol) and one equivalent of copper acetate (0.018 g, 0.100 mmol) were suspended in 20 mL of MeOH and stirred for 3 h at room temperature. The majority of the solvent was removed using the rotavapor; then diethyl ether was dropwise added until a precipitate formed, which was then filtered, washed with diethyl ether, and dried under reduced pressure. The desired product, denoted Cu(**AN-Et**)_2_, was obtained in quantitative yield as a dark-red solid and was investigated by EPR.

Mass spectrum: ESI-MS calc. for C_30_H_24_N_6_O_2_S_2_Cu [M + H]^+^: 628.0771; found: 628.0806.

### Synthesis of Copper Mono(4-Allyl-3-thiosemicarbazone) Acenaphthenequinone [Cu(**AN-Allyl**)_2_]

One equivalent of compound ****AN-Allyl**** (0.030 g, 0.100 mmol) and one equivalent of copper acetate (0.018 g, 0.100 mmol) were suspended in 20 mL of MeOH and stirred for 3 h at room temperature. The solvent was concentrated using the rotavapor; then diethyl ether was dropwise added until a precipitate formed, which was filtered, washed with diethyl ether, and then dried under vacuum. The final product was obtained as a dark-red solid in quantitative yield.

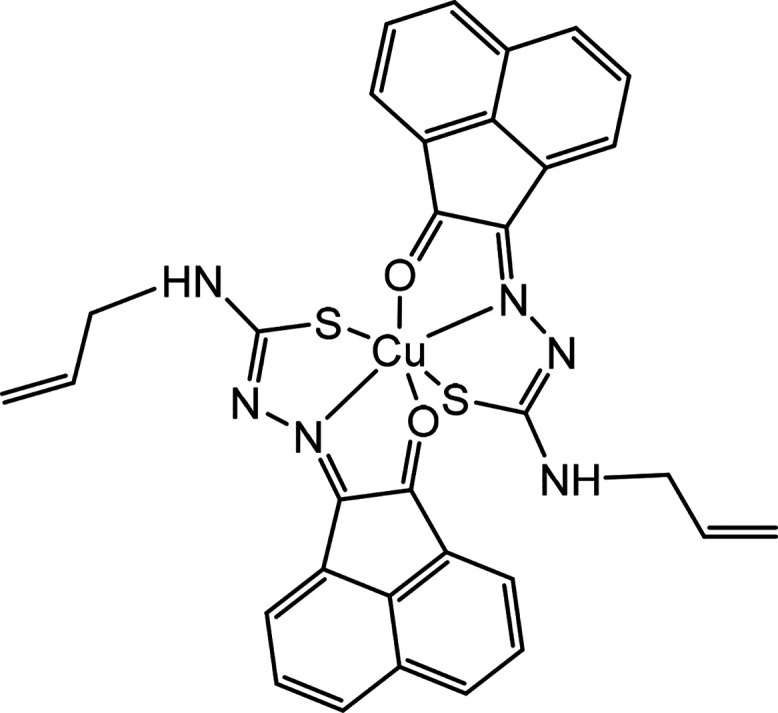



Mass spectrum: ESI-MS calc. for C_32_H_24_N_6_O_2_S_2_Cu [M + H]^+^: 652.0771; found: 652.0736.

### Alternative Procedure for the Synthesis of Copper Mono(4-Allyl-3-thiosemicarbazone) acenaphthenequinone [Cu(**AN-Allyl**)_2_]

Two equivalents of compound **AN-Allyl** (0.059 g, 0.200 mmol) and one equivalent of copper acetate (0.018 g, 0.100 mmol) were suspended in 20 mL of MeOH and stirred for 3 h at room temperature. The majority solvent was removed using the rotavapor; then diethyl ether was dropwise added until a precipitate formed. It was filtered, washed with diethyl ether, and then dried under vacuum. The final crude product was obtained as a dark red color solid in quantitative yield.

Mass spectrum: ESI-MS calc. for C_32_H_24_N_6_O_2_S_2_Cu [M + H]^+^: 652.0771; found: 652.0712.

### Synthesis of Copper Mono(4-Phenyl-3-thiosemicarbazone) Acenaphthenequinone [Cu(**AN-Ph**)_2_]

A microwave vial was filled with the ligand **AN-Ph** (0.050, 0.15 mMol), anydrous copper acetate (0.03g, 0.15 mMol), and 10 mL of EtOH. Immediately after the addition of EtOH, the reaction mixture turned red-brown and a dark brown precipitate started to form after ca. 2 minutes under mild sonication. Then, the sample was subjected to microwave irradiation (according to the general procedure) at 90 °C for 1 h. The slurry was left to cool down to room temperature filtered under air, washed with Et_2_O, and dried under reduced pressure. (Note: For the reaction carried out in the 2:1 Ligand:Cu(II) ratio, 0.10 g (0.30 mMol) of **AN-Ph** ligand and anhydrous Cu(OAc)_2_ (0.03 g, 0.15 mMol) together with 10 mL of EtOH were used, and the reaction under microwave-assisted irradiation conditions proceeded according to the general protocol given above. The desired product, denoted Cu(**AN-Ph**)_2_ was obtained in quantitative yield). Mass spectrometry for the products from both reactions gave rise to almost identical spectra.

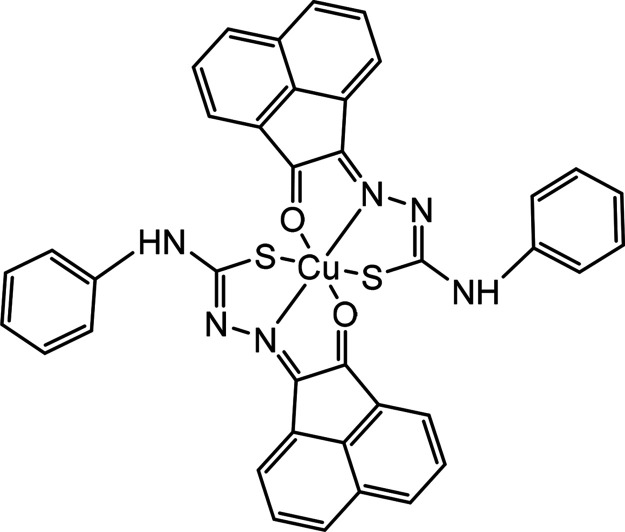



Mass spectrum: ESI^+^-MS calc. for C_38_H_25_CuN_6_O_2_S_2_ [M + H]^+^: 724.0771; found: 724.0765.

### Alternative Procedure for the Synthesis of Copper Mono(4-Phenyl-3-thiosemicarbazone) Acenaphthenequinone [Cu(**AN-Ph**)_2_]

One equivalent of compound ****AN-Ph**enyl** (0.033 g, 0.100 mmol) and one equivalent of copper acetate (0.018 g, 0.100 mmol) were suspended in 20 mL of MeOH and stirred for 3 h at room temperature. The majority of the solvent was removed using the rotavapor; then diethyl ether was dropwise added until a precipitate formed, which was filtered, washed with diethyl ether, and then dried under vacuum. The final product was obtained as a dark red color solid, which was investigated by EPR.

Mass spectrum: ESI-MS calc. for C_38_H_24_CuN_6_O_2_S_2_ [M + H]^+^: 724.0771; found: 724.0756.

### Alternative Procedure for the Synthesis of Copper Mono(4-Phenyl-3-thiosemicarbazone) Acenaphthenequinone [Cu(**AN-Ph**)_2_]

Two equivalent of compound **AN-Phenyl** (0.066 g, 0.200 mmol) and one equivalent of copper acetate (0.018 g, 0.100 mmol) were suspended in 20 mL of MeOH and stirred for 3 h at room temperature. The majority solvent was removed using the rotavapor; then, diethyl ether was dropwise added until a precipitate formed, which was then filtered, washed with diethyl ether, and then dried under vacuum. The final product was obtained as a dark red solid, which was investigated by EPR.

Mass spectrum: ESI-MS calc. for C_38_H_24_CuN_6_O_2_S_2_ [M + H]^+^: 724.0771; found: 724.0762.

### Experimental Methods for EPR Measurements

All solvents were of analytic grade and were purchased from Sigma-Aldrich (Dorset, UK). All EPR samples were prepared with either neat DMSO (5 mM; final concentration of Cu(II) complex) or 7:1 abs. ethanol: DMSO solvent mixture (625 μM; final concentration of Cu(II) complex) in an aerobic condition. Samples containing ∼5 mM/625 μM of Cu(II) complexes and polycrystalline powders of samples were transferred into 4 mm outer diameter/3 mm inner diameter Suprasil quartz EPR tubes (Wilmad LabGlass) and frozen in liquid N_2_. All EPR samples were measured on a Bruker EMXplus EPR spectrometer equipped with a Bruker ER 4112SHQ X-band resonator. Sample cooling was achieved using a Bruker Stinger cryogen free system mated to an Oxford Instruments ESR900 cryostat, temperature control was maintained using an Oxford Instruments MercuryITC.^38−40^ The optimum conditions used for recording the spectra are given below: microwave power 30 dB (0.219 mW), modulation amplitude 5 G, time constant 82 ms, conversion time 16.67 ms, sweep time 60 s, receiver gain 30 dB, and an average microwave frequency of 9.368 GHz. All EPR spectra were measured as frozen solutions at 20 K, respectively. The analysis of the continuous wave EPR spectra and simulations were performed using EasySpin toolbox (5.2.35) for the Matlab (MATLAB_R2022a) program package.^35^

### Computational Details

All calculations were carried out using density functional theory (DFT) as implemented in the Gaussian 09 package.^42^ A variety of exchange correlation functionals and basis sets were used (see ESI for structural parameters together with available experimental details). The optimum exchange correlation functional and the basis set found for ligands incorporating only C, N, O, S, and H were Perdew–Burke–Ernzerhof (PBE) and 6-31G**. For the compounds incorporating Cu and Zn centers, we used PBE exchange correlation functional and aug-cc-PVTZ-pp basis set as implemented in this code. This combination resembled well the experimental structures from X-ray diffraction studies and the Mulliken analysis^43^ was used to estimate the charges on the atoms in ligands and metal complexes well. Supporting Information contains main structural parameters of the metal complexes modelled with a range of different functionals and basis sets. The final structures were optimized using PBE exchange correlation functional. For Cu and Zn, aug-cc-PVTZ-pp basis sets were used. For ligands consisting of C, N, O, S, and H, 6-31G** basis sets were used, and corresponding.xyz files are also provided as Supporting Information.

### In Vitro Assays

The human prostate cancer cells (PC-3) and the human cervical cancer cells (HeLa) were purchased from American Type Culture Collection (ATCC). Cell culture was performed in Eagle’s Minimum Essential Medium (EMEM) for HeLa, RPMI-1640 medium for PC-3. The media contained fetal calf serum (FCS) (10% for HeLa and PC-3, and 15% for FEK-4), 0.5% penicillin/streptomycin (10,000 IU mL^–1^/10,000 mg mL^–1^), and 200 mM L-glutamine (5 mL). All steps were performed in absence of phenol red. Cells were cultured at 37 °C in 5% CO_2_ incubator in T75 flasks until 60–70% confluency and passaged by trypsinization. Cells were then counted using a hemocytometer and then seeded as appropriate for the necessary assays, as follows:

### Cellular Imaging

Experiments were carried out in the PC-3 cell line. Cells were cultured as above, then seeded in 35 mm glass bottom Petri-dishes at a density of 2 × 10^5^ cells/dish and cultured at least 48 h prior to the recording of control data in untreated cells, and cells incubated with the compounds: Zn(**AN-Allyl**)_2_, Zn(**AA-Allyl**)_2_, Zn(**PH-Allyl**)_2_ and Zn(**PY-Allyl**)_2_, at a final concentration of 100 μM (1% DMSO and 99% conditioned media) incubated for 20 min. The media was replaced by a phenol free serum-free medium before the image capturing.

#### MTT Assays in PC-3 and HeLa

Cytotoxic activity tests were carried out according to our established protocols and further details are given in Supporting Information. Cells were seeded in 96-well plates at a density of 7 × 10^3^ cells/well and cultured for 48 h to adhere fully. Then, cells were treated with AN free ligands, their Zn(II) derivatives, and *cis*-[PtCl_2_(NH_3_)_2_] at final concentrations (1% DMSO and 99% conditioned media) of 250 μM, 100 μM, 50 μM, 10 μM, 1 μM, 0.5 μM, 0.1 μM, and 0.001 μM in conditioned media for 48 h.

#### Crystal Structure Determination by X-ray Diffraction

Single crystal analysis of a range of compounds was performed by X-ray crystallography. The growth of the crystals suitable for measurements was performed generally by dissolving the compound of interest in the minimum amount of THF in a small glass vial and then placing this inside a larger vial. A small amount of pentane was placed in the larger vial, and the system was sealed from the outside atmosphere. This was then kept in a still place, allowing the crystals to grow slowly over the subsequent weeks. In alternative methods, the compound of choice was dissolved in the minimum amount of THF in a vial and the pentane was layered on top (THF: pentane ratio 1:3). Additionally, crystals suitable for X-ray diffraction for either the Zn(II) complexes or free ligands appeared over time, slowly, and over several weeks from concentrated solutions of DMSO or d^6^-DMSO in NMR tubes. Crystals were selected using the oil drop technique, in perfluoropolyether oil and mounted at 150(2) K with an Oxford Cryostream N2 open-flow cooling device. Intensity data were collected on a Nonius Kappa CCD single crystal diffractometer using graphite monochromated Mo–Kα radiation (λ = 0.71073 Å), whereby data were processed using the Nonius Software, or at Diamond using Synchrotron radiation (λ = 0.68890 Å) on a CrystalLogic Kappa (3 circle), Rigaku Saturn724 at 150 K, whereby data were processed using the Rikagu software package (CrystalClear-SM Expert 2.0 r5).

Alternative data collection was at 150(2) K on a Rigaku Xcalibur, EosS2 single crystal diffractometer using graphite monochromated Mo–Kα radiation (λ = 0.71073 Å), or on a Rigaku SuperNova Dual EosS2 single crystal diffractometer using monochromated Cu–Kα radiation (λ = 1.54184 Å), in which case the unit cell determination, data collection data reduction and absorption correction were performed using the CrysAlisPro software. For all structures a symmetry-related (multiscan) absorption correction had been applied. The structures were solved by direct methods using the programmes SIR97, SHELXS or SHELXTL followed by full-matrix least-squares refinement on F^2^ using SHELXL-2018/1-3 implemented in the WINGX-1.80 suite of programmes throughout or using the SHELXle platform. Additional programmes used for analyzing and graphically handling data included: PLATON, and ORTEP3 for Windows and Mercury.^44−53^

All non-hydrogen atoms were refined anisotropically, and the hydrogen atoms were placed onto calculated positions and refined isotropically only, using a riding model. Wherever possible heteroatom bound hydrogen atoms were first located in the difference Fourier map and were refined freely or with bond length restraints. All available crystallographic data were deposited to CCDC, and these structures were also uploaded as cif files. Deposition numbers and corresponding compound labels are as follows: CCDC 2218631: C_24_H_30_N_4_O_3_S – **AN-12**; CCDC 2218629: (C_20_H_22_N_4_O_4_) H_2_O – urea derivative byproduct (H_2_O adduct, isolated crystal/traces from synthesis of **AN-10**); CCDC 2218628: C_18_H_13_N_3_OS – **AA-Me**; CCDC 2218626: C_20_H_15_N_3_OS – **AA-Allyl**; CCDC 2218624: C_23_H_15_N_3_OS, C_2_H_3_N **AA-Ph** (CH_3_CN adduct); CCDC 2218623: C_18_H_15_N_3_OS – **PH-Allyl**; CCDC 2218622: C_17_N_15_N_3_OS – **PH-Ethyl**; CCDC 2218769: C_21_H_15_N_3_OS – **PH-Ph**; CCDC 2218621: C_23_H_15_N_3_OS – **PY-Ph**; CCDC 2218620: C_23_H_15_N_3_OS, C_2_H_6_OS **PY-Ph** (DMSO adduct); CCDC 2218619: C_20_H_15_N_3_OS – **PY-Allyl**; CCDC 2218617: C_19_H_15_N_3_OS – **PY-Et**; CCDC 2218616: C_18_H_13_N_3_OS – ****PY-Me****; CCDC 2218615: C_38_H_28_N_6_O_2_S_2_Zn, 2(C_2_H_6_OS) **Zn(PY-Ethyl)**_**2**_ (2 × DMSO adduct); CCDC 2218614: C_34_H_28_N_6_O_2_S_2_Zn, 2(C_4_H_8_O) **Zn(PH-Ethyl)**_**2**_ (2 × THF adduct); CCDC 2218613: C_36_H_28_N_6_O_2_S_2_Zn, 2(C_4_H_8_O) **Zn(PH-Allyl)**_**2**_ (2 × THF adduct); CCDC 2218612: 2(C_26_H_16_N_6_O_2_S_2_Zn), 2(C_26_H_16_N_6_O_2_S_2_Zn) – **Zn(AN-H)**_**2**_ (cocrystallized with a large number of disordered DMSO molecules).

### General Radiochemistry Procedures and ^64^Cu(II) Radiolabeling

Cyclotron-available ^64^Cu was produced according to established protocols^55^ from the proton irradiation of ^64^Ni according to the ^64^Ni(p,n)^64^Cu nuclear reaction, in a 16.5 MeV PETtrace cyclotron. The ^64^Cu^2+^_(aq)_ was then extracted from the ^64^Ni target and purified from ^64^Ni^2+^_(aq)_ using an ion exchange column, and formulated as an aqueous ^64^CuCl_2_ solution in 0.1 mol dm^–3^ HCl and supplied from the Wolfson Brain Imaging Centre, Cambridge in batches of ca. 100 MBq samples. This was then delivered and used at the Oxford Siemens Molecular Imaging Laboratories, where radiolabeling was carried out using established protocols. The ^64^Cu(II) species for these reactions was formulated in 0.1 N hydrochloric acid (0.2 mL). To this, 0.1 M sodium acetate (pH 5.5, 1.8 mL) was added to yield the stock solution of ^64^Cu(OAc)_2_. The activity of the stock solution was measured as ca. 90–100 MBq in 2 mL of aqueous solutions. ^64^Cu complexes were formed by the *in situ* deprotonation of the neutral pro-ligand, followed by metalation. Each radio reaction was generally carried out in 4–10 MBq activity levels. A stock solution of the free monothiosemicarbazone was prepared as either 1 or 2 mg/mL in DMSO, MeOH, or EtOH. Then, in the optimized procedures, standard solutions of ligand were prepared as 1 mg/mL in DMSO or 1 mg/mL in MeOH. 50 μL of stock solution were diluted with 400 μL of water and 50–100 μL of ^64^Cu(OAc)_2_ stock added. All manipulations of radioactive material were performed in a dedicated fume cupboard behind a lead screen. The radiolabeling reactions were stirred at room temperature for between 15 and 40 min, or heated for 90 min at temperatures at least 20 deg. below the boiling points of the solvent employed. From the reaction mixtures, 25 μL aliquots were taken for radioHPLC analysis. RadioHPLC was performed either on an Agilent 1100 series HPLC system (Agilent Technologies, Stockport, UK) equipped with a γ-RAM Model 3 gamma-detector (IN/US Systems Inc., Florida, USA) and a Laura 3 software (LabLogic, Sheffield, UK) equipped with a 250 mm × 4.6 mm Phenomenex Primesphere 5 C-18-HC 110H column. UV and radiodetection were used in series with a delay time of approximately 10 s. Generally min. 25 minute gradient methods were employed using acetonitrile/water as mobile phase, as follows, and extensive method development was performed to optimize peaks separation. The gradient elution was 0.1% TFA in milli-Q water as solvent A and 0.1% TFA in MeCN as solvent B. A reverse gradient was applied starting with A at 95% for 2 min, going up to 5% A at 12 min, isocratic level until 14 min and gradient until 95% A at 16 min, then hold to 25 min (Method C). Alternativelly, characterization was carried out on an Agilent 1100 series HPLC system (Agilent Technologies, Stockport, UK) equipped with a γ-RAM Model 3 gamma-detector (IN/US Systems Inc., Florida, USA) and a Laura 3 software (LabLogic, Sheffield, UK). The gradient elution was 0.1% TFA in milli-Q water as solvent A and 0.1% TFA in MeCN as solvent B. A reverse gradient was applied starting with A at 95% for 2 min, going up to 5% A at 12 min, isocratic level until 14 min and gradient until 95% A at 16 min, then hold to 25 min (Method D).

In most cases, the radiotraces obtained after purification indicated the presence of two new major copper-64 species and that of one other minor species, with Rt generally ranging between 10 and 17 min . The analysis of peak integrals indicated that the overall radio-incorporation yield was generally high for all ligands featuring the AN backbone investigated hereby, and, for these, virtually no traces of the unbound 64-Copper were found in the expected region (Rt ca. 2.5 min).

## Data Availability

The data that supports the findings of this study are available in the supplementary material of this article or from the authors.

## Supporting Information Available

X-ray CIF files (ZIP)
DFT data: xyz files for the optimized geometries (ZIP)
General experimental, and alternative synthesis methods, detailed NMR and Mass spectrometry, HLPC traces, UV–vis and Fluorescence spectrsocpy data, additional radiochemistry and cellular assays and DFT data (PDF)
