# Supplementary material for: Structural and Functional Diversity in Rigid Thiosemicarbazones with Extended Aromatic Frameworks: Microwave-Assisted Synthesis and Structural Investigations
Source: ACS Omega. 2023 Apr 25;8(18):16047–79. doi: 10.1021/acsomega.2c08157 (PMC10173449; doi:10.1021/acsomega.2c08157)
Supplement: Supplementary file 3 — ao2c08157_si_003.pdf [file ao2c08157_si_003.pdf]

Supplementary Information

Structural and Functional Diversity in Rigid Thiosemicarbazones with Extended Aromatic Frameworks: Microwave-Assisted Synthesis and Structural Investigations

Fernando Cortezon-Tamarit[1=#], Kexin Song[1=], Navaratnarajah Kuganathan[2], Rory L. Arrowsmith[1], Sara Raquel Mota Merelo de Aguiar[1], Philip A. Waghorn[3], Adam Brookfield, [4] Muralidharan Shanmugam, [4] David Collison, [4] Haobo Ge[1,5], Gabriele Kociok-Köhn[1], Charareh Pourzand[5,6], Jonathan Robin Dilworth[1,3] and Sofia Ioana Pascu[1,3,6]*

[1]Department of Chemistry, University of Bath, Claverton Down, Bath, BA2 7AY, United Kingdom
[2]Department of Materials, Imperial College London, Royal School of Mines, Exhibition Road, London SW7 2AZ, UK
[3]Department of Chemistry, Chemistry Research Laboratory, University of Oxford, Mansfield Road, Oxford, OX1 3TA, United Kingdom
[4]Department of Chemistry, and Photon Science Institute, The University of Manchester, Oxford Road, Manchester M13 9PL, United Kingdom
[5] Department of Life Sciences, University of Bath, Bath BA2 7AY, UK
[6] Centre of Therapeutic Innovation, University of Bath, Bath BA2 7AY, UK

Keywords: synthetic and coordination chemistry, X-ray crystallography, aromatic thiosemicarbazones, cellular fluorescence imaging, ^{64}Cu radiochemistry

= F. C-T and KS contributed equally to the work.
#Current Address: Radiobiology Research Institute, University of Oxford, Oxford OX3 7LE, UK

Table of contents:

1 Synthetic procedures.. S2

2 Selected HPLC traces S32

3 Optical spectroscopy of selected thiosemicarbazones S41

4 NMR spectroscopy of selected compounds.. S51

5 Mass spectrometry for representative ligands and complexes.. S61

6 Infrared spectroscopy for selected compounds.. S81

7 EPR and Magnetic Moment Measurements for selected Cu(II) compounds S89

8 Radiochemistry assays... S91

9 MTT Assays.. S104

10 Selected Structural Parameters and DFT Calculations………………..…………… S153

11 X-Ray Crystallography: CCDC Numbers S110

12 References.. S154

1 Synthetic procedures

General Methods

All chemicals and solvents were reagent grade and used as received unless otherwise specified. The free ligands are known AN-Me, AN-Et, AN-Allyl and AN-Ph, AN-en (or AN-10) and AN-en^{t}Boc (or AN-11) and were synthesized hereby according to the multistep protocols adapted and optimized with respect to published synthetic method [1-6] and these adapted methods are again given briefly below.

The microwave synthesizer used was the Biotage Initiator, Max power 400W, error in temperature ± 5°C. Elemental analyses were performed either by the microanalysis service at the Inorganic Chemistry Department, University of Oxford, or at the London Metropolitan University. Electronic absorption spectroscopy (UV/Vis) was performed using a Perkin-Elmer Lambda 19 spectrometer, running UV Winlab software. Spectra were measured using 1.00 cm quartz cuvettes.

Fluorescence spectra were recorded in 1.00 cm quartz cuvettes using a Hitachi F-4500 fluorescence spectrometer, running FL Solutions software.

Relative quantum yields were determined by comparison to either Fluorescein in 0.1 M NaOH (ΦR = 0.95 at 496nm) or $[Ru(bipy)_3](PF_6)_2$ in water (ΦR = 0.042 in water at 420 nm), using the following formula:

$$\Phi_S = \Phi_R \cdot (D_S/D_R) \cdot (A_R/A_S) \cdot (I_R/I_S) \cdot (\eta_S/\eta_R)^2$$

where Φ is the relative quantum yield, D is the integrated area of the fluorescence emission peak, A is the absorption of the solutions at the excitation wavelength, I is the flux at the excitation wavelength used and η is the solution refractive index. R and S subscripts refer to the reference and sample respectively

A range of different HPLC methods were applied, as follows.

Method A was carried out using a Symmetry® C-18 column (4.6 x 260 mm) with UV/visible detection measured at λ_{obs} = 200 nm, 300 nm, 400 nm, 450 nm, 500 nm, 600 nm, 700 nm and 800 nm. The gradient elution was 1.1 mL/minute, with 0.1% TFA MilliQ water as solvent A and 0.1% TFA MeCN as solvent B. Start 95 % A reverse gradient until 5% A at 7.5 minutes, isocratic until 15 minutes, reverse gradient until 17.5 minutes 95% A, then hold to 18 minutes. A variant of this HPLC Method (**Method A-1**) was performed using a Waters C-18 column (4.6 x 250 mm) with UV/Vis detection at λobs = 254nm and 410 nm with a 0.9 mL/min gradient elution method (Solvent A: THF with 0.1 % TFA v/v, Solvent B: water with 0.1% TFA v/v): start 5 % A, gradient over 12 min reaching 95 % A, hold to 15 min at 95 % A, reverse gradient till 18 min reaching 5 % A, then hold to 20 min at 5 % A.

Additional HPLC methods used:

Method B was carried out using a Symmetry® C-18 column (4.6 x 260 mm) with UV/visible detection measured at eight wavelengths from λ_{obs} = 200 nm, 220 nm, 280 nm, 300 nm, 400 nm, 450 nm, 500 nm, 600 nm, 700 nm and 800 nm. The gradient elution was 0.8 mL/minute, with 0.1% TFA MilliQ water as solvent A and 0.1% TFA MeCN as solvent B. Start 95 % A reverse gradient until 5% A at 7.5 minutes, isocratic until 15 minutes, reverse gradient until 17.5 minutes 95% A, then hold to 18 minutes.

Method C was carried out using a Acclaim® 120 C-18 column (4.6 x 150 mm) with UV/visible detection measured at up to four λ_{obs} = 214 nm, 220 nm, 254 nm, 280 nm, 300 nm and 400 nm. The gradient elution was 1.0 mL/minute, with 0.1% TFA MilliQ water as solvent A and 0.1% TFA

MeCN as solvent B. Start 95 % A reverse gradient until 5% A at 7.5 minutes, isocratic until 15 minutes, reverse gradient from 15.1 minutes 95% A, then hold to 18 minutes.

Method D was carried out using a Waters C-18 column (4.6 x 250 mm) with UV/visible detection measured at up to four λ_{obs} = 254 nm. The gradient elution was 1.0 mL/minute, with 0.1% TFA MilliQ water as solvent A and 0.1% TFA MeCN as solvent B. Start 95 % A reverse gradient until 5% A at 12 minutes, isocratic until 15 minutes, reverse gradient from 15.1 minutes 95% A, then hold until 21 minutes.

Method E was carried out using a Acclaim® 120 C-18 column (4.6 x 150 mm) with UV/visible detection measured at up to four λ_{obs} = 214 nm, 220 nm, 254 nm, 280 nm, 300 nm and 400 nm. The gradient elution was 1.0 mL/minute, with 0.1% TFA MilliQ water as solvent A and 0.1% TFA MeCN as solvent B. Start 95 % A reverse gradient until 5% A at 10 minutes, isocratic until 15 minutes, reverse gradient from 15.1 minutes 95% A, then hold to 18 minutes.

Method F was carried out using a Waters C-18 column (4.6 x 250 mm) with UV/visible detection measured at λ_{obs} = 254 nm. The gradient elution was 1.0 mL/minute, with 0.1% TFA MilliQ water as solvent A and 0.1% TFA MeCN as solvent B. Start 95 % A, reverse gradient until 5% A at 10 minutes, isocratic until 12 minutes, reverse gradient until 95% A at 14 minutes, then hold to 15 minutes at 95% A.

Method G was carried out using a Waters C-18 column (4.6 x 250 mm) with UV/visible detection measured at up to four λ_{obs} = 214 nm, 220 nm, 254 nm, 280 nm, 300 nm and 400 nm. The gradient elution was 1.0 mL/minute, with 0.1% TFA MilliQ water as solvent A and 0.1% TFA MeCN as solvent B. Start 95 % A reverse gradient until 5% A at 15 minutes, isocratic until 22.5 minutes, reverse gradient from 22.6 minutes 95% A, then hold to 25.5 minutes.

Method H was carried out using an Eclipse C-18 column (2 x 50 mm) with UV/visible detection measured at up to four λ_{obs} = 214 nm, 220 nm, 254 nm, 280 nm, 300 nm and 400 nm. The gradient elution was 1.0 mL/minute, with 0.1% TFA MilliQ water as solvent A and 0.1% TFA MeOH as solvent B. Start 95 % A reverse gradient until 5% A at 8 minutes, hold until 10 minutes.

Method I was carried out using a Phenomenex Ultracarb C-18 column (4.6 x 150 mm) with UV/visible detection measured at up to four λ_{obs} = 254 nm. The gradient elution was 1.0 mL/minute, with 0.1% TFA MilliQ water as solvent A and 0.1% TFA MeOH as solvent B. Start 95 % A reverse gradient until 5% A at 12 minutes, hold until 15 minutes.

Semi-Preparative HPLC was carried out using a Phenomenex® Gemini C-18 column (10 x 250 mm) with UV/visible detection measured at up to four λ_{obs} = 214 nm, 220 nm, 254 nm, 280 nm, 300 nm and 400 nm. The gradient elution was 1.0 mL/minute, with 0.1% TFA MilliQ water as solvent A and 0.1% TFA MeCN as solvent B. Start 95 % A reverse gradient until 5% A at 15 minutes, isocratic until 22.5 minutes, reverse gradient from 22.6 minutes 95% A, then hold to 25.5 minutes.

Synthesis of selected ligands, metal complexes and standard precursors by conventional heating

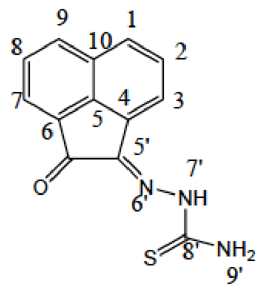

1. **Preparation of mono(thiosemicarbazone) acenaphthenequinone**

The ligand was obtained as reported in the literature from a 1:1 molar ratio of acenaphthenequinone and thiosemicarbazide in absolute ethanol. Acenaphtenequinone (0.500 g, 2.74 mmol) and thiosemicarbazide (0.250 g, 2.74 mmol) are suspended in absolute ethanol (15 ml) and heated under reflux for 2 hours. The solid was isolated by filtration whilst hot. Then, the solid was re-suspended in hot methanol (10 ml) and stirred for 15 minutes before filtering and further washing with methanol. The solid was then dried under reduced pressure. Yield 0.564 g, 2.21 mmol, 81 %.

^{1}H NMR (300 MHz, d_6 –DMSO, 25°C): δ 12.50 (s, 1H, H7'), 9.12 (s, 1H, NH_2), 8.83 (s, 1H, NH_2), 8.34 (d, 1H, H7), 8.11 (d,1H, H3), 8.09 (d, 1H, H9), 7.99 (d, 1H, H1), 7.86(dd , 1H, H8), 7.81(dd, 1H, H2).

^{13}C{^{1}H} NMR (300 MHz, d_6 –DMSO, 25°C): δ 189.0(C=O), 179.3 (C-8'), 139.6(C-5'), 137.8(C-5), 133.2(C-3), 130.8(C-4), 130.4(C-10), 130.3(C-8), 129.3(C-2), 129.0(C-6), 127.5(C-9), 122.8(C-7), 118.8(C-4). **IR**: 3404 m υ(NH_2), 3241 w υ(NH_2), 3147 m υ(N-H), 1689 s υ(C=O), 1606 s υ(C=N), 1575 m υ(C=N), 1486 sb, 1451 s υ(ring), 1140 m υ(C-S).

ESI MS+: Calc for $C_{13}H_{10}N_3OS$ $[M+H]^+$: 256.0545(100%); Found M/z 257.1(100%) $[M + H^+]$.

HPLC: R_f = 14.1 mins (Method A-1)

2. Preparation of mono (4-ally-3-thiosemicarbazone) 9,10 phenanthrenequinone (PH-Allyl)

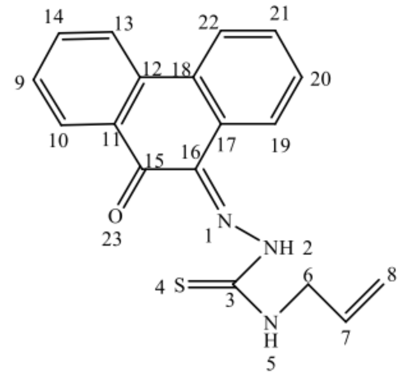

The thiosemicarbazone 4-allyl-3-thiosemicarbazone (0.073 g, 0.574 mmol) was reacted with 9,10-phenanthrenequinone (0.100 g, 0.480 mmol) in a 1.2:1 ratio respectively. About 20 ml of ethanol was added and the reaction refluxed for 4 hours at a temperature of 100°C. Once the temperature had been reached, a few drops of glacial acetic acid was added. The evaporation of the solvent left a yellow powder which was washed and filtered with diethyl ether to remove impurities. Yield 0.065 g, 0.20 mmol, 42%

^{1}H NMR (300MHz, d^6-DMSO, 25°C): δ14.01 (s, 1H, H2), 8.40 (d, 1H, H19), 8.39 (d, 1H, H10), 8. 24 (d, 1H, H13), 8.239 (d, 1H, H22), 7.86 (t, 1H, H9), 7.85 (t, 1H, H20), 7.57 (t, 1H, H14), 7.55 (t, 1H, H21), 6.88 (t, 1H, H5), 6.0 (m, 1H, H12), 5.45 (dd, 1H, H6), 4.3 (m, 1H, H8(a)), 3.95 (m, 1H, H8(b)).

ES MS$^+$ Calc for $C_{18}H_{16}N_3OS$ [M+H]$^+$: 322.1014 (100%); found M/z = 322.1 (100%).

HPLC (Method A'): Rf = 15.9 mins (reverse phase, C18, 20 min gradient, Method A, 0.1% v/v TFA); >95% purity.

3. Preparation of mono(4-ethyl-3-thiosemicarbazone) 9,10-phenanthrenequinone (PH-Ethyl)

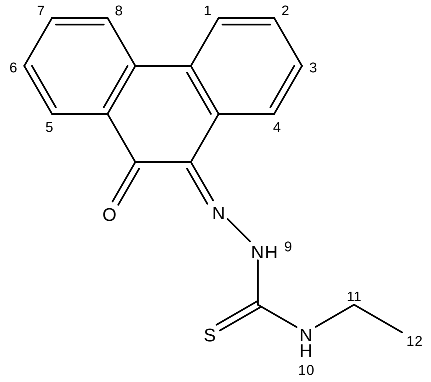

To a mixture of 4-ethyl-3-thiosemicarbazide (0.810 g, 6.84 mmol) and 9,10-phenanthrenequinone (0.470 g, 2.28 mmol) 10 mL of ethanol were and the resulted slurry was stirred gently. Then, one drop of sulphuric acid was and the mixture was heated under reflux for 24 hours. The reaction mixture was then filtered, the resulted mixture washed with ethanol and diethyl ether. Yield 0.612g, 1.97 mmol, 86.8%.

^{1}H NMR (300 MHz, d_6–DMSO, 25 °C): δ 9.65 (m, 1H, H10), 8.65 (d, 1H, H5), 8.42 (d,1H, H4), 8.33 (d, 1H, H8), 8.26 (d, 1H, H1), 7.85 (t, 1H, H6), 7.59 (t, 1H, H3), 7.51 (m, 1H, H7), 7.51 (m, 1H, H2), 3.73 (m, 2H, H11), 1.25 (t, 3H, H12).

ES MS$^+$: Calculated for $C_{17}H_{16}N_3OS$ [M + H]+: 310.1014; Found M/z = 310.1 (25%) [M + H]+.

HPLC: Rf =18.1 mins (Method A-1).

Elemental analysis: % Found (Calculated) C 65.22 (66.00), H 4.86 (4.89), N 13.80 (13.58), S 10.37 (10.36).

4. Preparation of mono(4-methyl-3-thiosemicarbazone) aceanthrenequinone (AA-Me ligand)

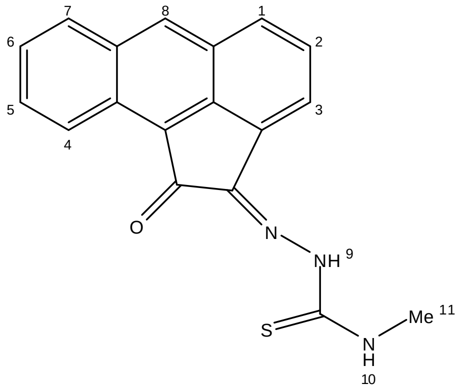

Aceanthrenequinone (0.250 g, 1.08 mmol) and $Zn(OAc)_2 \cdot 2H_2O$ was suspended in glacial acetic acid (4.5 mL) and heated to 110 °C. To this 4-methyl-3-thiosemicarbazide (1.130 g, 10.8 mmol) and a catalytic amount of TFA (0.25 mL) was added and the mixture subsequently heated under reflux for 4 hours. The solid was isolated by filtration whilst hot, then re-suspended in warm acetic acid (5 mL) and stirred for 15 minutes. The suspension was filtered again and washed with diethyl ether (50 mL) and then dried under vacuum. Yield 0.215g, 0.67 mmol, 62%

^{1}H NMR (300 MHz, d_6–DMSO, 25°C): δ 12.86 (s, 1H, H9), 9.40 (d, 1H, H4), 9.07 (s, 1H, H8), 8.91 (d, 1H, H3), 8.35 (d, 1H, H7), 8.20 (d, 1H, H1), 7.70-7.96 (b, multiple aromatic resonances, 4H, H2, H5, H6, H10), 3.12 (d, 3H, H11).

ES-MS$^+$ Calculated for $C_{18}H_{14}N_3OS$ $[M + H]^+$: 320.0858; Found M/z = 320.1 (20%)

HPLC: Rf = 18.7 mins (method A-1).

IR: 3415 sbr υ(NH), 1630 m υ(CO), 1600 m υ(CN), 1534 m υ(CN), 1486 ssh υ(ring), 1447 ssh υ(ring), 1130 υ(CS).

Elemental analysis: % Found (Calculated) C 67.63 (67.69), H 4.23 (4.10), N 13.10 (13.16).

5. Preparation of Zinc(II) bis[(mono-thiosemicarbazone) acenaphthenequinone], complex [Zn(AN-H)$_2$]

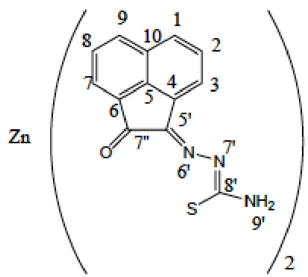

Mono(thiosemicarbazone) acenaphthenequinone (0.350 g, 1.37 mmol) and zinc acetate dihydrate (0.150 g, 0.68 mmol) were suspended in ethanol (50ml) with 10 drops of HCl(35 %) and heated under reflux for 24 hours. The red-orange solid formed was isolated by filtration whilst hot. The suspension was filtered and washed with diethyl ether (50 ml). The resulting solid was then dried under vacuum. Yield = 0.327 g, 0.57 mmol, 84 %.

^{1}H NMR (300 MHz, d_6 –DMSO, 25°C): δ 8.72 (d, 2H, NH_2), 8.63 (d, 1H, H-7), 8.24 (d, 1H, H-3), 8.05 (d,1H, H-9), 7.92 (d, 1H, H-1), 7.79 (t, 1H, H-8), 7.71 (t, 1H, H-2). **^{13}C NMR** (300 MHz, d_6 –DMSO, 25°C): δ 188. 3 (C-7’’), 186.8 (C-8’), 138.2 (C-5’), 138. 2 (C-5), 132.8 (C-3), 130.3 (C-4), 129.8 (C-10), 128.9 (C-8), 128.3 (C-2), 128.2 (C-6), 127.2 (C-9), 124.0 (C-7), 122.9 (C-1).

IR: 3446w 3355w υ(NH_2), 1685s υ(C=O), 1604sm υ(C=N), 1593s υ(C=N), 1486s υ(ring), 1176m υ(C-S), 1118mw υ(N-N).

ESI MS+: Calculated for $C_{26}H_{17}N_6O_2S_2Zn$, $[M + H]^+$: 573.0146 (100%); Found M/z: 573.0 (100%).

6. Preparation of Zinc (II) [mono (4-ethyl-3-thiosemicarbazone) 9,10 phenanthrenequinone], complex [Zn(PH-Ethyl)$_2$]

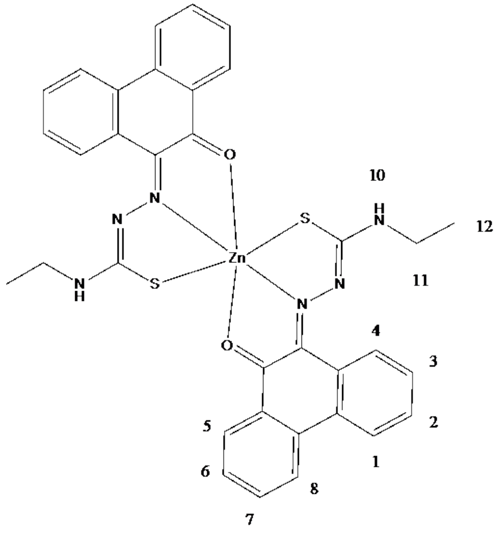

9,10-phenanthrenequinone (0.250g, 1.2 mmol), 4-ethyl-3-thiosemicarbazide (0.310g, 2.64 mmol) and $Zn(OAc)_2$ •$2H_2O$ (0.400 g, 1.82 mmol) were added to methanol (5 mL). One drop of sulphuric acid was then added and the mixture was heated under reflux for 24 hours under an atmosphere of argon. The reaction mixture was then filtered under gravity and washed with ethanol and diethyl ether and subsequently recrystalised from THF. HPLC (Gilson

Unipoint instrument using a reverse phase column with a CH_3CN/H_2O mobile phase, 25 min gradient, adapted method A-1) showed the presence of the free ligand (R_f =18.4 mins) additionally to the desired product [Zn(PH-Ethyl)$_2$] with the corresponding trace at 13.5 mins, which was then further separated by reversed phase semi-prep HPLC.

^{1}H NMR (300 MHz, d8 - THF, 25 ^{o}C): Complex spectrum, NH prótons not assignable. δ: 8.48 (d, 1H, H5), 8.40 (d,1H, H4), 8.37 (d, 1H, H8), 8.30 (d, 1H, H1), 7.80 (t, 1H, H6), 7.55 (t, 1H, H3), 7.51 (m, 1H, H7), 7.42 (m, 1H, H2), 1.24 (m, H11), 0.91 (t, H12).

ES MS$^+$: Calculated for $C_{34}H_{28}N_6O_2S_2Zn$ [M+H+]: 681.1007; found 681.2 (5%).

7. **Preparation of Zinc (II) [mono (4-ally-3-thiosemicarbazone) 9,10 phenanthrenequinone] complex [Zn(PH-Allyl)$_2$]**

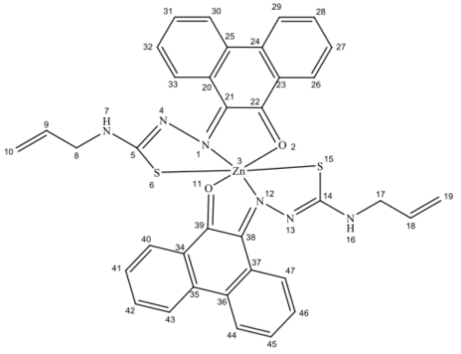

Method 1. The product **[Zn(PH-Allyl)$_2$]** was obtained either from a 1:1 molar ratio of mono (4-ally-3-thiosemicarbazone) 9,10 phenanthrenequinone with Zinc Acetate in THF, or from the 1:2 reaction, described at Method 2 below. For the 1:1 reaction, zinc Acetate (0.007 g, 0.0311 mmol) was added to the compound **1** (0.010 g, 0.0311 mmol) in THF (20 ml). The solution was stirred for 3 hours. A minimum amount of hexane was added and the red solid formed was isolated by filtration. Crystals suitable for X-ray diffraction appeared from the ^{1}H NMR tube and were analyzed by single crystals X-ray diffraction.

^{1}H NMR (300MHz, d^6-DMSO, 25°C) δ 8.85 (s, 1H, H7), 8.40 (d, 1H, H26), 8.39 (d, 1H, H33), 8. 24 (d, 1H, H29), 8.239 (d, 1H, H30), 7.86 (t, 1H, H27), 7.85 (t, 1H, H32), 7.57 (t, 1H, H28), 7.55 (t, 1H, H31), 6.0 (m, 1H, H9), 5.45 (dd, 2H, H8), 4.3 (m, 1H, H19(a)), 3.95 (m, 1H, H19(b)).
ES MS$^+$: Calc. for $C_{36}H_{29}N_6O_2S_2Zn$ $[M+H]^+$: 705.1085; Found M/z:705.11 (65.4%)

HPLC: R_f = 8.2 mins (reverse phase, C18, 25 min gradient, 0.1% v/v TFA, Method A); >80% purity (degradation on HPLC column in presence of TFA)

***Method 2*. Reaction between 9,10 phenanthrenequinone, 4-allyl-3-thiosemicarbazide and Zinc(OAc)$_2$**

9,10 phenanthrenequinone (0.600 g, 2.74 mmol) was suspended in 10ml of glacial acetic acid and heated under reflux in a nitrogen atmosphere to 120° C. 4 allyl-3-thiosemicarbazide (3.800 g, 24.66 mmol) and $Zn(OAc)_2.2H_2O$ (1.900 g, 8.24 mmol) were then added to the suspension. The mixture was heated under reflux for 2 hours and 30 minutes with a deep red solution produced. Diethyl ether was subsequently added and the reaction was stirred for 20 minutes. An orange solid is isolated by filtration and washed with diethyl ether and methanol. The presence of a major, additionally to a minor product (ca 3:1 ratio) was observed by HPLC (Rf: 15 min, 8 min, respectively). These were separated by semi-prep HPLC from the crude mixture and crystals suitable for X-ray analysis were obtained for both components. The major product proved to be the free, unreacted, mono-ligand PH-Allyl. The minor product proved to be the Zn complex [Zn(PH-Allyl)$_2$].

ES MS$^+$: Calc for $C_{18}H_{16}N_3OS$: 322.1014; found M/z 322.1 [free ligand PH-Allyl, M+H]$^+$, and Calc. C36H29N6O2S2Zn: 705.1085; found M/z 705.1 [Zn(PH-Allyl)$_2$+H]$^+$

7. Synthesis of mono (4-allyl-3-thiosemicarbazone) aceanthrenequinone ligand (AA-Alyl)

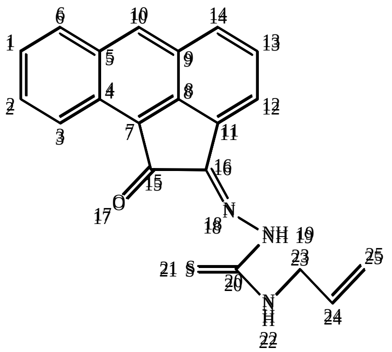

Aceanthrenequinone (0.100 g, 0.43 mmol) and 1.2 equivalents of 4-ally-3-thiosemicarbazone (0.068 g, 0.52 mmol) were mixed together with 40 ml of ethanol and refluxed for 4 hours. Once the reaction temperature reached of 100 ^{o}C, a few drops of glacial acetic acid were added. Then a red-orange solid was obtained after concentration of the solution, filtration, washing with diethyl ether and drying under reduced pressure. Yield 0.107 g, 0.31 mmol, 72%

^{1}H NMR (300MHz, d_6-DMSO, 25°C) δ 12.97 (s, 1H, H19), 9.60 (d, 1H, H3), 9.2 (s, 1H, H22), 8.5 (dd, 1H, H14), 8.4 (dd, 1H, H6), 8.3 (t, 1H, H10), 7.85 (m, 2H, H12 + H13) 7.7 (m, 2H, H1 + H2), 6.0 (m, 1H, 24), 5.2 (m, 2H, H25), 4.2 (dd, 1H, H23).

ESI MS^{+}: calc. for $C_{20}H_{15}N_3OSNa$ [M + Na]$^{+}$: 368.0834; found M/z = 368.0 (100%); calc. for $C_{20}H_{16}N_3OS$ [M + H^{+}]: 346.1014; found M/z = 346.0 (17.9%).

9. Synthesis of zinc mono (4-allyl-3-thiosemicarbazone) aceanthrenequinone, Zn(AA-Allyl)$_2$

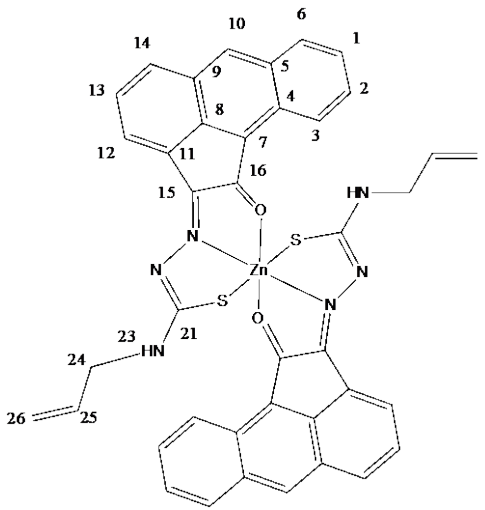

Free ligand AA-Allyl (0.025 g, 0.07 mmol) and zinc acetate (0.016 g, 0.07 mmol) and 20 ml of THF were mixed together. The reaction proceeded for 3 hours at the room temperature and then the solvent was removed by evaporation. Filtration of the solid yielded an orange compound that was separated by filtration and washed with Et_2O to remove impurities. Yield 0.042 g, 0.06 mmol, 79%.

^{1}H NMR (300MHz, d6-DMSO, 25oC). δ 9.60 (d, 1H, H3), 9.2 (s, 1H, H23), 8.5 (dd, 1H, H14), 8.4 (dd, 1H, H6), 8.3 (t, 1H, H10),. 8.0 (m, 2H, H12 + H13), 7.7 (m, 2H, H1 + H2), 6.0 (m, 1H, H24), 5.2 (m, 2H, H25), 4.2 (dd, 1H, H23).

ESI MS$^+$: calc. for $C_{40}H_{29}N_6O_2S_2Zn$ $[M + H]^+$: 753.1085; found**:** M/z 753.1077 and 757.1110.

10. Tert-butyl (2-aminoethyl)carbamate

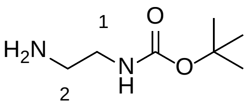

Di-tert-butyl dicarbonate (3.000 g, 13.70 mmol) in $CHCl_3$ (100 mL) was added dropwise to a stirring solution of ethylenediamine (4.960 g, 82.50 mmol) dissolved in $CHCl_3$ (100 mL) at 0 ºC over a period of 2 h. The reaction mixture was allowed to reach room temperature and stirred for 16 h. The solvent was removed under vacuum, the white residue re-suspended in $CHCl_3$ (150 mL) and washed with sat. aqueous sodium carbonate

(150 mL) and brine (100 mL). The organic phase was dried over $MgSO_4$ and concentrated under vacuum. The product was obtained as a yellowish oil (2.150 g, 98%).

^{1}H NMR (400 MHz, $CDCl_3$, 25 ºC): δ 4.99 (brs, 1H, NH), 3.21 – 3.07 (m, 2H, H-1), 2.76 (t, ^3J = 5.9 Hz, 3H, H-2), 1.42 (s, 9H, *(CH_3)$_3$*), 1.23 (s, 2H, *NH_2*). **^{13}C NMR** (125 MHz, $CDCl_3$, 25 ºC): δ 156.5 (*CO)*, 79.4 (*C*(CH_3)$_3$), 43.2 (C-1), 41.7 (C-2), 28.4 (*C*H_3)$_3$).

IR (solid): ν (cm^{-1}) 3359, 2986, 1684, 1528, 1268, 1165.

Mass spectrum: ESI-MS$^+$ calc. for $C_7H_{16}N_2NaO_2$ $[M+Na]^+$: 183.1109; found: 183.1110.

11. Tert-butyl (6-aminohexyl)carbamate

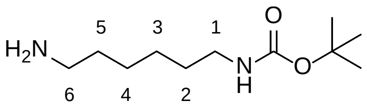

Hexamethylenediamine (39.930 g, 0.34 mol) was dissolved in CH_2Cl_2 (150 mL), triethylamine added (10 mL) and the reaction mixture cooled to 0 ºC. Di-tert-butyl dicarbonate (15.000 g, 68.70 mmol) dissolved in CH_2Cl_2 (150 mL) was then added dropwise over a 2 h period and the reaction mixture was allowed to warm-up to room temperature and stirred for another 16 h. The solvent was removed under vacuum to yield a white slurry that was re-dissolved in CH_2Cl_2 (150 mL) and washed with sat. sodium carbonate. The aqueous phase was extracted with CH_2Cl_2 (3x 100 mL), the organic layers collected, washed with brine, dried over $MgSO_4$ and concentrated under vacuum. The resulting residue was dissolved in CH_2Cl_2 and purified by flash column chromatography using CH_2Cl_2 / MeOH (0 - 10%) as eluent. The product was obtained as a yellowish oil that solidified over time (14.289 g, 96%).

^{1}H NMR (500 MHz, $CDCl_3$, 25 ºC): δ 4.52 (brs, 1H, *NH*), 3.12 – 2.96 (m, 2H, H-1), 2.68 (t, ^3J = 7.0 Hz, 2H, H-6), 1.49 – 1.37 (m, 13H, H-3, H-5, (CH_3)$_3$), 1.38 – 1.28 (m, 4H, H-3, H-4). **^{13}C NMR** (125 MHz, $CDCl_3$, 25 ºC): δ 156.1 (CO), 79.2 (*C*(CH_3)$_3$), 42.2 (C-6), 40.6 (C-1), 33.6 (C-5), 30.2 (C-3), 28.6 ((*C*H_3)$_3$), 26.8 (C-4), 26.6 (C-3). **Mass spectrum**: ESI-MS calc. for $C_{11}H_{24}N_2NaO_2$ [M+Na]$^+$: 239.1735; found: 239.1731. **IR (solid): ν (cm^{-1})** 3367, 2930, 1683, 1520, 1167.

12. Tert-butyl (2-(2-(2-aminoethoxy)ethoxy)ethyl)carbamate

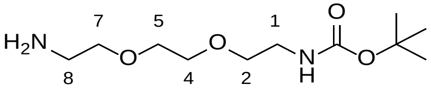

2,2’-(ethylenedioxy)bis(ethylamine) (20.507 g, 0.14 mol) was dissolved in 1,4-dioxane (40 mL) and cooled to 0 ºC. Di-tert-butyl dicarbonate (5.033 g, 0.02 mol) dissolved in 40 mL of 1,4-dioxane was added dropwise over a period of 2 h. The reaction mixture was allowed to warm to room temperature and left stirring for 16 h. The solvent was removed under vacuum to yield a white slurry that was re-dissolved in CH_2Cl_2 (500 mL) and washed with water (3x 300 mL) and brine (3x 150 mL). The organic phase was dried over $MgSO_4$ and concentrated under vacuum. The oil obtained was purified by flash column chromatography using CH_2Cl_2 / MeOH (0 – 10%) as eluent. The product was obtained as a yellowish oil (4.620 g, 81%).

^{1}H NMR (500 MHz, $CDCl_3$, 25 ºC): δ 5.23 (s, 1H, N*H*), 3.60 (s, 4H, H-4, H-5), 3.55 – 3.49 (m, 4H, H-2, H-7), 3.29 (brs, 2H, H-1), 2.87 (t, ^3J = 5.2 Hz, 2H, H-8), 1.42 (s, 9H, (CH_3)$_3$). **^{13}C NMR** (125 MHz, $CDCl_3$, 25 ºC): δ 156.2 (*C*O), 79.3 (*C*(CH_3)$_3$), 72.6, 70.3 (C-2, C-7), 70.3,

70.2 (C-4, C-5), 41.5 (C-8), 40.4 (C-1), 28.5 (*CH_3*)*3*). **Mass spectrum**: ESI-MS calc. for $C_{11}H_{24}N_2NaO_4$ $[M+Na]^+$: 271.1634; found: 271.1614.

IR (solid): ν (cm^{-1}) 3351, 2974, 1695, 1516, 1365, 1103.

13. 4-N-(2-tert-butoxycarbonylaminoethyl)-3-thiosemicarbazide

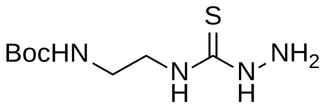

This compound was synthesised by a variation of the published method [2], as follows. Methyl-N-(2-tert-butoxycarbonylaminoethyl)dithiocarbamate (3.760 g, 15.00 mmol) was dissolved in ethanol (25 mL), hydrazine monohydrate (1.050 g, 21.00 mmol) added. Then, the mixture was heated under reflux for 2.5 h, the solvent was concentrated under vacuo and the residue resuspended in chloroform (20 mL). This was purified using a silica plug, washing with $CHCl_3$ (25 mL) first and then eluting with MeOH (50 mL). The methanolic fraction was concentrated under vacuo obtaining a yellowish oil that solidified over time (3.270g, 93%).

^{1}H NMR (400 MHz, *$CDCl_3$*, 25 ˚C): δ 7.80 (brs, 1H, *NH*NH_2), 5.04 (brs, 1H, *NH*Boc), 3.71 (appq, 2H, *CH_2*NHCS), 3.44 (s, 1H), 3.34 (appq, 2H, *CH_2*NHBoc), 1.40 (s, 9H, O*(CH_3)$_3$*).

ESI-MS$^+$ calculated for $C_8H_{18}N_4NaO_2S^+$ $[M+Na]^+$ 257.1048, found 257.1042

14. 4-N-(10-tert-butoxycarbonylaminohexyl)-3-thiosemicarbazide

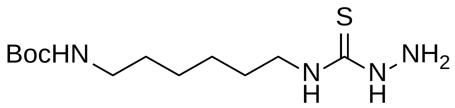

Methyl-N-(6-tert-butoxycarbonylaminohexyl)dithiocarbamate (4.410 g, 14.40 mmol) was dissolved in EtOH (50 mL), hydrazine hydrate (1.05 mL, 21.60 mmol) added and the reaction mixture heated under reflux for 2.5 h. then, the reaction mixture was concentrated under vacuum, the residue resuspended in $CHCl_3$ (25 mL) and the product passed through a silica plug, washed with $CHCl_3$, then eluted with MeOH (50 mL) and concentrated under vacuo. A yellowish solid was obtained (3.756 g, 90%).

^{1}H NMR (400 MHz, *$CDCl_3$*, 25 °C): δ 8.28, (brs, 1H, *NH*NH_2), 7.67 (brs, 1H, *NH*CS), 4.61 (brs, 1H, *NH*Boc), 3.57 (appq, 2H, *CH_2*NHCS), 3.07 (appq, 2H, *CH_2*NHBoc), 1.67 – 1.52 (m, 2H, *CH_2*CH_2NHCS), 1.49 – 1.42 (m, 2H, *CH_2*CH_2NHBoc), 1.40 (s, 9H, O*$(CH_3)_3$*), 1.37 – 1.26 (m, 4H, *CH_2CH_2*CH_2CH_2NHCS)

15. 4-ethylenediamine-3-thiosemicarbazone acenaphthenequinone (AN-10)

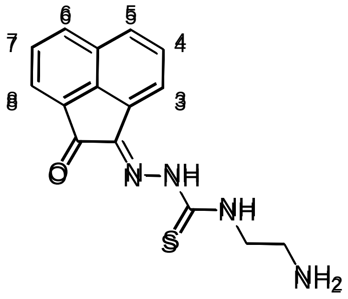

This compound was synthesized hereby by a variation of a published method, whereby the synthetic protocol using conventional heating was pursued, as follows. [2] Acenaphthenequinone (0.148 g, 0.81 mmol) and 4-N-(2-tert-butoxycarbonylaminoethyl)-3-thiosemicarbazide (0.200 g, 0.85 mmol) were suspended in ethanol under a nitrogen gas flow. The reaction mixture was heated up and 2 drops of concentrated hydrochloric acid were added upon reflux. After refluxing for 4 h the reaction mixture was filtered whilst hot, washed with diethyl ether and dried under vacuo. The product was obtained as a yellow solid (0.168 g, 60%).

^{1}H NMR (300 MHz, d^6-DMSO, 25°C) δ 12.69 (s, 1H, N*NH*CS), 9.71 (t, J = 5.8 Hz, 1H, *NH*CH_2), 8.37 (d, J = 8.1 Hz, 1H, *H-8*), 8.29 (brs, 2H, CH_2NH_2), 8.15 – 8.07 (m, 3H, *H-3, H-5, H-6*), 7.87 (dd, J = 7.6, 6.7 Hz, 1H, *H-7*), 7.82 (dd, J = 7.8, 6.7 Hz, 1H, *H-4*), 3.95 (appq, 2H, CSNH*CH_2*), 3.19 – 3.05 (m, 3H, *CH_2*NH_2).

^{13}C NMR (75 MHz, d^6-DMSO, 25°C) δ 188.66, 178.05, 139.31, 137.65, 132.94, 130.40, 129.93, 128.98, 128.67, 127.29, 122.60, 118.80, 41.90, 37.77, 36.52

16. 4-N-Boc-ethylenediamine-3-thiosemicarbazone acenaphthenequinone (AN-11)

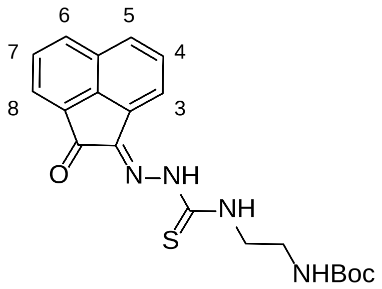

This compound was synthesized by a variation of a published method, whereby the synthetic protocol by conventional heating was carried out, as follows. [2] Acenaphthenequinone (0.125 g, 0.69 mmol) was suspended in 10 mL of ethanol in a Schlenk flask under a nitrogen stream. 4-Boc-ethylenediamine-3-thiosemicarbazide (0.160 g, 0.69 mmol) and 1 mL of acetic acid were then added to the suspension and the mixture heated under reflux for 3 h. The solid was isolated by filtration, washed with diethyl ether (20 mL) and dried under vacuo to give the desired compound as a bright yellow solid (0.147 g, 37%).

^{1}H NMR (400 MHz, *d*^{6}-DMSO, 25 ˚C): δ 12.61 (s, 1H, N*NH*), 9.42 (t, J = 5.1 Hz, 1H, CS*NH*), 8.37 (d, J = 8.1 Hz, 1H, *H-8*), 8.14 (d, J = 8.1 Hz, 1H, *H-3*), 8.10 (d, J = 7.1 Hz, 1H, *H-6*) 8.00 (d, J = 6.8 Hz, 1H, *H-5*), 7.90 – 7.80 (m, 2H, *H-4, H-7*), 7.09 (t, J = 5.5 Hz, 1H, *NH*Boc), 3.66 (appq, 2H, CSNH*CH_2*), 3.26 (appq, 2H, *CH_2*NHBoc), 1.39 (s, 9H, CO*$(CH_3)_3$*).

^{13}C NMR (75 MHz, DMSO) δ 188.54, 177.63, 156.28, 139.17, 137.24, 132.87, 132.32, 130.44, 130.01, 129.93, 128.87, 128.68, 128.51, 127.20, 122.57, 121.29, 118.28, 78.05, 44.95, 28.26

17. Tert-butyl (6-(((methylthio)carbonothioyl)amino)hexyl)carbamate

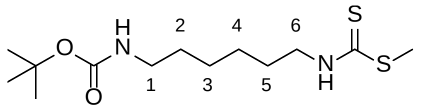

To a stirring ethanol solution of tert-butyl (6-aminohexyl)carbamate (4.150 g, 19.20 mmol) and triethylamine (5.3 mL, 38.30 mmol), at 25 ºC, CS2 (1.73 mL, 28.8 mmol) was added dropwise. The reaction mixture was stirred for 1.5 h when iodomethane (1.8 mL, 28.80 mmol) was added dropwise. After the addition finished, the stirring continued for another 2.5 h. The solvent was removed under vacuum and the residue re-suspended in ethyl acetate (50 mL) and washed with 1M HCl (50 mL), aq. sat. $NaHCO_3$ (50 mL) and water (50 mL). The organic fraction was dried over $MgSO_4$ and the solvent removed under vacuum. The product was obtained as a yellowish oil that solidified on standing (4.414 g, 75%).

^{1}H NMR (400 MHz, $CDCl_3$, 25 ºC): δ 7.72 (s, 1H, N*H*CS), 4.64 (s, 1H, CON*H*), 3.67 (q, ^3J = 6.7 Hz, 2H, H-6), 3.05 (q, ^3J = 6.7 Hz, 2H, H-1), 2.56 (s, 3H, SC*H*$_3$), 1.67 – 1.57 (m, 2H, H-5), 1.48 – 1.36 (m, 11H, H-2, (C*H*$_3$)$_3$), 1.34 – 1.28 (m, 4H, H-3, H-4). **^{13}C NMR** (125 MHz, $CDCl_3$, 25 ºC): δ 198.8 (*C*S), 156.2 (*C*O), 79.1 (*C*(C*H*$_3$)$_3$), 46.9 (C-6), 46.2, 40.1 (C-1), 30.0 (C-2), 28.5 ((*C*H$_3$)$_3$), 28.4 (C-5), 26.3, 26.2 (C-3, C-4), 18.1 (S*C*H$_3$). **ESI-MS+:** Calc. for $C_{13}H_{26}N_2NaO_2S_2$ $[M+Na]^+$: 329.1333; found: 329.1313

18. Tert-butyl (3-thioxo-7,10-dioxa-2-thia-4-azadodecan-12-yl)carbamate

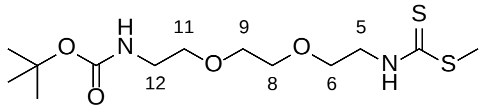

Tert-butyl (2-(2-(2-aminoethoxy)ethoxy)ethyl)carbamate (2.500 g, 10.10 mmol) and triethylamine (1.41 mL, 10.10 mmol) were dissolved in ethanol (50 mL) and warmed to 25 ºC. Carbon disulphide (0.606 mL, 10.1 mmol) was added dropwise and the reaction mixture stirred at 25 ºC for 2 h. Methyl iodide (0.63 mL, 10.1 mmol) was added to the reaction mixture further stirred for 2 h. The solvent was concentrated under vacuum, the residue dissolved in 50 mL AcOEt and washed with HCl 1M, sat. $NaHCO_3$ and water. The organic phase was dried over $MgSO_4$ and the solvent removed under vacuum. The resulting oil was passed through a silica plug using CH_2Cl_2 first and

then a mixture 8:2 of CH_2Cl_2 / MeOH. The second fraction was concentrated and dried under reduced pressure. The product was obtained as an off-white solid (2.971 g, 87%).

^{1}H NMR (500 MHz, $CDCl_3$, 25 ºC): δ 8.02 (s, 1H, N*H*CS), 5.10 (s, 1H, CON*H*), 3.78 (brs, 2H, H-5), 3.60 – 3.52 (m, 2H, H-6), 3.48 (s, 4H, H-8, H-9), 3.41 – 3.39 (m, 2H, H-11), 3.16 (brs, 2H, H-12), 2.45 (s, 3H, SC*H₃*), 1.28 (s, 9H). **^{13}C NMR** (125 MHz, $CDCl_3$, 25 ºC): δ 198.8 (*C*S), 170.8, 155.7 (*C*O), 78.9 (*C*(CH_3)$_3$), 69.9 (C-8, C-9), 69.9 (C-11), 68.1 (C-6), 46.5 (C-5), 40.0 (C-12), 28.1 ((*C*H_3)$_3$), 17.8 (S*C*H_3).

ESI-MS$^+$: Calc. for $C_{13}H_{26}N_2NaO_4S_2$ $[M+Na]^+$: 361.1232; found: 361.1218.

19. Tert-butyl (2-(hydrazinecarbothioamido)ethyl)carbamate was also synthesised following an optimised adaptation of the reported procedure using microwave irradiation [2] from the precursor tert-butyl (2-(((methylthio)carbonothioyl)amino)ethyl)carbamate. The spectroscopic data obtained by both conventional heating and microwave irradiation indicated the formation of the same compound in both cases. This product was obtained as a yellowish oil that solidified over time in 95% yield.

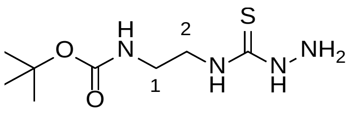

^{1}H NMR (500 MHz, $CDCl_3$, 25 ºC): δ 8.33 (s, 1H, N*H*NH_2), 7.89 (s, 1H, N*H*CS), 4.94 (s, 1H, CON*H*), 3.86 – 3.71 (m, 2H, H-2), 3.47 – 3.27 (m, 2H, H-1), 1.43 (s, 9H, (CH_3)$_3$). **^{13}C NMR** (125 MHz, $CDCl_3$, 25 ºC): δ 178.6 (*C*S), 159.3 (*C*O), 83.0 (*C*(CH_3)$_3$), 45.0 (*C*-2), 40.2 (*C*-1), 28.5 (*C*H_3)$_3$. **Mass spectrum**: ESI-MS calc. for $C_8H_{18}N_4NaO_2S$ $[M+Na]^+$: 257.1048; found: 257.1042. **IR (solid): ν (cm^{-1})** 3358, 3315, 3245, 2976, 2942, 1670, 1519, 1140. **HPLC** (Method A): Rt (min) 8.12. This was further synthesied according to the procedure reported by us earlier, [2] to give compounds mono(4-(2-aminoethyl)-3-thiosemicarbazone) acenaphthenequinone and mono (4-(tertbutyl-(2-aminoethyl)carbamate)-3-thiosemicarbazone) acenaphthenequinone below:

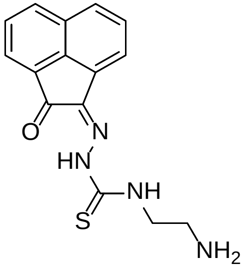

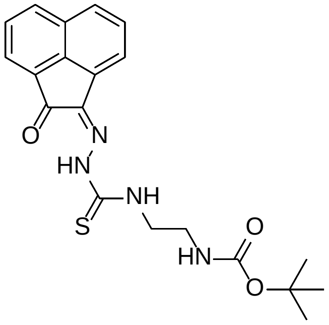

These were isolated here as yellow solids in 62% yield and 51% yields, respectively.

Mass spectrometry AN-10: ESI-MS calc. for $C_{15}H_{15}N_4OS$ $[M+H]^+$: 299.0967; found: 299.0959. **IR (solid): ν (cm^{-1})** 3280, 3204, 2836, 1693, 1523, 1467, 1452, 1050. **HPLC** (Method A): Rt 7.4 min.

Mass spectrometry AN-11: ESI-MS calc. for $C_{20}H_{22}N_4NaO_3S$ $[M+Na]^+$: 421.1310; found: 421.1329. **IR (solid): ν (cm^{-1})** 3384, 3326, 3260, 2980, 1719, 1685, 1670, 1512, 1480. **HPLC** (Method A): Rt (min) 10 min.

20. Tert-butyl (6-(hydrazinecarbothioamido)hexyl)carbamate

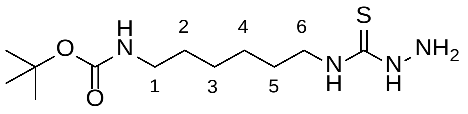

Methyl-N-(2-t-butoxycarbonylaminohexyl)dithiocarbamate (5) (4.414 g, 14.40 mmol) and hydrazine hydrate (1.0 mL, 21.60 mmol) were heated under reflux in ethanol for 2.5 h. The solvent was removed under vacuum and the residue redissolved in CH_2Cl_2 (20 mL) and passed through a silica plug that was washed with CH_2Cl_2 and the product eluted with MeOH. The product was obtained as a yellowish oil that solidified on standing (3.756 g, 90%).

^{1}H NMR (500 MHz, $CDCl_3$, 25 °C): δ 8.32 (brs, 1H, N*H*NH_2), 7.43 (brs, 1H, N*H*CS), 4.53 (brs, 1H, CON*H*), 3.77 (brs, 2H, NHNH_2), 3.62 (td, $^{3,3}J$ = 7.2, 5.8 Hz, 1H, H-6), 3.13 – 3.06 (m, 2H, H-1), 1.70 – 1.57 (m, 1H, H-5), 1.52 – 1.43 (m, 2H, H-2), 1.43 (s, 9H, $(CH_3)_3$), 1.41 – 1.32 (m,

4H, H-3, H-4). **^{13}C NMR** (125 MHz, $CDCl_3$, 25 ºC): δ 177.9 (*C*S), 156.0 (*C*S), 79.2 (*C*(C*H*$_3$)$_3$), 44.0 (C-6), 40.3 (C-1), 30.1 (C-2), 29.3 (C-5), 28.3 (*C*H$_3$)$_3$), 26.6, 26.5 (C-3, C-4). **Mass spectrum**: ESI-MS calc. for $C_{12}H_{27}N_4O_2S$ $[M+H]^+$: 291.1855; found: 291.1852. **IR (solid): ν (cm^{-1})** 3336, 3196, 2930, 2861, 1682, 1520, 1248, 1166. **HPLC** (Method A): Rt (min) 8.10.

21. Tert-butyl (2-(2-(2-(hydrazinecarbothioamido)ethoxy)ethoxy)ethyl)carbamate

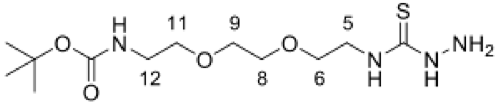

Tert-butyl (3-thioxo-7,10-dioxa-2-thia-4-azadodecan-12-yl)carbamate (2.776 g, 8.80 mmol) was dissolved in ethanol (60 mL), hydrazine hydrate (0.9 mL, 12.10 mmol) added and the reaction mixture heated under reflux for 3 h. The solution was left to cool and the solvent removed under vacuum. The residue was dissolved in $CHCl_3$ and passed through a silica plug, washing with $CHCl_3$ before eluting the product with MeOH. The resulting fraction was concentrated under vacuum to yield the product as a grey solid (1.928 g, 68%). **^{1}H NMR** (500 MHz, $CDCl_3$, 25 ºC): δ 7.98 (s, 1H, N*H*NH$_2$), 7.69 (s, 1H, N*H*CS), 5.13 (s, 1H, CON*H*), 3.92 (brs, 2H, N*H*$_2$), 3.75 (appq, ^3J = 5.3 Hz, 2H, H-5), 3.57 (t, ^3J = 5.1 Hz, 2H, H6), 3.54 (s, 4H, H-8, H-9), 3.45 (t, ^3J = 5.2, 1H, H-11), 3.21 (appq, ^3J = 5.7 Hz, 2H, H-12), 1.35 (s, 9H). **^{13}C NMR** (125 MHz, $CDCl_3$, 25 ºC): δ 181.8 (*C*S), 156.0 (*C*O), 79.2 (*C*(C*H*$_3$)$_3$), 70.1 (C-8, C-9), 70.1 (C-11), 69.5 (C-6), 43.6 (C-5), 40.3 (C-12), 28.4 (*C*H$_3$)$_3$). **Mass spectrum:** ESI-MS calc. for $C_{12}H_{27}N_4O_4S$ $[M+H]^+$: 323.1753; found: 323.1734. **IR (solid): ν (cm^{-1})** 3333, 3193, 3126, 2980, 2869, 1692, 1679, 1530, 1278, 1120. **HPLC** (Method A): Rt (min) 7.74.

22. Mono(4-(tertbutyl (2-(2-(2-aminoethoxy)ethoxy)ethylcarbamate)-3-thiosemicarbazone acenaphthenequinone AN-13

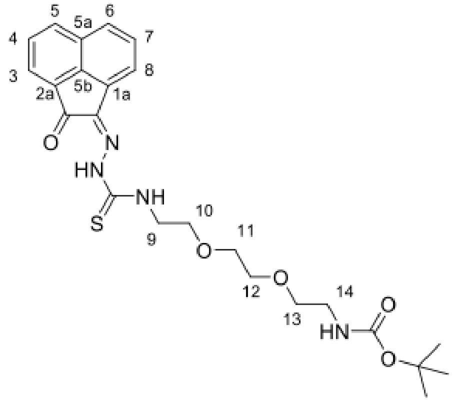

Acenaphthenequinone (0.042 g, 0.22 mmol) and tert-butyl (2-(2-(2-(hydrazinecarbothioamido)ethoxy)ethoxy)ethyl)carbamate (0.071 g, 0.22 mmol) were heated to 78 ºC for 3 h in ethanol (60 mL, 10% acetic acid). The product was purified by column chromatography using CH_2Cl_2 / MeOH (0 – 20%) as eluent. The product was obtained as a dark yellow oil that solidified over time (0.856 g, 88%).

^{1}H NMR (500 MHz, d^6-DMSO, 25 ºC): δ 12.62 (s, 1H, NN*H*), 9.29 (t, J = 5.7 Hz, 1H, N*H*CH_2), 8.36 (dd, $^{3,4}J$ = 8.3, 0.7 Hz, 1H, H-5), 8.12 (dd, $^{3,4}J$ = 8.4, 0.7 Hz, 1H, H-6), 8.07 (dd, $^{3,4}J$ = 7.1, 0.7 Hz, 1H, H-3), 7.95 (dd, $^{3,4}J$ = 7.0, 0.7 Hz, 1H, H-8), 7.86 (dd, $^{3,3}J$ = 8.2, 7.0 Hz, 1H, H-4), 7.82 (dd, $^{3,3}J$ = 8.3, 7.0 Hz, 1H, H-7), 6.74 (t, ^3J = 5.7 Hz, 1H, N*H*CO), 3.82 (q, ^3J = 5.9 Hz, 2H, H-9), 3.69 (t, ^3J = 5.9 Hz, 2H, H-10), 3.63 – 3.52 (m, 4H, H-11, H-12), 3.40 (t, ^3J = 6.1 Hz, 2H, H-13), 3.07 (q, ^3J = 6.0 Hz, 2H, H-14), 1.34 (s, 9H, $(CH_3)_3$).

^{13}C{^{1}H} NMR (125 MHz, d^6-DMSO, 25 ºC): δ 188.8 (*C*O), 178.6 (*C*S), 155.4, 140.2 (C-5b), 137.5 (*C*N), 132.7 (C-5), 130.8, 130.5, 130.2 (C-1a, C-2a, C-5a) 128.9 (C-7), 128.5 (C-4), 127.4 (C-6), 122.8 (C-3), 118.2 (C-8), 70.5, 70.5, 70.4 (C-11, C-12, C13), 69.4 (C-10), 44.6 (C-9), 40.5 (C-14), 28.6 ($(CH_3)_3$).

ESI-MS$^+$ Calc. for $C_{24}H_{31}N_4O_5S$ $[M+H]^+$: 487.2015; found: 487.2041. **Elem. Anal. (%).** Found (calc.) for $C_{24}H_{30}N_4O_5S$: C, 59.24 (57.94); H, 5.75 (6.21); N, 11.14 (11.51). **IR (solid): ν (cm^{-1})** 3358, 3270, 2974, 2868, 1687, 1524, 1476, 1171. **HPLC** (Method A): Rt (min) 9.99.

Mono(4-(tertbutyl-(6-aminohexyl)carbamate)-3-thiosemicarbazone acenaphthenequinone AN-12

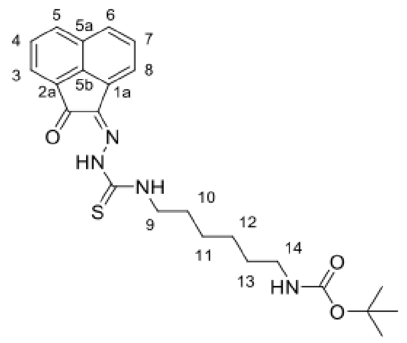

The compound denoted AN-12 was prepared from acenaphthenequinone (0.050 g, 0.27 mmol), tert-butyl (6-(hydrazinecarbothioamido)hexyl)carbamate (8) (0.318 g, 1.10 mmol) and hydrochloric acid (0.010 g, 0.27 mmol) heating in ethanol (5 mL) for 10 min at 90 ºC under microwave irradiation. The product was obtained as a bright yellow solid (0.112 g, 93%).

^{1}H NMR (300 MHz, d^6-DMSO, 25 ºC): δ 12.60 (s, 1H, NN*H*), 9.39 (t, ^3J = 5.9 Hz, 1H, *NH*CH$_2$), 8.38 (d, ^3J = 8.2 Hz, 1H, H-5), 8.14 (d, ^3J = 8.3 Hz, 1H, H-6), 8.09 (d, ^3J = 7.0 Hz, 1H, H-3), 8.01 (d, ^3J = 6.9 Hz, 1H, H-8), 7.91 – 7.86 (m, 1H, H-4), 7.86 – 7.79 (m, 1H, H-7), 6.77 (t, ^3J = 5.1 Hz, 2H, N*H*CO), 3.63 (q, ^3J = 6.8 Hz, 2H, H-9), 2.91 (q, ^3J = 6.4 Hz, 2H, H-14), 1.70 – 1.63 (m, 2H, H-10), 1.45 – 1.34 (m, 11H, H-13, (C*H*$_3$)$_3$), 1.34 – 1.27 (m, 4H, H-11, H-12).

^{13}C{^{1}H} NMR (75 MHz, d^6-DMSO, 25 ºC): δ 188.4 (*C*O), 177.1 (*C*S), 155.6 (NH*C*O), 139.0 (C-5b), 137.0 (*C*N), 132.8 (C-5), 130.4, 130.0, 129.8 (C-5a, C-2a, C-1a), 128.8 (C-7), 128.6 (C-4), 127.0 (C-8), 122.4 (C-6), 118.4 (C-3), 77.25 (*C*(CH$_3$)$_3$), 44.2 (C-9), 39.0 (C-14), 28.4 (C-10), 28.2 ((*C*H$_3$)$_3$), 26.0, 25.9 (C-11, C-12), 25.6 (C-13).

ESI-MS$^+$ Calc. for $C_{24}H_{29}N_4O_3S$ [M-H]$^-$: 453.1960; found: 453.1976. **HPLC** (Method A): Rt (min) 7.72.

General microwave-assisted protocols for optimised synthesis of simple, well-known AN-substituted ligands

The known ligands AN-Me, AN-Et, AN-Allyl and AN-Ph were synthesized as benchmark compounds and precursors for new copper complexes. These previously reported mono(substituted) thiosemicarbazones were re-synthesized for this study by the microwave-assisted heating, by treating one equivalent of the thiosemicarbazide containing N-R functionalities with one equivalent of corresponding quinone in the presence of an acid catalyst, as described below. Overall, all reactions were repeated at least three times and results were compared with those emerging from conventional synthesis protocols which were carried out according to published methods. [1-3][5-8] Briefly, the reaction mixtures were microwaved at 90°C while stirring for 10 minutes, affording compounds at high yields. All these known compounds were fully characterized aiming to probe whether they demonstrate identical spectroscopic characteristics to previously reported batches of compounds. These compounds were also shown to have a high purity by HPLC and were characterized by ^{1}H, $^{13}C\{^{1}H\}$ NMR, Infrared spectroscopy, and mass spectrometry. Additionally, an investigation into the use of an appropriate solvent for the microwave-driven protocols was also carried out, with 10% acetic acid in ethanol and 100% acetic acid. Results show that both solvents are appropriate giving identical compounds, in similar yields, and high purity by HPLC (characterized by the same retention times). Furthermore, for the known AN-Ph, extensive X-ray crystallographic characterization was carried out on single crystals grown from the batch emerging from microwave-assisted chemistry. Crystallography confirmed that this has an identical structure to the crystals isolated from previous batches. [2] Previously, An-Et was first reported and characterised structurally as a side product emerging from the synthesis by conventional heating of bis-thiosemicarbazones (BTSCs), [6] similarly AN-Allyl were also isolated by either conventional or microwave heating methods, and fully characterised structurally, to aid their applications in the synthesis of BTSCs, or corresponding gallium complexes formation. [1, 2, 7] These protocols were repeated hereby, and further optimised both by using conventional heating and microwave irradiation. The characterisation data of these known monoTSCs matched perfectly the earlier reported spectroscopic and X-ray diffraction data. [1-8]

Table S1 Comparison of optimized reaction yields for simple, known ligands.

HL	Conventional heating	Microwave-assisted irradiation
AN-Allyl	63%	93%
AN-Ethyl	85%	81%
AN-Phenyl	85%	87%

Mono(4-ethyl-3-thiosemicarbazone) acenaphthenequinone, AN-Ethyl

This compound was synthesised via an (optimised) adaptation of previously reported protocols by applying microwave irradiation [1-4].

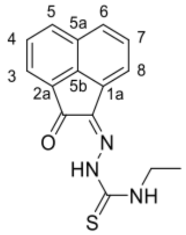

Briefly, 1 Equivalent of acenaphthenequinone (0.500 g, 2.75 mmol) added to 1 equivalent of 4-ethyl-3-thiosemicarbazide (0.361 g, 2.75 mmol) in ethanol (15ml) 3 drops of conc. HCl were added while stirring. The reaction mixture was then microwaved at 90°C for 10 minutes with 30 seconds stirring, yielding a yellow precipitate. The solid was filtered under reduced pressure and washed with ethanol (3x20 mL) and ether (3X20 mL). The resulting yellow solid was dried overnight. (0.630 g, 2.22 mmol, crude yield 81%). The product was further recrystalised from THF (70% yield).

^{1}H NMR (500 MHz, d^{6}-DMSO, 25 ºC): δ 12.60 (s, 1H, NN*H*), 9.42 (t, ^{3}J = 5.7 Hz, 1H, N*H*CH_2), 8.38 (d, ^{3}J = 8.2 Hz, 1H, H-5), 8.14 (d, ^{3}J = 8.3 Hz, 1H, H-6), 8.10 (d, ^{3}J = 7.0 Hz, 1H, H-3), 8.01 (d, ^{3}J = 7.0 Hz, 1H, H-8), 7.89 (dd, $^{3,3}J$ = 8.2, 7.0 Hz, 1H, H-4), 7.84 (dd, $^{3,3}J$ = 8.3, 7.0 Hz, 1H, H-7), 3.75 – 3.63 (m, 2H, CH_2), 1.23 (t, ^{3}J = 7.2 Hz, 3H, CH_3). **^{13}C NMR** (125 MHz, d^{6}-DMSO, 25 ºC): δ 188.5 (*C*O), 176.9 (*C*S), 139.1 (C-5b), 137.1 (*C*N), 132.8 (C-5), 130.4, 130.1, 129.9 (C-1a, C-2a, C-5a), 128.9 (C-7), 128.6 (C-4), 127.0 (C-6), 122.4 (C-3), 118.2 (C-8), 39.5 (*C*H_2), 14.0 (*C*H_3). **ESI-MS$^+$:** Calc. for $C_{15}H_{14}N_3OS$ [M+H]$^+$: 284.0857; found 284.0860. **IR (solid): ν (cm^{-1})** 3302, 3280, 2977, 1685, 1538, 1475, 1056, 1027. **HPLC**: Rt 11.0 min (HPLC Method A) or 9.90 min (HPLC method B).

Mono(4-allyl-3-thiosemicarbazone) acenaphthenequinone, AN-Allyl

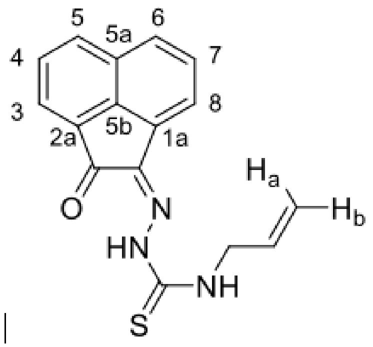

This compound was synthesised via optimised adaptations of previously reported protocols, and applying microwave irradiation [2]. Briefly, 1 equivalent of acenaphthenequinone (0.501 g, 2.75 mmol) added to 1 equivalent of 4-allyl-3-thiosemicarbazide (0.364 g, 2.75 mmol) in ethanol (15mL) 3 drops of conc. HCl were added while stirring. The reaction mixture was then microwaved at 90°C for 10 minutes with 30 seconds stirring, yielding a yellow precipitate. The solid was filtered under vacuum in a buchner funnel and washed with ethanol (3x20 mL) and ether (3X20 mL). The resulting yellow solid was dried overnight. (0.752 g, 2.55 mmol, crude yield= 93%). After recrystallisation from THF, the product was obtained as a yellow solid in 76% yield.

^{1}H NMR (500 MHz, d^6-DMSO, 25 ºC): δ 12.65 (s, 1H, NN*H*), 9.57 (t, ^3J = 5.8 Hz, 1H, N*H*CH$_2$), 8.38 (d, ^3J = 8.1 Hz, 1H, H-5), 8.14 (d, ^3J = 8.2 Hz, 1H, H-6), 8.10 (d, ^3J = 6.9 Hz, 1H, H-3), 8.01 (d, ^3J = 6.8 Hz, 1H, H-8), 7.88 (dd, $^{3,3}J$ = 8.2, 7.1 Hz, 1H, H-4), 7.84 (dd, $^{3,3}J$ = 8.3, 7.0 Hz, 1H, H-7), 6.04 – 5.89 (m, 1H, C*H*), 5.24 (ddd, $^{3,2,4}J_{trans, gem}$ = 17.2, 3.1, 1.5 Hz, 1H, Ha), 5.17 (ddd, $^{3,2,4}J_{cis,gem}$ = 10.3, 3.1, 1.5 Hz, 1H, Hb), 4.35 – 4.26 (m, 2H, NHCH_2). **^{13}C NMR** (125 MHz, d^6-DMSO, 25 ºC): δ 188.5 (*C*O), 177.6 (*C*S), 139.1 (C-5b), 137.3 (*C*N), 134.0 (*C*H), 132.8 (C-5), 130.4, 130.0, 129.9 (C-1a C-2a C-5a), 128.9 (C-7), 128.6 (C-4), 127.1 (C-6), 122.4 (C-3), 118.3 (C-8), 116.3 (*C*H$_2$), 46.4 (NH*C*H$_2$).

ESI-MS$^+$ Calc. for $C_{16}H_{13}N_3NaOS$ $[M+Na]^+$: 318.0677; found: 318.0666.

IR (solid): ν (cm^{-1}) 3318, 3049, 1688, 1523, 1478, 1178, 773.

HPLC (Method A): Rt (min) 11.38.

Mono(4-methyl-3-thiosemicarbazone) acenaphthenequinone, AN-Me

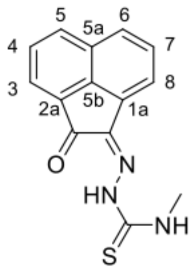

This compound was synthesised via optimised adaptations of previously reported protocols, and applying microwave irradiation hereby, [1-4] by analogy with **AN-ET** above, and resulted in a yellow solid in 70% yield. **^{1}H NMR** (500 MHz, d^{6}-DMSO, 25 ºC): δ 12.64 (s, 1H, NN*H*), 9.38 (q, ^{3}J = 4.5 Hz, 1H, N*H*CH_3), 8.37 (d, ^{3}J = 8.1 Hz, 1H, H-5), 8.13 (d, ^{3}J = 8.3 Hz, 1H, H-6), 8.09 (d, ^{3}J = 7.0 Hz, 1H, H-3), 7.97 (d, ^{3}J = 7.0 Hz, 1H, H-8), 7.88 (dd, $^{3,3}J$ = 8.1, 7.0 Hz, 1H, H-4), 7.84 (dd, $^{3,3}J$ = 8.3, 7.0 Hz, 1H, H-7), 3.11 (d, ^{3}J = 4.5 Hz, 3H, C*H*$_3$). **^{13}C{^{1}H} NMR** (125 MHz, d^{6}-DMSO, 25 ºC): δ 188.5 (*C*O), 177.9 (*C*S), 139.0 (C-5b), 137.1 (*C*N), 132.8 (C-5), 130.4, 130.1, 129.9 (C-1a C-2a C-5a), 128.9 (C-7), 128.6 (C-4), 127.0 (C-6), 122.4 (C-3), 118.1 (C-8), 31.4 (*C*H_3). **ESI-MS^{+}** Calc. for $C_{14}H_{11}N_3OS$ [M+H]$^{+}$: 270.0701; found 270.0700. **IR (solid): ν (cm^{-1})** 3219, 1689, 1540, 1475, 1055, 1027.
HPLC (Method A): Rt (min) 9.53.

Mono(4-phenyl-3-thiosemicarbazone) acenaphthenequinone (AN-Ph)

This compound was synthesised via further improved adaptations of previously reported protocols, and applying microwave irradiation [1-4]. Briefly, 1 Equivalent of acenaphthenequinone (0.501 g, 2.75 mmol) added to 1 equivalent of 4-phenyl-3-thiosemicarbazide (0.460 g, 2.75 mmol) in ethanol (15 mL) 3 drops of conc. HCl were added while stirring. The reaction mixture was then microwaved at 90°C for 10 minutes with 30 seconds pre-stirring, yielding an orange precipitate. The solid was filtered under vacuum in a Buchner funnel and washed with ethanol (3x20 mL)

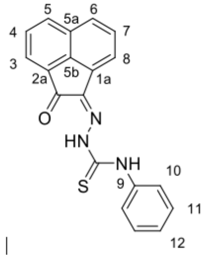

and ether (3 x 20 mL). The resulting orange solid was dried overnight. (0.789 g, 2.38 mmol, yield= 87%). A sample of advanced purity was obtained as an orange solid in 78% yield after additional recrystallisation from THF.

^{1}H NMR (500 MHz, d^{6}-DMSO, 25 ºC): δ 12.83 (s, 1H, NN*H*), 10.95 (s, 1H, CSN*H*), 8.40 (dd, $^{3,4}J$ = 8.3, 0.7 Hz, 1H, H-5), 8.16 (dd, $^{3,4}J$ = 8.4, 0.7 Hz, 1H, H-6), 8.14 (d, ^{3}J = 6.8 Hz, 1H, H-3), 8.12 (d, ^{3}J = 7.0 Hz, 1H, H-8), 7.90 (dd, $^{3,3}J$ = 8.2, 7.0 Hz, 1H, H-4), 7.86 (dd, $^{3,3}J$ = 8.3, 7.0 Hz, 1H, H-7), 7.65 (dd, $^{3,4}J$ = 8.5, 1.2 Hz, 2H, H-10), 7.45 (dd, $^{3,3}J$ = 8.4, 7.4 Hz, 2H, H-11), 7.32 – 7.28 (m, 1H, H-12). **^{13}C NMR** (125 MHz, d^{6}-DMSO, 25 ºC): δ 188.6 (*C*O), 176.6 (*C*S), 139.4 (C-5b), 138.5 (C-9), 137.7 (*C*N), 132.8 (C-5), 130.4, 129.9, 129.9 (C-1a, C-2a, C-5a), 128.9 (C-7), 128.6 (C-4), 128.4 (C-11), 127.2 (C-6), 126.1 (C-12), 125.7 (C-10), 122.5 (C-3), 118.8 (C-8).

ESI-MS^{+} Calc. for $C_{19}H_{14}N_{3}OS$ $[M+H]^{+}$: 332.0856; found: 332.0841. **IR (solid): ν (cm^{-1})** 3336, 3269, 3059, 1688, 1671, 1596, 1523, 1475.

HPLC Rt = 11.7 min (Method A); (Method B): Rt (min) 10.23.

X-ray diffraction studies (CCDC 2218630 for C19H13N3O1S1, C2H6OS demonstrated the identical structure of AN-Ph (DMSO adduct) to the previously reported structure [Ref 2, CCDC 2130510] and that this is a polymorph of the known structure 21340508, [2] .

Zn(II)[mono(allyl-thiosemicarbazonato) acenaphthenequinone]$_2$

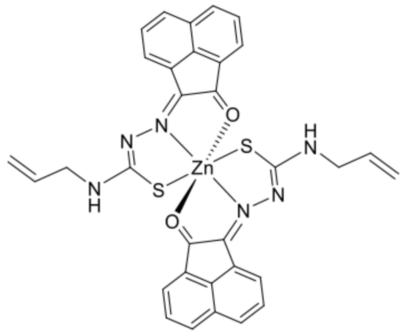

This compound was synthesised as previously reported [2] either by conventional heating, or by microwave synthesis. This was re-synthesised hereby as a benchmark compound for a comparison within the series. The product was obtained as an orange solid in 91% yield. **Mass spectrum**: ESI-MS calc. for $C_{32}H_{25}N_6O_2S_2Zn$ $[M+H]^+$: 653.0772; found: 653.0804. **Elem. Anal.** (%). Found (calc.) for $C_{32}H_{24}N_6O_2S_2Zn{\cdot}CHCl_3$: C, 50.53 (51.25); H, 3.15 (3.26); N, 10.90 (10.87). **IR** (solid): ν (cm^{-1}) 3321, 3059, 1675, 1484, 1247, 1090, 1026. **HPLC** (Method A): Rt (min) 9.98.

Similarly, Zn(II)[mono(ethyl-thiosemicarbazonato) acenaphthenequinone]$_2$ [Zn(AN-Ethyl)$_2$]; Zn(II)[mono(methyl)thiosemicarbazonato) acenaphthenequinone]$_2$ [Zn(AN-Me)$_2$] and Zn(II)[mono(phenyl)-thiosemicarbazonato)acenaphthenequinone]$_2$ [Zn(AN-Ph)$_2$] complexes were all synthesised according to published methods [2] and their purity was veriffied using HPLC and mass spectrometry. These were prepared by either conventional or microwave irradiation methods as benchmark compounds. Their identity and purity were checked by 1H NMR, MS and HPLC and were found to perfectly match the published data. [2]

2 Selected HPLC traces

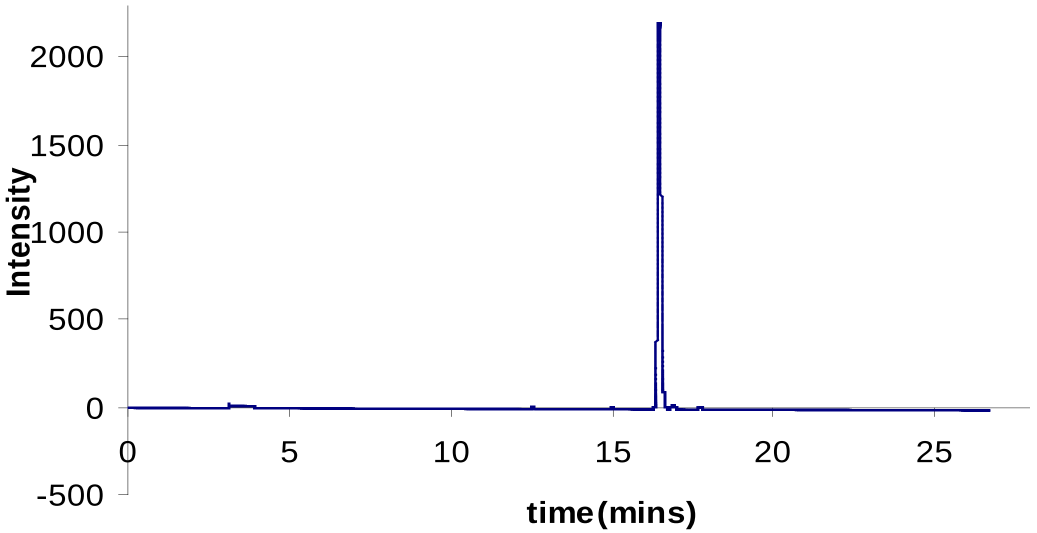

Figure S1 HPLC Trace of mono (4-allyl-3-thiosemicarbazone) phenantrenequinone PH-Allyl synthesized by conventional heating (UV detection 280 nm – HPLC Method A-1).

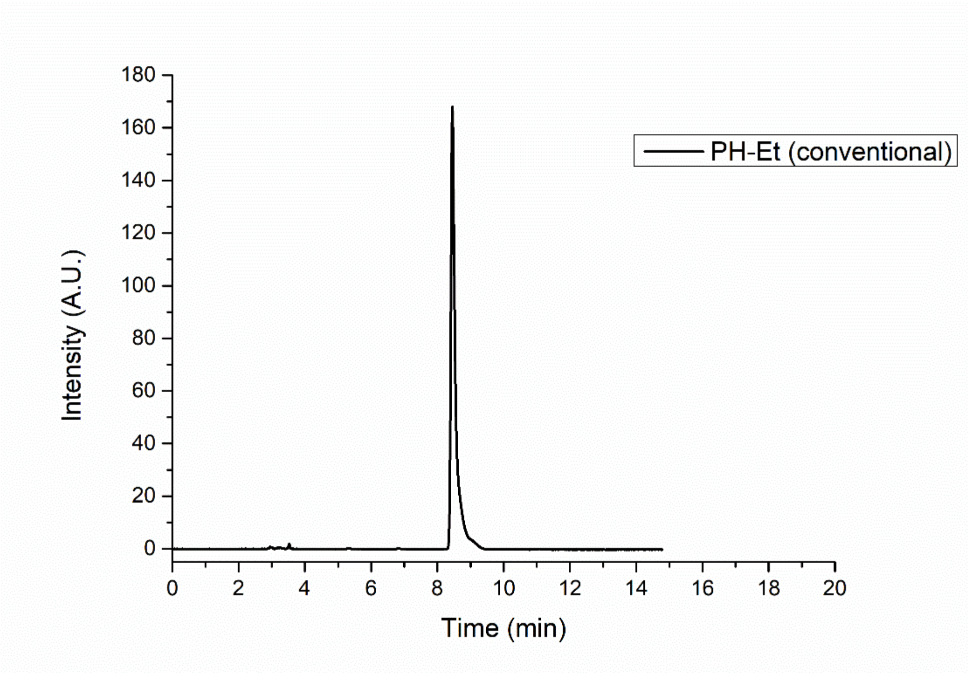

Figure S2 HPLC Trace of mono (4-ethyl-3-thiosemicarbazone) phenantrenequinone PH-Ethyl synthesized by conventional heating (UV detection 280 nm, Method B)

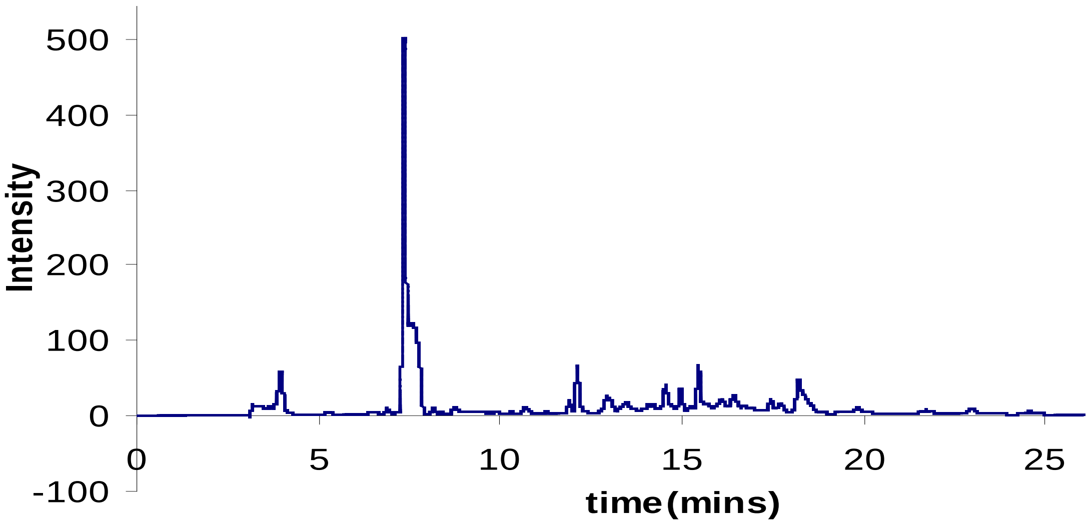

Figure S3 HPLC Trace of the Zn(II) complex of the mono (4-allyl-3-thiosemicarbazone) phenantrenequinone denoted Zn(PH-Allyl)$_2$, synthesized by conventional heating (HPLC Method A-1, UV detection 280 nm)

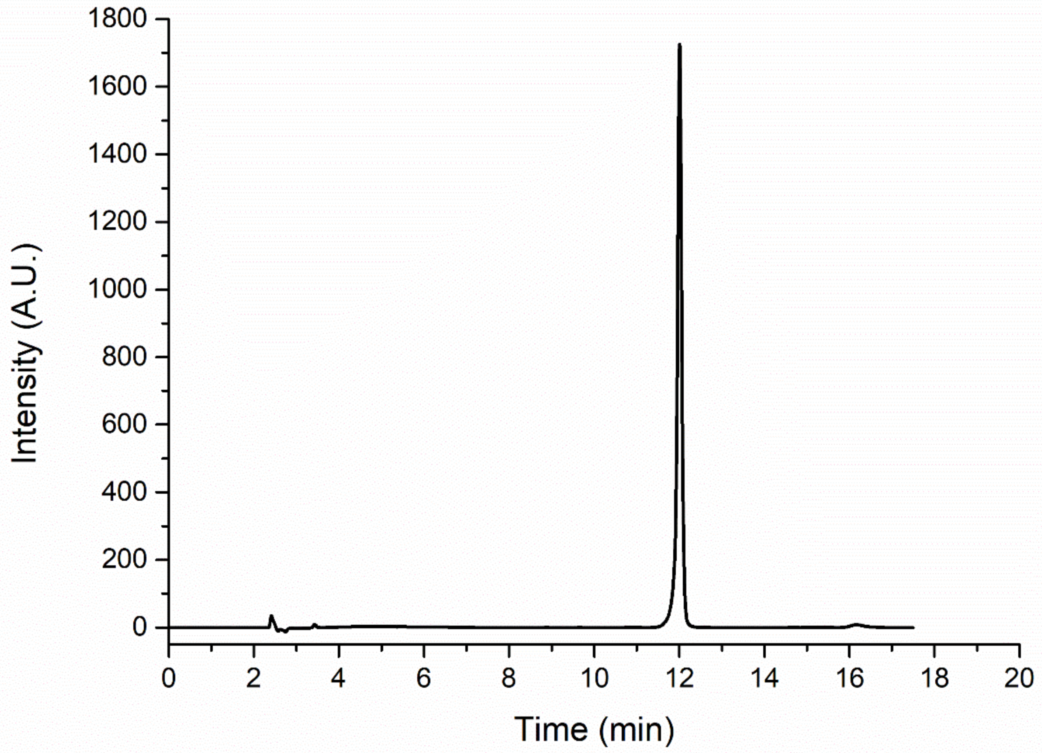

Figure S4 HPLC Trace of **AN-12** synthesized by microwave-assisted irradiation, (UV detection 450 nm, Method B)

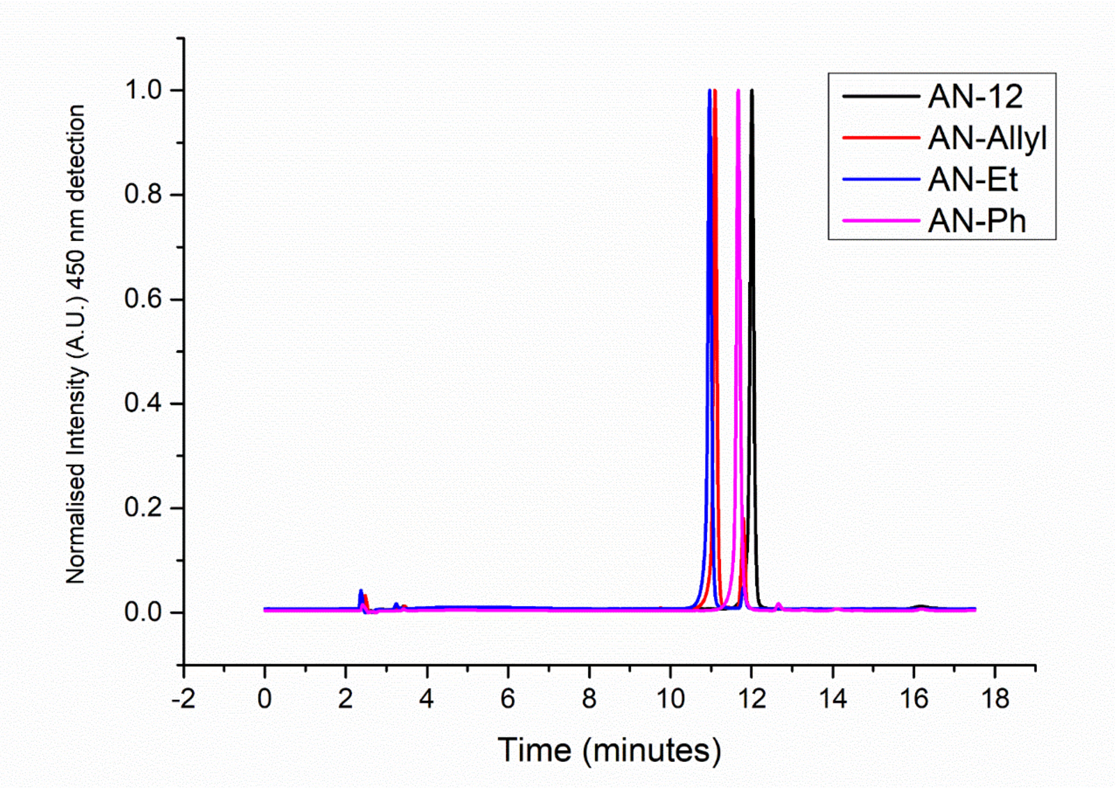

Figure S5. HPLC Trace of a series of TSC ligands with AN backbones (all synthesized by microwave-assisted irradiation), under UV detection 450 nm (Method B).

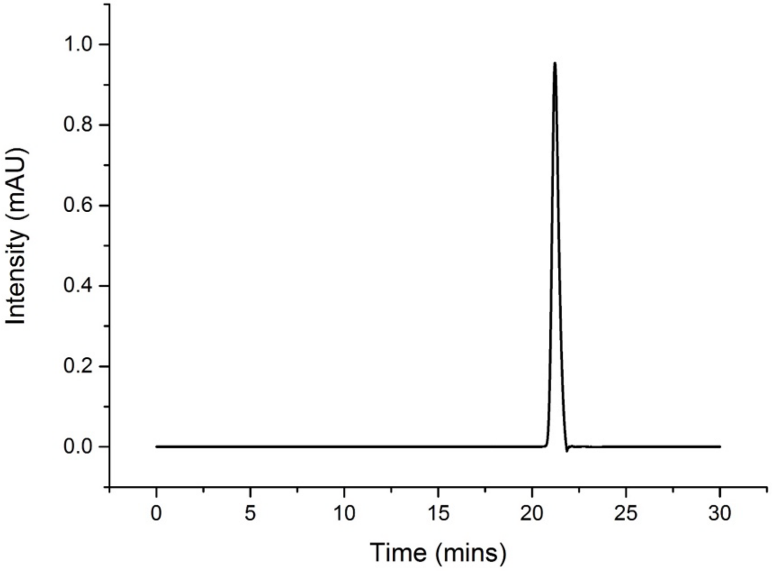

Figure S6. HPLC Trace of Zn(II) complex of mono (4-ethyl-3-thiosemicarbazone) acenapthenequinone Zn(AN-Et)$_2$ – optimized synthesis, by microwave-assisted irradiation. (UV detection 280 nm). Method C: reverse phase CH_3CN/H_2O with 0.1% TFA v/v; Dionex C-18 column (4.6 x 150mm), Ultimate 3000 Diode Array Detector. The pump flow was set to 1.8mL/min at 37 bar pressure.

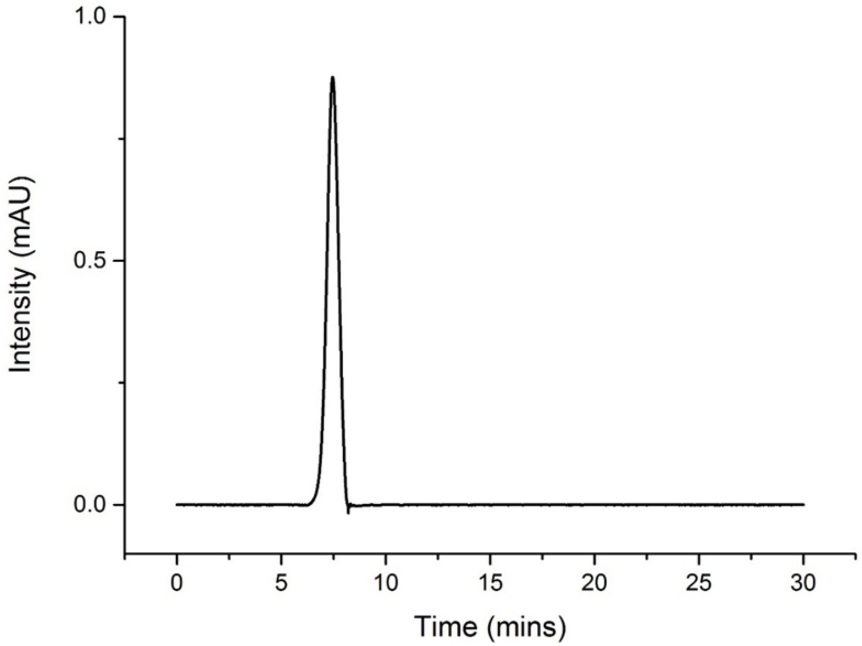

Figure S7. HPLC Trace of mono (4-ethyl-3-thiosemicarbazone) phenanthrenequinone (PH-Et, synthesized by microwave-assisted irradiation). (UV detection 280 nm), method Method C: reverse phase CH_3CN/H_2O with 0.1% TFA v/v; Dionex C-18 column (4.6 x 150mm), Ultimate 3000 Diode Array Detector. The pump flow was set to 1.8mL/min at 37 bar pressure.

.

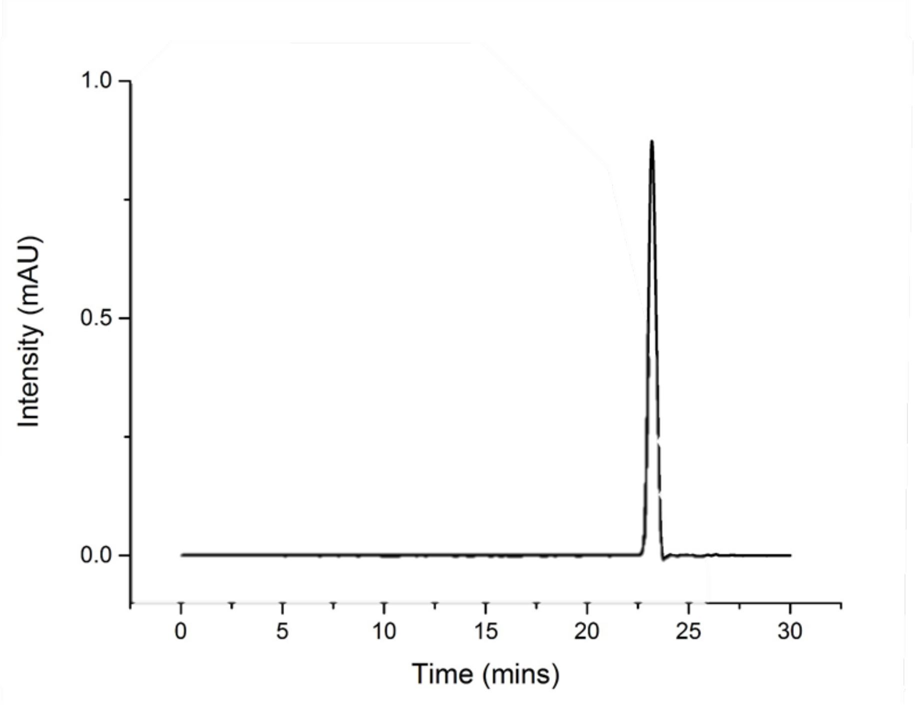

Figure S8. HPLC Trace of mono (4-ethyl-3-thiosemicarbazone)-4,5-pyrenedione (PY-Et, synthesized by microwave-assisted irradiation). (UV detection 280 nm, Method C)

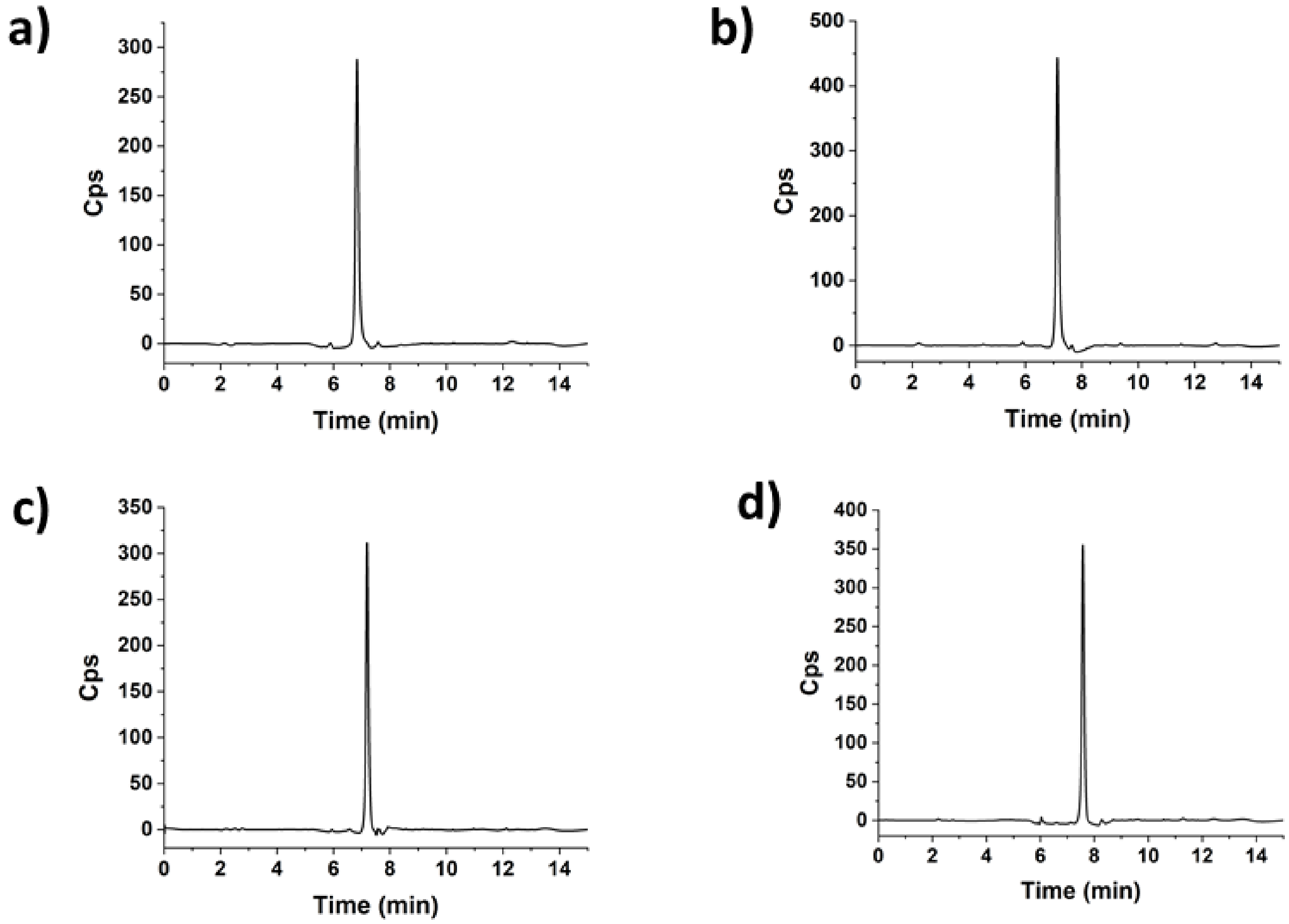

Figure S9 A comparison of HPLC traces: a) AN-Me, 6.84 min; b) AN-Et, 7.14 min; c) AN-Allyl, 7.19 min; d) AN-Ph, 7.58 min (synthesized by microwave-assisted irradiation) (UV detection 280 nm, Method B).

3 Optical spectroscopy of selected thiosemicarbazones

Table S2. Spectroscopic properties of ligands with AN backbones obtained by (optimized) microwave irradiation methods.

Compound	$\lambda_{em\text{-}max}$ / nm	Emission range / nm
AN-Allyl (1.1)	338	352 - 590
AN-Ethyl (1.2)	368	356 - 590
AN-Phenyl (1.3)	375	356 - 590
AN-12 (1.4) (AN-Hexyl-tBoc)	366	350 - 550

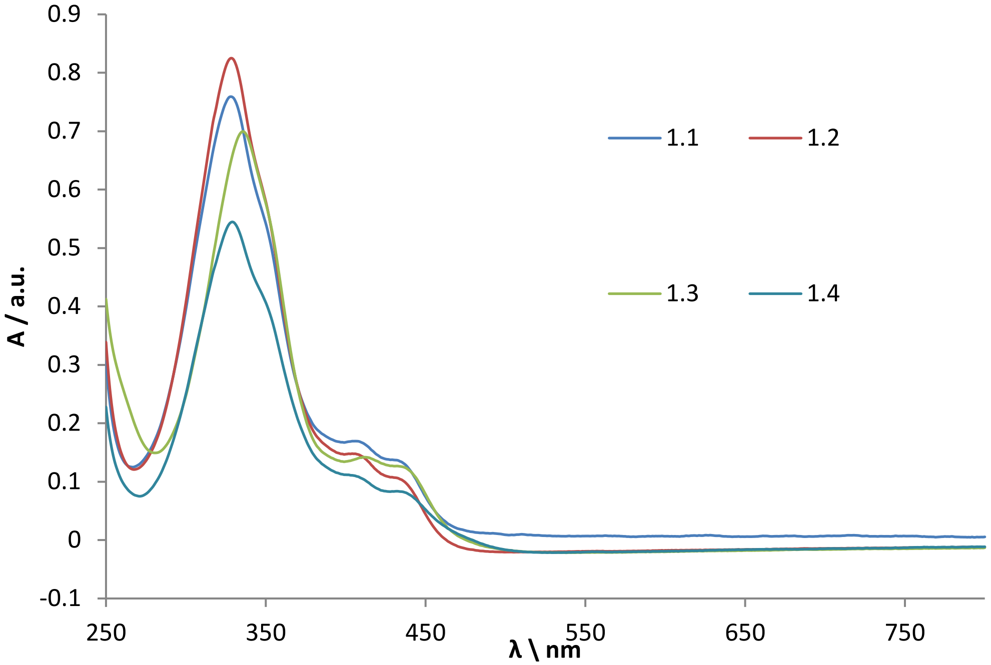

Figure S10. UV Vis spectroscopy of simple free ligands (50 µM conc in DMSO): AN-Allyl (1.1) AN-Ethyl (1.2) AN-Phenyl (1.3) AN-12 (1.4).

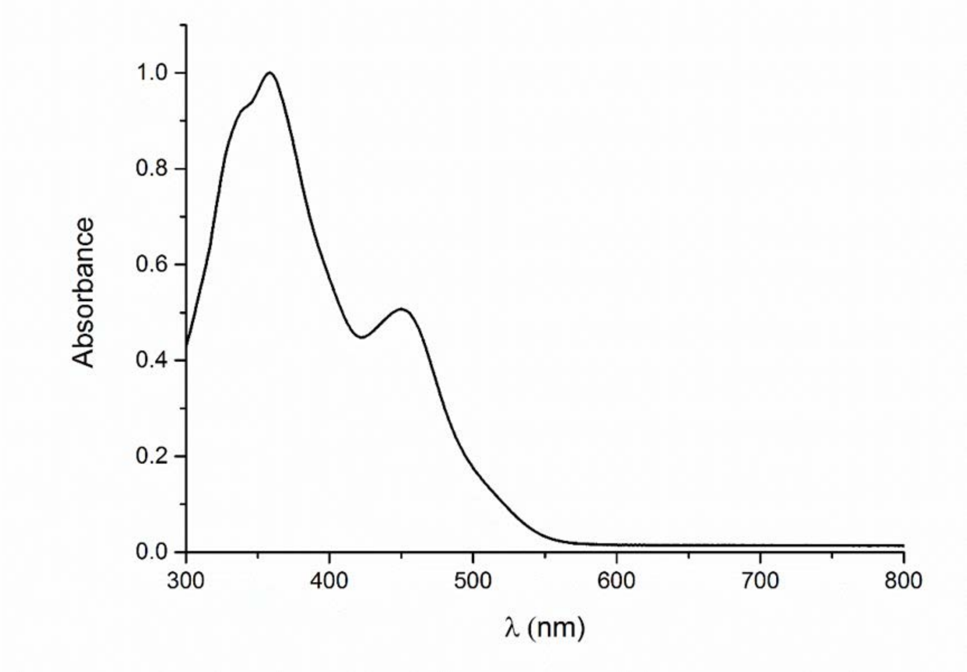

Figure S11 UV-VIS Spectrum of Zn(II) complex of mono (4-ethyl-3-thiosemicarbazone) acenapthenequinone [Zn(AN-Ethyl)$_2$], 25µM in DMSO.

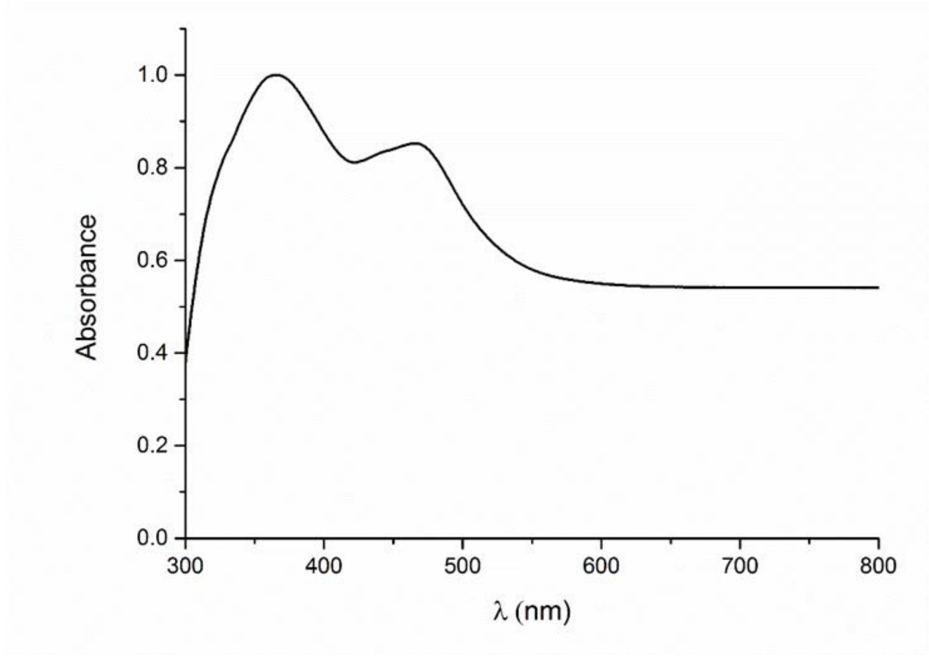

Figure S12 Selected range (300-800 nm) of the UV-VIS Spectrum of Zn(II) complex of mono (4-phenyl-3-thiosemicarbazone) acenapthenequinone, [Zn(AN-Phenyl)$_2$], 25µM in DMSO.

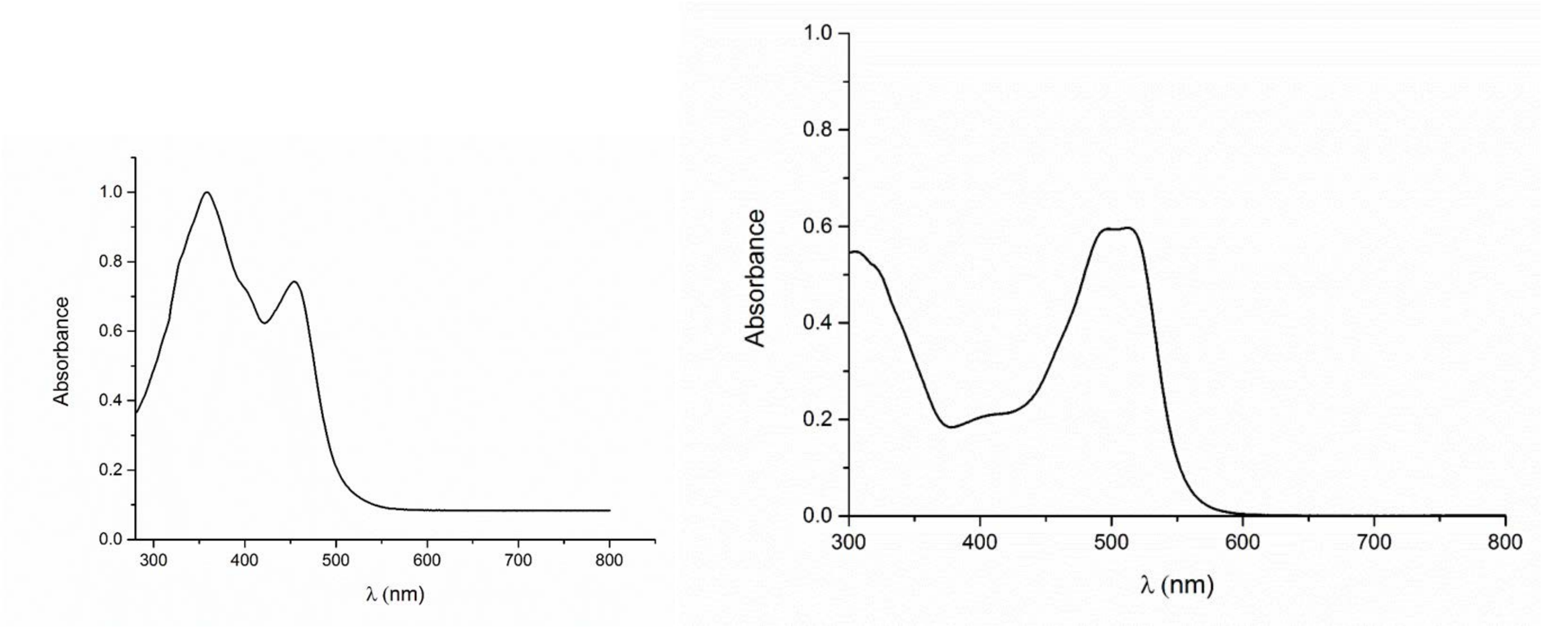

(a) (b)

Figure S13 Selected range of the **(a)** UV-VIS Spectrum of Zn(II) complex of mono(4-ethyl-3-thiosemicarbazone) aceanthrenequinone [Zn(AA-Ethyl)$_2$], 25µM in DMSO (280-800 nm). **(b)** UV-VIS Spectrum of Zn(II) complex of mono(4-ethyl-3-thiosemicarbazone) phenanthrenequinone [Zn(PH-Ethyl)$_2$], 25µM in DMSO (300-800 nm).

Table S3. Structural representations and spectral properties of selected Zn(II) complexes in DMSO solutions.

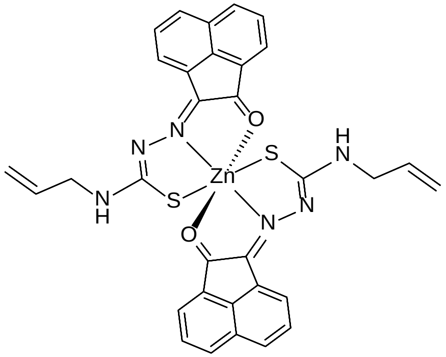

Zn(AN-Allyl)$_2$

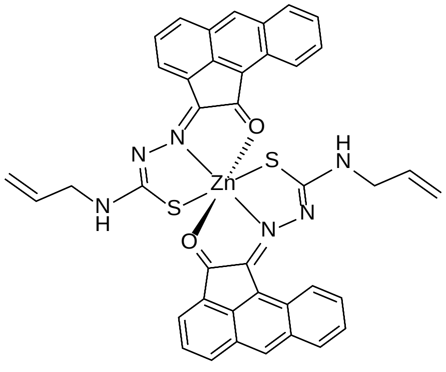

Zn(AA-Allyl)$_2$

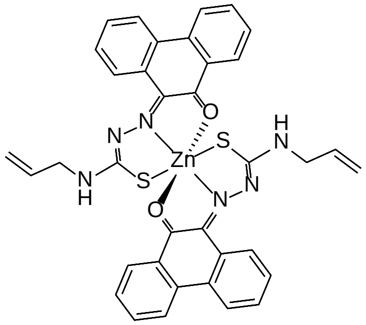

Zn(PH-Allyl)$_2$

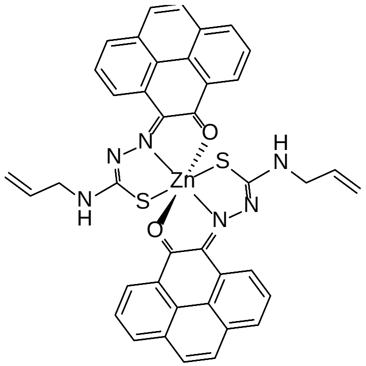

Zn(PY-Allyl)$_2$

Compound	$\lambda_{max\text{-}abs}$	$\lambda_{max\text{-}em}$	$\Delta\lambda$	Φ_f
Zn(AN-Allyl)2	360	628	268	4.48x10^{-}
Zn(PH-Allyl)2	517	610	93	3.83x10^{-}
Zn(PY-allyl)2	538	621	83	6.34x10^{-}
Zn(AA-Allyl)2	427	542	115	4.81x10^{-}

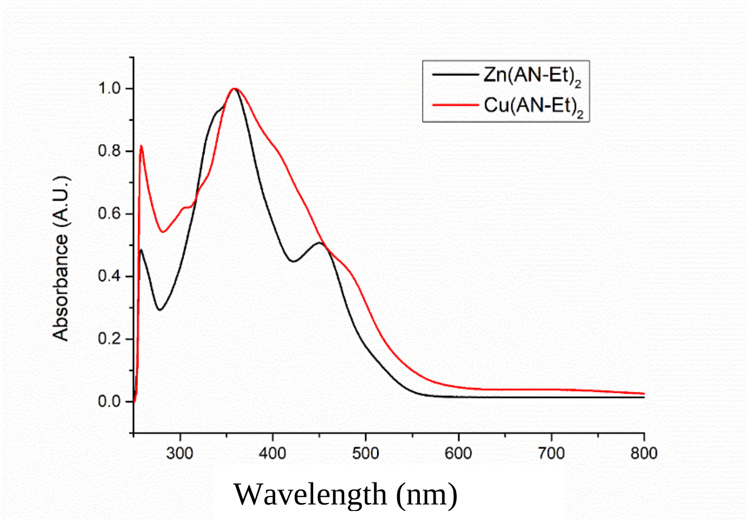

(a)

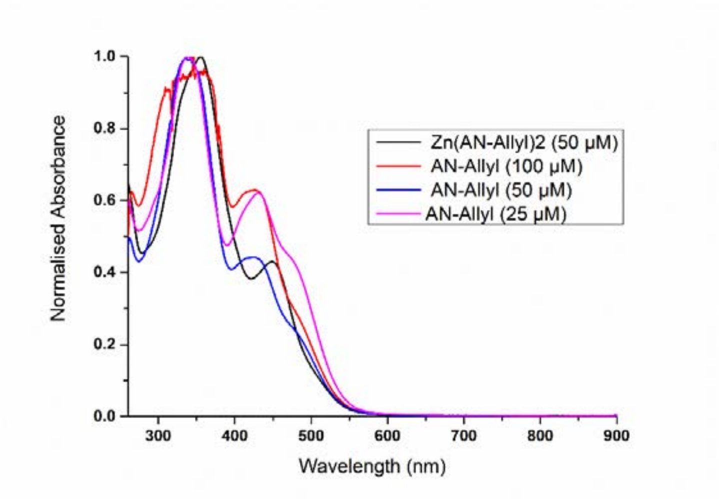

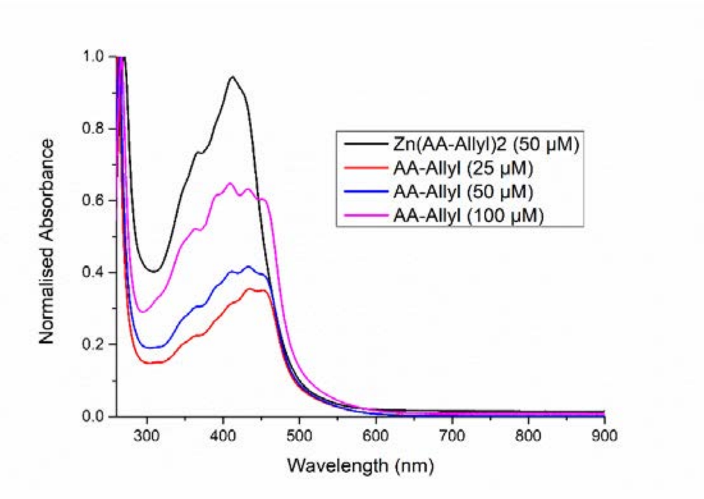

(b) **(c)**

Figure S14. Selected spectral range in the UV-vis spectroscopy: (a) Comparative UV Vis spectroscopy of Zn(II) and Cu(II) complexes of AN-Et ligand (25 uM conc, resulting from a 1:2 metal precursor:ligand reaction ratio, 280-800 nm)**; (b)** Comparative UV Vis spectrscopy of Zn(II) complex and free ligand AN-Allyl in a range of concentrations in the spetcral range 280-900 nm; **c)** Comparative UV Vis spectrscopy of Zn(II) complex and free ligand AA-Allyl in a range of concentrations 280-900 nm.

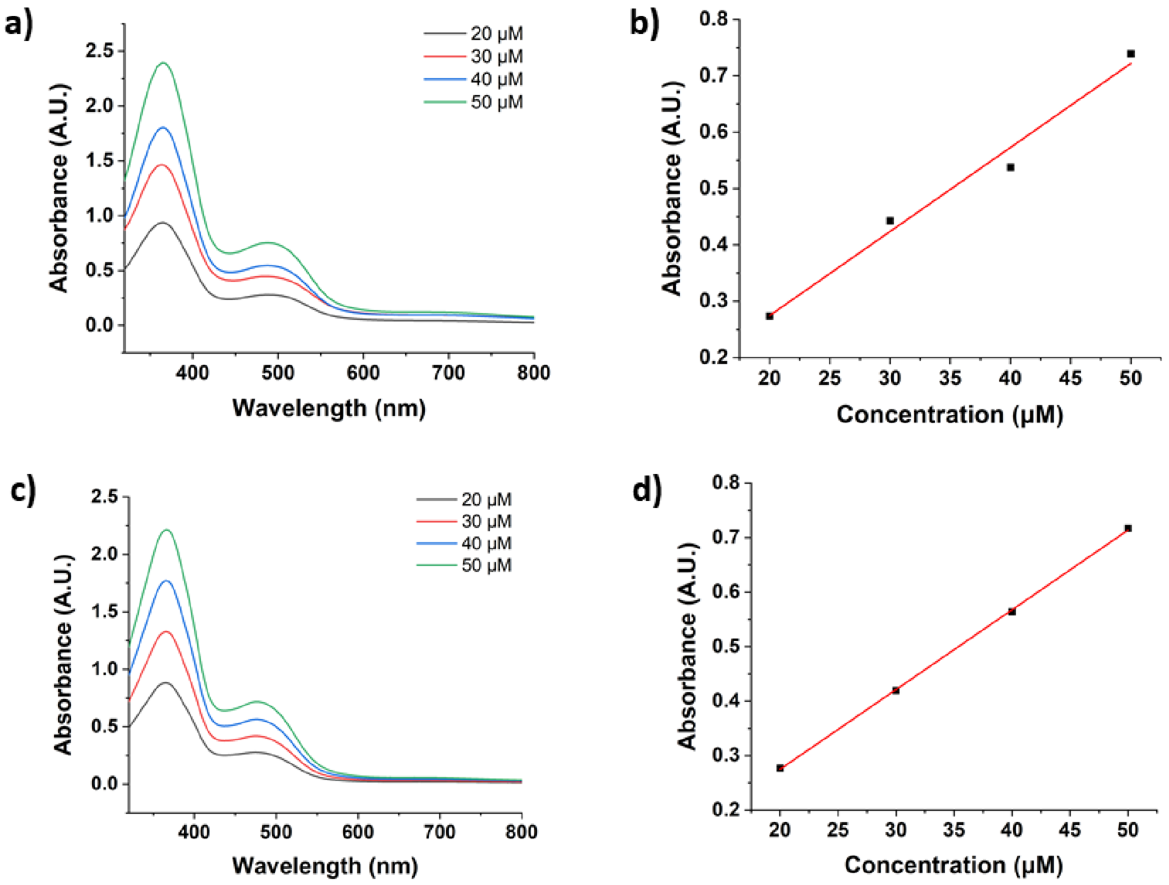

Figure S15 a) UV-Vis spectroscopy of the Cu(II) complex of the AN-Me ligand resulting from a 1:1 metal precursor:ligand ratio, denoted Cu-(AN-Me)-1:1. Spectra were recorded in DMSO; b) UV-Vis absorbance of Cu-(AN-Me)-1:1 ratio recorded in DMSO at 476 nm, ε=8693 L mol^{-1} cm^{-1}; c) UV-Vis spectroscopy of the Cu(II) complex of the AN-Me ligand resulting from a 1:2 metal precursor:ligand ratio, denoted -(AN-Me)-1:2; Spectra were recorded in DMSO in the 280-800 nm spectra range; d) UV-Vis absorbance of Cu-(AN-Me)-1:2 ratio recorded in DMSO at 476 nm, ε=8432 L mol^{-1} cm^{-1}.

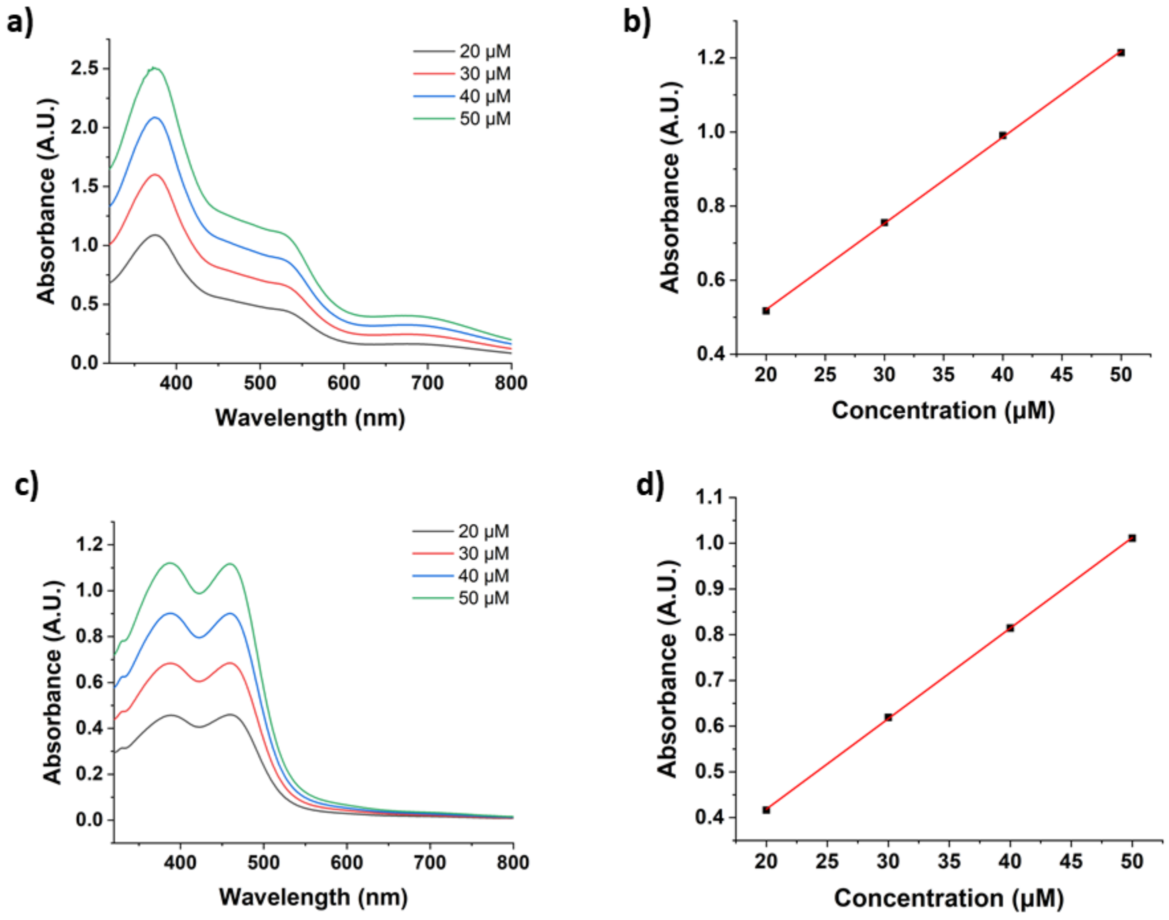

Figure S16 a) UV-Vis spectra of Cu-(AN-Ph)-1:1 ratio recorded in DMSO; **b)** UV-Vis absorbance of Cu-(AN-Ph)-1:1 ratio recorded in DMSO at 476 nm, ε=14284 L mol^{-1} cm^{-1}; **c)** UV-Vis spectra of Cu-$(AN\text{-}Ph)_2$-1:2 ratio recorded in DMSO; **d)** UV-Vis absorbance of Cu-$(AN\text{-}Ph)_2$-1:2 ratio recorded in DMSO at 476 nm, ε=11895 L mol^{-1} cm^{-1}. Spectra were recorded in the 280-800 nm spectra range.

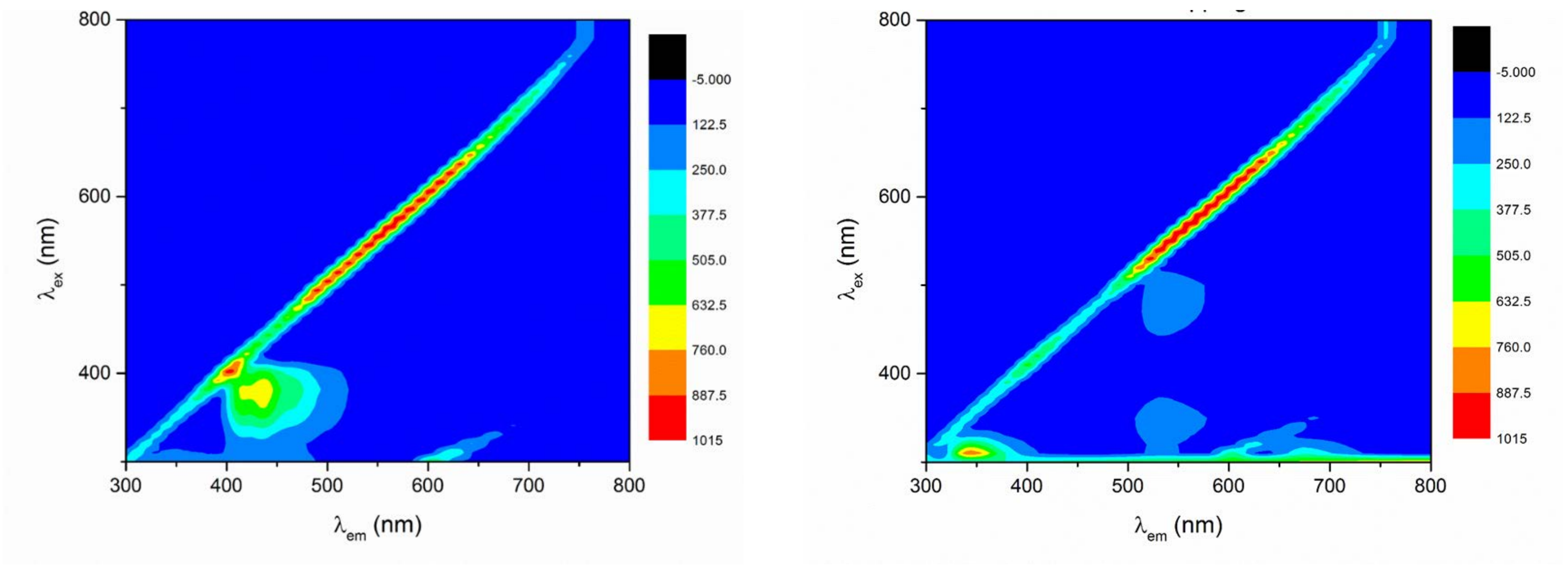

(a) **(b)**

Figure S17 (a) Excitation-Emission maps for Zn(II) complex of mono (4-ethyl-3-thiosemicarbazone) acenapthenequinone [Zn(AN-Ethyl)$_2$] at 25µM in DMSO; (b) Excitation-Emission maps for Zn(II) complex of mono (4-phenyl-3-thiosemicarbazone) acenapthenequinone [Zn(AN-Ph)$_2$] at 25µM in DMSO.

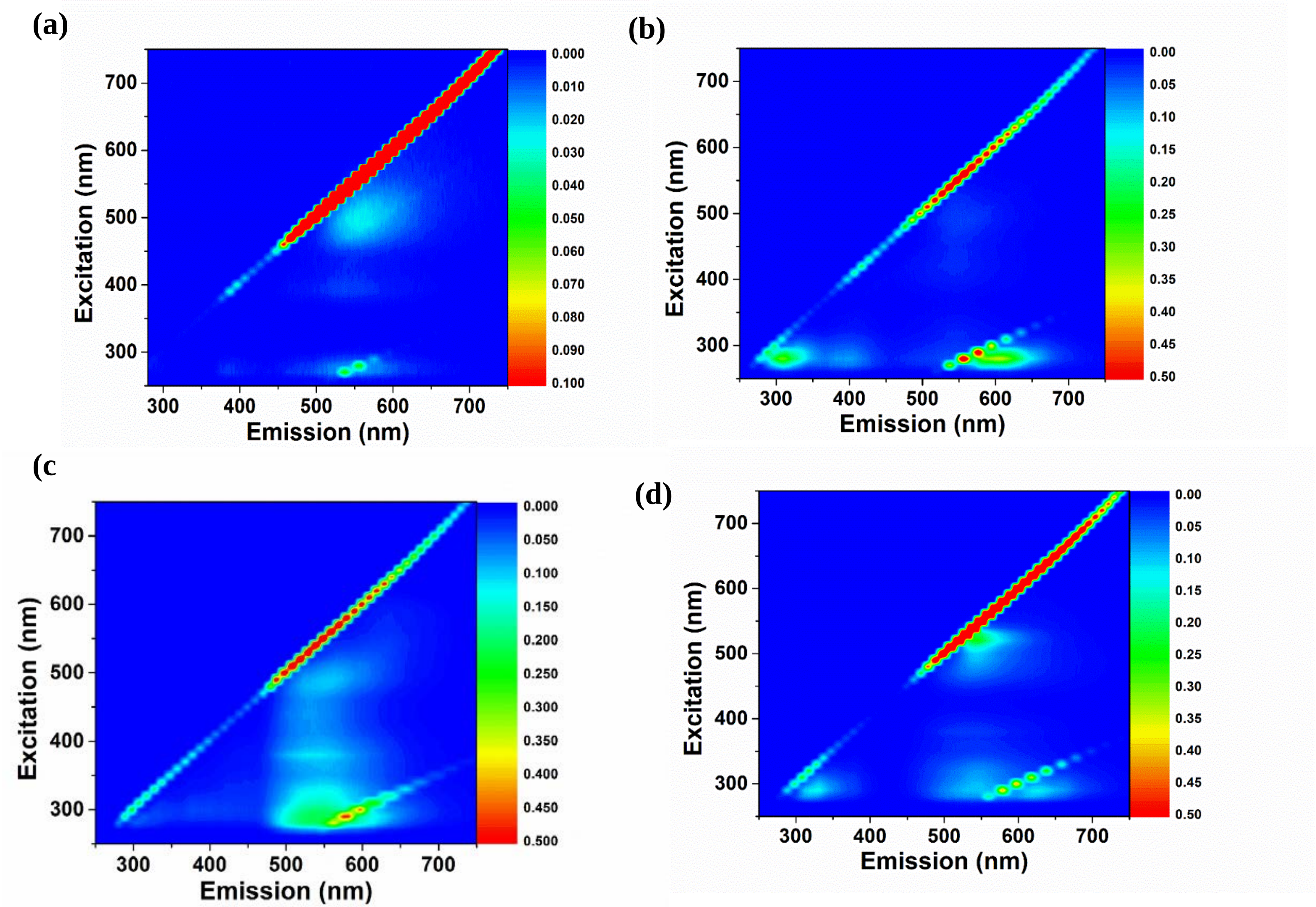

Figure S18 Normalized Excitation-Emission maps (50 µM in DMSO) for: (**a)** mono (4-allyl-3-thiosemicarbazone) acenapthenequinone ligand AN-Allyl; **(b)** the corresponding Zn(II) complex [Zn(AN-Allyl)$_2$]; **(c)** mono (4-allyl-3-thiosemicarbazone) aceanthrenquinone ligand AA-allyl and **(d)** the corresponding Zn(II) complex [Zn(AA-Allyl)$_2$].

4 NMR spectroscopy of selected compounds

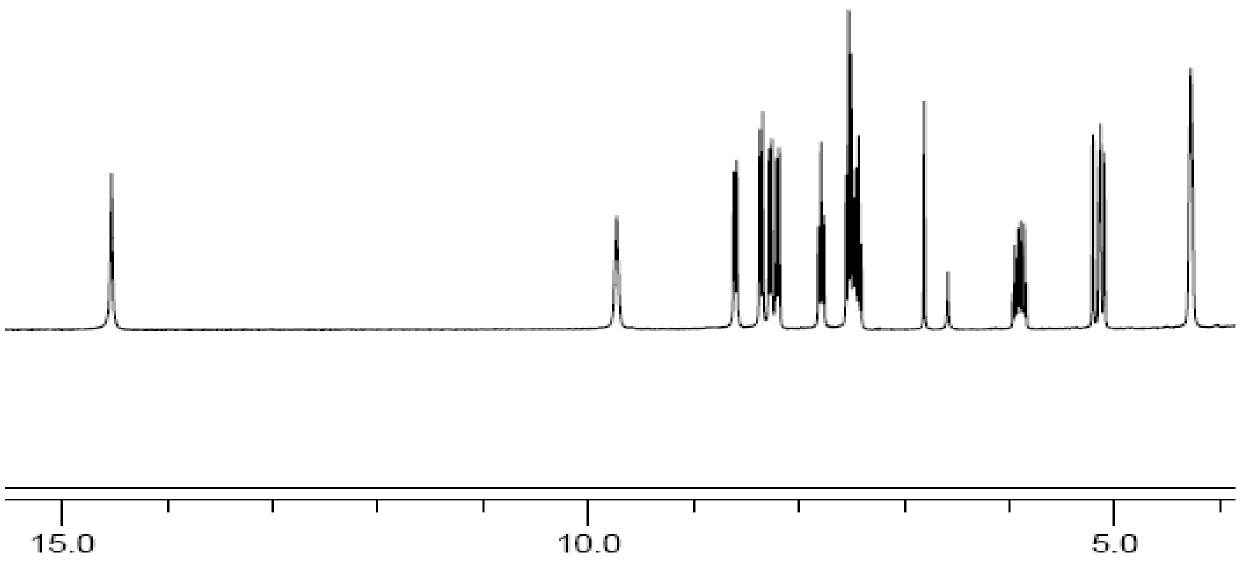

Figure S19 ^{1}H NMR (300MHz, d^6-DMSO, 25°C) of PH-Allyl ligand synthesized by conventional heating (expansion).

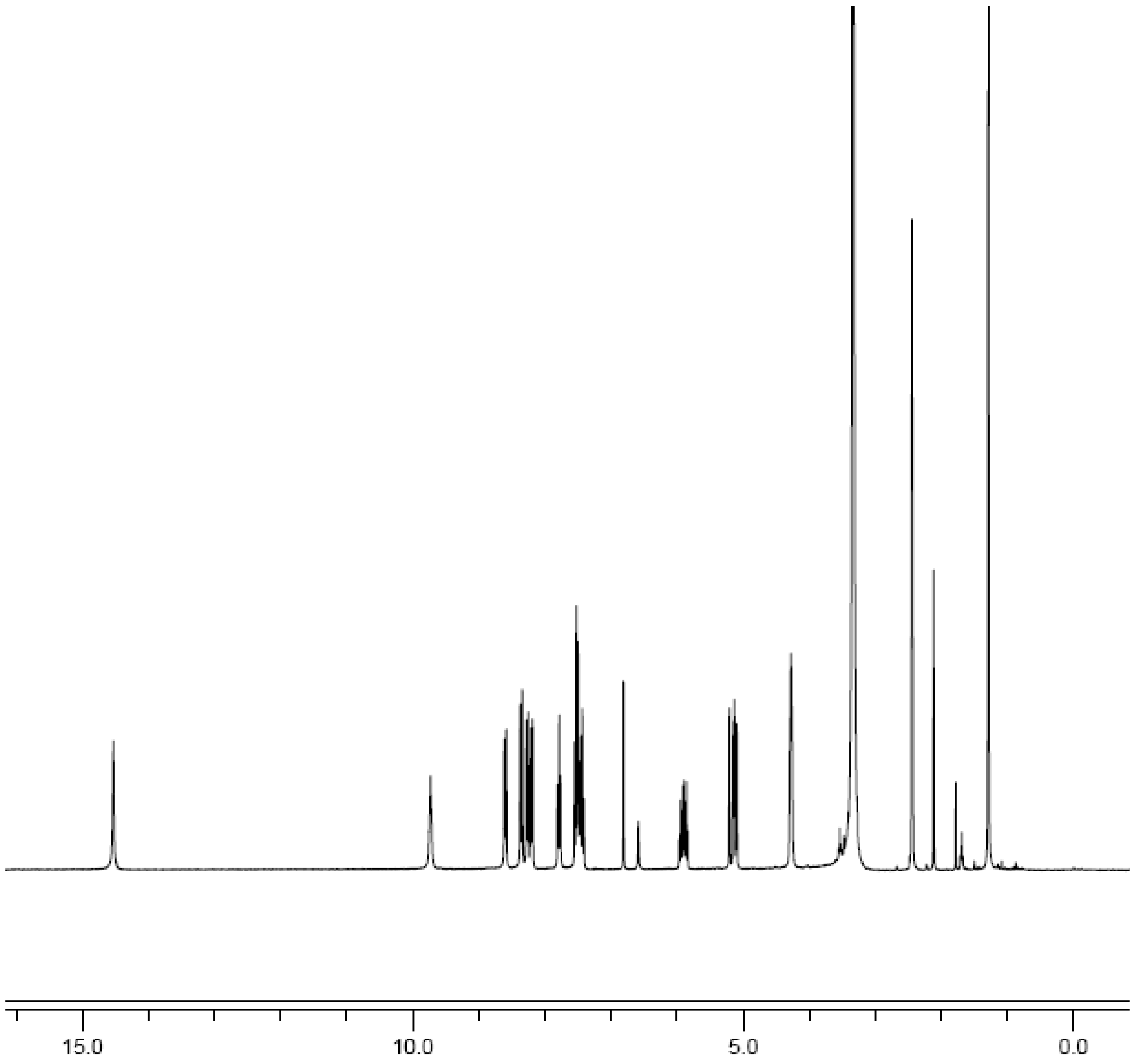

Figure S20 ^{1}H NMR (300MHz, d^{6}-DMSO, 25°C) of PH-Allyl ligand synthesized by conventional heating (full spectrum).

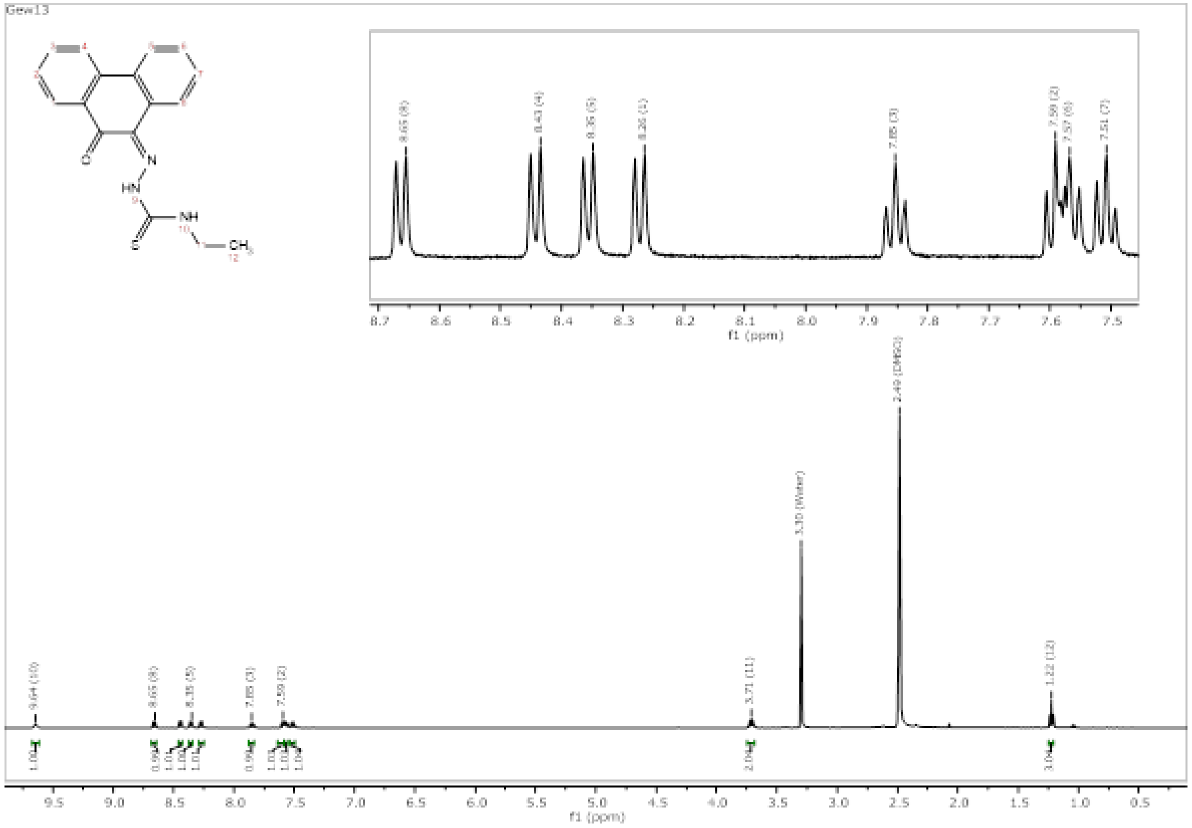

Figure S21 ^{1}H NMR spectrum (500 MHz, d^{6}-DMSO, 25℃) of PH-Et ligand synthesized by the microwave-assisted irradiation method. Insert: An expansion of the aromatic region of the ^{1}H spectrum of PH-Et.

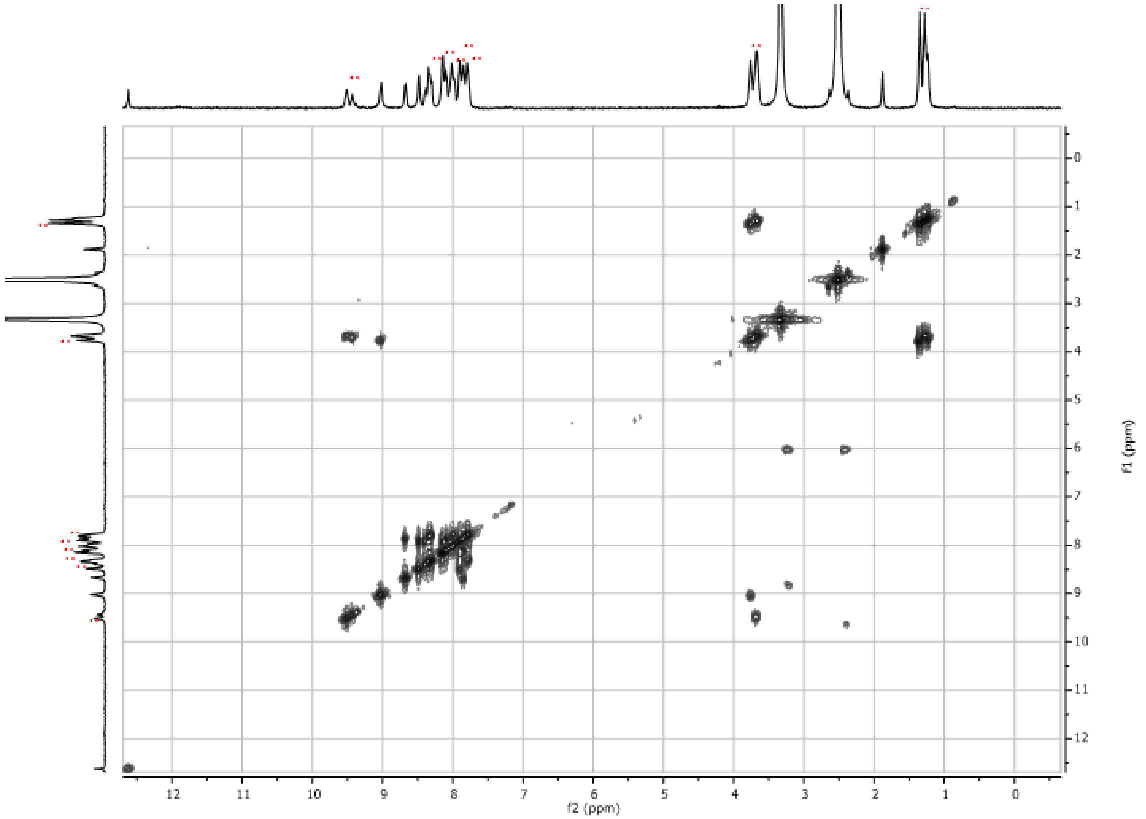

Figure S22 ^{1}H -^{1}H COSY NMR spectrum (500 MHz, d^{6}-DMSO, 25°C) of the Zn(II) complex of mono (4-ethyl-3-thiosemicarbazone) acenapthenequinnone, [Zn(AN-Et)$_2$]

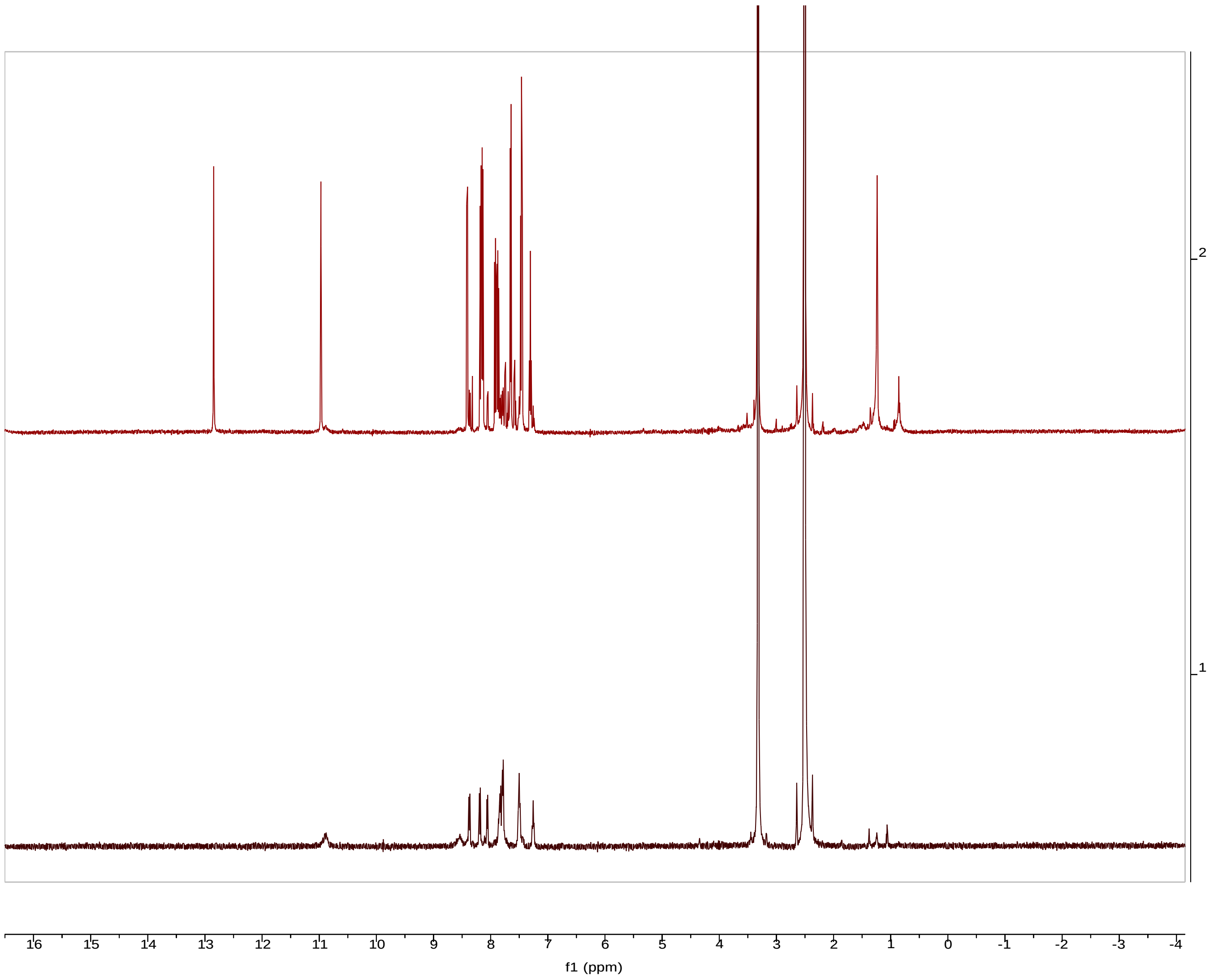

Figure S23 Comparison of ^{1}H NMR spectrum (500 MHz, d^{6}-DMSO, 25°C) of AN-Ph (top spectrum), and the corresponding Zn(II) complex [Zn(AN-Ph)$_2$] (bottom spectrum), recorded from samples of corresponding compounds synthesized by microwave-assisted irradiation.

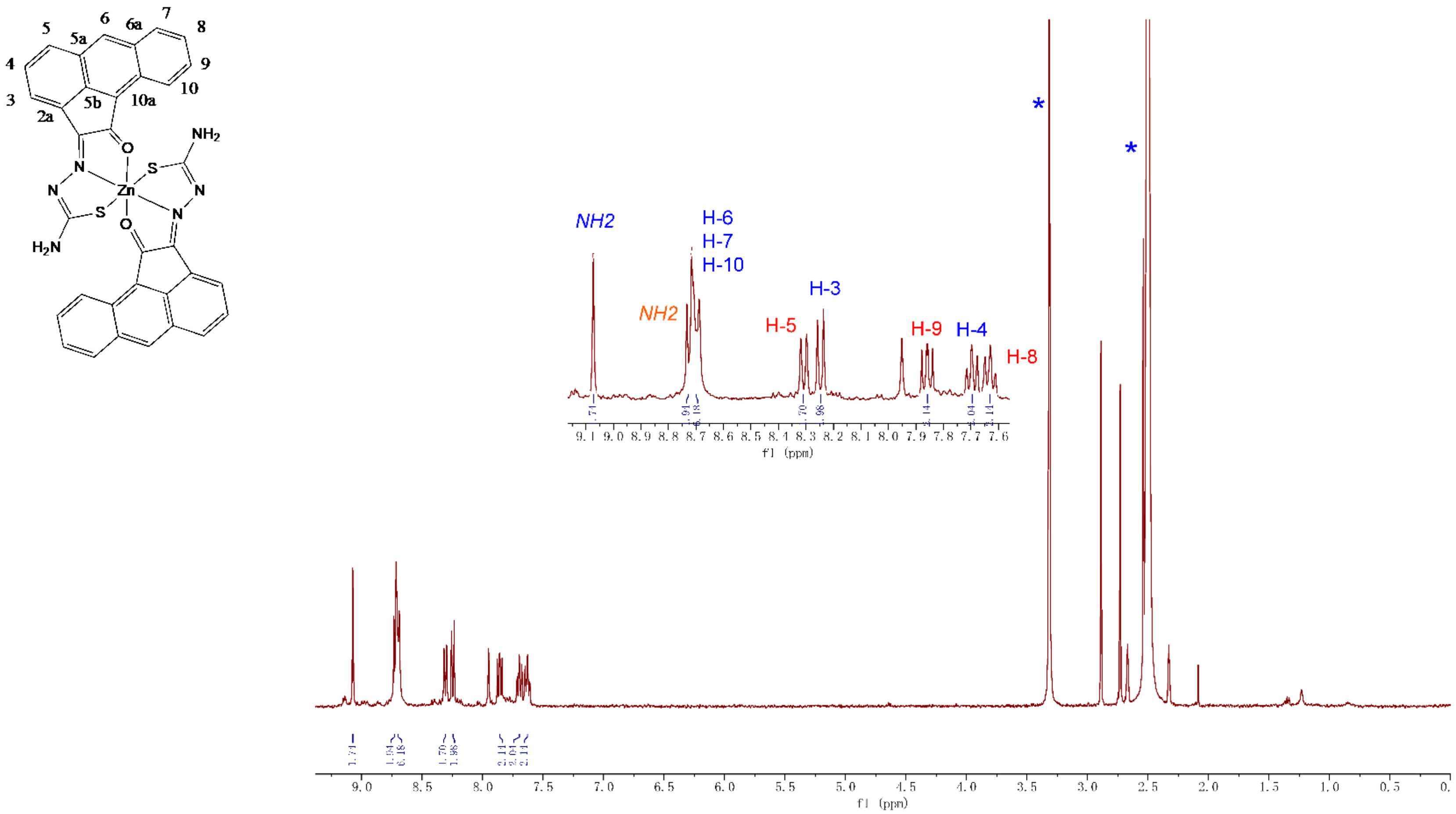

Figure S24 ^{1}H-NMR spectrum (400 MHz, d^{6}-DMSO) of [**Zn(AA-H)$_2$**], recorded from samples of the compound synthesised by microwave irradiation. (* correspond to the residual solvents, d^{6}-DMSO and water). Insert: An expansion of the aromatic region of the ^{1}H spectrum of PH-Et.

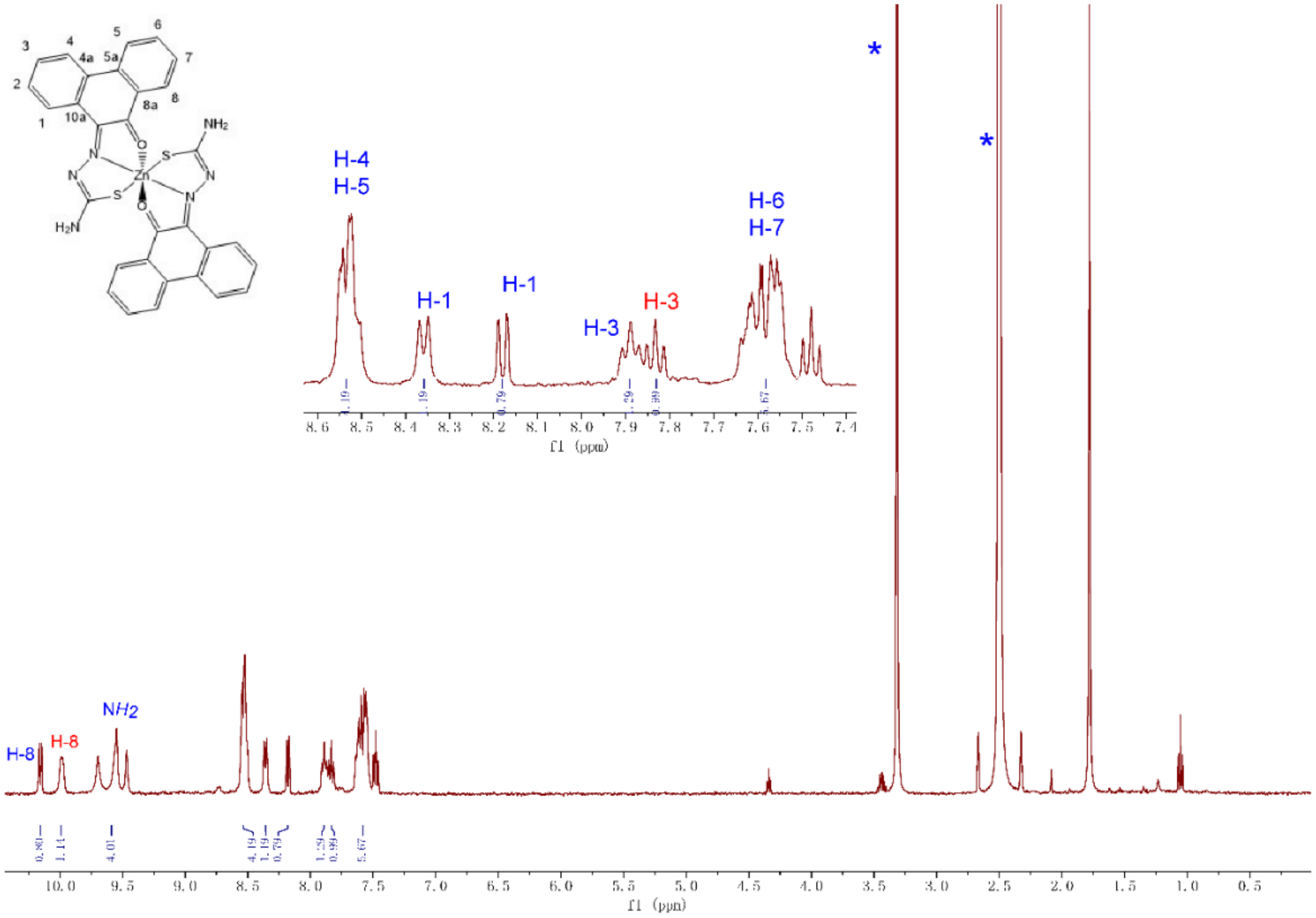

Figure S25 ^{1}H-NMR spectrum (400 MHz, d^6-DMSO) of [**Zn(PH-H)$_2$**], recorded from samples of the compound synthesised by microwave irradiation. (* correspond to the residual solvents, d^6-DMSO and water).

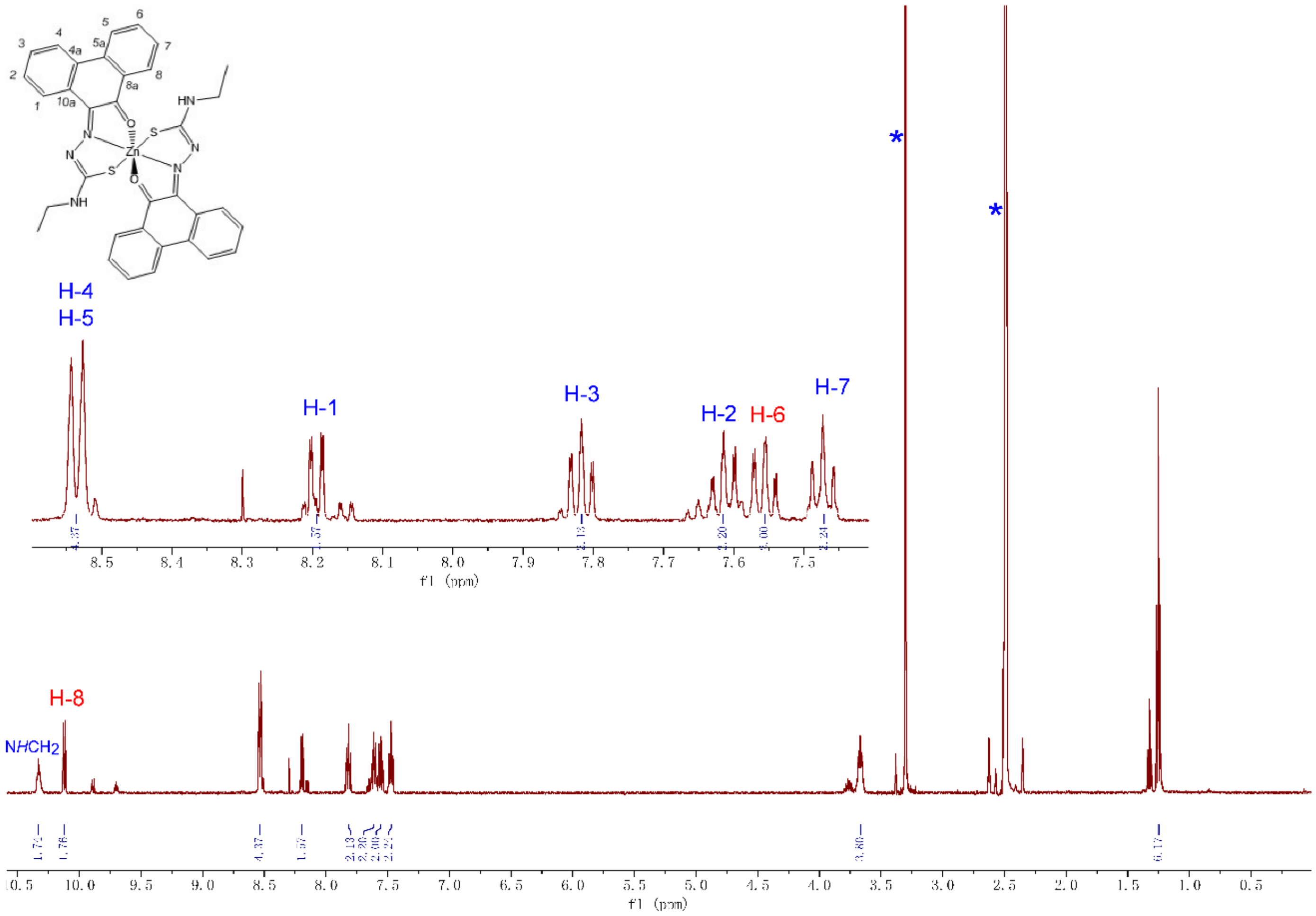

Figure S26 ¹H-NMR spectrum (500 MHz, d⁶-DMSO) of [**Zn(PH-Et)₂**,] recorded from samples of the compound synthesised by microwave irradiation. (* correspond to the residual solvents, d⁶-DMSO and water).

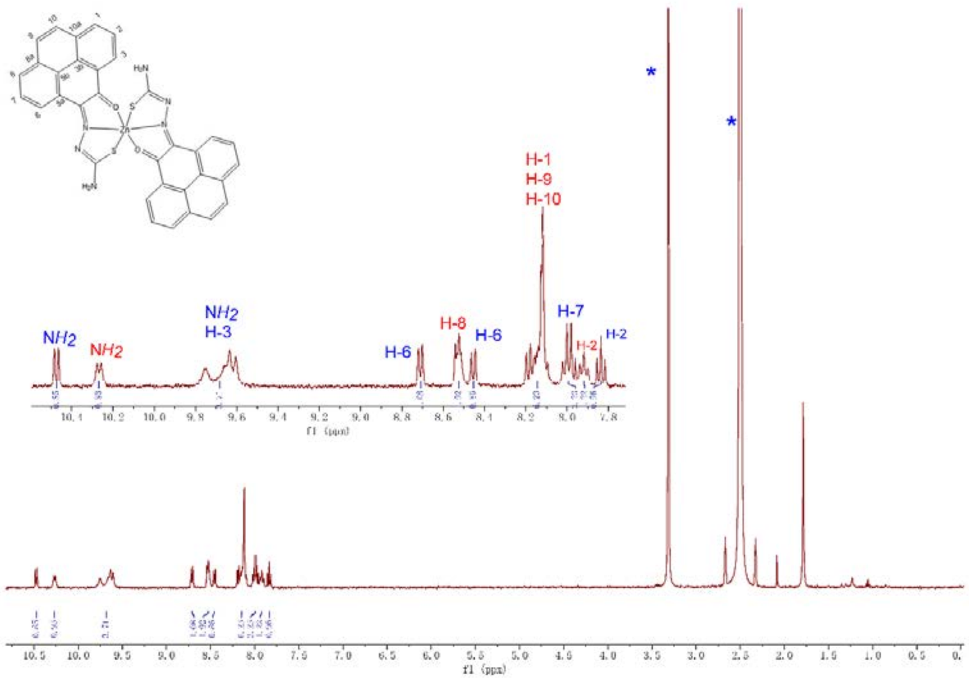

Figure S27 ^{1}H-NMR spectrum (500 MHz, d^{6}-DMSO) of [**Zn(PY-H)$_2$**], recorded from samples of the compound synthesised by microwave irradiation. (* correspond to the residual solvents, d^{6}-DMSO and water).

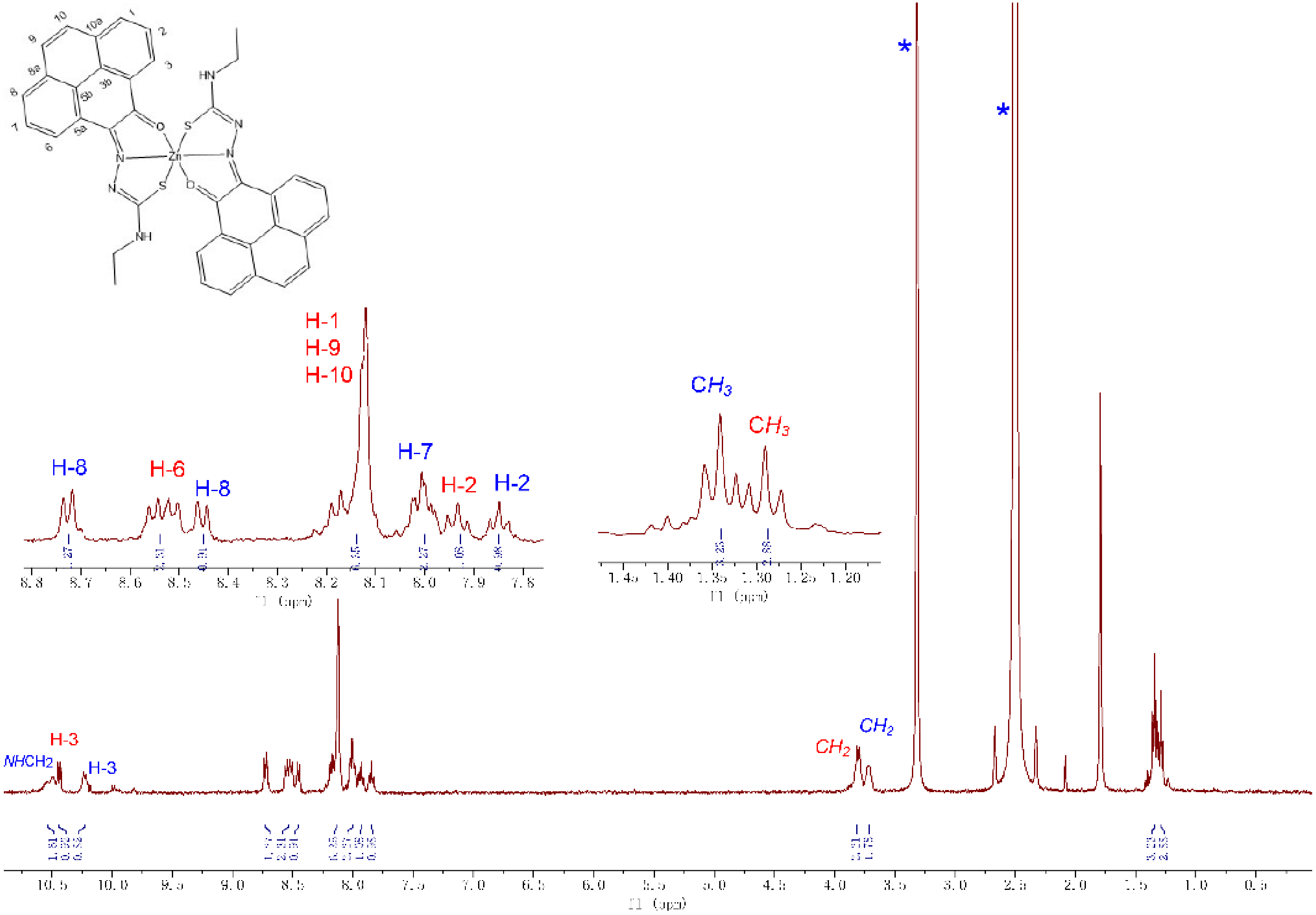

Figure S28 ^{1}H-NMR spectrum (500 MHz, d^6-DMSO) of [**Zn(PY-Et)$_2$**], recorded from samples of the compound synthesised by microwave irradiation. (* correspond to the residual solvents, d^6-DMSO and water).

5 Mass spectrometry for representative ligands and complexes

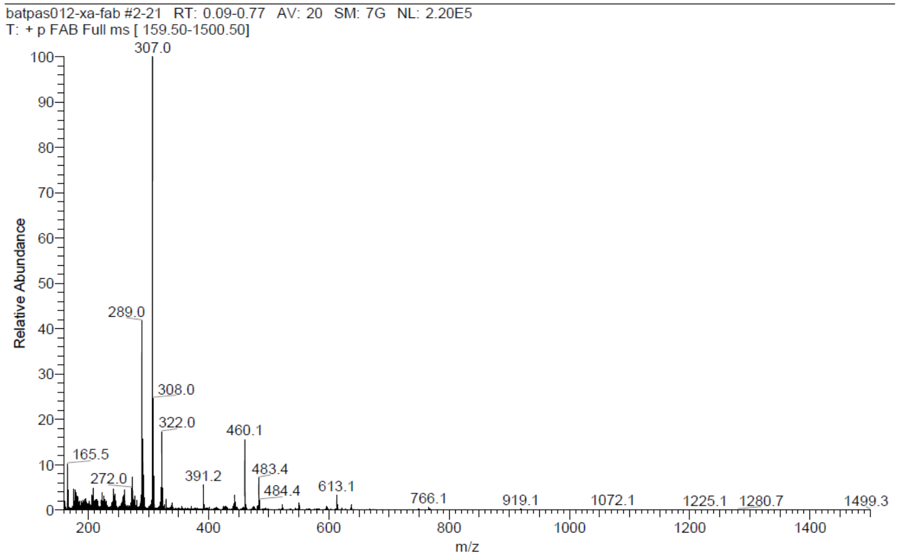

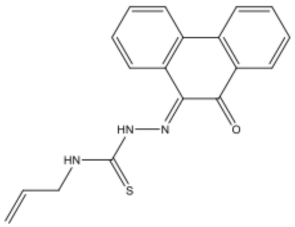

Chemical Formula: $C_{18}H_{15}N_3OS$
Exact Mass: 321.0936
Molecular Weight: 321.3980
m/z: 321.0936 (100.0%), 322.0969 (19.5%), 323.0894 (4.5%), 323.1003 (1.8%), 322.0906 (1.1%)

Chemical Formula: $C_{18}H_{15}N_3NaOS$
Exact Mass: 344.0834
Molecular Weight: 344.3878
m/z: 344.0834 (100.0%), 345.0867 (19.5%), 346.0791 (4.5%), 346.0901 (1.8%), 345.0804 (1.1%)

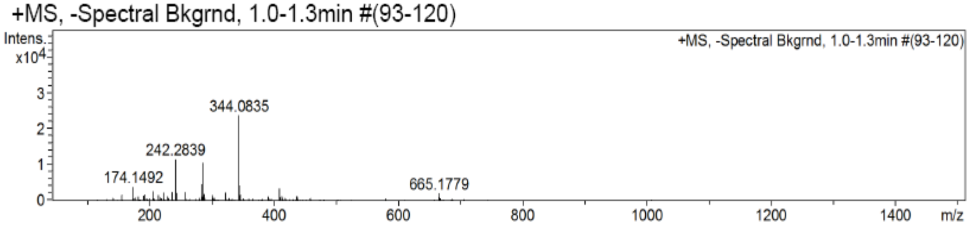

#	m/z	I	I %	Area	S/N
1	174.1492	3602	15.3	68	423.3
2	206.0597	2448	10.4	58	178.5
3	236.1875	2317	9.8	65	111.9
4	242.2839	11436	48.5	177	517.0
5	258.1678	2242	9.5	60	86.9
6	284.2374	4380	18.6	133	137.5
7	287.0255	10449	44.3	271	321.5
8	344.0835	23563	100.0	789	705.7
9	345.0869	4005	17.0	149	119.9
10	409.1628	3204	13.6	126	114.7

Figure S29 ESI^+ mass spectrometry of the PH-Allyl ligand (sample obtained from the conventional heating method).

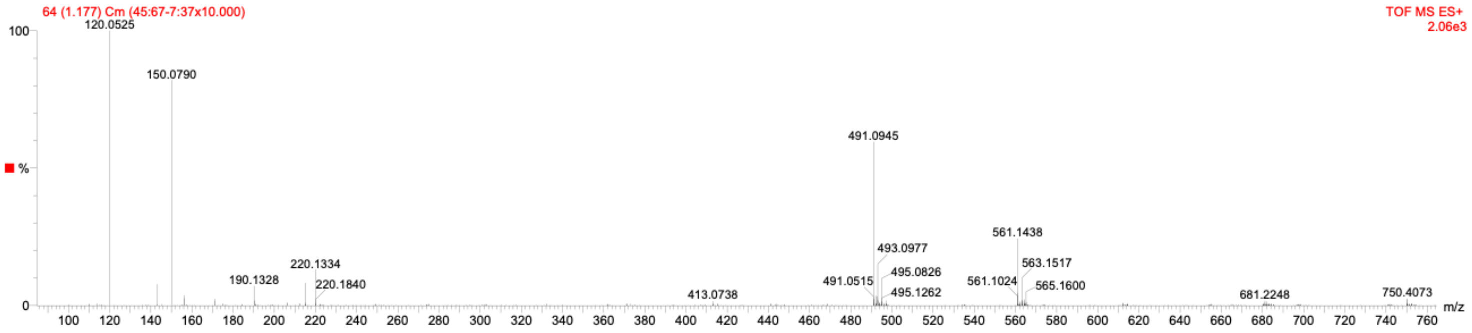

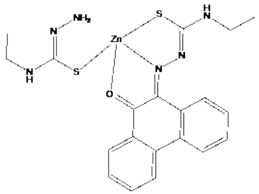

Chemical Formula: $C_{18}H_{22}N_6OS_2Zn$
Exact Mass: 490.0588
Molecular Weight: 491.9570
m/z: 490.0588 (100.0%), 492.0557 (57.4%), 494.0545 (38.6%), 491.0622 (21.6%), 493.0590 (12.4%), 492.0546 (9.0%), 493.0568 (8.4%), 495.0579 (8.9%), 494.0515 (5.2%), 496.0508 (3.5%), 492.0655 (2.2%), 491.0558 (2.2%), 493.0579 (2.0%), 494.0601 (1.8%), 491.0582 (1.6%), 494.0624 (1.3%), 496.0550 (1.9%), 493.0527 (1.3%), 495.0548 (1.1%)

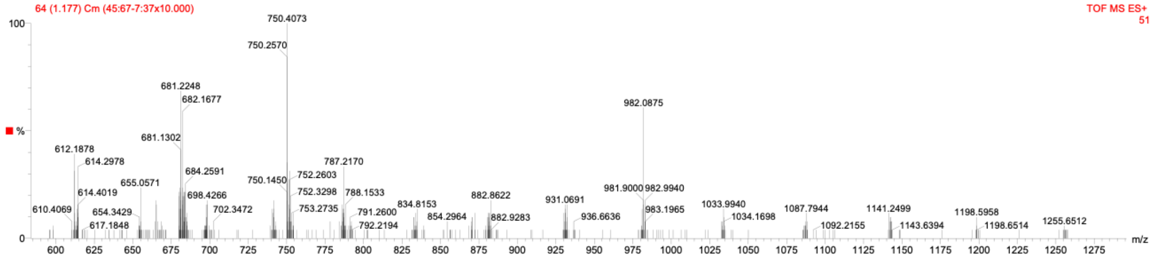

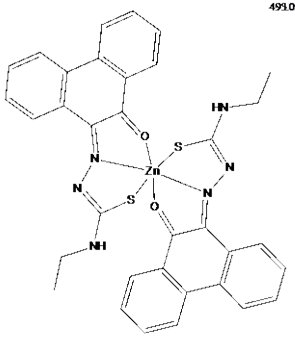

Chemical Formula: $C_{34}H_{28}N_6O_2S_2Zn$
Exact Mass: 680.1007
Molecular Weight: 682.1380
m/z: 680.1007 (100.0%), 682.0976 (57.4%), 684.0964 (38.6%), 681.1040 (36.8%), 683.1009 (21.1%), 685.0997 (14.2%), 682.0965 (9.0%), 683.0986 (8.4%), 682.1074 (6.6%), 684.0933 (5.2%), 684.1043 (3.8%), 686.0922 (3.5%), 683.0998 (3.3%), 684.1020 (3.1%), 686.1031 (2.5%), 681.0977 (2.2%), 685.0967 (1.9%), 681.1000 (1.6%), 686.0968 (1.6%), 687.0955 (1.3%), 683.0946 (1.3%)

Figure S30 Mass spectrometry of $Zn(PH\text{-}Ethyl)_2$ complex obtained from PH-Ethyl and $Zn(OAc)_2$ (2:1 ratio) resulting from protocols conducted entirely under conventional heating.

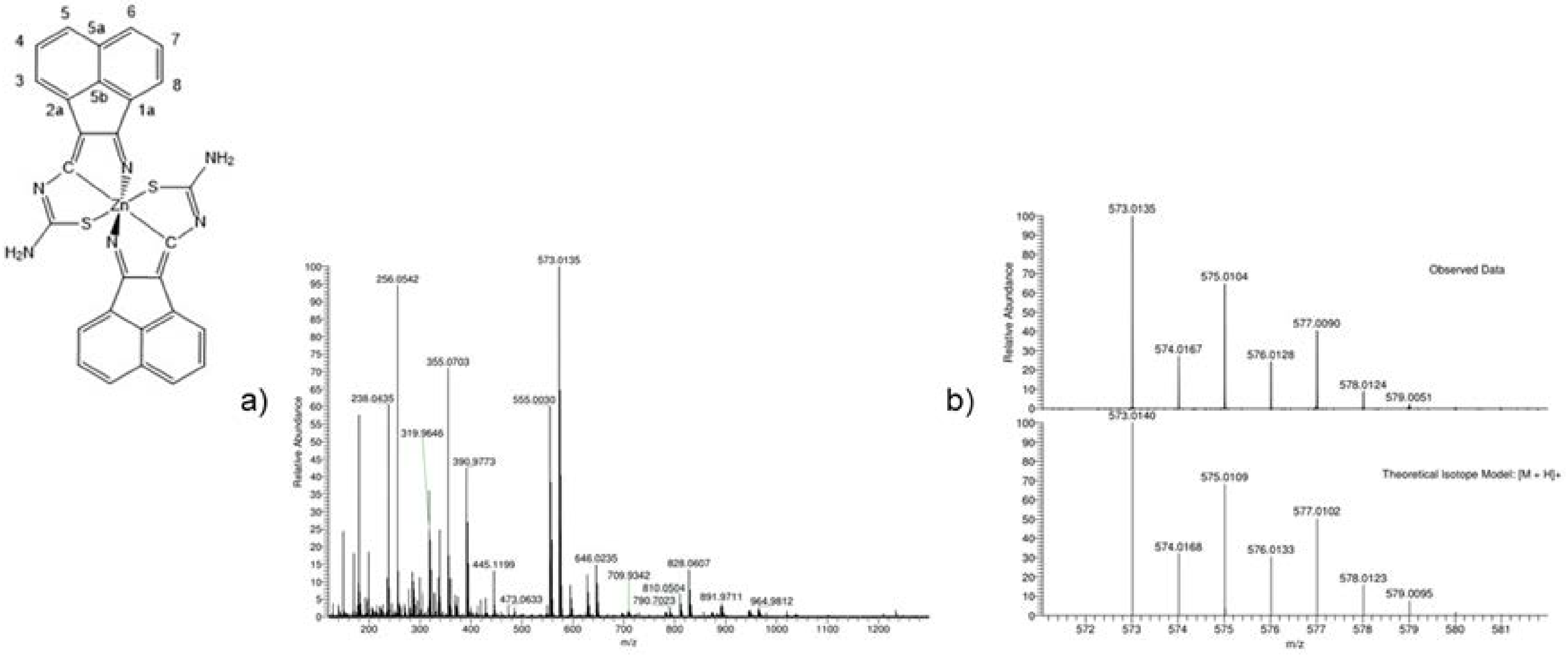

Figure S31 NSI-MS of compound **Zn(AN-H)₂**. a) full spectra, b) top row shows the observed data of the isotopic model of $[M+H]^+$, the bottom row shows the theoretical isotope model of $[M+H]^+$. (recorded from samples of the compound synthesised by microwave irradiation).

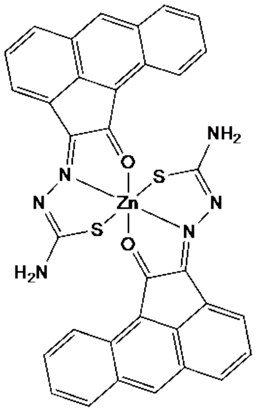

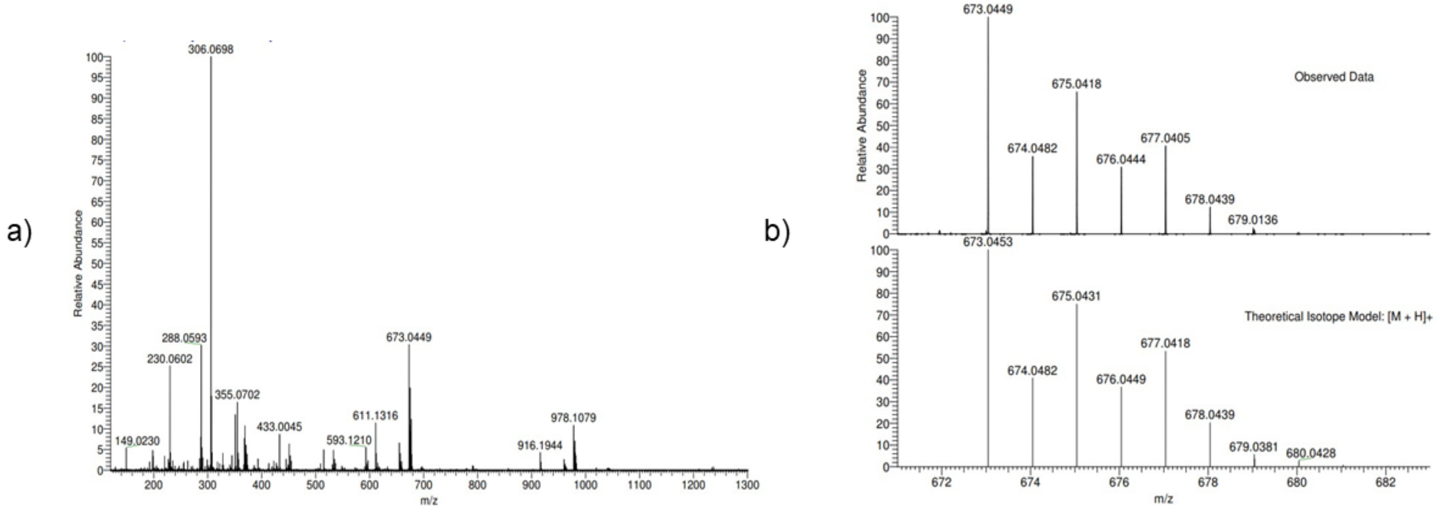

Figure S32 NSI-MS of compound [**Zn(AA-H)₂**] recorded from samples of the compound synthesised by microwave irradiation, 1: 1 ratio. a) full spectra, b) top row shows the observed data of the isotopic model of $[M+H]^+$, the bottom row shows the theoretical isotopic model of $[M+H]^+$.

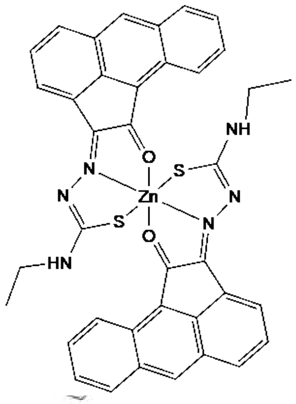

a)

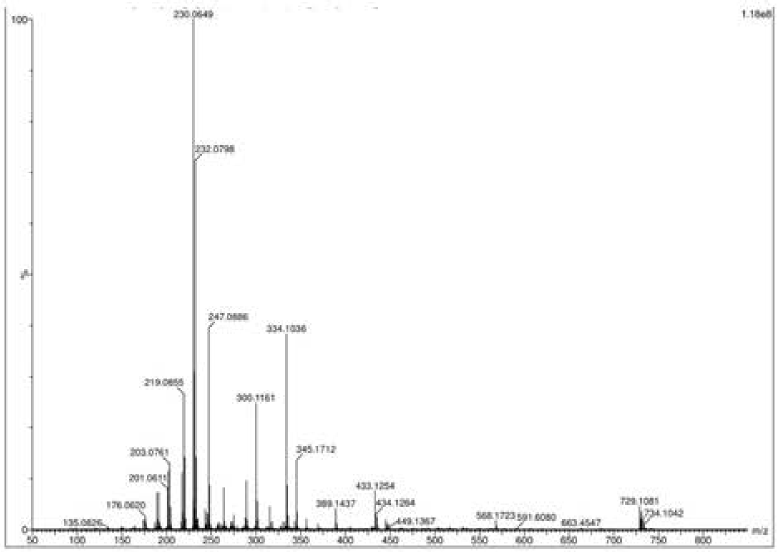

b)

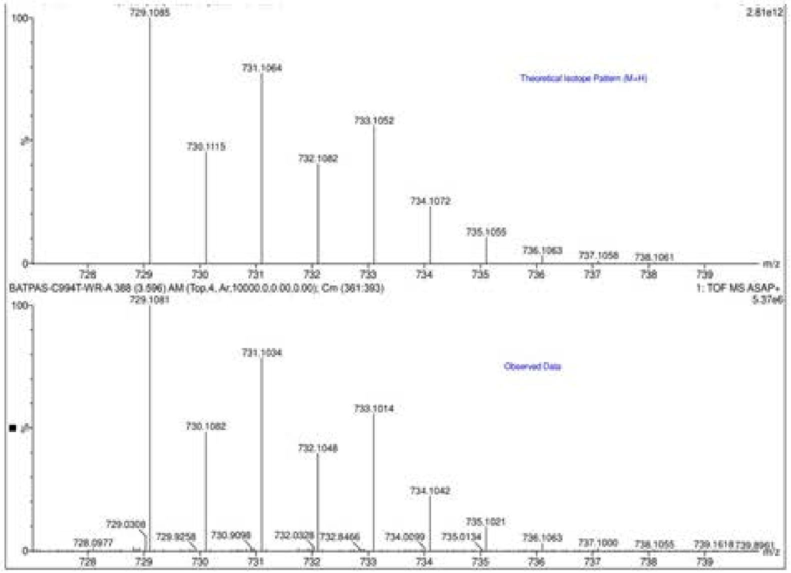

Figure S33 ASAP-MS of compound [**Zn(AA-Et)₂**]. a) full spectra, b) top row shows the observed data of the isotopic model of [M+H]$^+$, the bottom row shows the theoretical isotopic model of [M+H]$^+$. Recorded from samples of the compound synthesised by microwave irradiation, in 1:1 ratio.

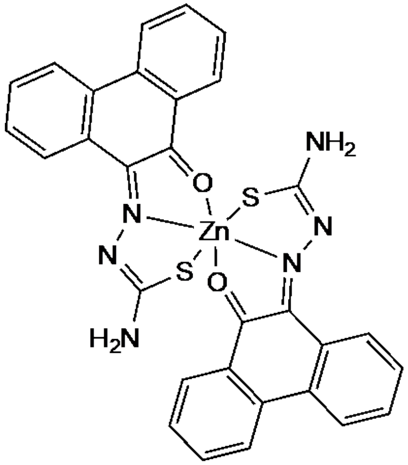

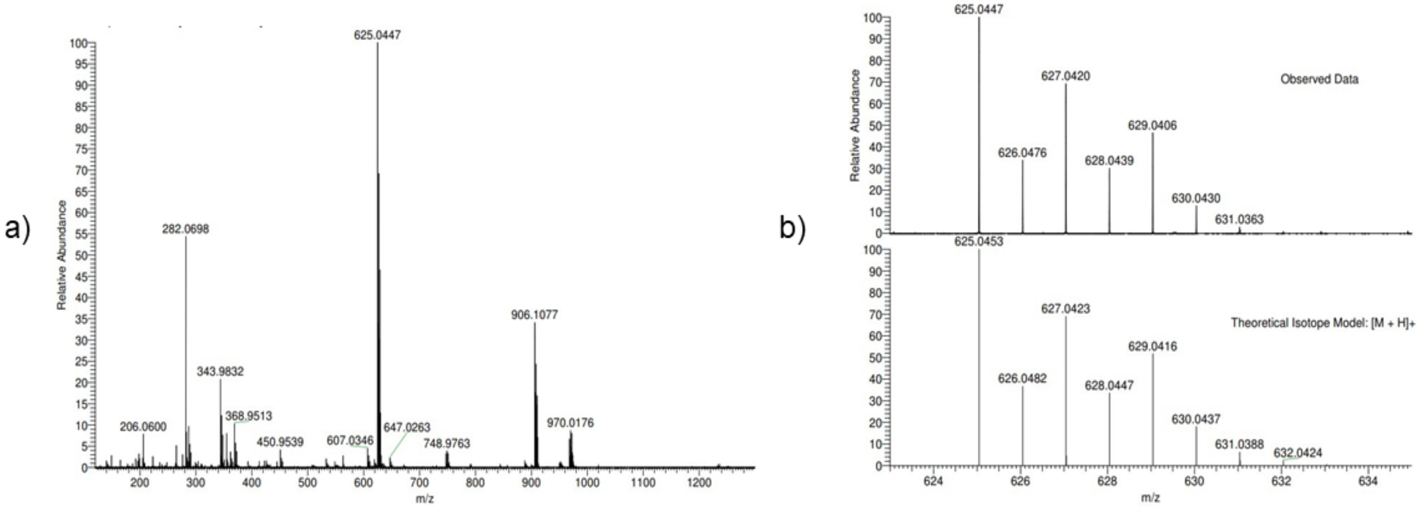

Figure S34 NSI-MS of compound [**Zn(PH-H)2**]. a) full spectra, b) top row shows the observed data of the isotopic model of $[M+H]^+$, the bottom row shows the theoretical isotopic model of $[M+H]^+$. Recorded from samples of the compound synthesised by microwave irradiation, in 1:1 ratio.

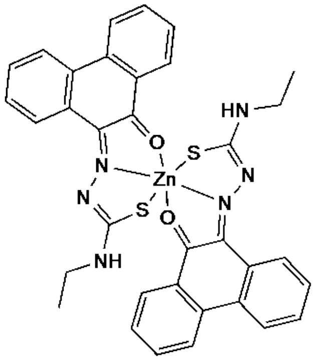

a)

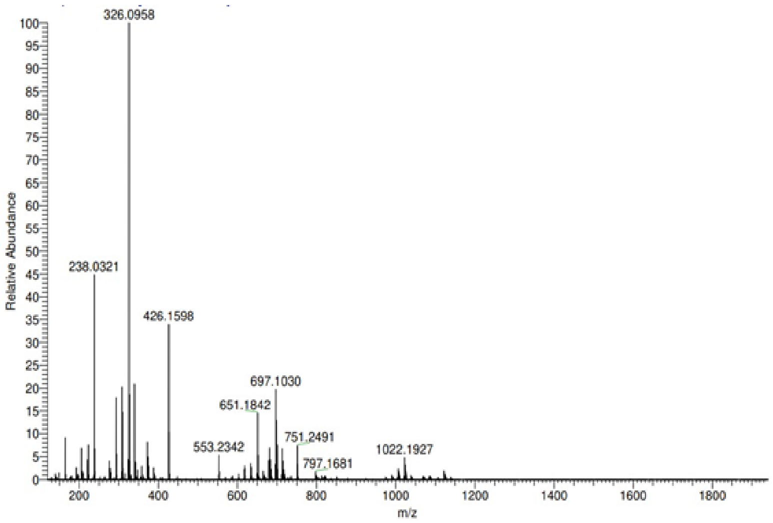

b

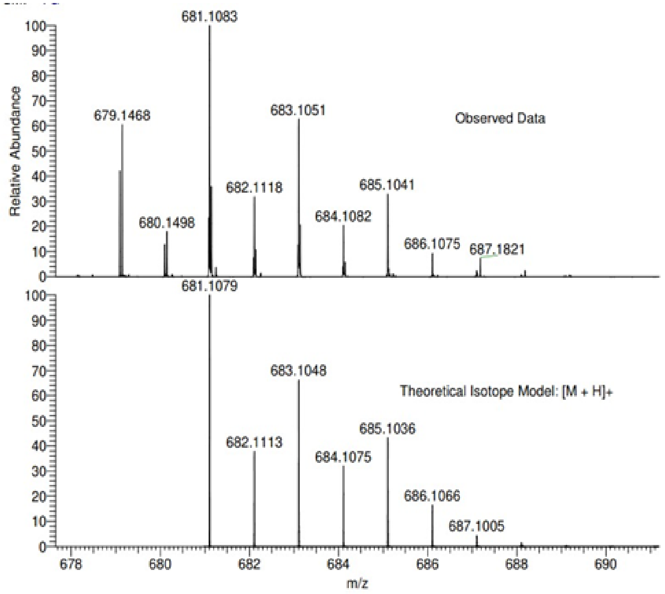

Figure S35 NSI-MS of compound [**Zn(PH-Et)₂**] a) full spectra, b) the top row shows the observed data of the isotopic model of $[M+H]^+$, the bottom row shows the theoretical isotopic model of $[M+H]^+$. Recorded from samples of the compound synthesised by microwave irradiation, in 1:1 ratio.

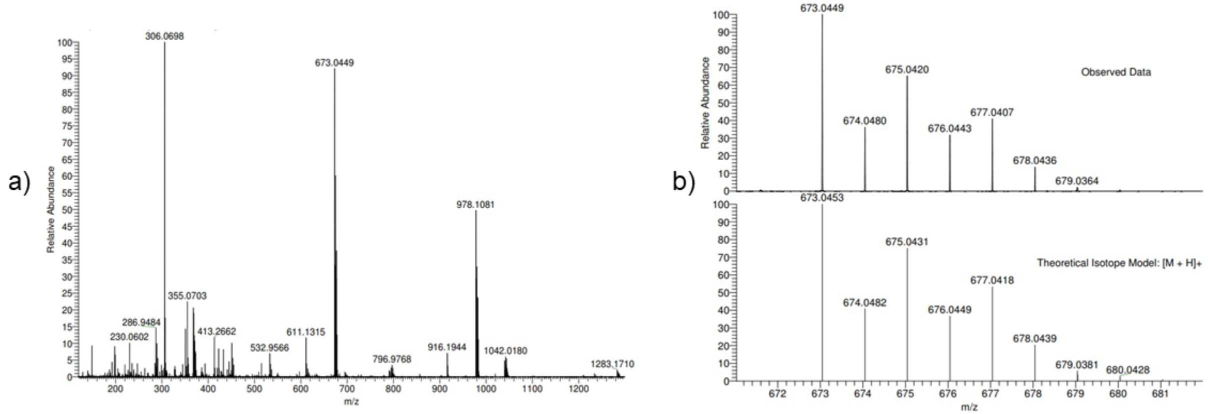

Figure S36 NSI-MS of compound [**Zn(PY-H)$_2$**]. a) full spectra, b) top row shows the observed data of the isotope model of $[M+H]^+$, the bottom row shows the theoretical isotope model of $[M+H]^+$. Recorded from samples of the compound synthesised by microwave irradiation, in 1:1 ratio.

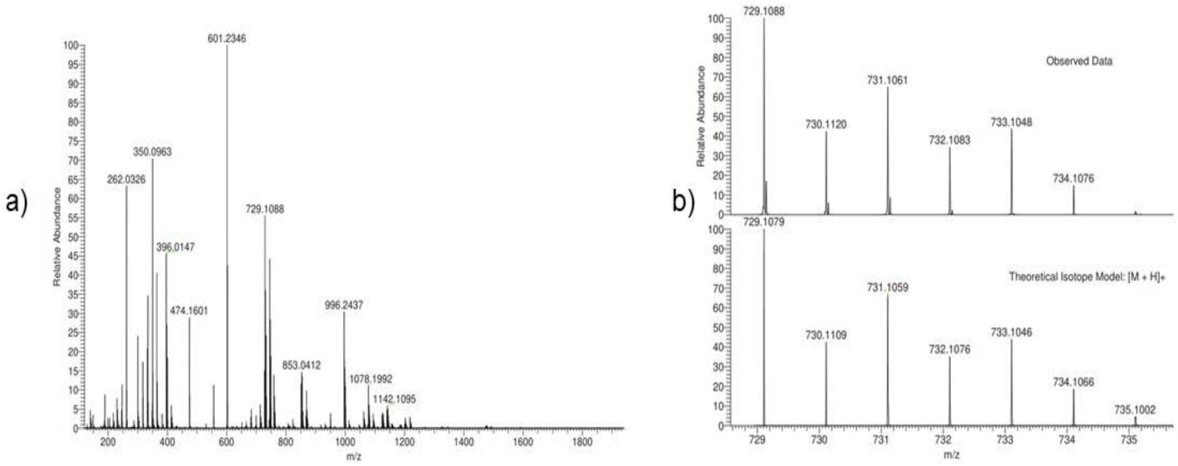

Figure S37 NSI-MS of compound [**Zn(PY-Et)₂**]. a) full spectra, b) top row shows the observed data of the isotope model of $[M+H]^+$, the bottom row shows the theoretical isotope model of $[M+H]^+$. Recorded from samples of the compound synthesised by microwave irradiation, in 1:1 ratio.

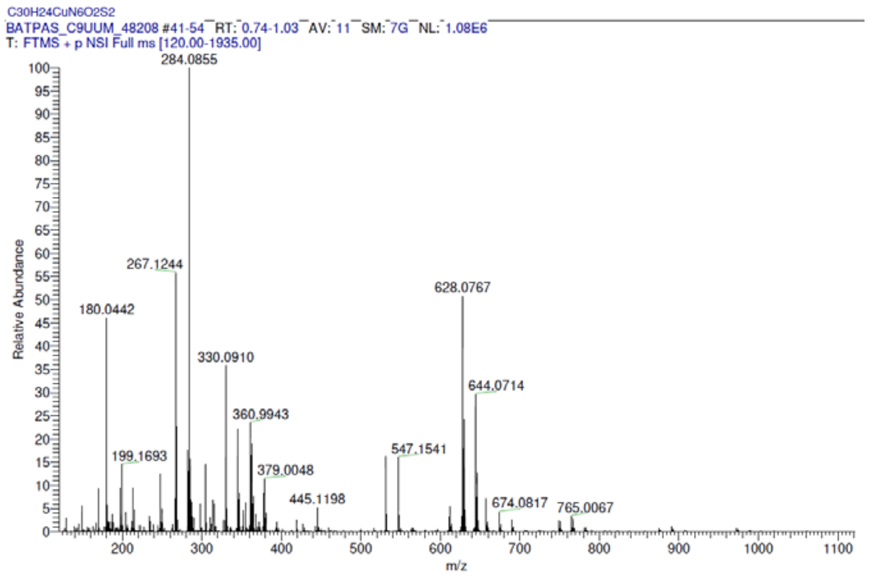

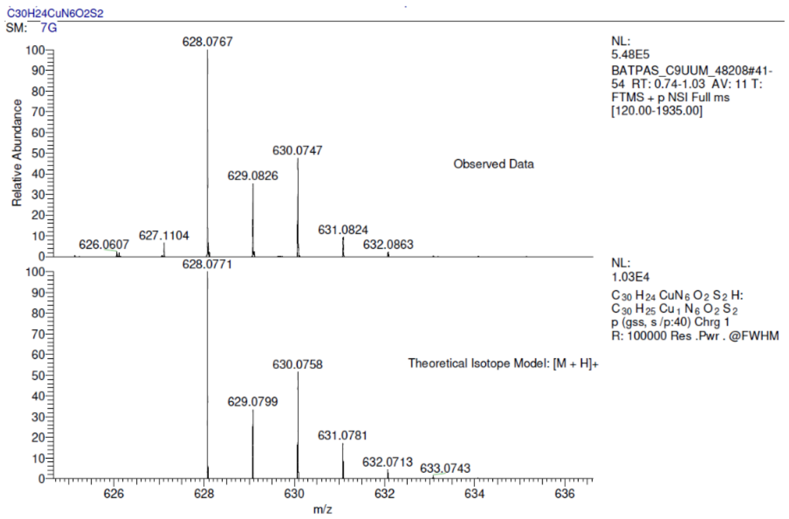

(a) **(b)**

Figure S38 HR MS (NSI-MS) of compound [**Cu(AN-Et)$_2$**]. a) full spectra, b) Top row shows the observed data of the isotope model of $[M+H]^+$, the bottom row shows the theoretical isotope model of $[M+H]^+$. Recorded from samples of the compound synthesised by microwave irradiation (1:1 ligand : metal precursor anhydrous $Cu(OAc)_2$ reaction, 2 min stirring at r.t. followed by 90 min microwave irradiation).

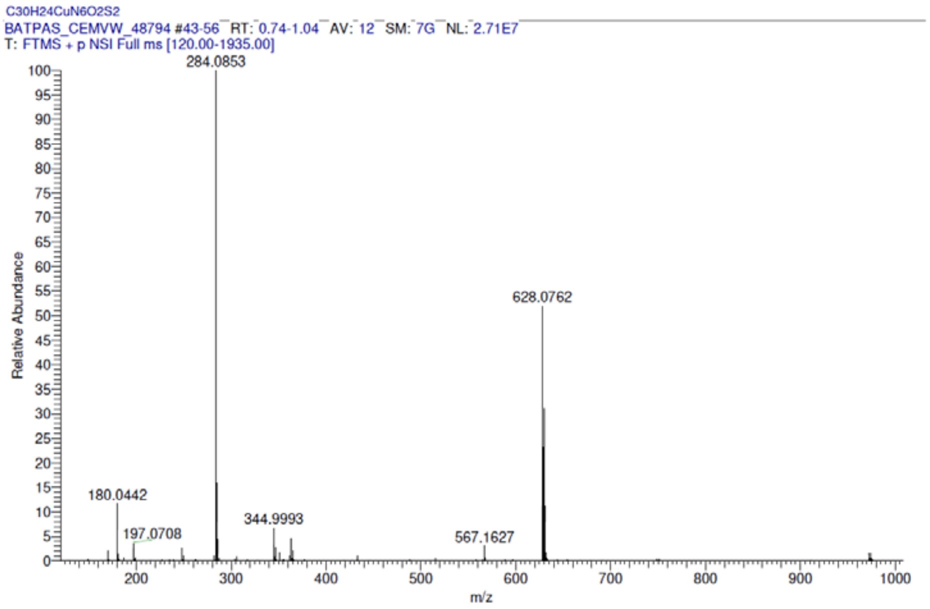

(a)

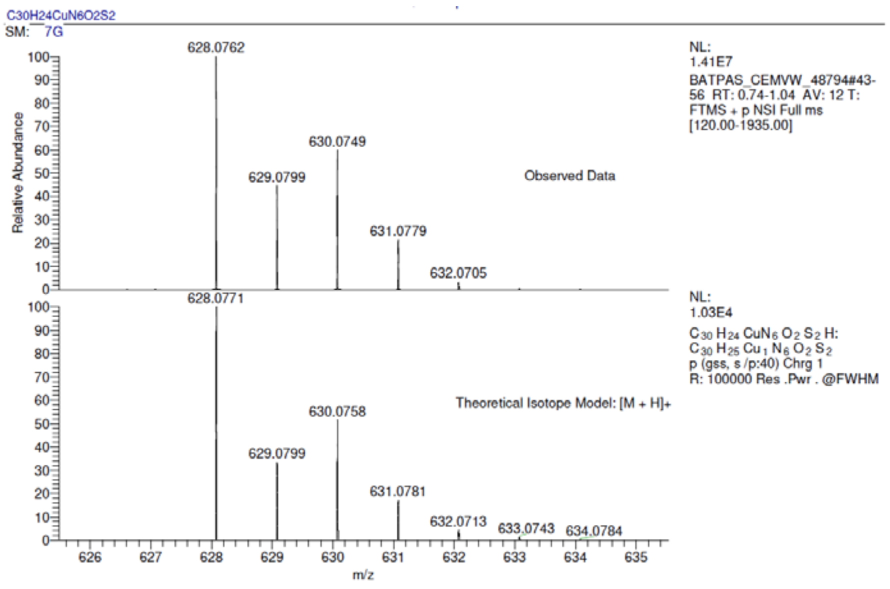

(b)

Figure S39 HR MS (NSI-MS) of compound [**Cu(AN-Et)₂**]. a) full spectra, b) top row shows the observed data of the isotope model of $[M+H]^+$, the bottom row shows the theoretical isotope model of $[M+H]^+$. Recorded from samples of the compound synthesised by microwave irradiation (2:1 ligand : metal precursor anh. $Cu(OAc)_2$ reaction, 2 min stirring at r.t. followed by 90 min microwave irradiation).

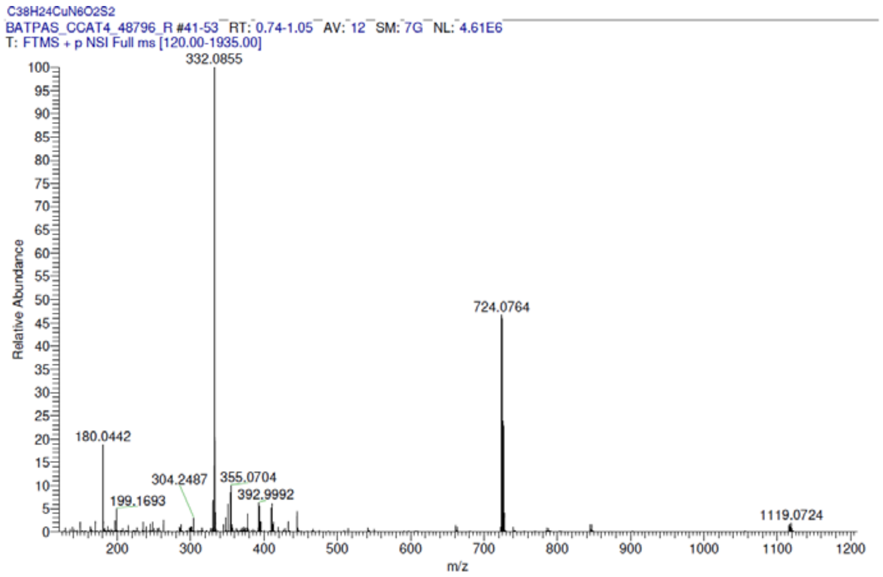

(a)

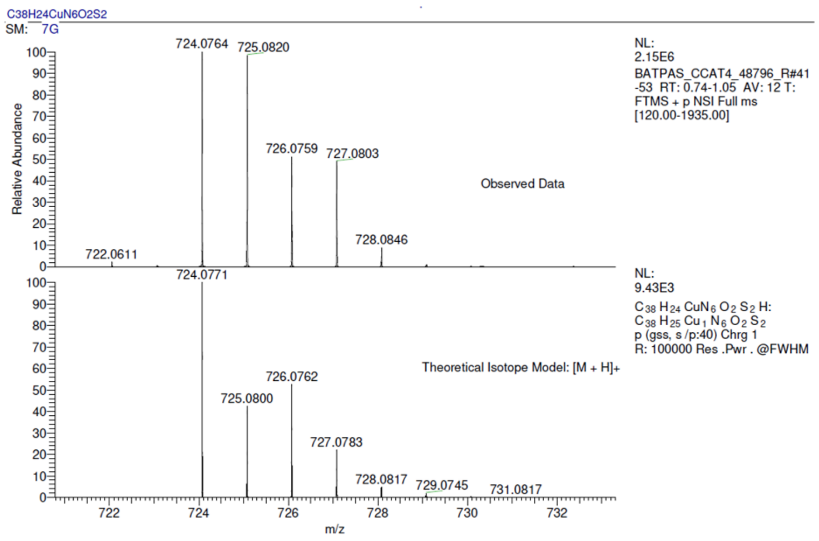

(b)

Figure S40 HR MS (NSI-MS) of compound [**Cu(AN-Ph)$_2$**]. a) full spectra, b) top row shows the observed data of the isotope model of $[M+H]^+$, the bottom row shows the theoretical isotope model of $[M+H]^+$. Recorded from samples of the compound synthesised by microwave irradiation (2:1 ligand : metal precursor anh. $Cu(OAc)_2$ reaction, 2 min stirring at r.t. followed by 90 min microwave irradiation).

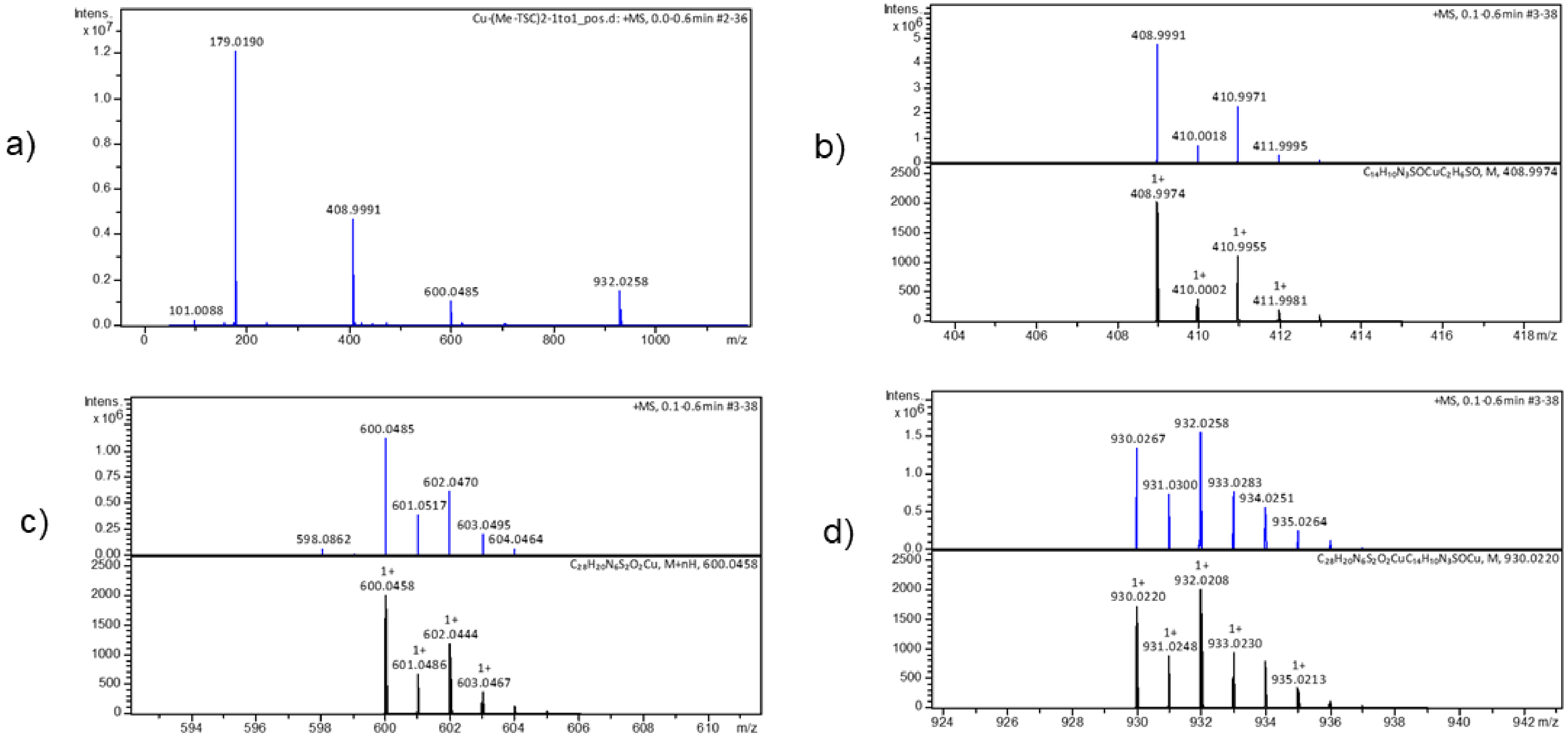

Figure S41 Mass spectrometry (ESI-MS$^+$) results of Cu(II) complex of AN-Me ligand (HL), resulting from the r.t. reaction where the 1:1 ratio of metal:ligand was used. a) full spectra, b) $[L+Cu+DMSO]^+$, c) $[CuL_2+H]^+$, d) $[CuL_2+L+Cu]^+$. Background Contamination Ions: $[(DMSO)_2+Na]^+$: 179.0171.

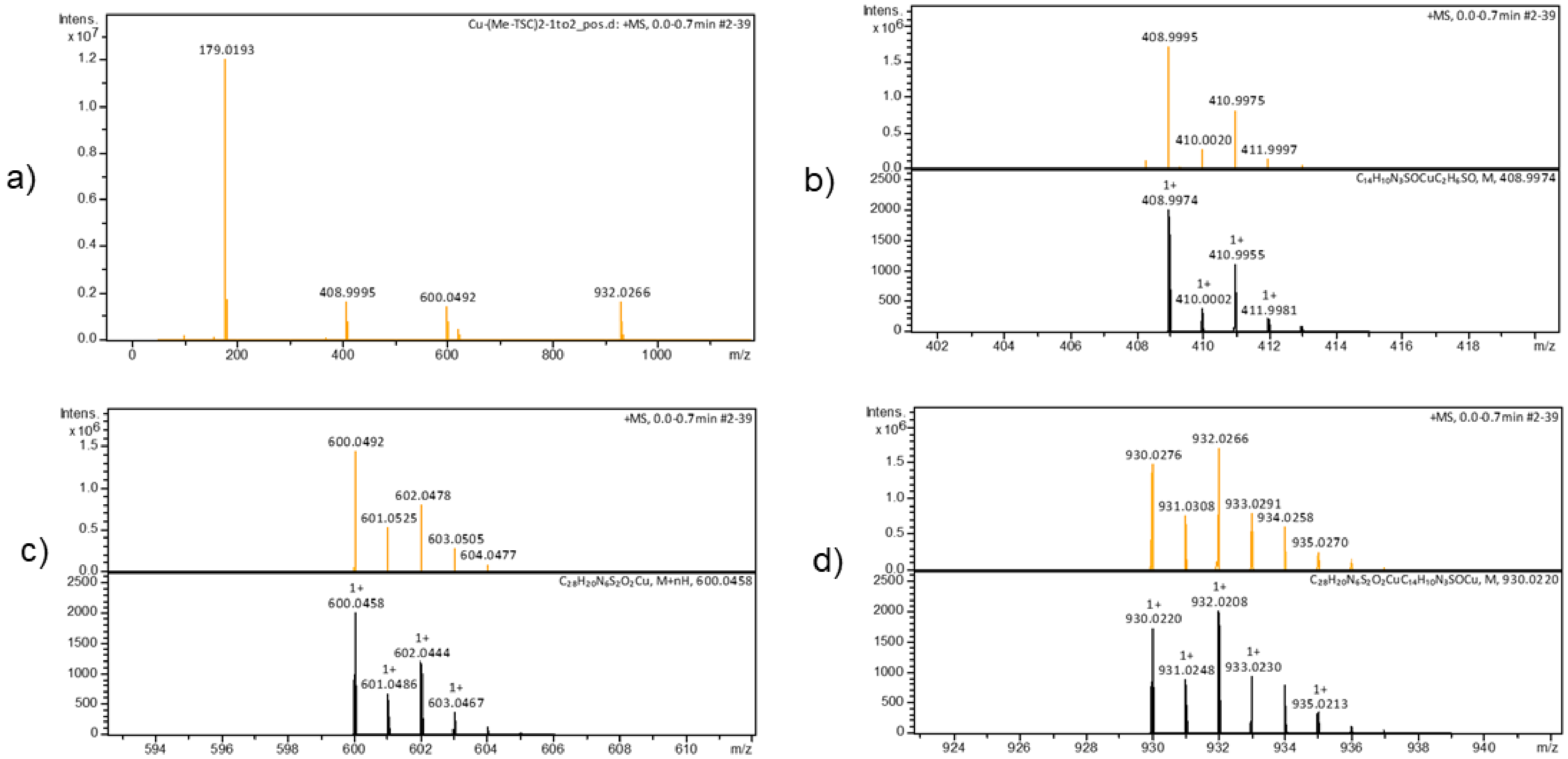

Figure S42 Mass spectrometry (ESI$^+$) of Cu(AN-Me)$_2$ resulting from the resulting from the r.t. reaction reaction where the 1:2 ratio of metal:ligand was used. a) full spectra, b) [L+Cu+DMSO]$^+$, c) [CuL_2+H]$^+$, d) [CuL_2+L+Cu]$^+$.

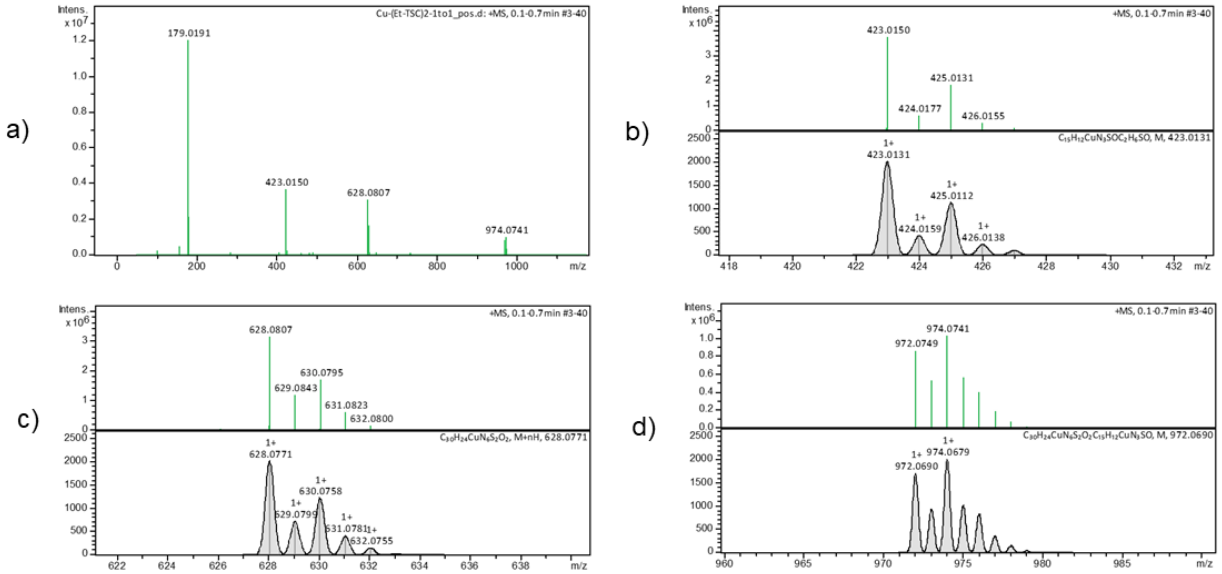

Figure S43 Mass spectrometry results of of Cu(II) complex of AN-Et ligand (HL), resulting from the reaction where the 1:1 ratio of metal:ligand was used. a) full spectra, b) $[L+Cu+DMSO]^+$, c) $[CuL_2+H]^+$, d) $[CuL_2+L+Cu]^+$.

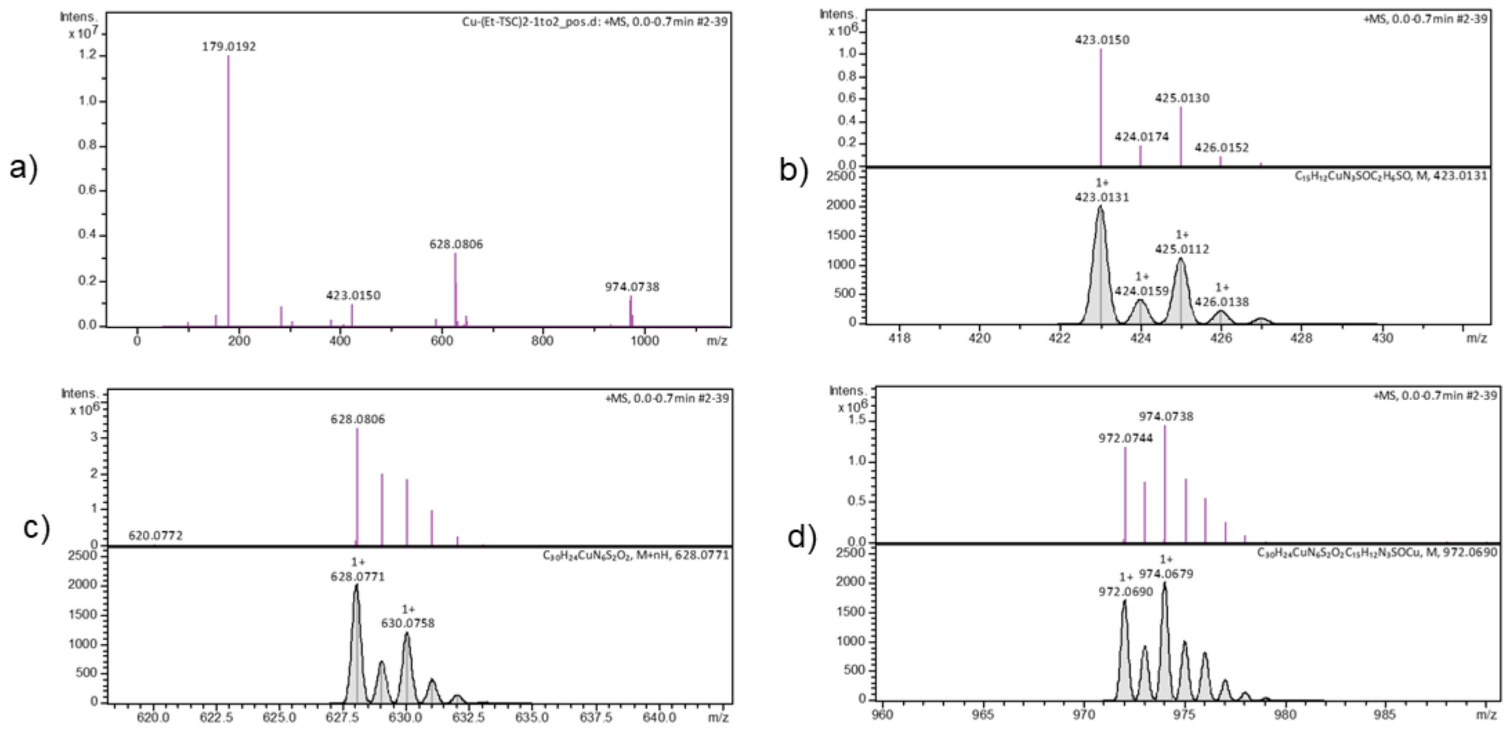

Figure S44 Mass spectrometry (ESI^+) results of Cu(AN-Et)$_2$ -sample resulting from the 1:2 ratio reaction of Cu(II) and HL at the r.t. a) full spectra, b) $[L+Cu+DMSO]^+$, c) $[CuL_2+H]^+$, d) $[CuL_2+L+Cu]^+$.

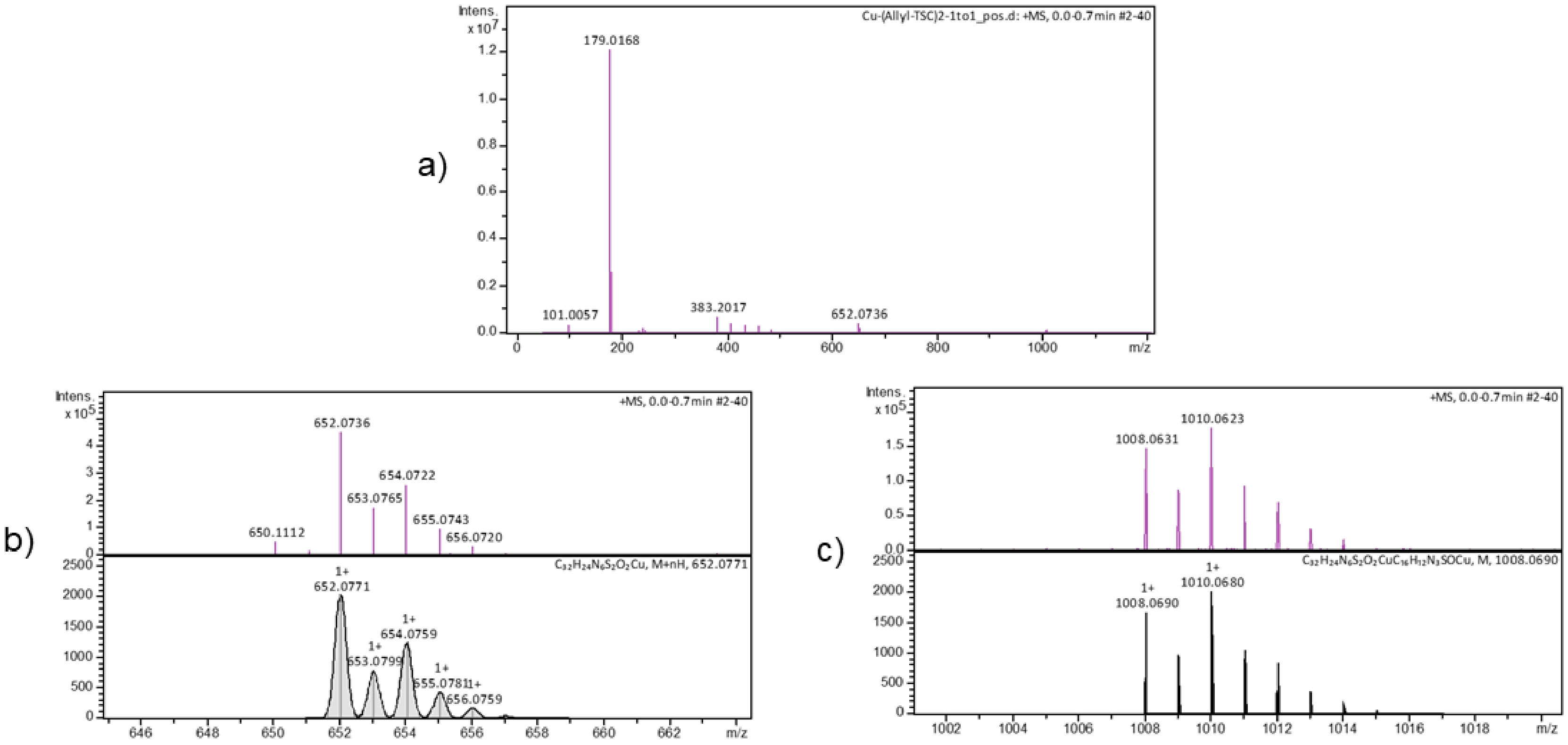

Figure S45 Mass spectrometry (ESI$^+$) results of Cu(II) complex of AN-Allyl ligand (HL), resulting from the r.t reaction where the 1:1 ratio of metal:ligand was used. a) full spectra, b) $[CuL_2+H]^+$, c) $[CuL_2+L+Cu]^+$.

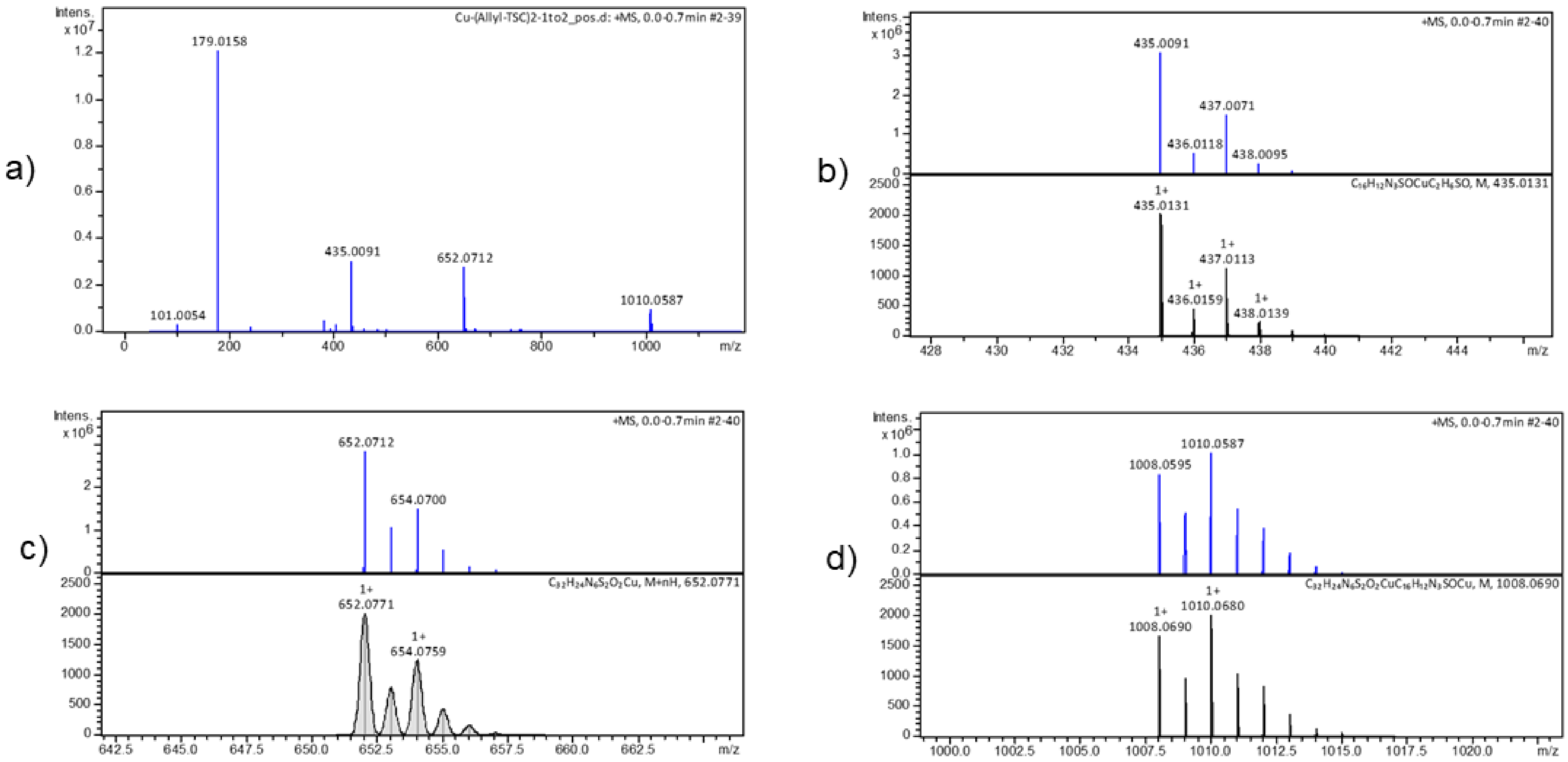

Figure S46 Mass spectrometry (ESI$^+$) results of [Cu(AN-Allyl)$_2$]. Sample resulting from the r.t reaction where the 1:2 ratio of Cu(OAc)$_2$ and HL was used. a) full spectra, b) [L+Cu+DMSO]$^+$, c) [CuL$_2$+H]$^+$, d) [CuL$_2$+L+Cu]$^+$.

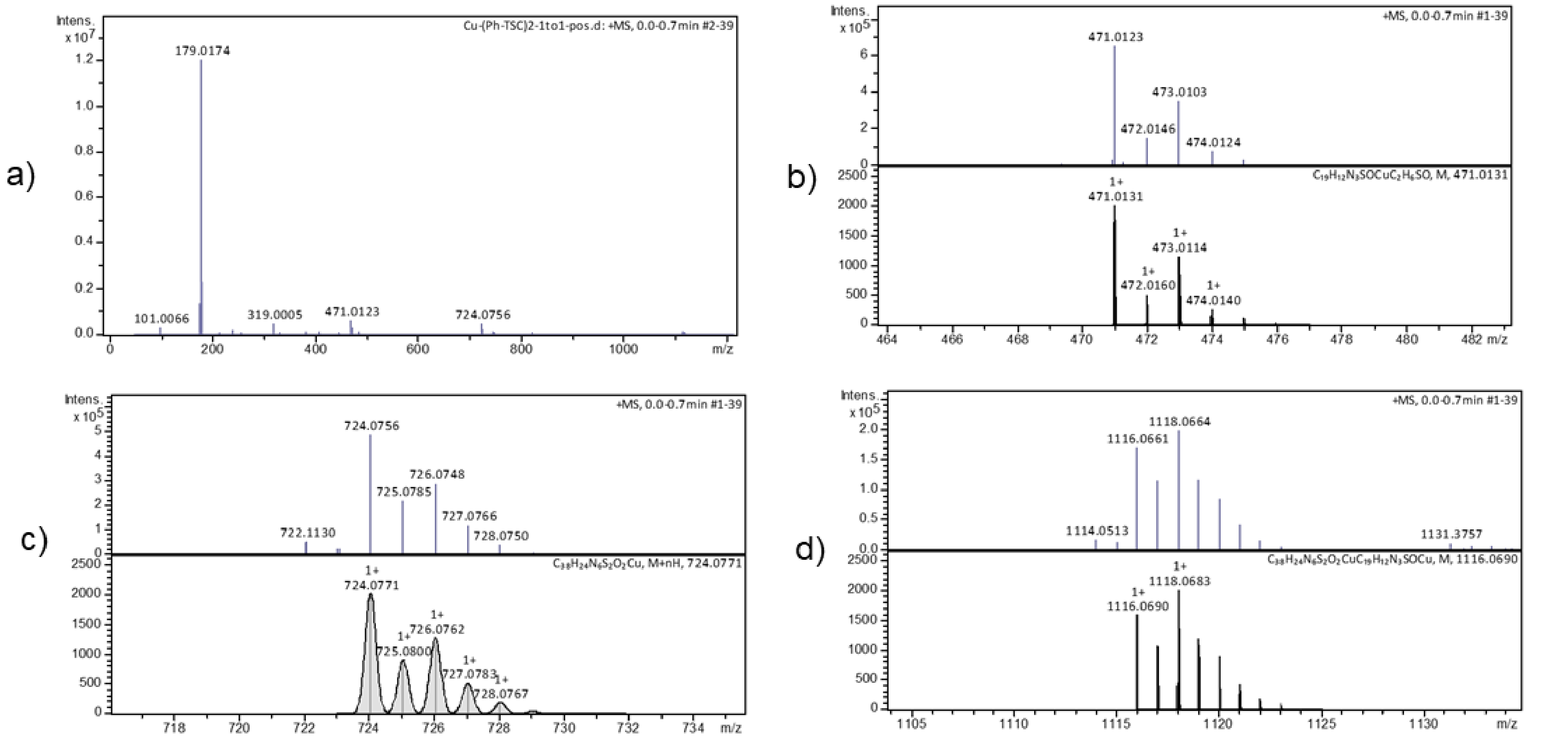

Figure S47 Mass spectrometry (ESI+) results of Cu(II) complex of AN-Ph ligand (HL), resulting from the r.t. reaction where the 1:1 ratio of metal:ligand was used. a) full spectra, b) $[L+Cu+DMSO]^+$, c) $[CuL_2+H]^+$, d) $[CuL_2+L+Cu]^+$.

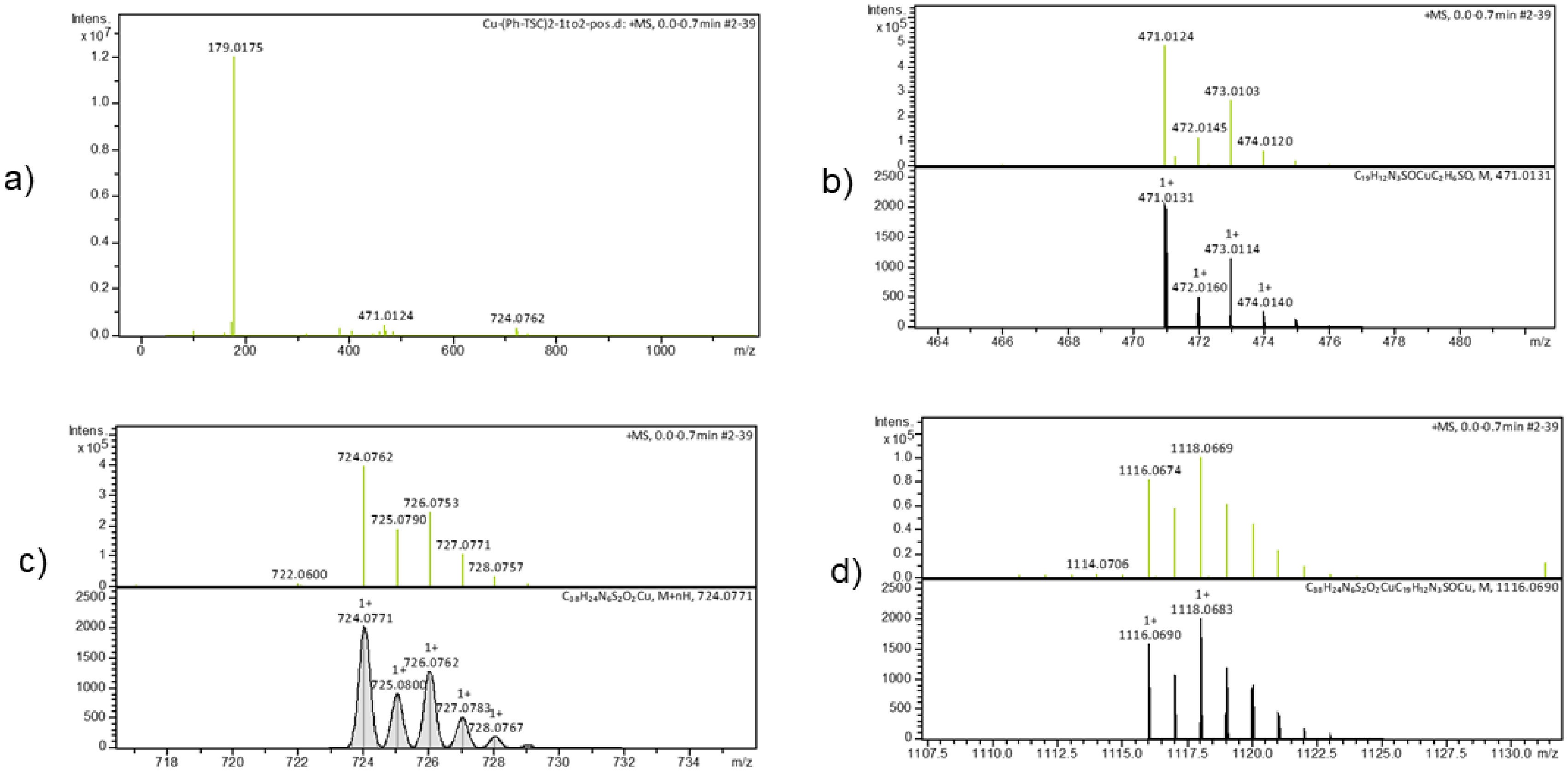

Figure S48 Mass spectrometry results of Cu(AN-Ph)$_2$ from the 1:2 ratio reaction in positive mode. a) full spectra, b) [L+M+DMSO]$^+$, c) [ML$_2$+H]$^+$, d) [ML$_2$+L+M]$^+$.

6 Infrared spectroscopy for selected compounds

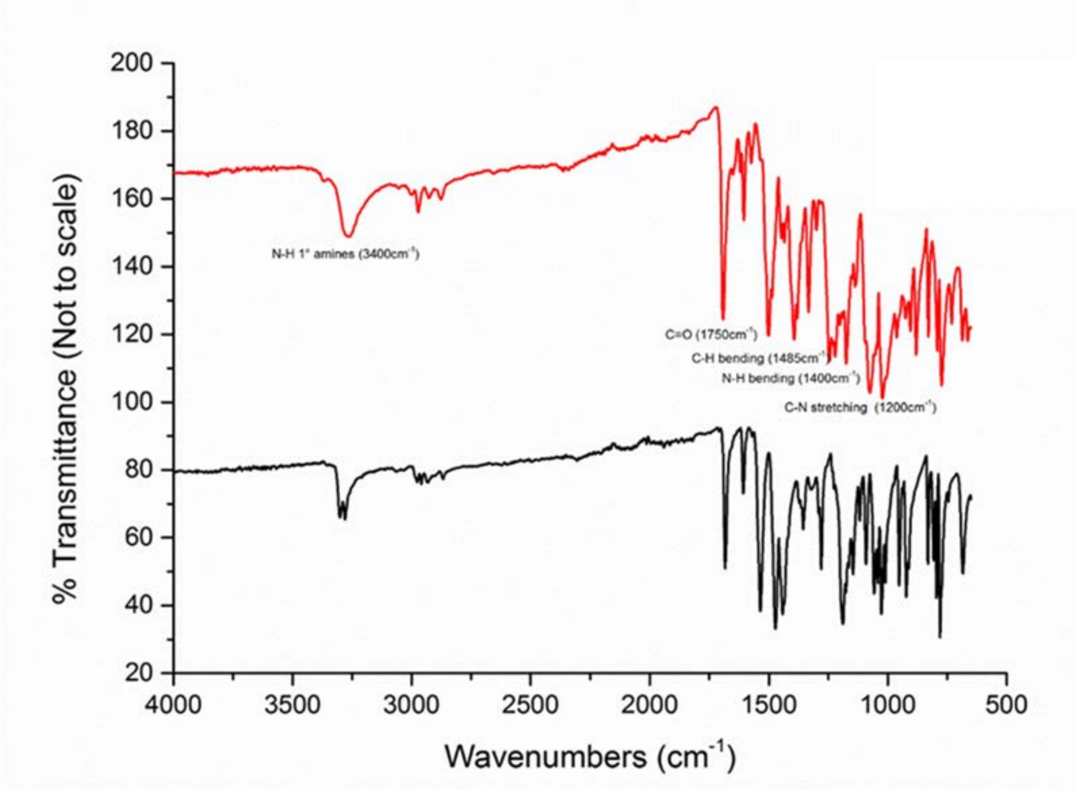

Figure S49 IR spectra for **AA-Et** (black) and [**Zn(AA-Et)$_2$**] (red).

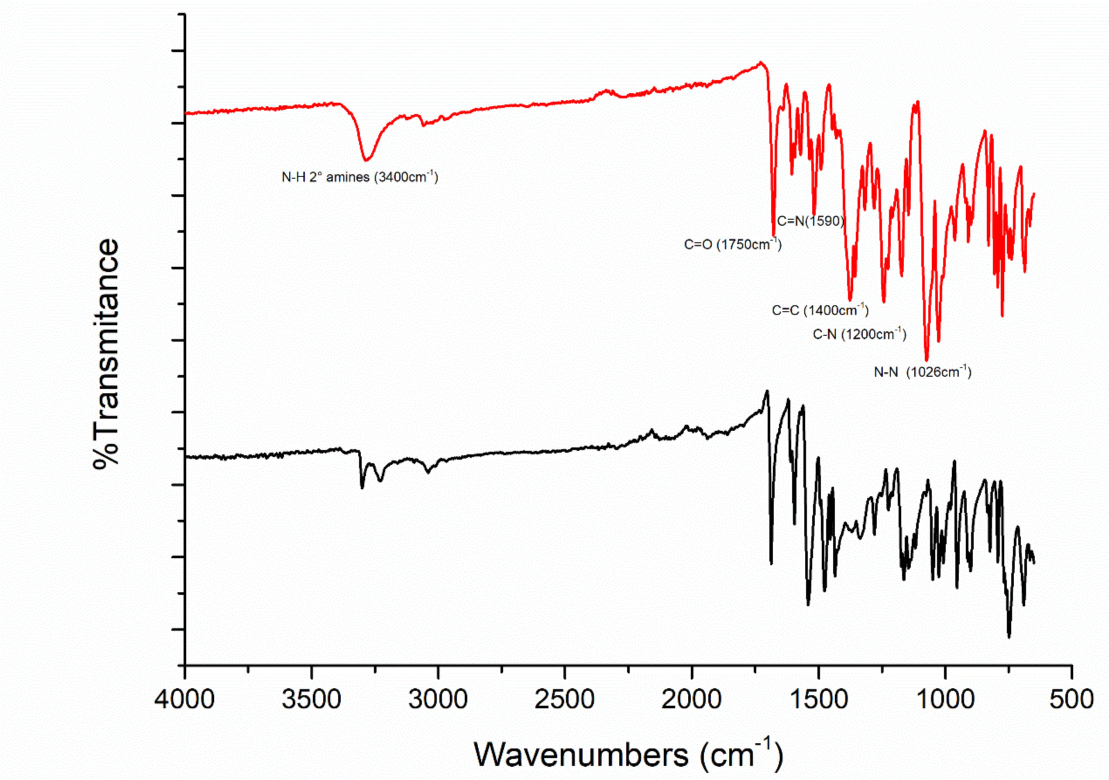

Figure S50 IR spectra for **AN-Ph ligand** (black) and [**Zn(AN-Ph)$_2$**] (red).

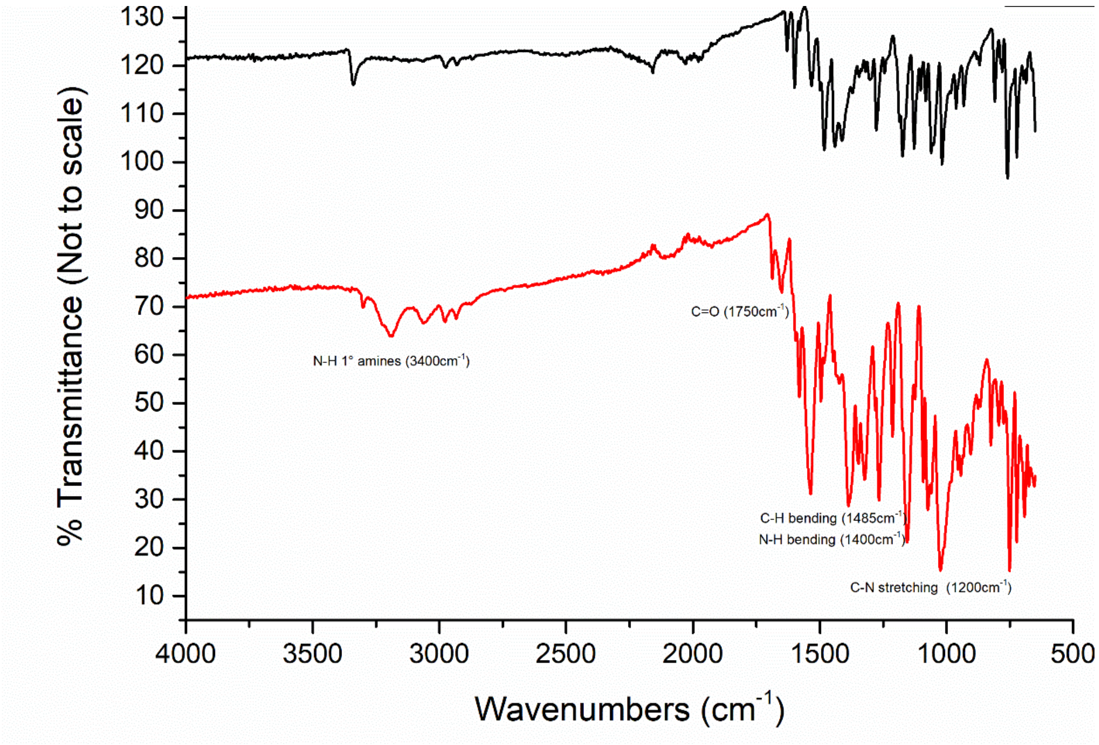

Figure S51. IR spectra for **PH-Et ligand** (black) and [**Zn(PH-Et)$_2$**] (red).

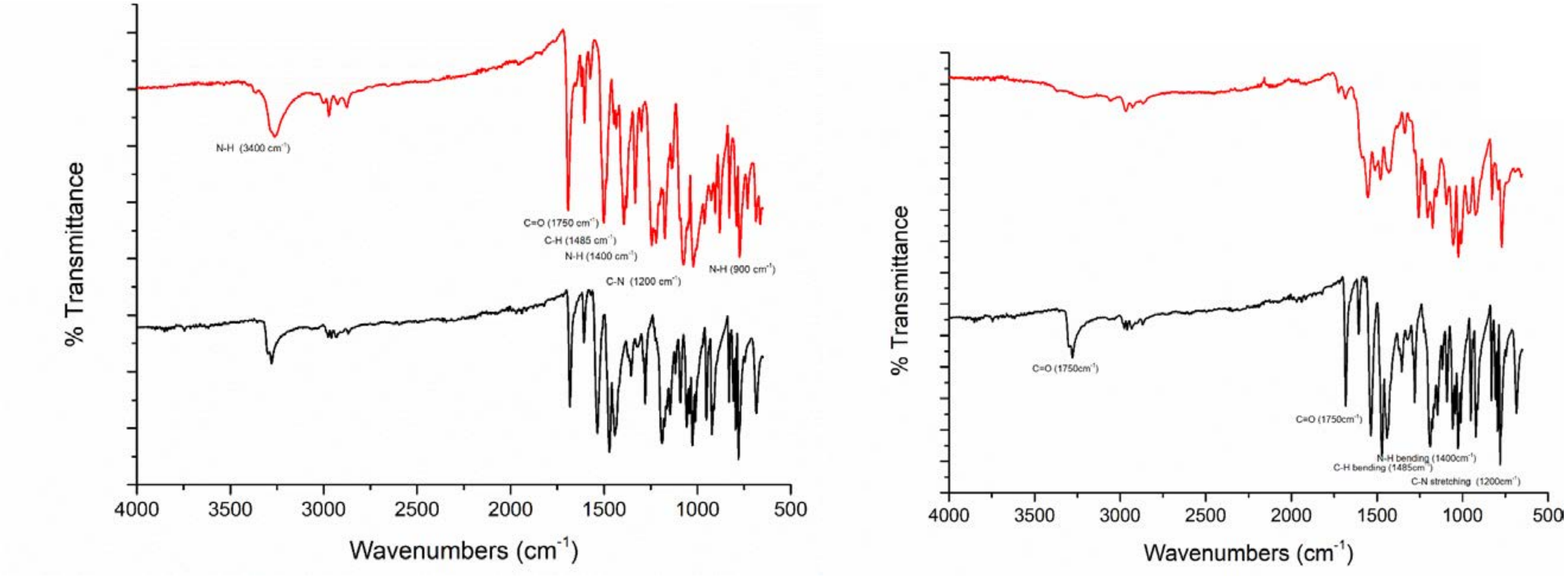

(a) **(b)**

Figure S52 (a) IR spectra for **AN-Et ligand** (black) and [**Zn(AN-Et)$_2$**] (red); **(b)** IR spectra for **AN-Et ligand** (black) and [**Cu(AN-Et)$_2$**] (red) isolated from a 2:1 ligand to metal precursor reaction conducted under microwave-assisted irradiation protocol.

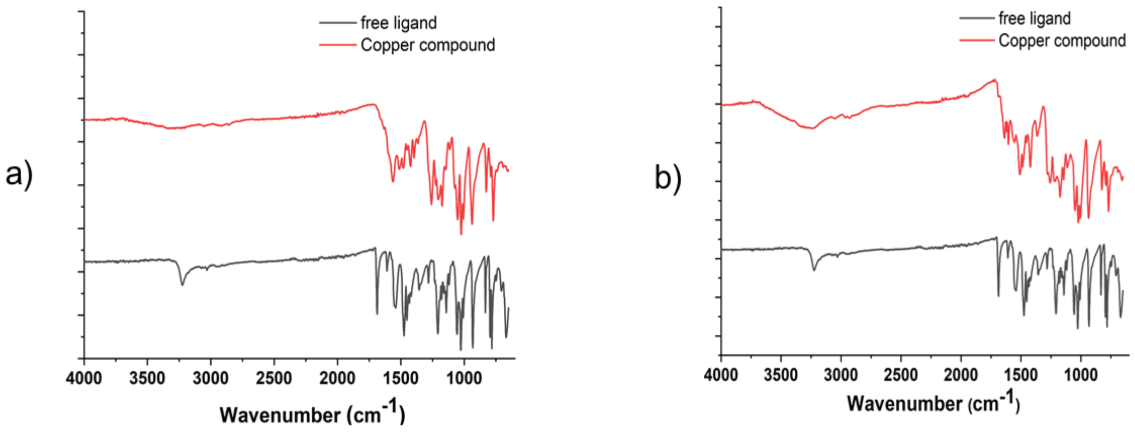

Figure S53 Comparative IR spectra of free ligand and copper compound. a) free ligand, and Cu-(AN-Me) species isolated from a 1:1 ligand:metal precursor ratio reaction at the room temperature, b) free ligand, and corresponding [Cu(AN-Me)$_2$] complex (isolated from the reaction with 2:1 ligand:metal precursor ratio, conducted at the room temperature). IR spectroscopy indicates that virtually identical products emerge from the two reaction methods.

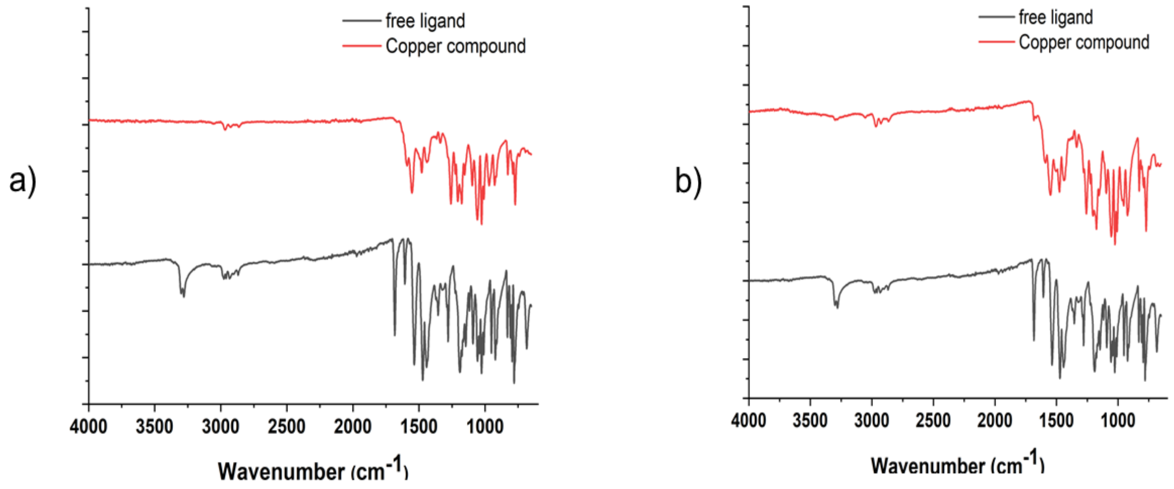

Figure S54 Comparative IR spectra of free ligand and copper compound. a) free ligand, and Cu-(AN-Et) species isolated from a 1:1 ligand:metal precursor ratio reaction at the room temperature, b) free ligand, and corresponding [Cu(AN-Et)$_2$] complex (isolated from the reaction with 2:1 ligand:metal precursor ratio, conducted at the room temperature). IR spectroscopy indicates that virtually identical products emerge from the two reaction methods.

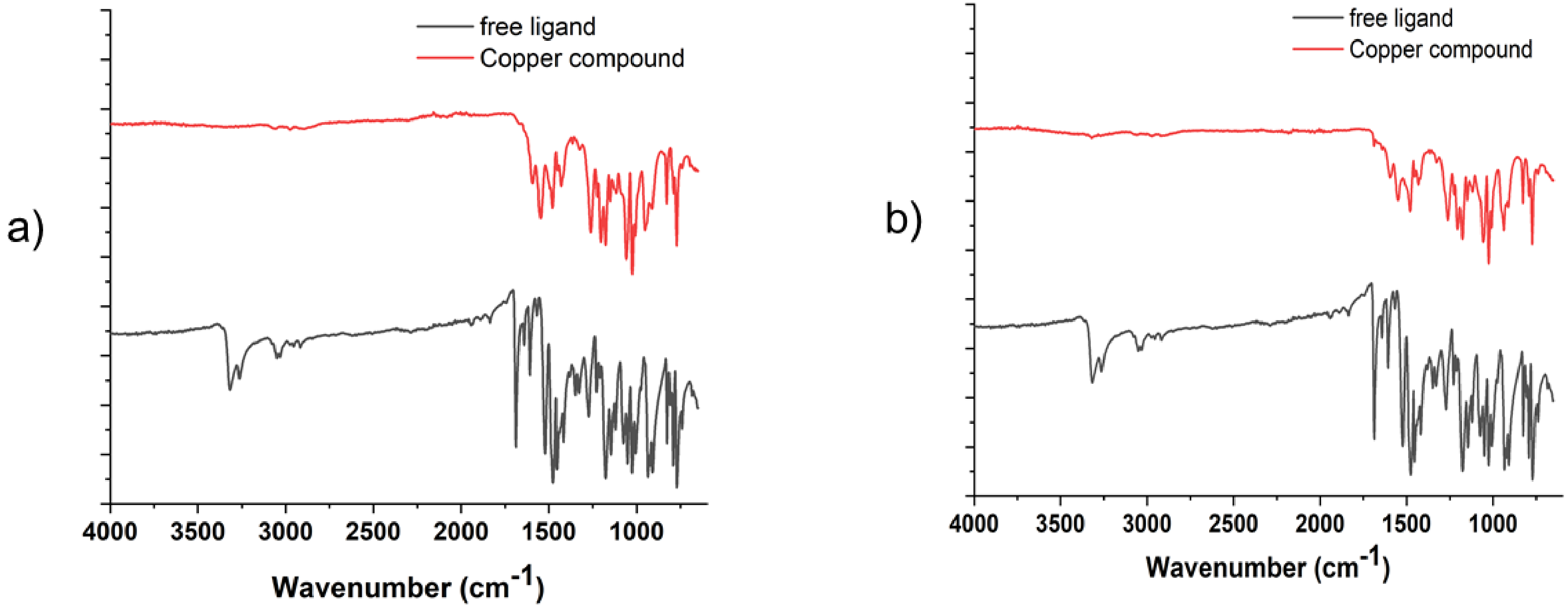

Figure S55. Comparative IR spectra of free ligand and copper compound. a) free ligand, and Cu-(AN-Allyl) species isolated from a 1:1 ligand:metal precursor ratio reaction at the room temperature, b) free ligand, and corresponding [Cu(AN-Allyl)$_2$] complex (isolated from the reaction with 2:1 ligand:metal precursor ratio, conducted at the room temperature). IR spectroscopy indicates that virtually identical products emerge from the two reaction methods.

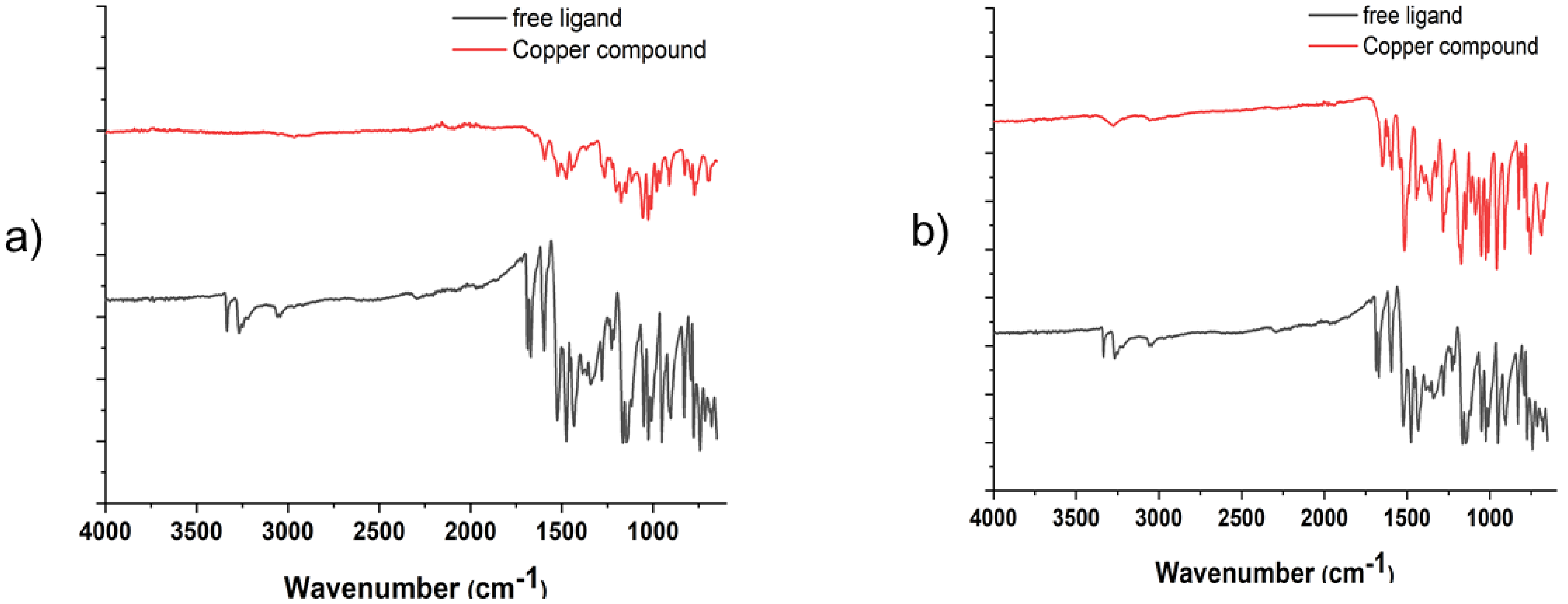

Figure S56. Comparative IR spectra of free ligand and copper compound. a) free ligand, and Cu-(AN-Ph) species isolated from a 1:1 ligand:metal precursor ratio reaction at the room temperature, b) free ligand, and corresponding [Cu(AN-Ph)$_2$] complex (isolated from the reaction with 2:1 ligand:metal precursor ratio, conducted at the room temperature). IR spectroscopy indicates that virtually identical products emerge from the two reaction methods.

7 EPR and Magnetic Moment Measurements for selected Cu(II) compounds

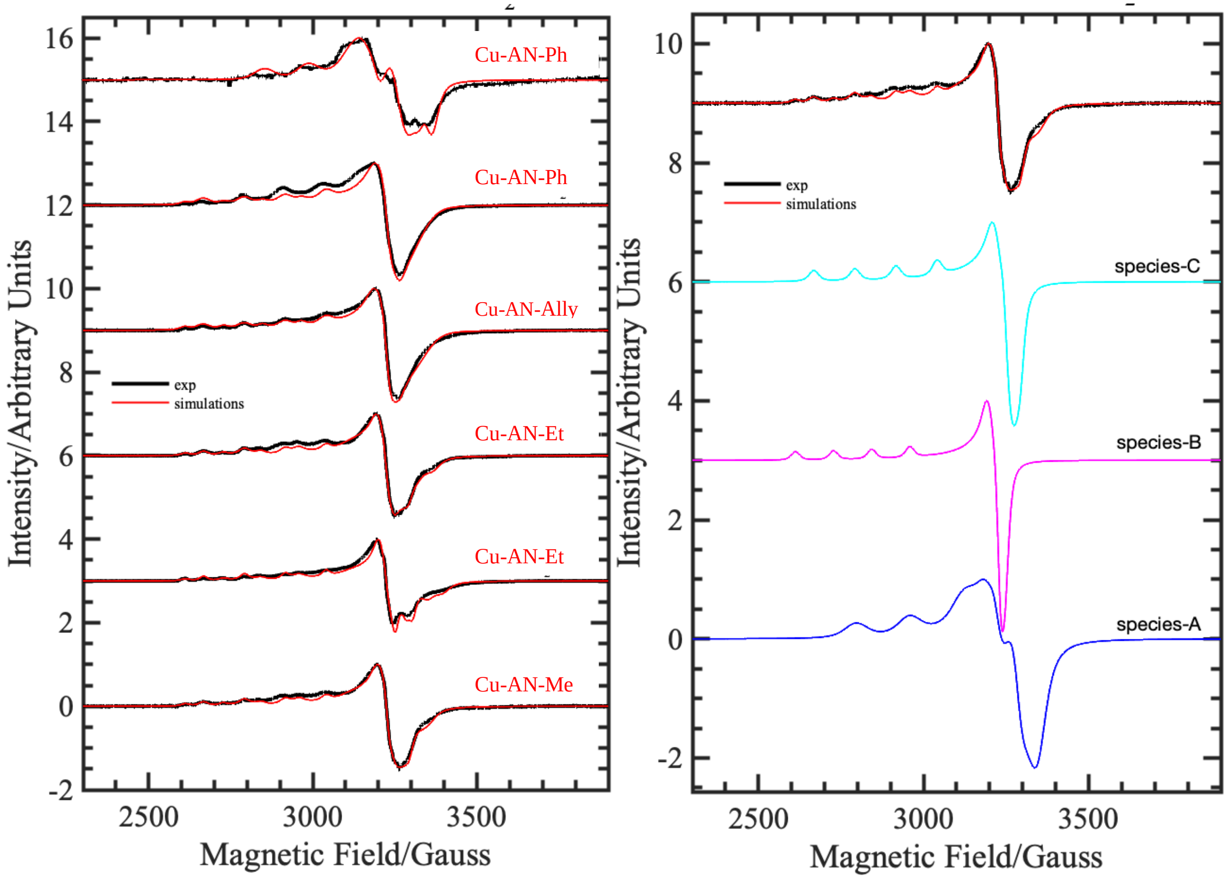

Figure S57. The LH panel shows the experimental cw EPR spectra of Cu-AN-Me, Cu-AN-Et-a, Cu-AN-Et-b, Cu-AN-Allyl, Cu-AN-Ph-a and Cu-AN-Ph-b species in frozen 7:1 *v/v* EtOH:DMSO solutions (black) and the simulated spectra (red). The RH panels displays the cw EPR spectrum of the different species identified in the sample denoted Cu-AN-Me (emerging from the 1:1 reaction of $Cu(OAc)_2$ with the AN-Me TSC ligand. These spectra were added together (see Table 6, main text for the weight/population) to model the experimental spectrum of the Cu-AN-Me (1:1). A similar procedure has been used to model all the EPR spectra of the species investigated here.

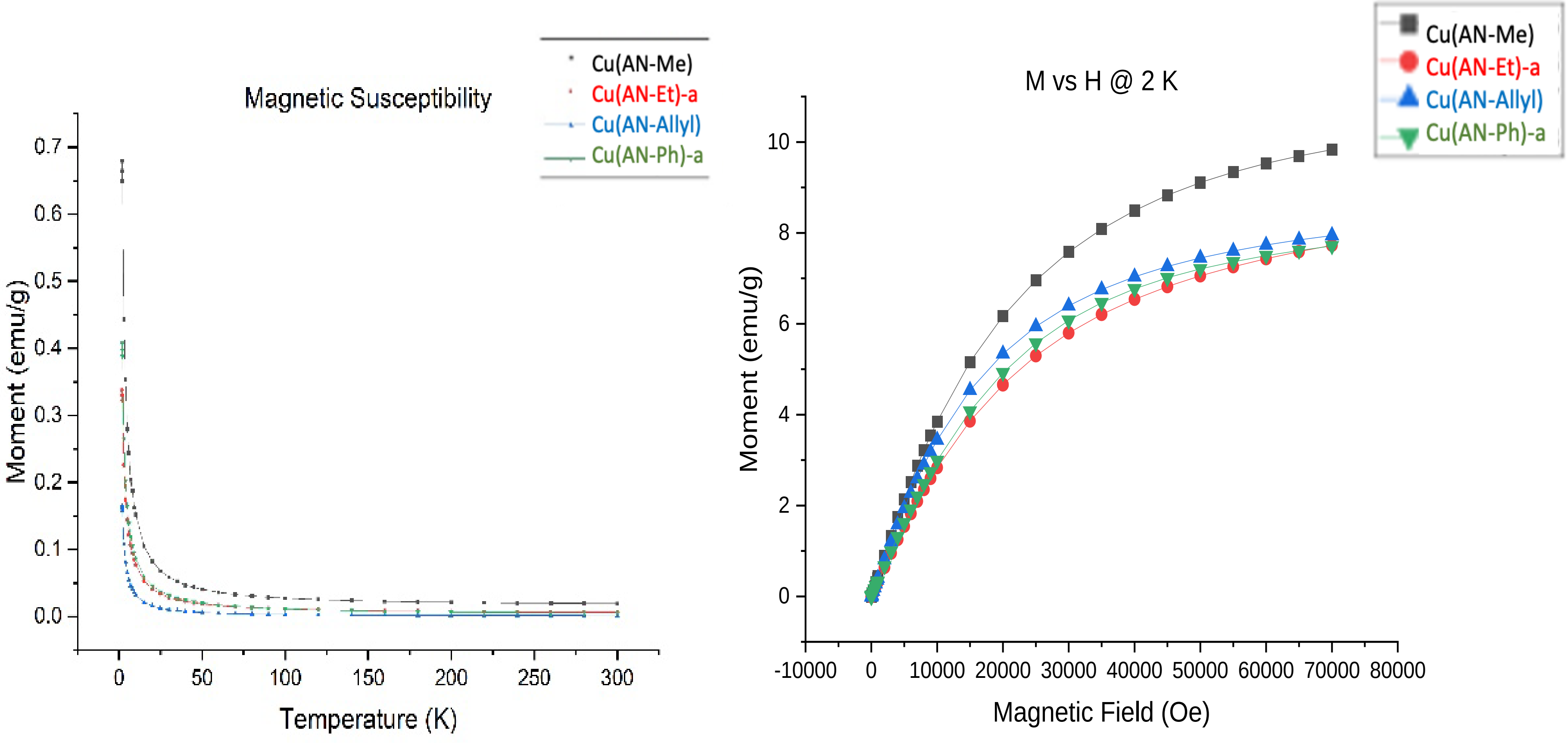

Figure S58 Qualitative magnetometry of selected samples emerging from the 1:1 reactions of $Cu(OAc)_2$ with TSCs Carried out at the room temperature. Measurements were recorded in the same samples as those analyzed by EPR, as polycrystalline powders dispersed in eicosane. Left panel, magnetisation versus temperature, right panel magnetisation versus applied magnet field at 2 K.

8 Radiochemistry assays

General Radiolabeling Procedure: The ligand or complex was prepared as a 1.0 mg/mL solution in DMSO and the reaction followed closely the similar protocol applied for the ‘cold’ copper incorporation in DMSO. To 1 mL of ligand in DMSO (of approximate concentrations: AN allyl, AN-ethyl, AN-Me and AN-Ph: 0.4 mM; AA-allyl 0.4 mM; PH-allyl 0.4 mM; PH-Ethyl 0.5mM) ca. 1.5 mg/mL of $Cu(OAc)_2$ in MeOH was added. The heating proceeded for ca. 2 hrs heated to 60 ^{o}C. For radiochemistry, ^{64}Cu stock solutions of ca. 100-165 MBq were used. $^{64}Cu^{2+}$ extracted from nickel target and purified from $^{64}Ni^{2+}$ using an ion exchange column. An aqueous $^{64}CuCl_2$ solution was acquired in 0.1 mol dm^{-3} HCl, which was diluted with 0.2 mL $^{64}CuCl_2$ in 0.1 mol dm^{-3} $NaOAc_2$. To generate the $^{64}Cu(OAc)_2$ stock, 0.1M sodium acetate (pH 5.5, max 500 mL) was added to an aqueous solution of $^{64}CuCl_2$ (used as received).

Method 1. To each 2 mL reaction vial, a 200 μL aliquot (10 MBq) of ^{64}CuOAc in MeOH was added. To this, ca. 100 μL ligand aliquot (from the stock solution prepared as above) in DMSO, 400 μL water was added, and the reaction was monitored by radioHPLC (with 25 μL aliquots injected in the HPLC) at 20 minutes, 1 h and 2 h intervals. Reactions were conducted either at the room temperature or with conventional heating. It was observed that microwave irradiation and shorter reaction times did not improve incorporation yield for the copper radiolabelling. HPLC conditions: Reverse phase column c18 gradient run 95% water (0.1% TFA) and 5% acetonitrile (0.1% TFA), 25 minutes.

Method 2. Alternative method for copper-64 radiolabelling at the room temperature involved the AN ligands with simple substitutents R = Me, Et, Allyl, Ph). In each case, 10 µL of the stock ligand solution was diluted with 40 µL of DMSO and 50 µL $^{64}Cu(OAc)_2$ was added (< 10 MBq per experiment) and the reaction stirred for 20 min at room temperature. Reactions were carried out wit and without the addition of 1 equivalent of NH_4OH or LiOH as a base to deprotonate the ligand. At that time, an aliquot (20 µL) was removed via syringe, and analysed by HPLC (radio and UV detection, in series).

Method 3. Alternative labelling methods with larger activity levels were also applied: $^{64}Cu(OAc)_2$ activity of 185 MBq (ca 5 mCi) for the AN-Et and or 111 MBq (ca 3 mCi) for each of the reactions for the labelling of the AN-Ph derivative. Solutions of the precursors (ANEt, ANPh) were prepared as 1mg/ml in dimethyl sulphoxide or THF. In each borosilicate glass reaction tube, 50µl of the precursor solution were diluted with 150µl Millipore water and 150 µl of the $^{64}Cu(OAc)_2$ solution added. Mixtures were vortexed for 5 minutes followed by heating at 90 ^{o}C between 30 and 120 minutes. To monitor the reaction progress, 20 µl of each reaction mixture were taken for HPLC and analyses were carried out every 30 min. A 20 minute gradient method (C18) was employed using acetonitrile/water 5:95 as the mobile phase (0.1% TFA was used in both mobile phases), as follows. In all cases the 20 min HPLC gradient methods A, B, or C:

RadioHPLC Method A was performed using a Waters C-18 column (4.6 x 250 mm) with UV/Vis detection at λobs = 254nm and 410 nm with a 0.9 mL/min gradient elution method (Solvent A: THF with 0.1 % TFA v/v, Solvent B: water with 0.1% TFA v/v): start 5 % A, gradient over 12 min reaching 95 % A, hold to 15 min at 95 % A, reverse gradient till 18 min reaching 5 % A, then hold to 20 min at 5 % A.

RadioHPLC Method B was performed using a Waters C-18 column (4.6 x 250 mm) with UV/Vis detection at λobs = 254nm and 410 nm with a 1.0 mL/min gradient elution method (Solvent A: acetonitril, Solvent B: water with 0.1% NH4OAc v/v): start 5 % A, gradient over 12 min reaching 65 % A, hold to 15 min at 65 % A, reverse gradient till 18 min reaching 5 % A, then hold to 20 min at 5 % A.

RadioHPLC Method C was performed using a Waters C-18 column (4.6 x 250 mm) with UV/Vis detection at λobs = 254nm and 410 nm with a 1.0 mL/min gradient elution method (Solvent A: acetonitrile with 0.1 % TFA v/v, Solvent B: water with 0.1% TFA v/v): start 5 % A, gradient over 12 min reaching 65 % A, hold to 15 min at 65 % A, reverse gradient till 18 min reaching 5 % A, then hold to 20 min at 5 % A.

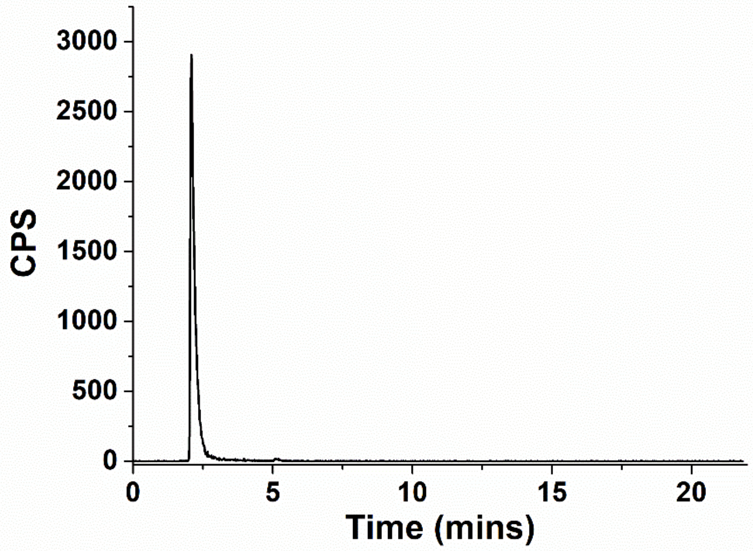

Figure S59 RadioHPLC trace of the $^{64}Cu(OAc)_2$ stock.

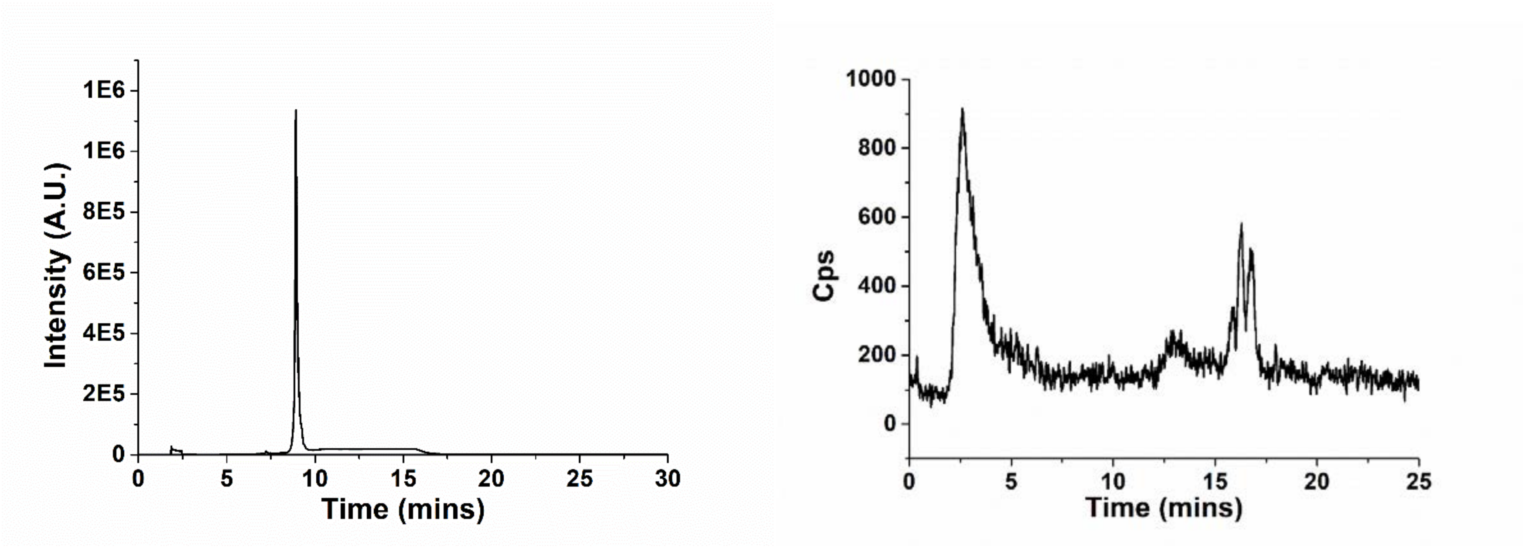

(a) **(b)**

Figure S60 (a) HPLC with UV detection of AN-Et labelling reaction mixture, pre-purification/separation (b) RadioHPLC trace of the ^{64}Cu labelled AN-Et using Method 3 (90 minutes heating at 90 ^{o}C).

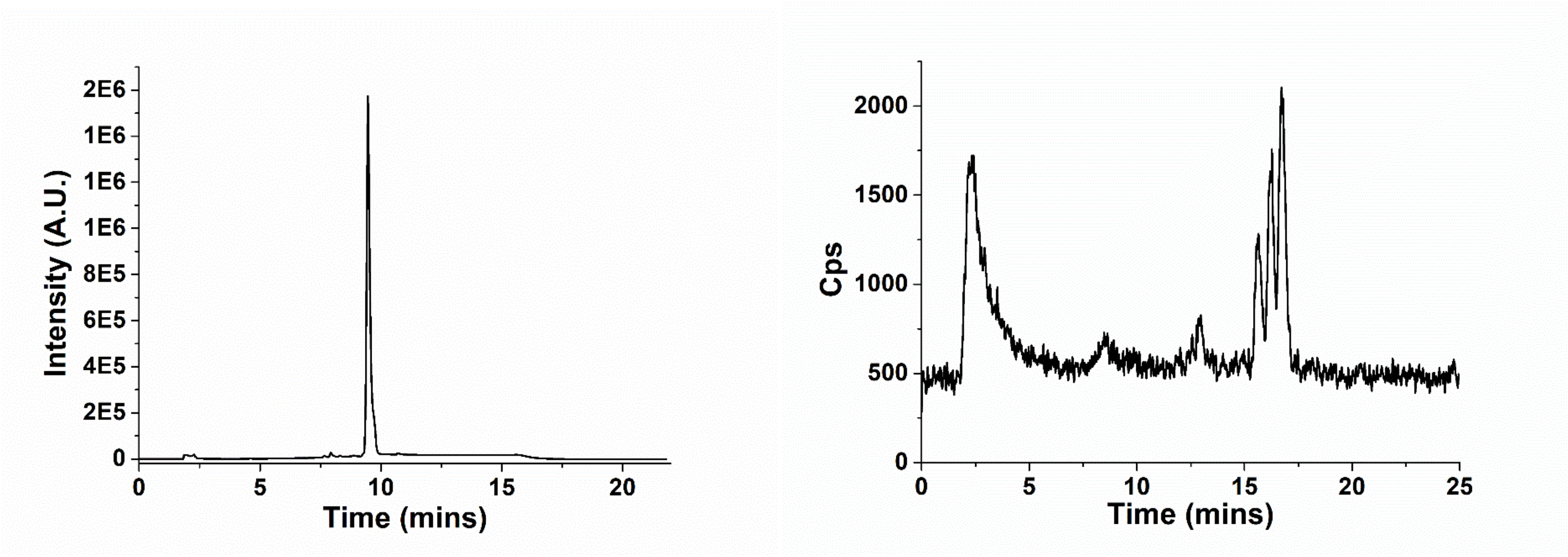

(a) **(b)**

Figure S61 (a) HPLC with UV detection of AN-Ph labelling reaction mixture, pre-purification/separation; (b) RadioHPLC trace of the ^{64}Cu labelled AN-Et using Method 3 (90 minutes heating at 90 ^{o}C).

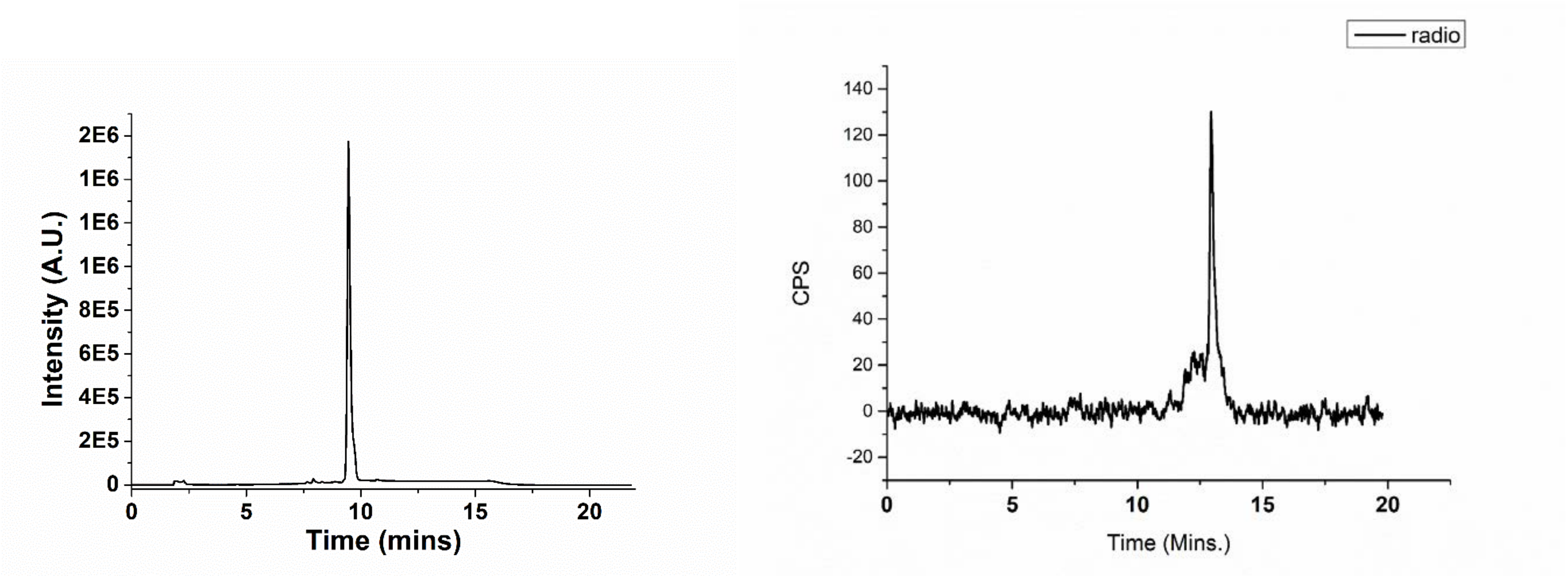

Figure S62 (a) HPLC with UV detection of AN-Allyl labelling reaction mixture, pre-purification/separation; (b) RadioHPLC trace of the ^{64}Cu labelled AN-Allyl ligand using radiolabelling Method 2 above (10 MBq; 60 ^{o}C, 2 h).

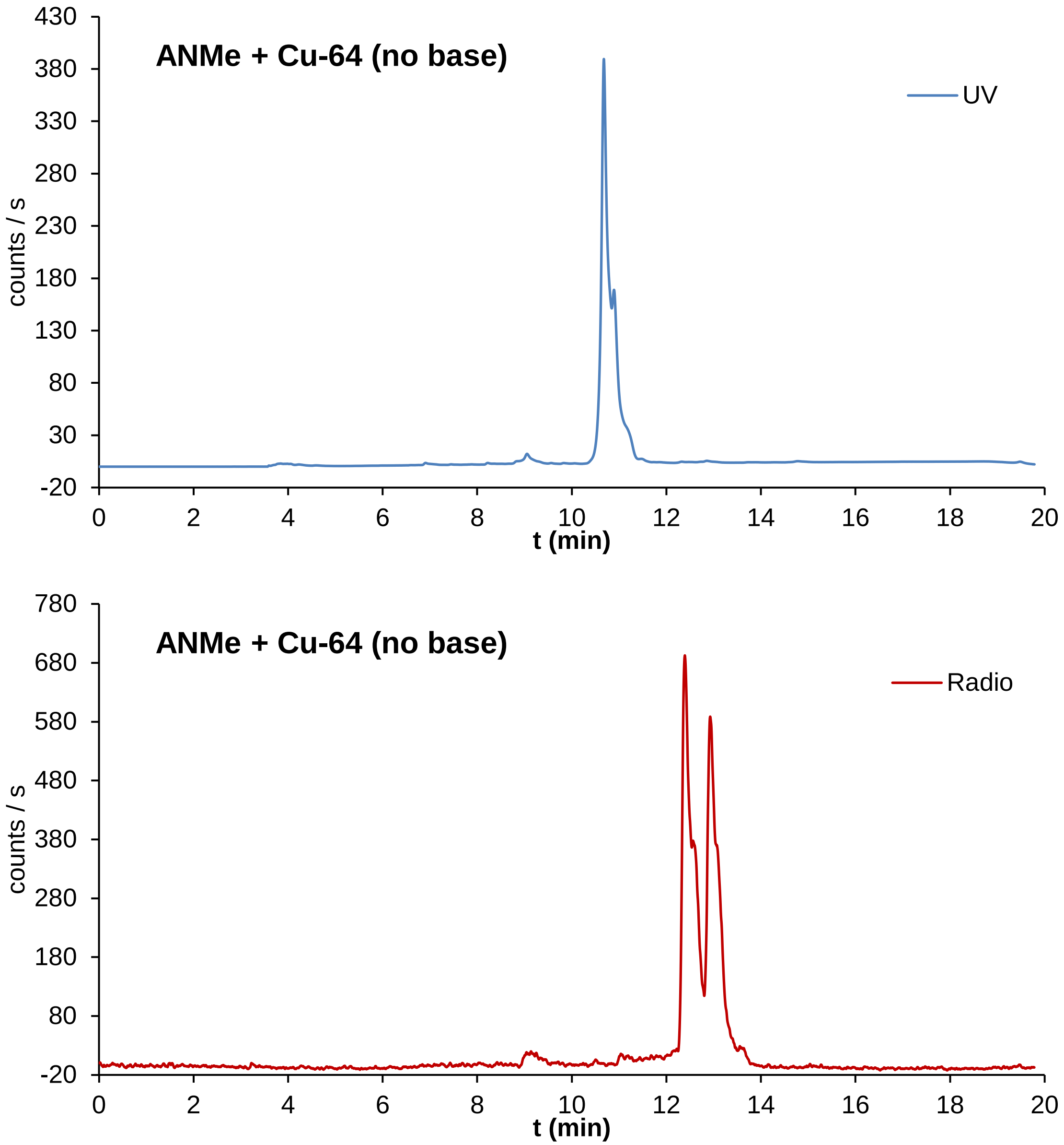

Figure S63 HPLC traces, post-optimisation and purification: UV detection, 280nm(blue) and radio HPLC (red). AN-Me radiolabeled with ^{64}Cu applying conventional heating techniques (Method 2, 60 ^{o}C, 2 h)

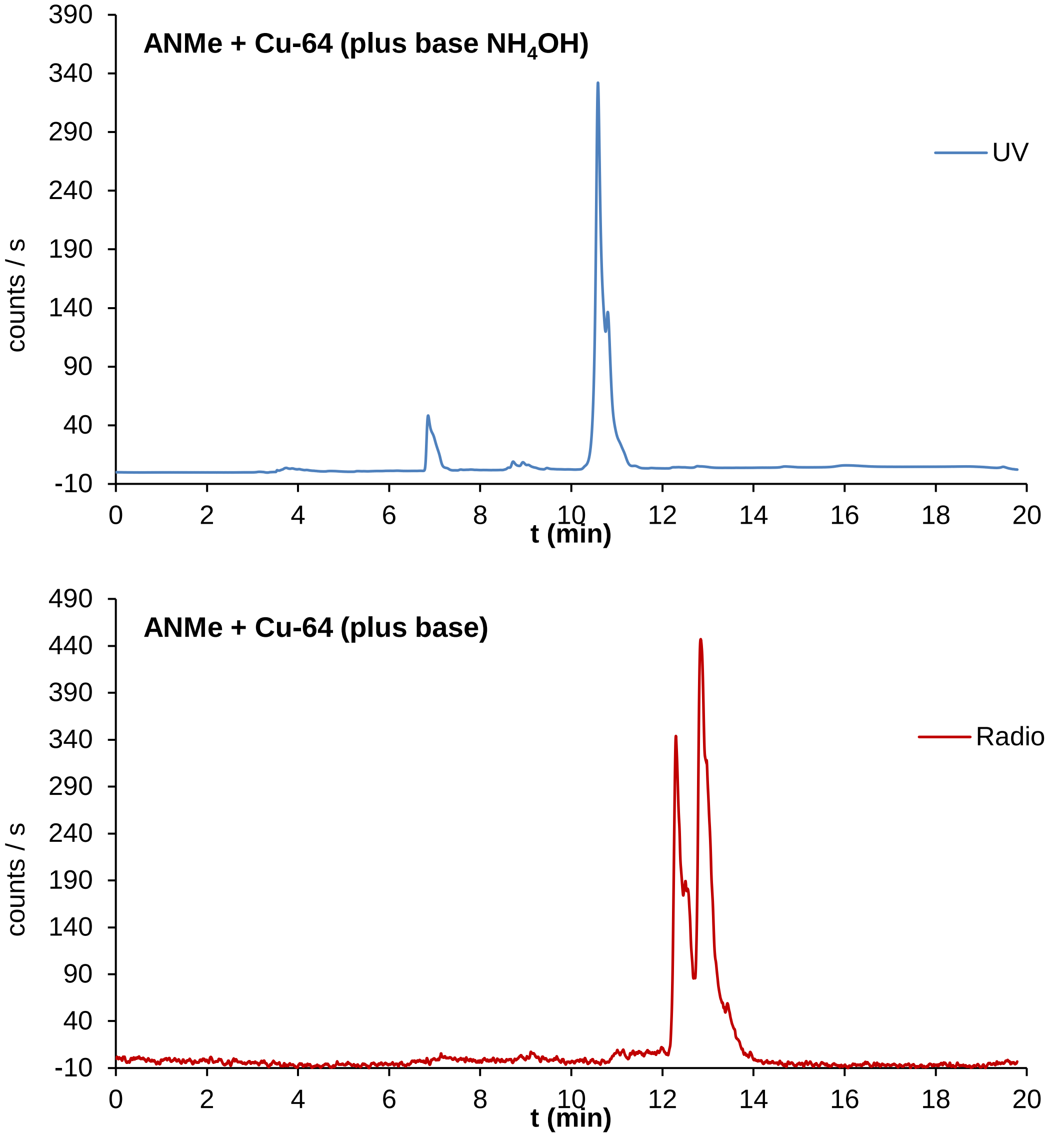

Figure S64 HPLC traces from optimizations and purification assays: UV-detection (280 nm) HPLC (blue) and radio HPLC (red) traces of AN-Me radiolabeled with ^{64}Cu applying conventional heating techniques (Method 2, 60 ^{o}C, 2 h, using NH_4OH as the base).

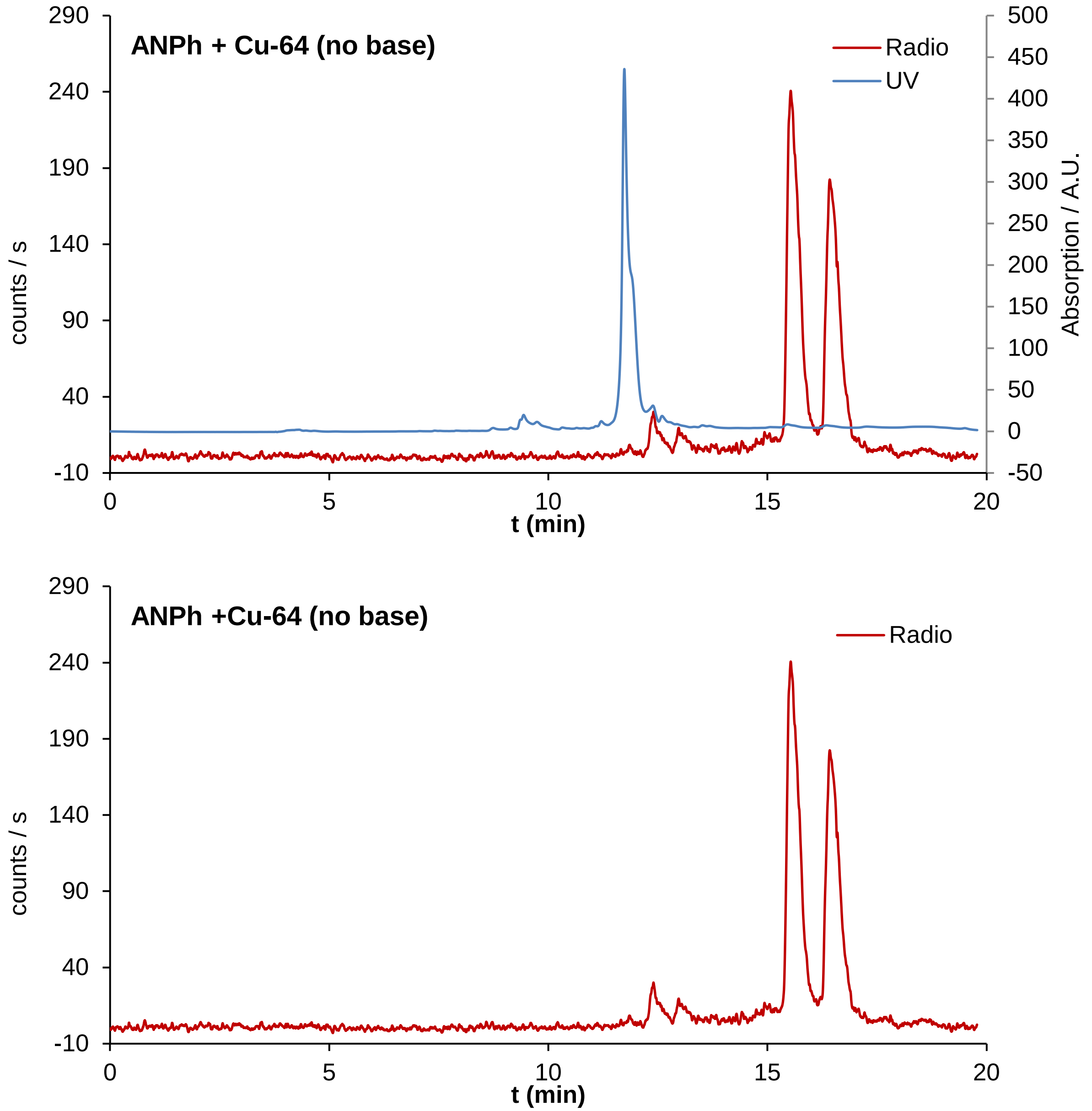

Figure S65 UV-detection (280 nm) HPLC (blue) and radio HPLC (red) traces of AN-Ph radiolabeled with ^{64}Cu applying conventional heating techniques (Method 2, 60 ^{o}C, 2 h).

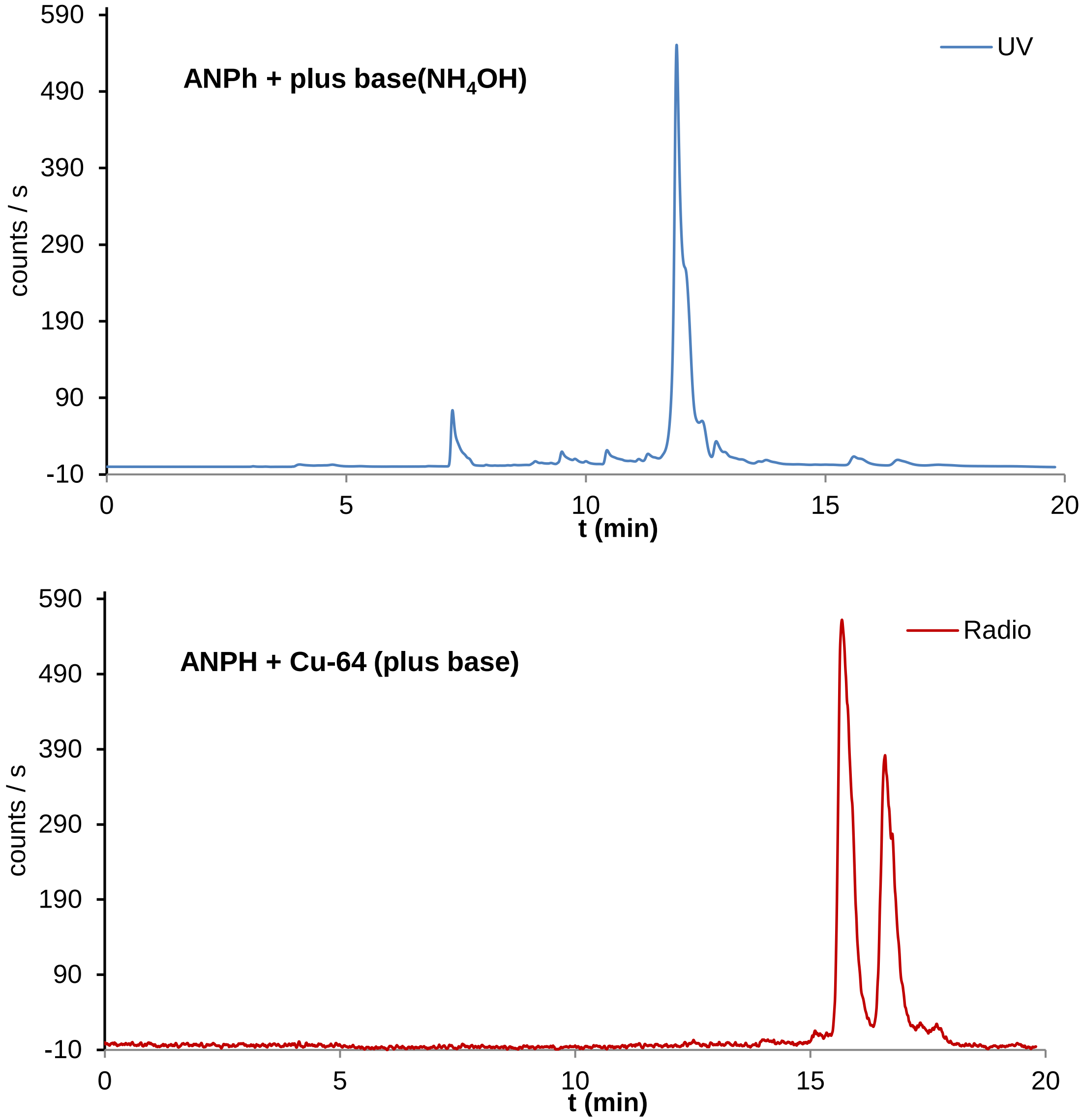

Figure S66 UV-detection HPLC (280 nm, blue) and radio HPLC (red) traces of AN-Ph radiolabeled with ^{64}Cu applying conventional heating techniques. The base added prior to radiolabelling was (excess) NH_4OH (Method 2, 60 ^{o}C, 2 h).

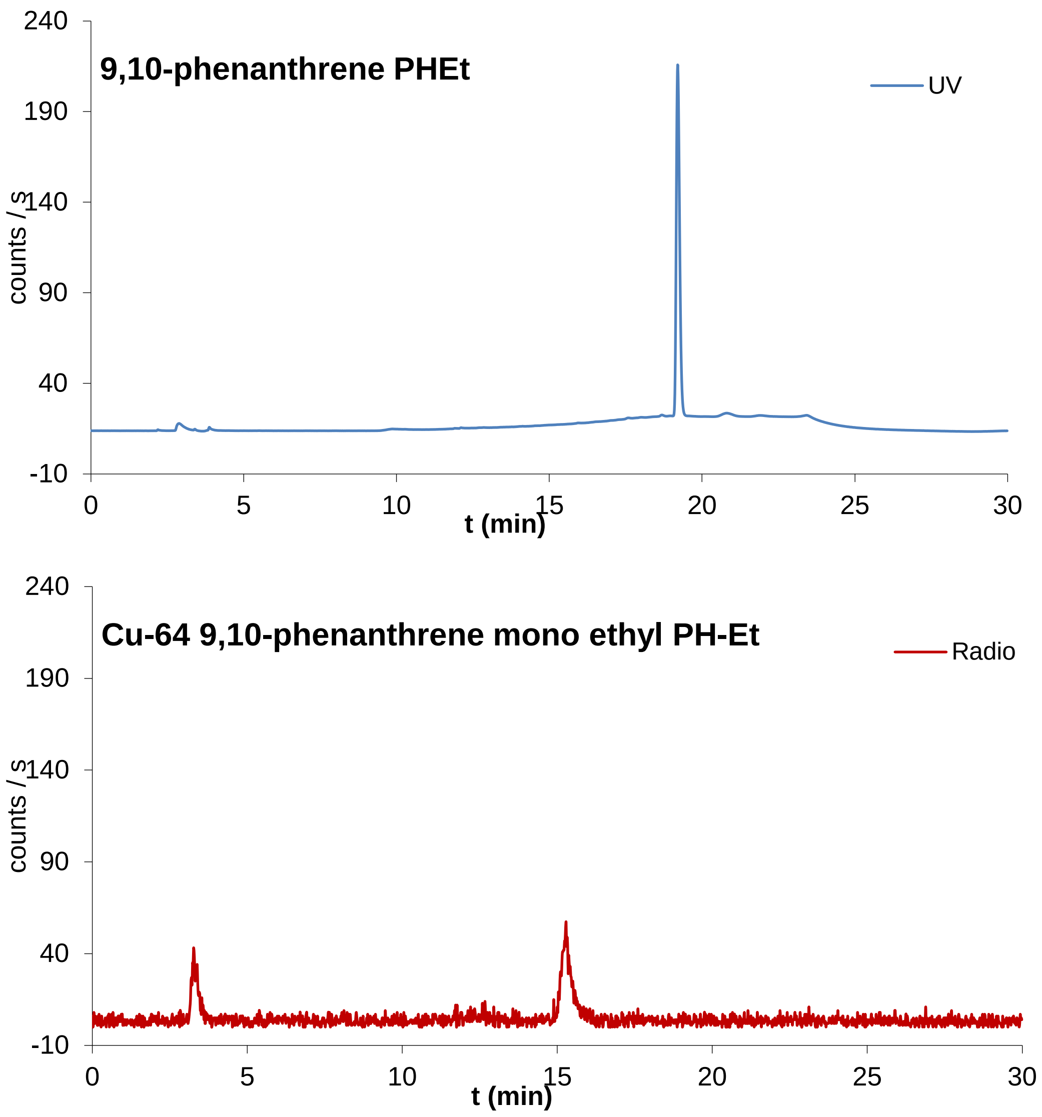

Figure S67 UV-detection HPLC (280 nm, blue) HPLC (blue) and radio HPLC (red) traces of mono(4-ethyl-3-thiosemicarbazone) 9,10-phenanthrenequinone radiolabeled with ^{64}Cu applying conventional heating techniques (Method 1, room temperature, 1 h).

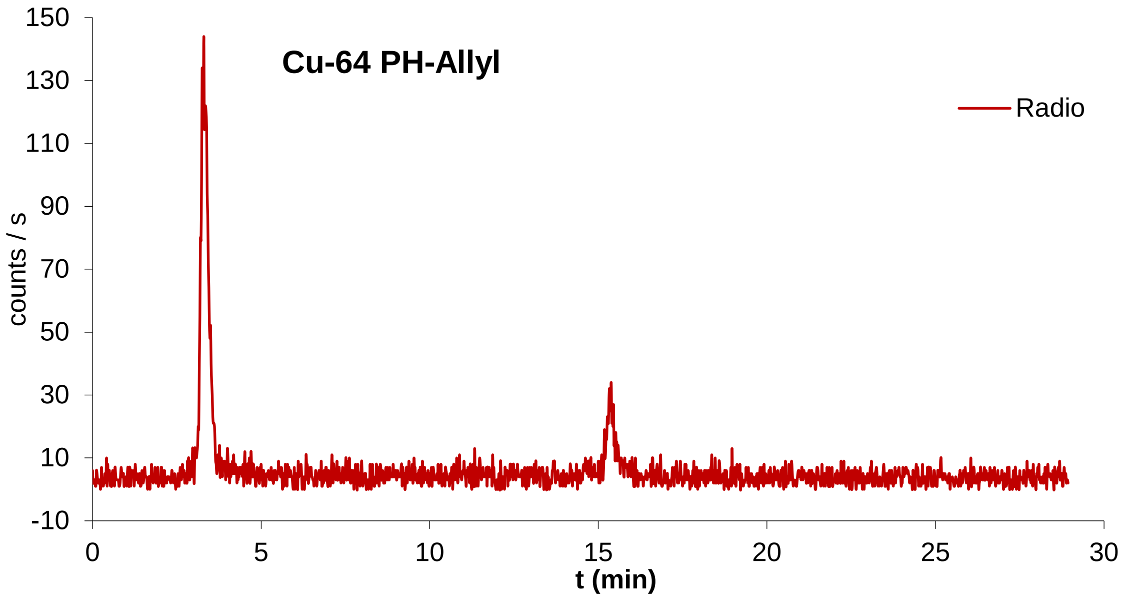

Figure S68 Radio HPLC (red) trace of mono(4-allyl-3-thiosemicarbazone) 9,10-phenanthrenequinone radiolabeled with ^{64}Cu (Method 1, room temperature, 1 h)

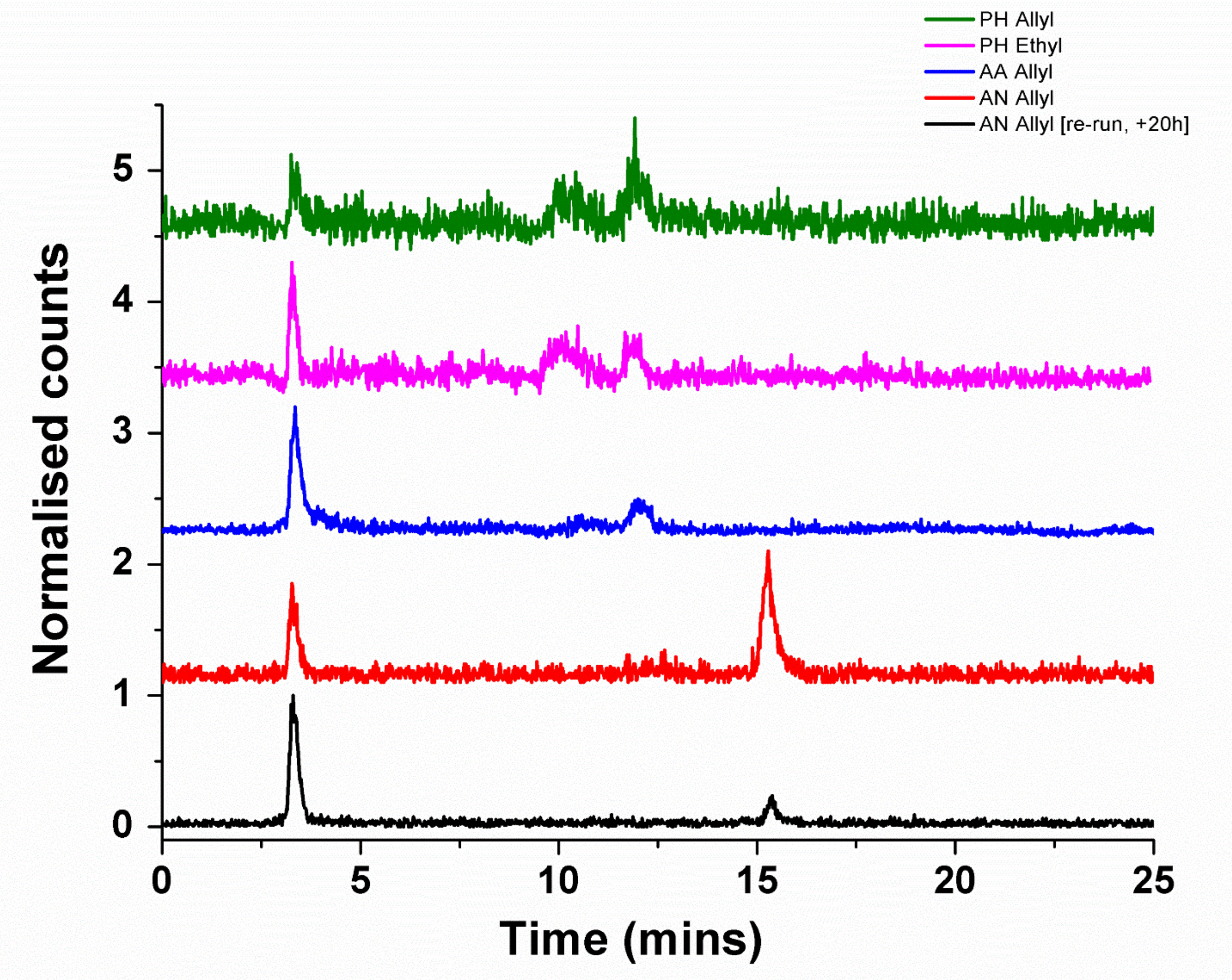

Figure S69. Overview of the normalised radiotraces showing the room temperature radiochemistry for the radiolabelling of a range of TSCs ligands using $^{62}Cu(OAc)$.

9 MTT Assays

The optical density (OD) was measured at 550 nm in BMG Labtech Fluostar Optima microplate reader. Cell viability was calculated by comparing the OD of treated *versus* non-treated control cells by the formula: % Cell viability = ($OD_{treatment}$ -OD_{blank}) / ($OD_{control}$-OD_{blank}) x 100. Data were obtained from five consistent results and IC_{50} was calculated using Origin 8 as half the height of the fitted curve for each compound and for each individual experiment.

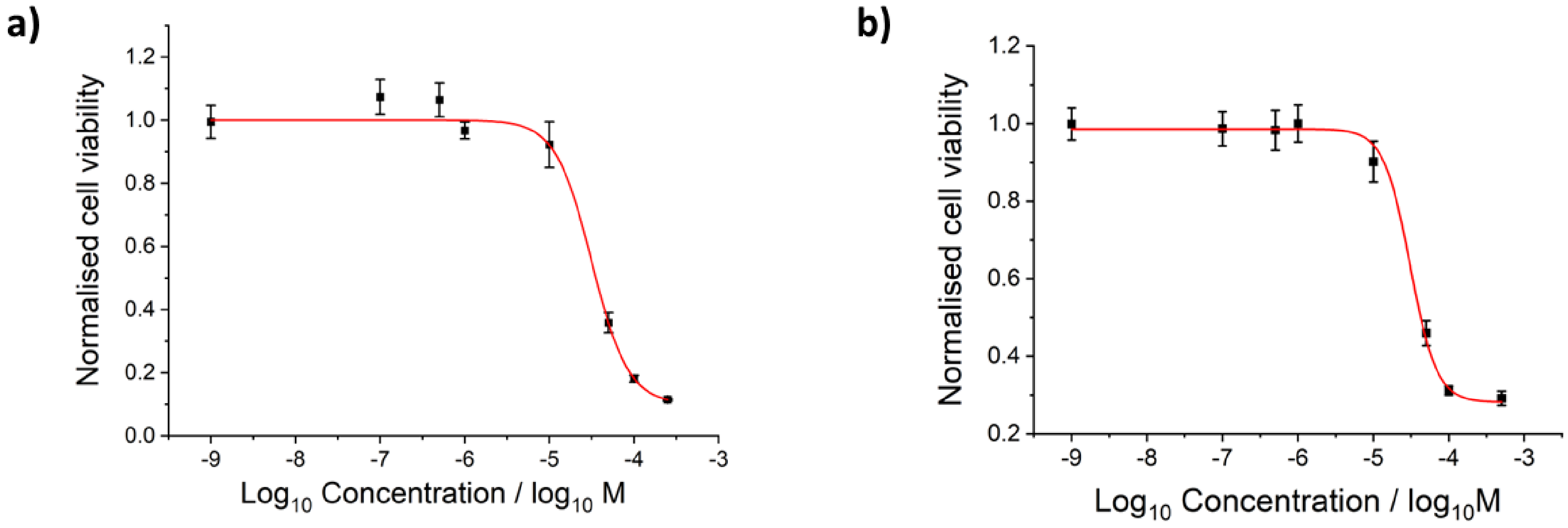

Figure S70. Dose response for the normalized (average) 48 h MTT assays of cis-platin in: a) HeLa cells, IC_{50} value is (31.28 ± 9.38) µM and b) PC-3 cells, IC_{50} value is (30.64 ± 3.26) µM.

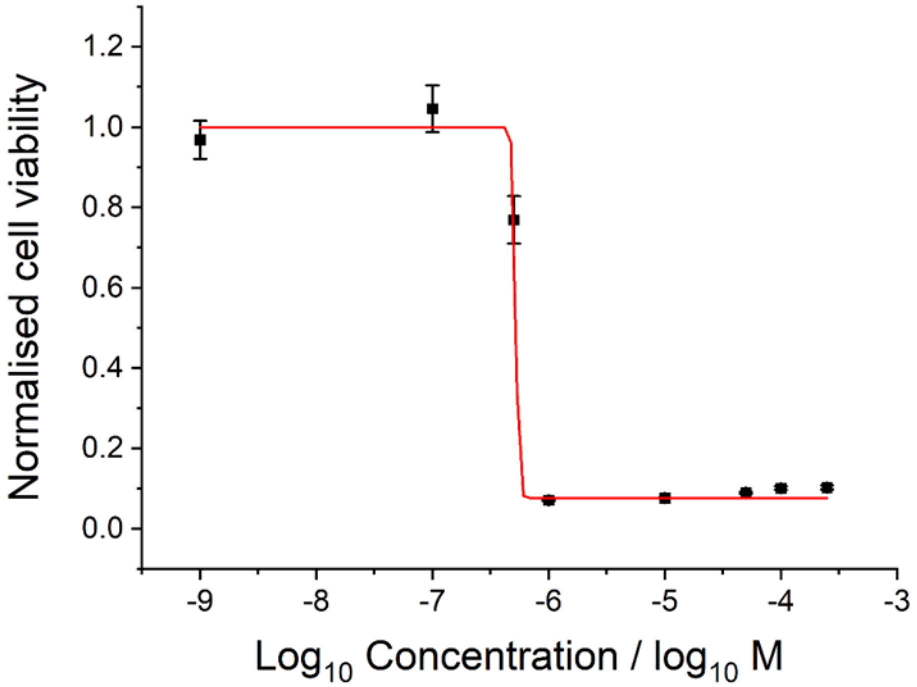

Figure S71 Dose response for the normalized (average) 48 h MTT assays of [Cu(ATSM)] in HeLa cells, IC_{50} value is (0.52 ± 0.01) µM.

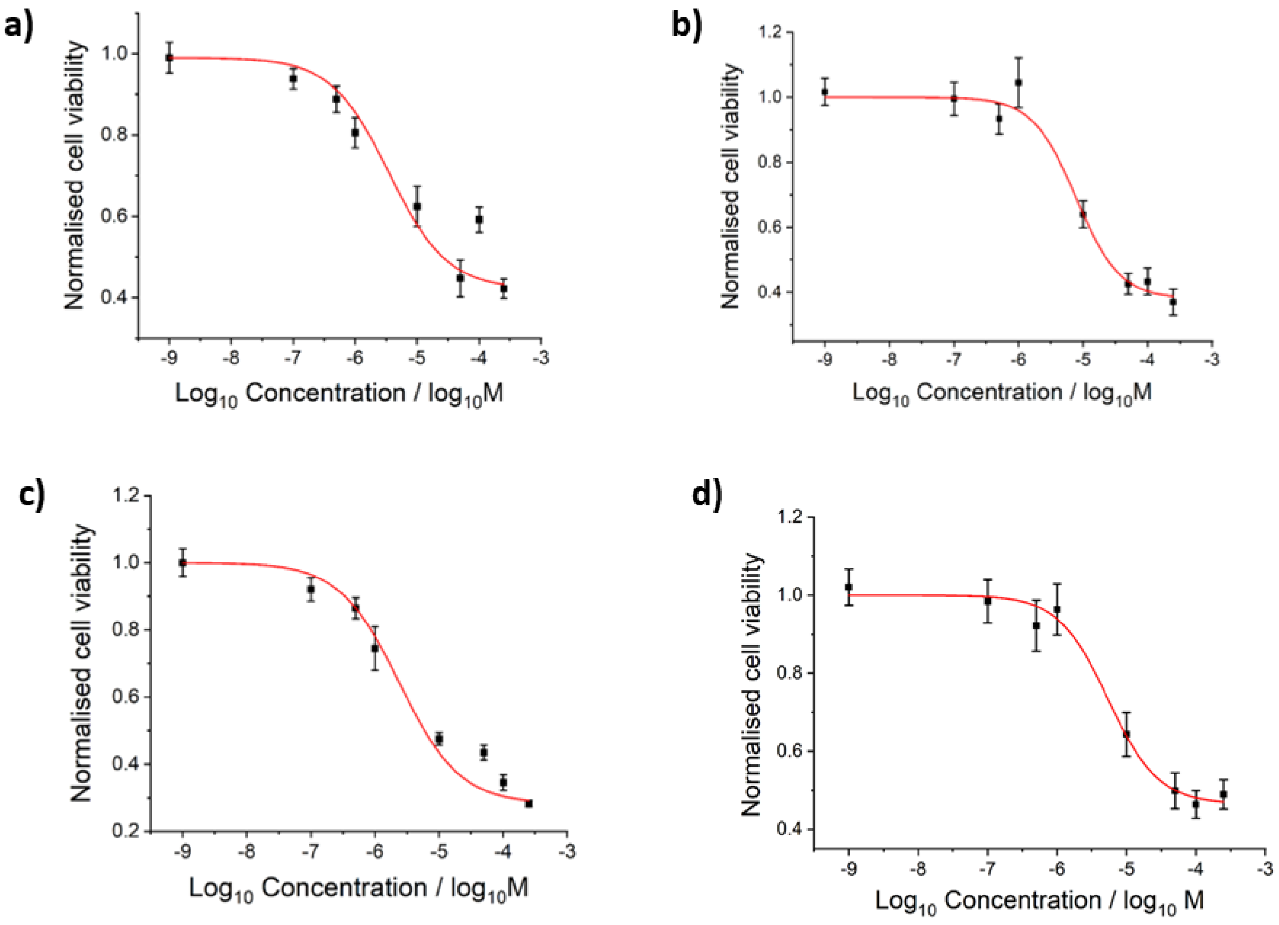

Figure S72 Dose response for the normalised (average) 48 h MTT assays of simple monothiosemicarbazone ligands in PC-3 cells. a) AN-Me, IC_{50} value is (3.54 ± 0.03) μM; b) AN-Et, IC_{50} value is (7.80 ± 0.04) μM; c) AN-Allyl, IC_{50} value is (3.47 ± 0.03) μM; d) AN-Ph, IC_{50} value is (5.43 ± 0.02) μM.

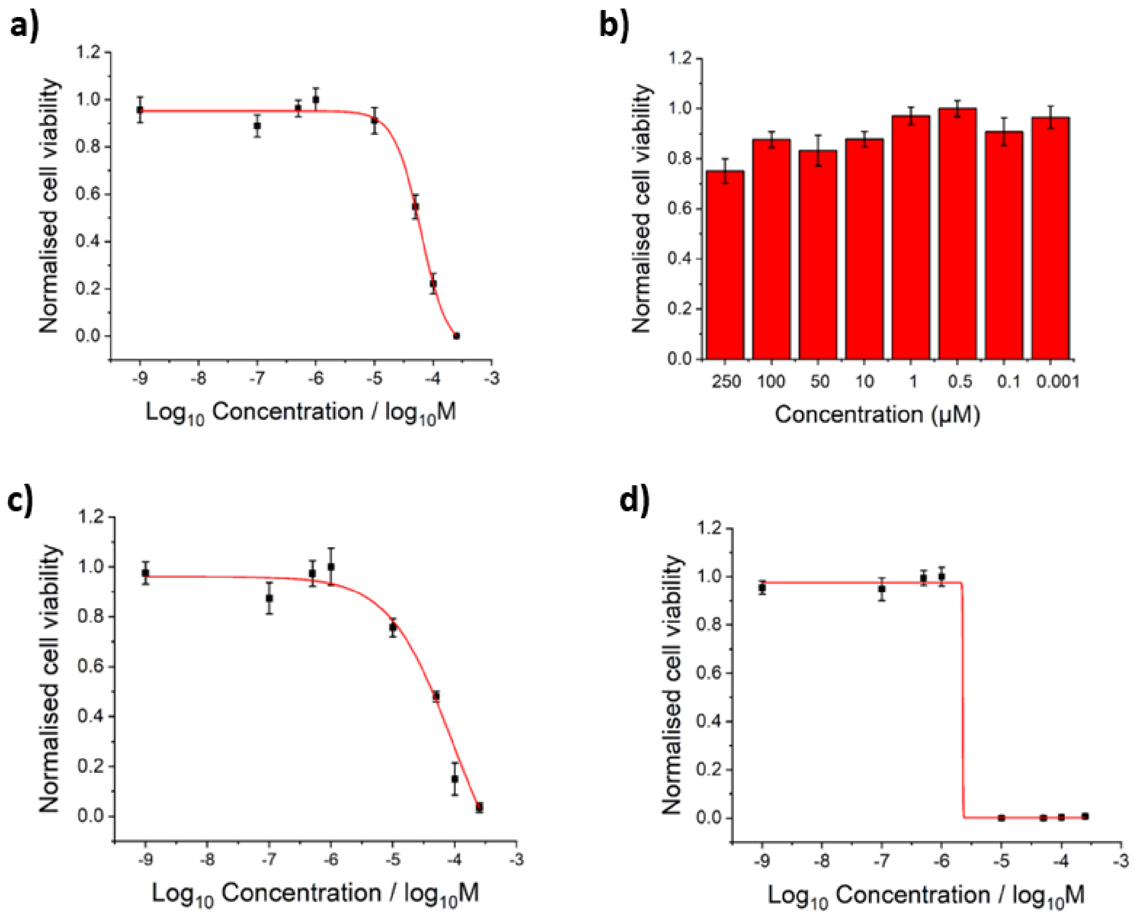

Figure S73 Dose response for the normalized (average) 48 h MTT assays of selected Zn(II) compounds and Cu(II) compounds in PC-3 cells. a) [Zn(AN-Et)$_2$] IC$_{50}$ value is (61.58 ± 5.38) µM; b) [Zn(AN-Ph)$_2$]: this compound showed a low cytotoxicity and therefore the dose response curve did not give a suitable IC$_{50}$; the cell viability is presented as the bar chart here; c) [Zn(AA-Et)$_2$] IC$_{50}$ value is (44.11 ± 2.08) µM; d) [Cu(AN-Et)$_2$] IC$_{50}$ value is (2.25 ± 0.01) µM.

Table S4 An overview of the IC_{50} values for HeLa and PC-3 cells following exposure to AN ligands and cis-platin for 48 h based on the dose-response curves as derived from MTT assays; data presented as mean ± SD, n = 5; SD: standard deviation.

	**HeLa**	**PC-3**
**AN-Me**	0.11 µM ± 0.01 [7]	3.54 µM ± 0.03
**AN-Et**	14.12 µM ± 2.67 [8]	7.80 µM ± 0.04
**AN-Allyl**	0.21 µM ± 0.06 [7]	3.47 µM ± 0.03
**AN-Ph**	10.04 µM ± 1.72	5.43 µM ± 0.02
**Cis-platin**	31.28 µM ± 9.38	30.64 µM ± 3.26

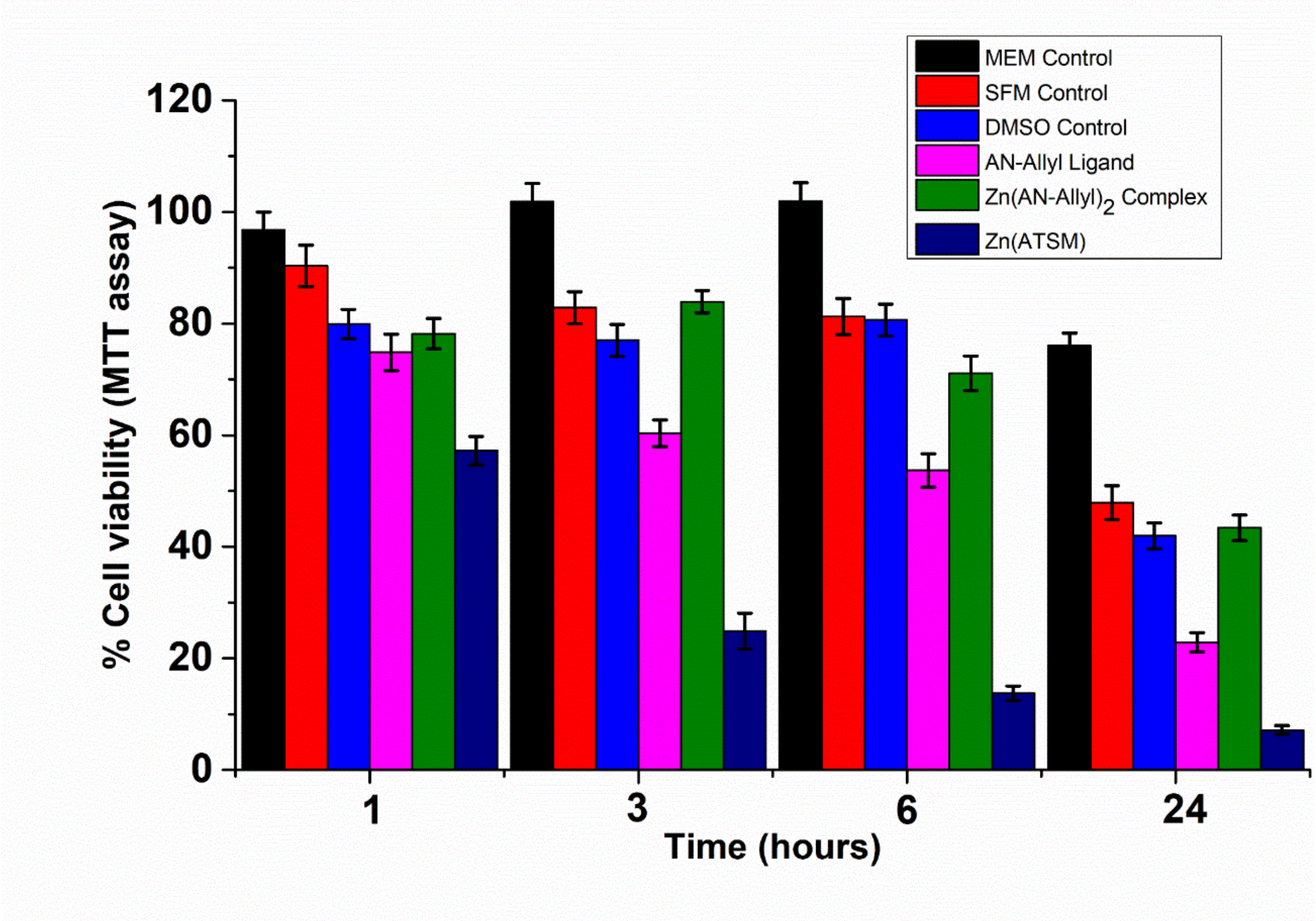

Figure S74 Qualitative comparative time course by MTT assays in HeLa cells conducted at 10 μM concentration to compare the effect of AN-Allyl, Zn(AN-allyl) with that of the well known [Zn(ATSM)] compound over 24 h assays.

10 Selected Structural Parameters and DFT Calculations

Table S5. Experimental selected bond distances and angles for compounds AN-12 and the known mono(4-ethyl-3-thiosemicarbazone) acenaphthenequinone AN-Et, determined from X-ray diffraction crystallography. The X-ray structure of AN-Et was previously reported by us [2]. The .cif file for the compound denoted AN-Et was downloaded from CSD (CCDC: 2131107) and relevant structural parameters were included here for comparison purposes only.

	**AN-12**	**AN- Et**
**O-C1 (Å)**	**1.225 (2)**	**1.223 (2)**
**C1-C2 (Å)**	**1.514 (2)**	**1.515 (2)**
**N1-C2 (Å)**	**1.296 (2)**	**1.294 (2)**
**O-C1-C2 (º)**	**126.24 (17)**	**125.6 (2)**
**C1-C2-N1 (º)**	**127.29 (16)**	**128.2 (2)**

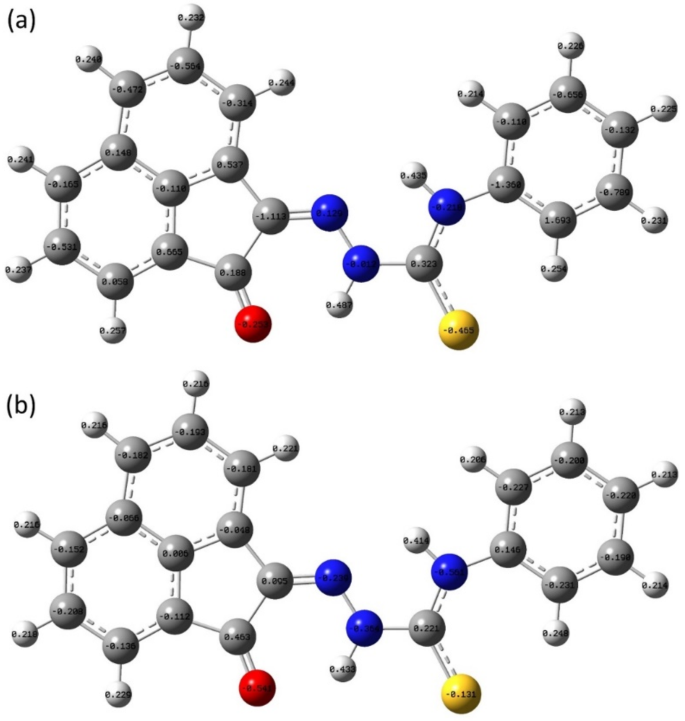

(c)

Figure S75. DFT calculated structure of the free base AN-Ph ligand: **(a)** Mulliken and **(b)** natural bonding orbital charge distribution of thiosemicarbozone. Structure was optimized using the BVP86 exchange correlation functional and 6311+G* basis set; **(c)** The x-ray structure of a polymorph of the AN-Ph ligand (content of the asymmetric unit shows co-crystallization of AN-Ph ligand with one molecule of DMSO which is H-bonded to one of the NH groups).

Table S6. Selected bond lengths and bond angles for thiosemicarbazone ligand calculated using DFT with different functionals and basis sets. Values compare closely to those found experimentally for AN-Ph in this, and our previous studies. [2]

Bond/ Angle	Bond lengths (Å) / º			
	Experimental (av. X-ray data)	BVP86 6-311+G*	PBEPBE 6-31G**	B3PW91 SDD
S1-C19	1.673	1.672	1.675	1.703
C19-N3	1.333	1.360	1.360	1.358
C19-N5	1.375	1.406	1.404	1.405
N3-C8	1.424	1.411	1.408	1.416
N5-N7	1.352	1.337	1.337	1.353
N7-C20	1.290	1.313	1.316	1.316
C21-O2	1.223	1.236	1.243	1.258
S1-C19-N3	128.56	130.61	130.67	130.55
N3-C19-N5	114.37	112.09	111.96	112.37
C19-N5-C7	119.36	121.52	121.17	120.32
N5-N7-C20	117.77	118.63	117.91	119.81
C20-C21-O1	125.34	125.33	125.29	124.69
C22-C21-O2	128.99	128.94	128.91	129.24

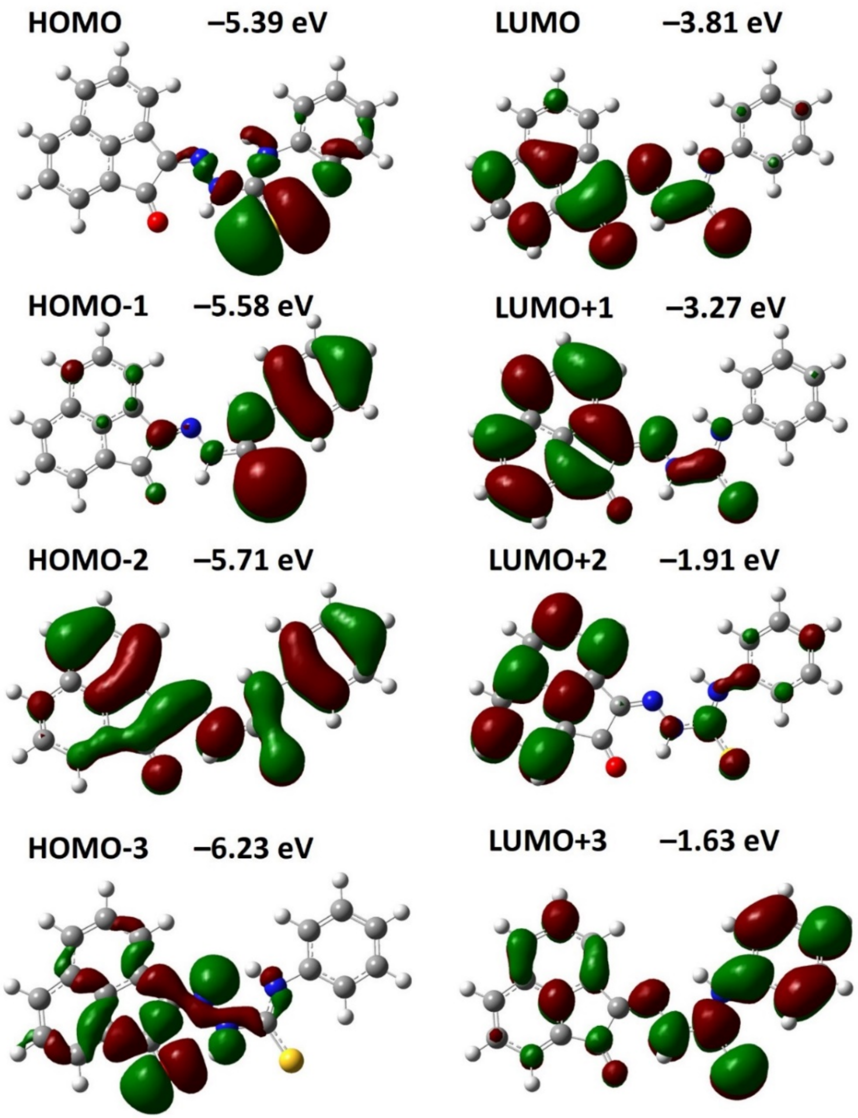

Figure S76. Shapes and energies of frontier orbitals for thiosemicarbazone AN-Ph structure optimized using the BVP86 exchange correlation functional and 6311+G* basis set.

Table S7. Selected bond lengths and bond angles for thiosemicarbazone ligand AN-Ph calculated using DFT with different functionals and basis sets

Bond/ Angle	Bond lengths (Å) / º			
	X-ray (average from polymorphs)	BVP86 6-311+G*	PBEPBE 6-31G**	B3PW91 SDD
S1-C19	1.673	1.672	1.675	1.703
C19-N3	1.333	1.360	1.360	1.358
C19-N5	1.375	1.406	1.404	1.405
N3-C8	1.424	1.411	1.408	1.416
N5-N7	1.352	1.337	1.337	1.353
N7-C20	1.290	1.313	1.316	1.316
C21-O2	1.223	1.236	1.243	1.258
S1-C19-N3	128.56	130.61	130.67	130.55
N3-C19-N5	114.37	112.09	111.96	112.37
C19-N5-C7	119.36	121.52	121.17	120.32
N5-N7-C20	117.77	118.63	117.91	119.81
C20-C21-O1	125.34	125.33	125.29	124.69
C22-C21-O2	128.99	128.94	128.91	129.24

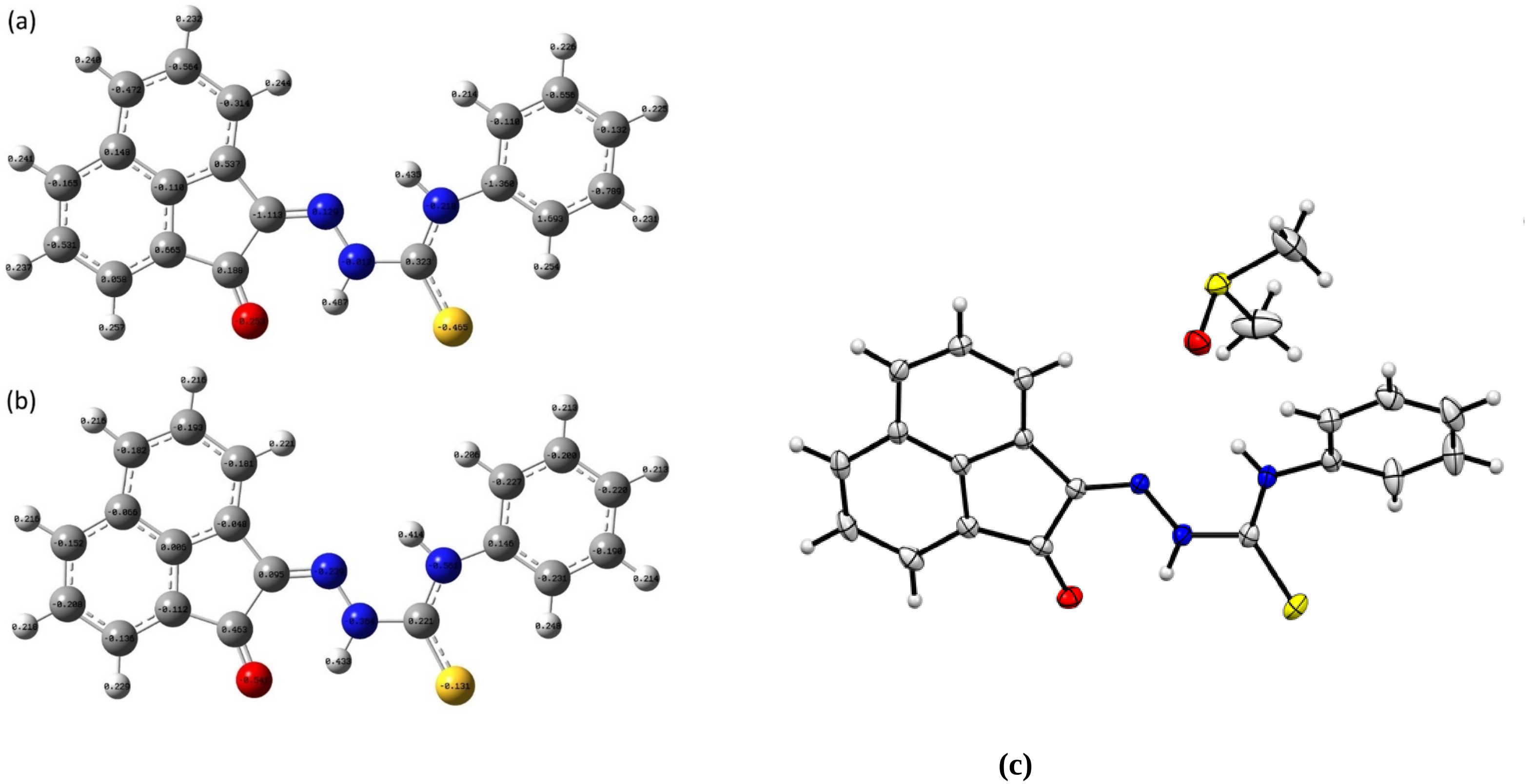

Figure S77 DFT calculated and experimental and structures for AN-Ph: (a) optimized structure showing the Mulliken and (b) natural bonding orbital charge distribution of AN-Ph. Structure was optimized using the BVP86 exchange correlation functional and 6311+G* basis set. (c) X-ray diffraction structure of AN.Ph co-crystallized with DMSO (CCDC 2218630)

Table S8. Selected bond lengths and angles for the DFT optimized structure of complex Cu(AN-Ph)$_2$

Bond	Bond length (Å)	Angle	Bond angles (°)
Cu1-S2	2.374	S2-Cu1-S3	107.76
Cu1–S3	2.402	S2-Cu1-N7	83.29
Cu1–N7	1.977	S3-Cu1-N11	82.75
Cu1-N11	1.974	N11-Cu1-O13	78.44
Cu1-O12	2.442	N7-Cu1-O12	78.47
Cu1-O13	2.463	Cu1-N7-N6	123.66
N6-N7	1.334	O12-Cu1-O13	77.66
N10-N11	1.327	Cu1-S3-C33	92.21
N6-C14	1.352	Cu1-S2-C14	92.36
N7-C15	1.325	Cu1-O12-C16	101.37
N11-C34	1.327	Cu1-O13-C35	100.99
C15-C16	1.492		
C35-C34	1.488		
C16-O12	1.245		
C35-O13	1.245		

Table S9. Selected bond lengths and angles for the optimized structure of the complex Zn(AN-Ph)$_2$

Bond	Bond length (Å)	Angle	Bond angles (°)
Zn1-S2	2.401	S2-Zn1-S3	103.82
Zn1–S3	2.431	S2-Zn1-N7	82.09
Zn1–N7	2.102	S3-Zn1-N11	81.18
Zn1-N11	2.098	N11-Zn1-O13	76.47
Zn1-O12	2.475	N7-Zn1-O12	75.20
Zn1-O13	2.405	Zn1-N7-N6	122.29
N6-N7	1.345	O12-Zn1-O13	82.81
N10-N11	1.330	Zn1-S3-C33	94.25
N6-C14	1.357	Zn1-S2-C14	93.74
N7-C15	1.310	Zn1-O12-C16	104.46
N11-C34	1.322	Zn1-O13-C35	105.23
C15-C16	1.490		
C35-C34	1.487		
C16-O12	1.244		
C35-O13	1.248		

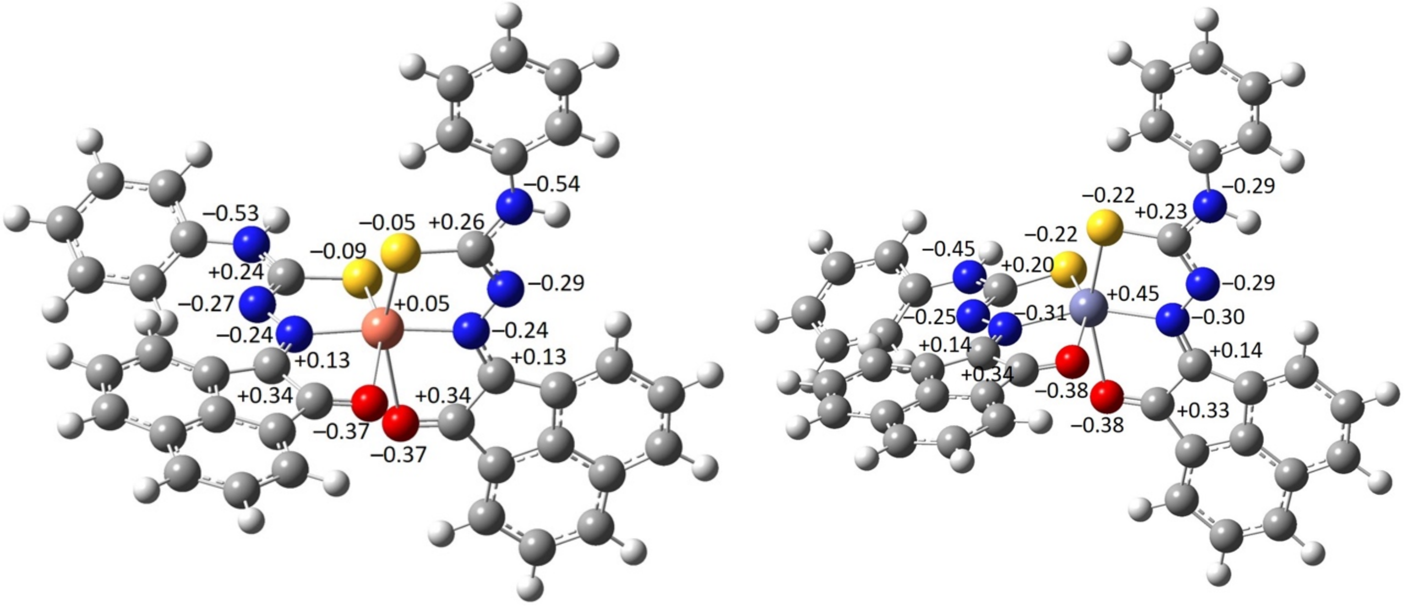

Figure S78 A comparison of the DFT optimized structures and Mulliken charges of $Cu(AN\text{-}Ph)_2$ (*Left*) and for $Zn(AN\text{-}Ph)_2$ (*Right*).

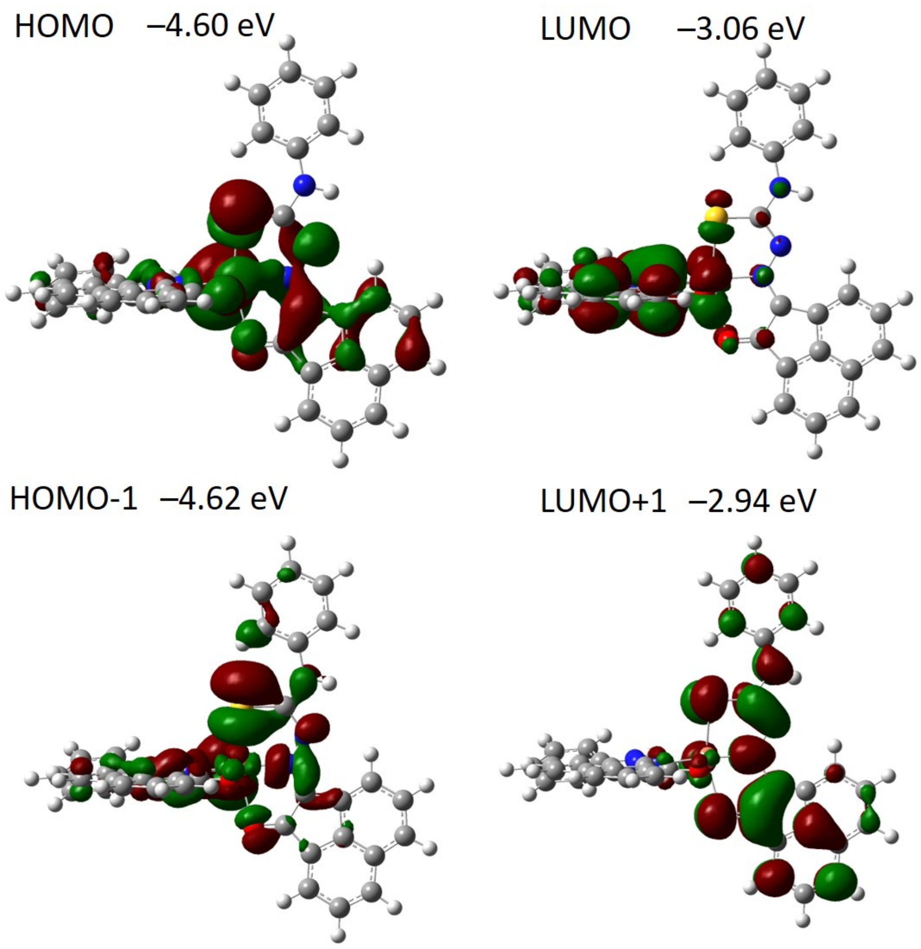

Figure S79 Shapes and energies of frontier orbitals (HOMO and LUMO) for Cu(AN-Ph)$_2$

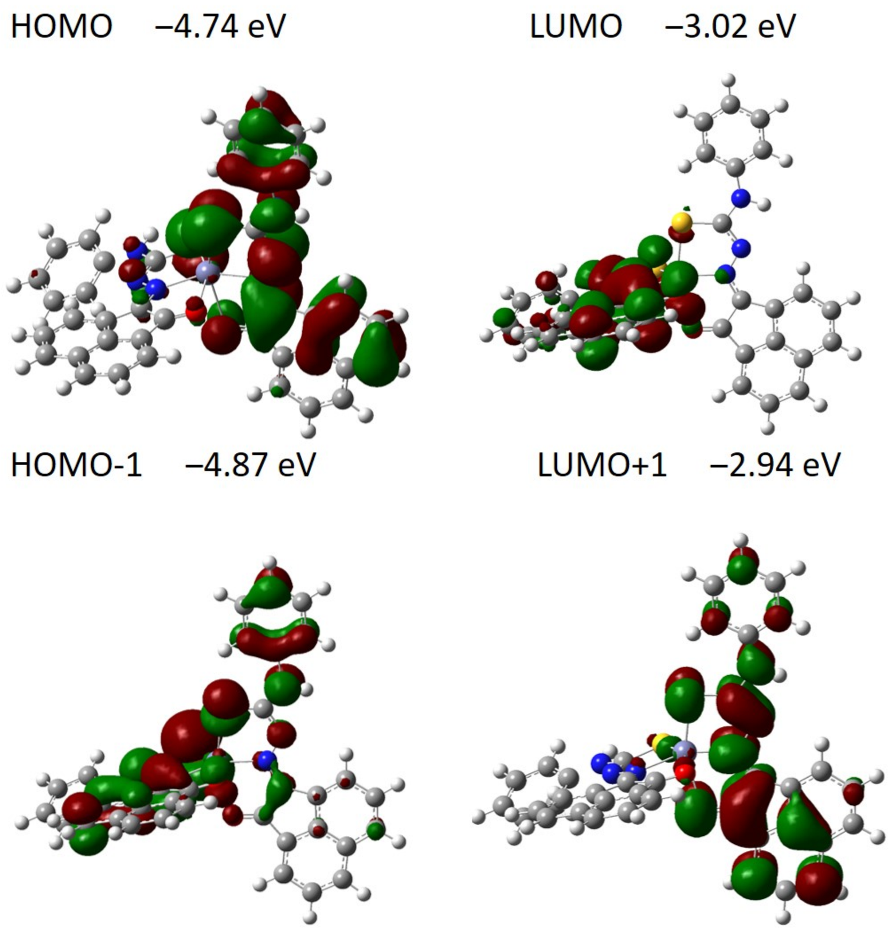

Figure S80 Shapes and energies of frontier orbitals (HOMO and LUMO) for Zn(AN-Ph)$_2$

Table S10. Functionals and basis sets used for different atoms in Zn(II) or Cu(II) metal complex modelled hereby.

Keyword	Functional	Basis set	
		Metal (Zn or Cu)	Non-metal (C, H, O, N, and S)
BVP86 SDD	BVP86	SDD	SDD
BVP86 LANL2DZ	BVP86	LANL2DZ	LANL2DZ
B3LYP LANL2MB	B3LYP	LANL2MB	LANL2MB
B3PW91 LANL2DZ	B3PW91	LANL2DZ	LANL2DZ
B3PW91 SDD	B3PW91	SDD	SDD
HSEH1PBE LANL2DZ	HSEH1PBE	LANL2DZ	LANL2DZ
MPW1PW91 LANL2DZ	MPW1PW91 LANL2DZ	LANL2DZ	LANL2DZ
PBEPBE SDD	PBEPBE	SDD	SDD
PBEPBE LANL2DZ	PBEPBE	LANL2DZ	LANL2DZ
PBEPBE LANL2TZ 631G**	PBEPBE	LANL2TZ	631G**
PBEPBE Def2 TZVPPD 631G**	PBEPBE	Def2 TZVPPD	631G**
PBEPBE aug- cc-PVTZ-pp 631G**	PBEPBE	aug- cc-PVTZ-pp	631G**

Table S11 Selected bond (Å) / angle (º) for Cu(AN-H)$_2$ calculated using DFT with different functionals and basis sets

	PBEPBE aug-cc-PVTZ-pp 6-31G**	PBEPBE Def2TZVPPD 6-31G**	PBEPBE LANL2TZ 631G**
Cu1-S2	2.388	2.407	2.443
Cu1-S3	2.388	2.407	2.443
Cu1-N8	1.976	1.998	1.999
Cu1-N13	1.976	1.998	1.999
Cu1-O14	2.484	2.465	2.428
Cu1-O15	2.484	2.463	2.429
N7-N8	1.332	1.359	1.328
N12-N13	1.332	1.359	1.328
S2-C16	1.726	1.725	1.725
S3-C35	1.726	1.725	1.725
C18-O14	1.243	1.243	1.244
C37-O15	1.244	1.243	1.244
S2-Cu1-S3	109.18	108.64	107.20
S3-Cu1-N13	83.31	82.50	81.98
O15-Cu1-N13	78.08	78.00	78.78
O14-Cu1-O15	76.95	77.73	78.16
N8-Cu1-S2	83.35	82.50	81.99
Cu1-O15-C37	100.85	101.44	101.76
Cu1-O14-C18	100.76	101.44	101.75
Cu1-S3-C35	81.68	91.96	91.44
Cu1-S2-C16	91.53	91.98	91.43
Cu1-N8-N7	122.98	123.49	123.95
Cu1-N13-N12	123.04	123.51	124.01

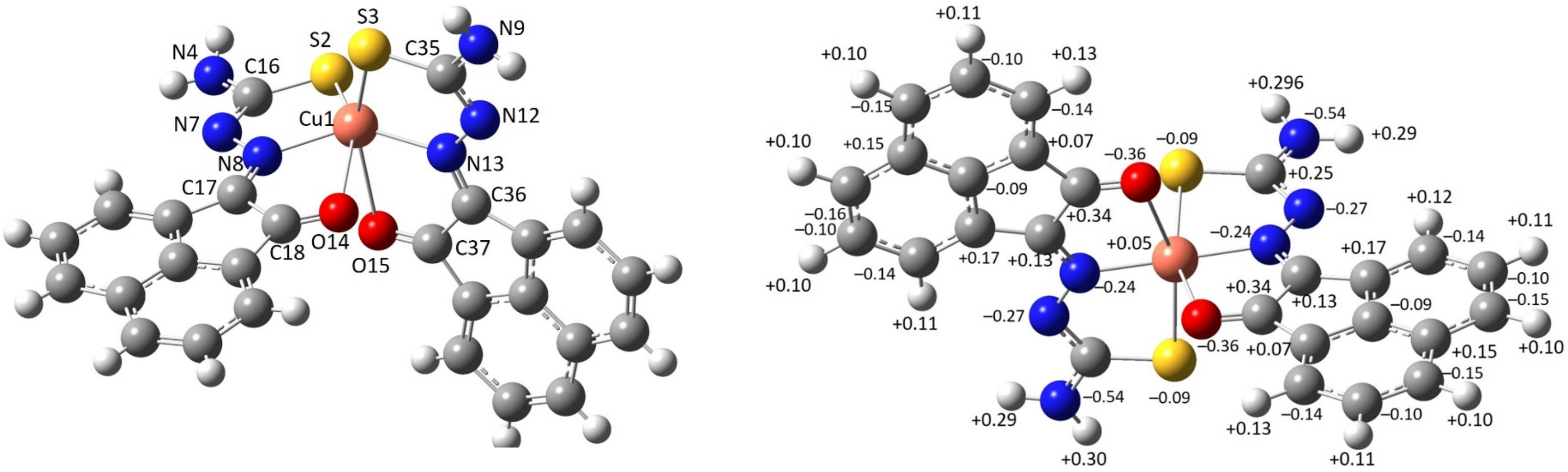

Figure S81 Different views of the DFT optimized relaxed structure of Cu(AN-H)$_2$] and corresponding Mulliken charges of the model complex Cu(AN-H)$_2$ using PBEPBE exchange correlation function together with a basis set aug- cc-PVTZ pp/ 6-31G**.

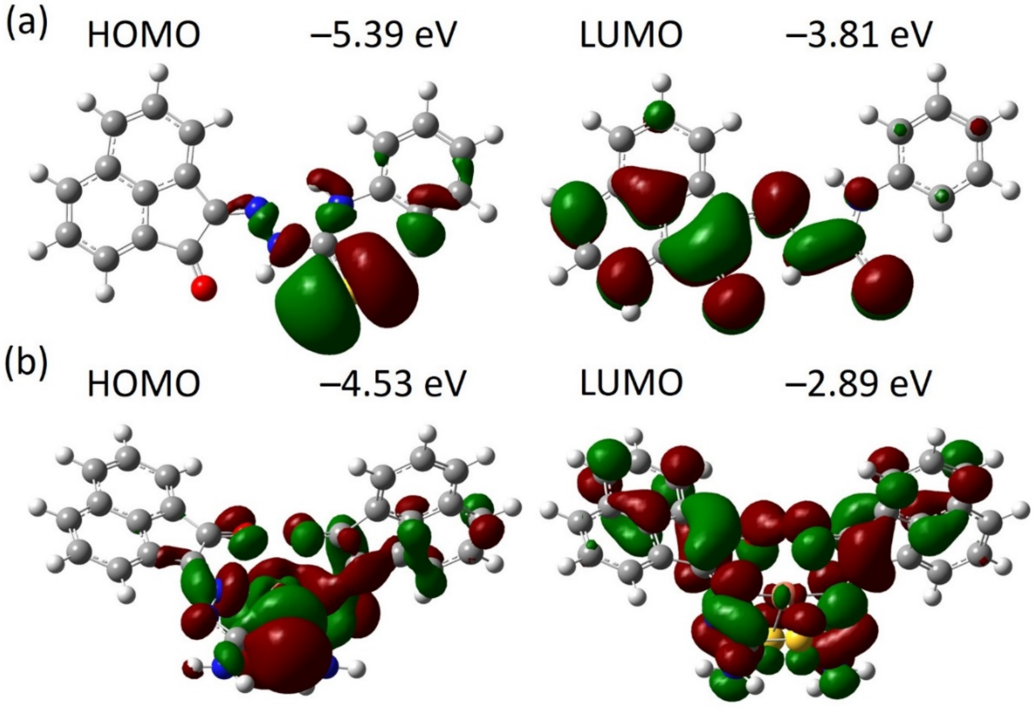

Figure S82. Shapes and energies of frontier orbitals (HOMO and LUMO) for the model Cu-thiosemicarbazone complex Cu(AN-H)$_2$. Additional frontiers orbitals and their energies are given below. Structures optimised using the PBEPBE exchange correlation functional and aug- cc-PVTZ pp/ 6-31G** basis set.

Table S12 Selected bond lengths and bond angles for the modelled Cu(II) complex of AN-H: DFT calculations using different functionals and basis sets..

Bond	Bond length (Å)		Angle	Bond angles (°)	
	aug- cc-PVTZ pp/ 6-31G**	Def2TZVPPD 631G**		aug- cc-PVTZ pp/ 6-31G**	Def2TZVPPD 631G**
Cu1-S2	2.388	2.407	S2-Cu1-S3	109.18	108.64
Cu1–S3	2.388	2.407	S3-Cu1-N13	83.31	82.50
Zn1–N8	1.976	1.998	O15-Cu1-N13	78.08	78.00
Cu1-N13	1.976	1.998	O14-Cu1-O15	76.95	77.73
Cu1-O14	2.484	2.465	N8-Cu1-S2	83.35	82.50
Cu1-O15	2.484	2.463	Cu1-O15-C37	100.85	101.44
N7-N8	1.332	1.359	Cu1-O14-C18	100.76	101.44
N12-N13	1.332	1.359	Cu1-S3-C35	81.68	91.96
S2-C16	1.726	1.725	Cu1-S2-C16	91.53	91.98
S3-C35	1.726	1.725	Cu1-N8-N7	122.98	123.49
C18-O14	1.243	1.243	Cu1-N13-N12	123.04	123.51
C37-O15	1.244	1.243			

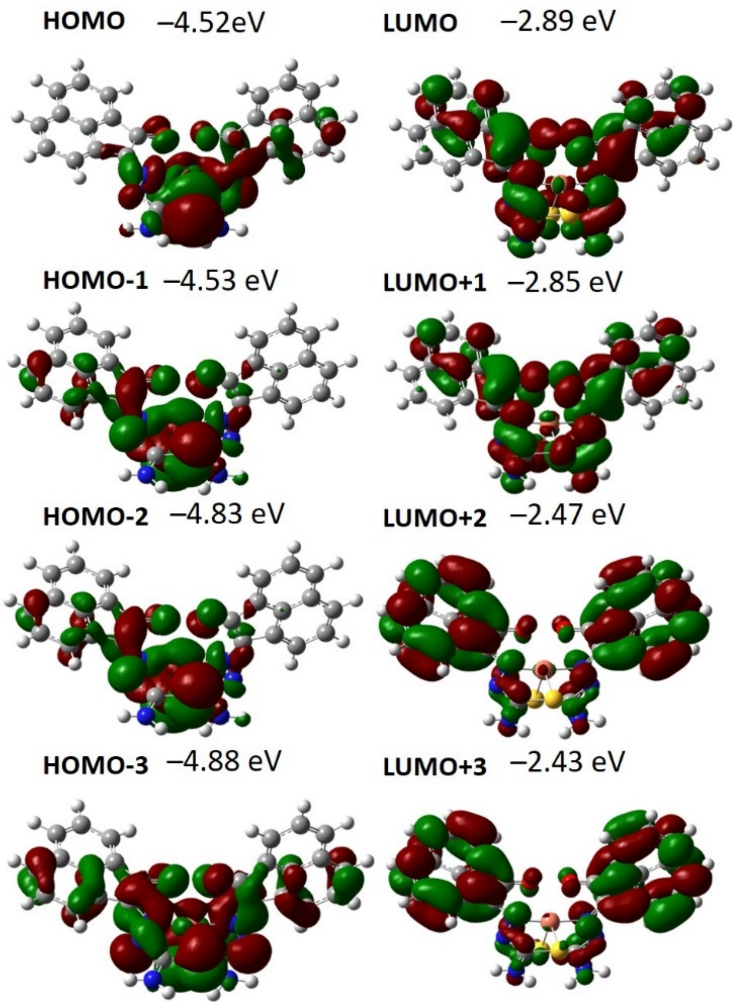

Figure S83 DFT calculated shapes and energies of frontier orbitals for the Cu(AN-H)$_2$ structure, optimized using the PBEPBE exchange correlation functional and aug-cc-PVTZ-pp/631G** basis set.

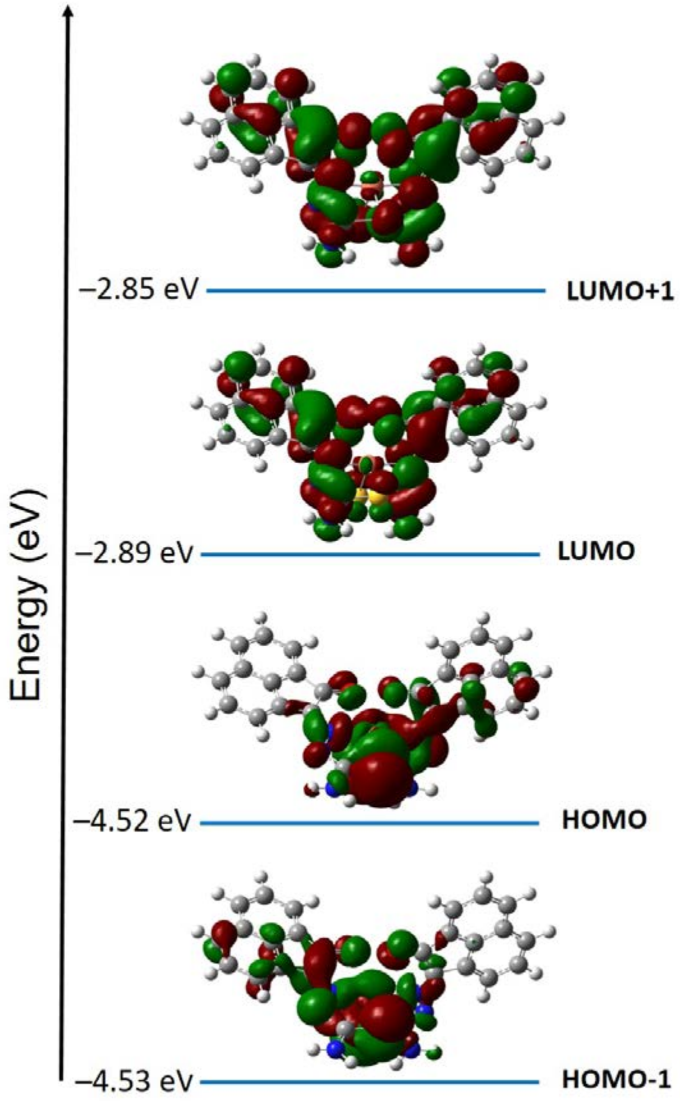

Figure S84 DFT calculated relevant energy levels likely involved in UV-Vis/Fluorescence spectroscopy. Structures were optimized using the PBEPBE exchange correlation functional and aug-cc-PVTZ-pp/631G** basis set.

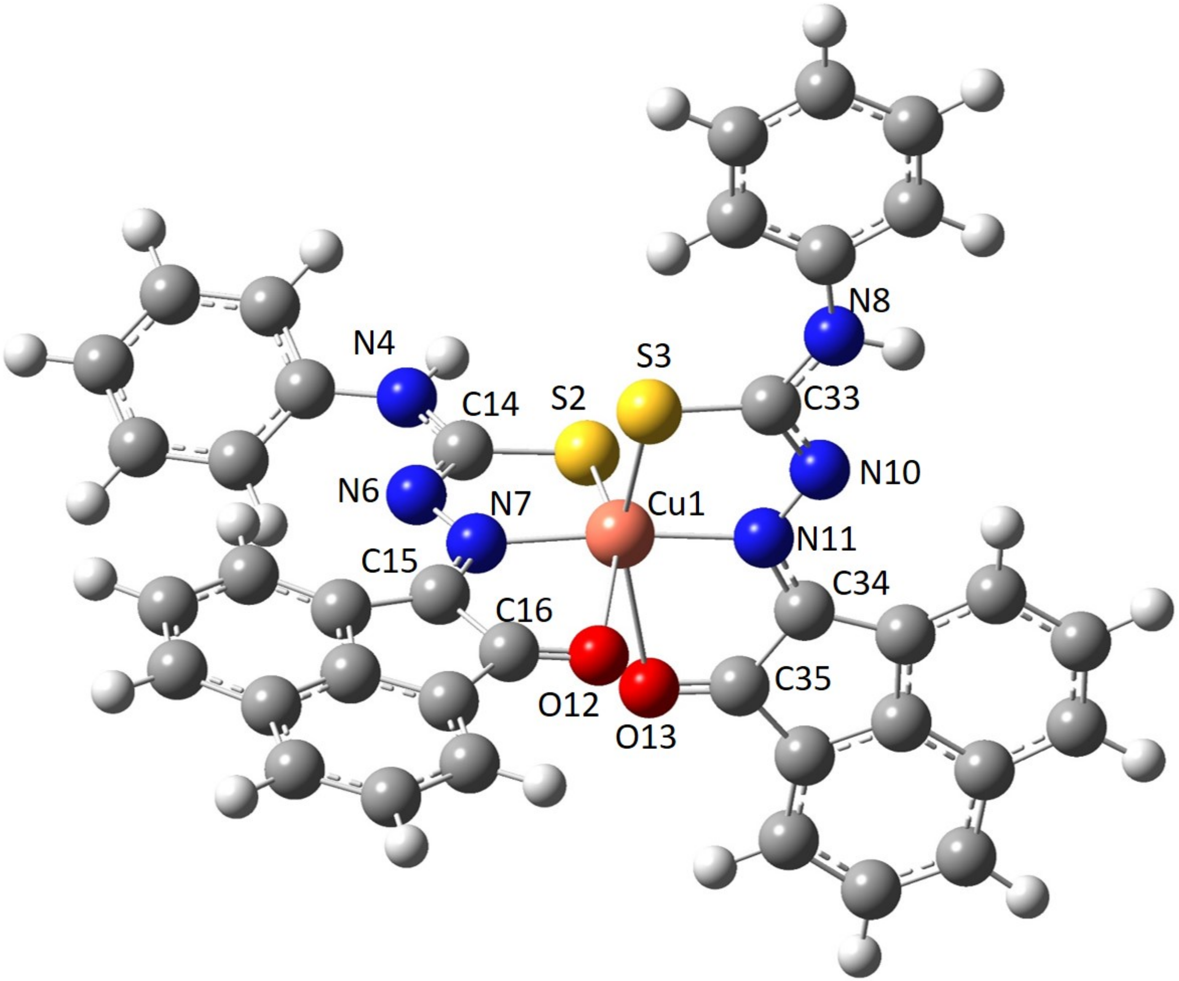

Figure S85 DFT optimization: the relaxed structure of the Cu(AN-Ph)$_2$ thiosemicarbazone complex.

Table S13 Selected bond lengths and angles for the optimized structure of the $Cu(AN\text{-}Ph)_2$ thiosemicarbazone complex shown in Figure S85, optimized using the PBEPBE exchange correlation functional and aug-cc-PVTZ-pp/631G** basis set.

Bond	Bond length (Å)	Angle	Bond angles (°)
Cu1-S2	2.374	S2-Cu1-S3	107.76
Cu1–S3	2.402	S2-Cu1-N7	83.29
Cu1–N7	1.977	S3-Cu1-N11	82.75
Cu1-N11	1.974	N11-Cu1-O13	78.44
Cu1-O12	2.442	N7-Cu1-O12	78.47
Cu1-O13	2.463	Cu1-N7-N6	123.66
N6-N7	1.334	O12-Cu1-O13	77.66
N10-N11	1.327	Cu1-S3-C33	92.21
N6-C14	1.352	Cu1-S2-C14	92.36
N7-C15	1.325	Cu1-O12-C16	101.37
N11-C34	1.327	Cu1-O13-C35	100.99
C15-C16	1.492		
C35-C34	1.488		
C16-O12	1.245		
C35-O13	1.245		

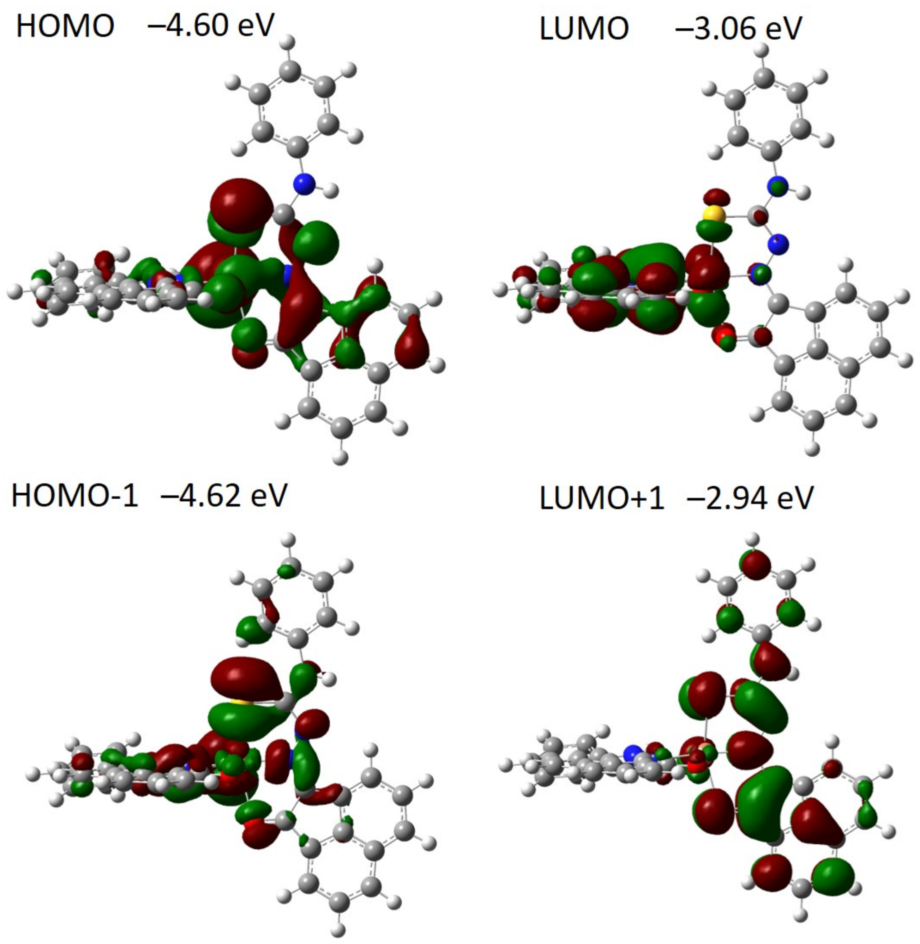

Figure S86 DFT calculated shapes and energies of frontier orbitals for the $Cu(AN\text{-}Ph)_2$ structure, optimized using the PBEPBE exchange correlation functional and aug-cc-PVTZ-pp/631G** basis set.

Table S14. The functionals and basis sets used for different atoms in Zn(II) and Cu(II) model metal complexes

Method	Functional	Basis set	
		Metal (Zn or Cu)	Non-metal (C, H, O, N, and S)
BVP86 SDD	BVP86	SDD	SDD
BVP86 LANL2DZ	BVP86	LANL2DZ	LANL2DZ
B3LYP LANL2MB	B3LYP	LANL2MB	LANL2MB
B3PW91 LANL2DZ	B3PW91	LANL2DZ	LANL2DZ
B3PW91 SDD	B3PW91	SDD	SDD
HSEH1PBE LANL2DZ	HSEH1PBE	LANL2DZ	LANL2DZ
MPW1PW91 LANL2DZ	MPW1PW91	LANL2DZ	LANL2DZ
PBEPBE SDD	PBEPBE	SDD	SDD
PBEPBE LANL2DZ	PBEPBE	LANL2DZ	LANL2DZ
PBEPBE LANL2TZ 631G**	PBEPBE	LANL2TZ	631G**
PBEPBE Def2 TZVPPD 631G**	PBEPBE	Def2 TZVPPD	631G**
PBEPBE aug- cc-PVTZ-pp 631G**	PBEPBE	aug- cc-PVTZ-pp	631G**

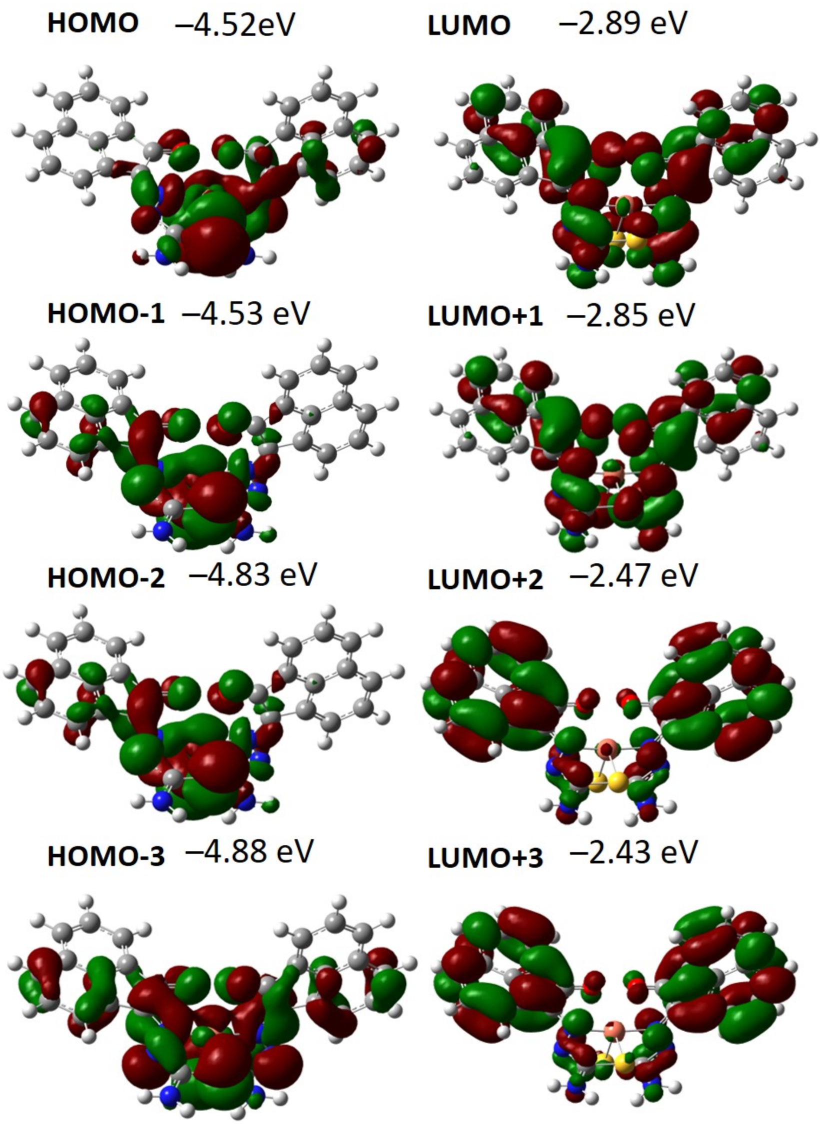

Figure S87 DFT calculated shapes and energies of frontier orbitals for the model $Cu(AN\text{-}H)_2$ complex: structure optimized using the PBEPBE exchange correlation functional and aug-cc-PVTZ-pp/631G** basis set.

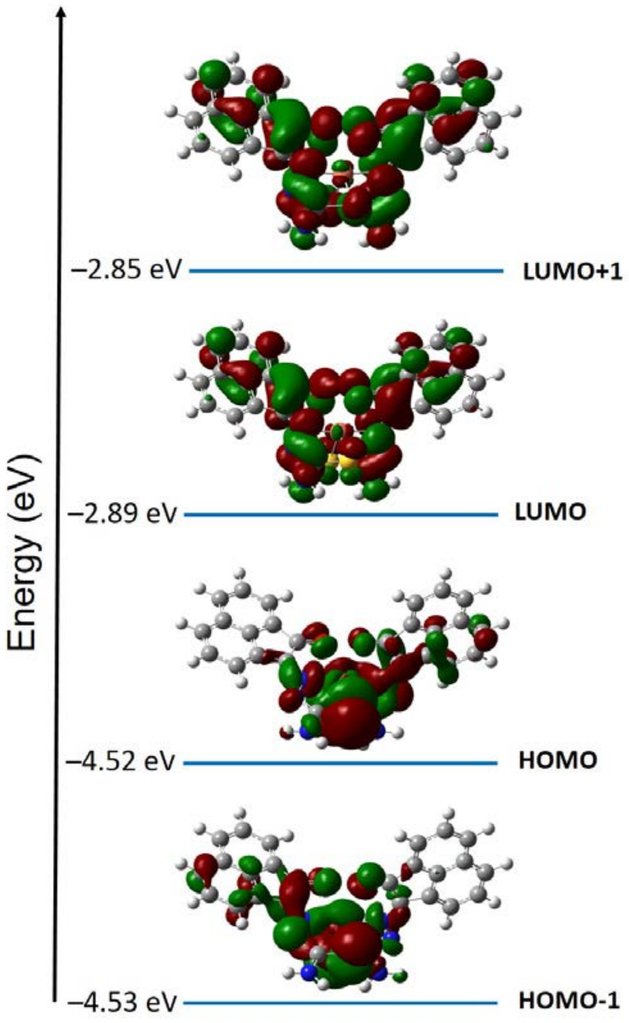

Figure S88 DFT calculated relevant energy levels likely involved in UV-Vis/Fluorescence spectroscopy for Cu(AN-H)$_2$ theoretical complex Structures optimised using the PBEPBE exchange correlation functional and aug-cc-PVTZ-pp/631G** basis set.

.

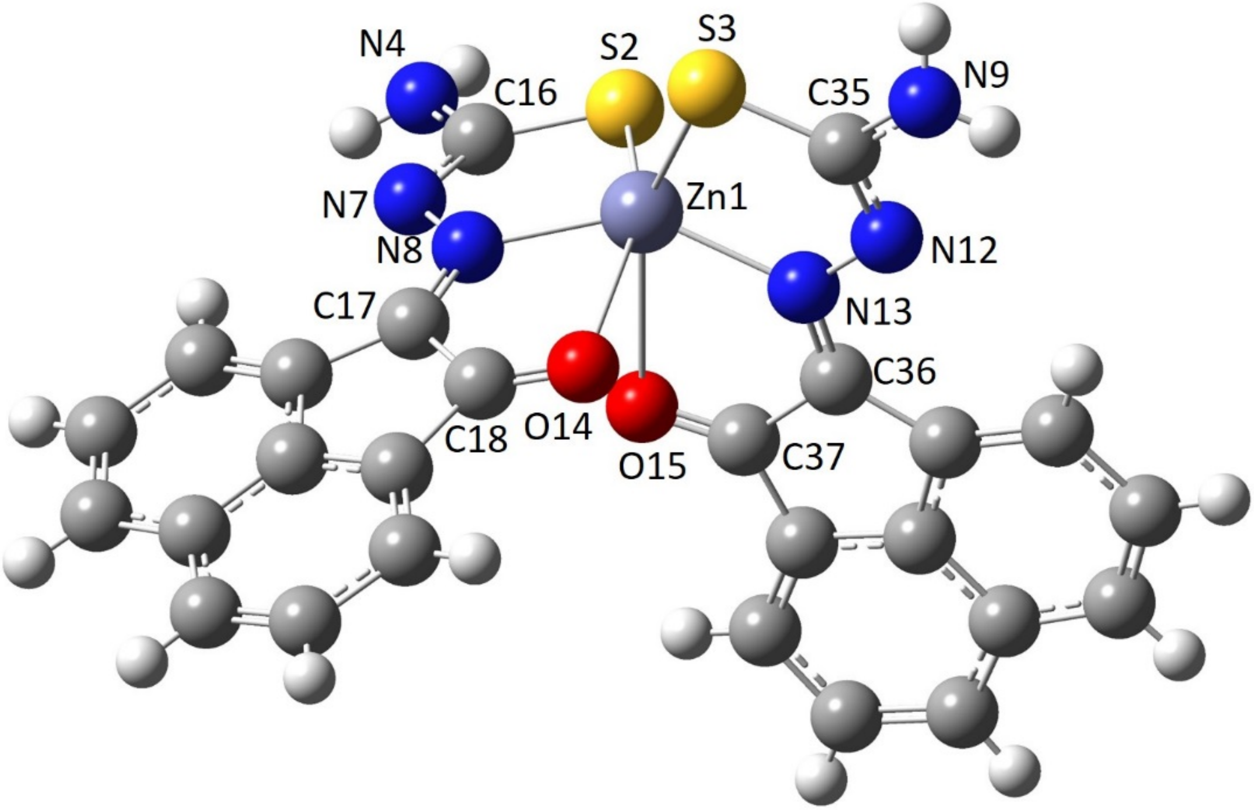

Figure S89 DFT Calculated molecular structure of $Zn(AN\text{-}H)_2$

Table S15 Selected bond lengths (Å) and bond angles (º) for $Zn(AN\text{-}H)_2$ calculated using DFT with different functionals and basis sets

	X-ray	PBEPBE aug-cc-PVTZ-pp 631G**	PBEPBE Def2TZVPPD 6-31G**	B3PW91 SDD
Zn1-S2	2.335	2.412	2.442	2.466
Zn1-S3	2.360	2.412	2.442	2.466
Zn1-N8	2.050	2.102	2.134	2.095
Zn1-N13	2.052	2.102	2.135	2.096
Zn1-O14	2.651	2.473	2.393	2.365
Zn1-O15	2.588	2.473	2.394	2.364
N7-N8	1.353	1.327	1.324	1.344
N12-N13	1.351	1.327	1.324	1.344
S2-C16	1.720	1.724	1.723	1.754
S3-C35	1.722	1.724	1.724	1.754
C18-O14	1.225	1.245	1.247	1.261
C37-O15	1.216	1.245	1.247	1.261
S2-Zn1-S3	110.52	104.94	103.38	102.22
S3-Zn1-N13	83.89	82.20	80.67	81.91
O15-Zn1-N13	73.45	75.35	76.02	76.96
O14-Zn1-O15	78.22	81.05	81.42	81.54
N8-Zn1-S2	84.47	82.18	80.69	81.92
Zn1-O15-C37	102.59	104.47	106.20	106.06
Zn1-O14-C18	102.87	104.52	106.17	106.09
Zn1-S3-C35	93.62	93.29	93.98	92.65
Zn1-S2-C16	93.24	93.26	93.97	92.68
Zn1-N8-N7	120.39	122.18	123.16	123.03
Zn1-N13-N12	120.58	122.14	123.15	123.04

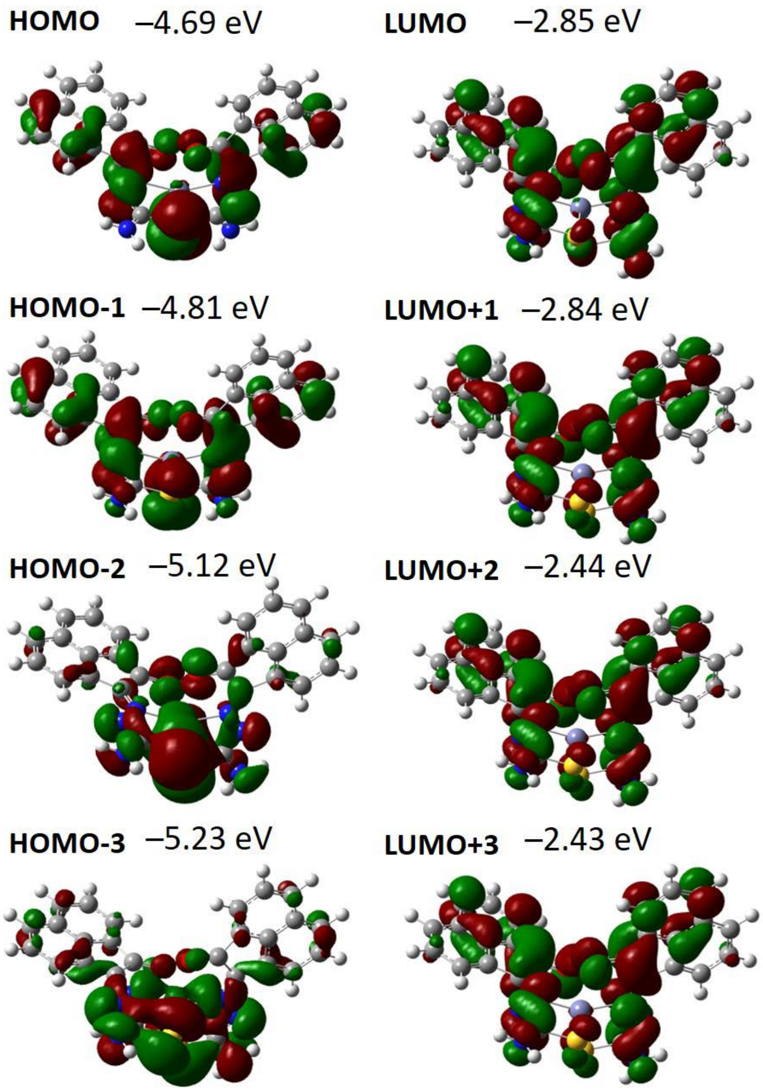

Figure S90 DFT calculated shapes and energies of frontier orbitals for Zn.thiosemicarbazone structures optimised using the PBEPBE exchange correlation functional and aug-cc-PVTZ-pp/631G** basis set.

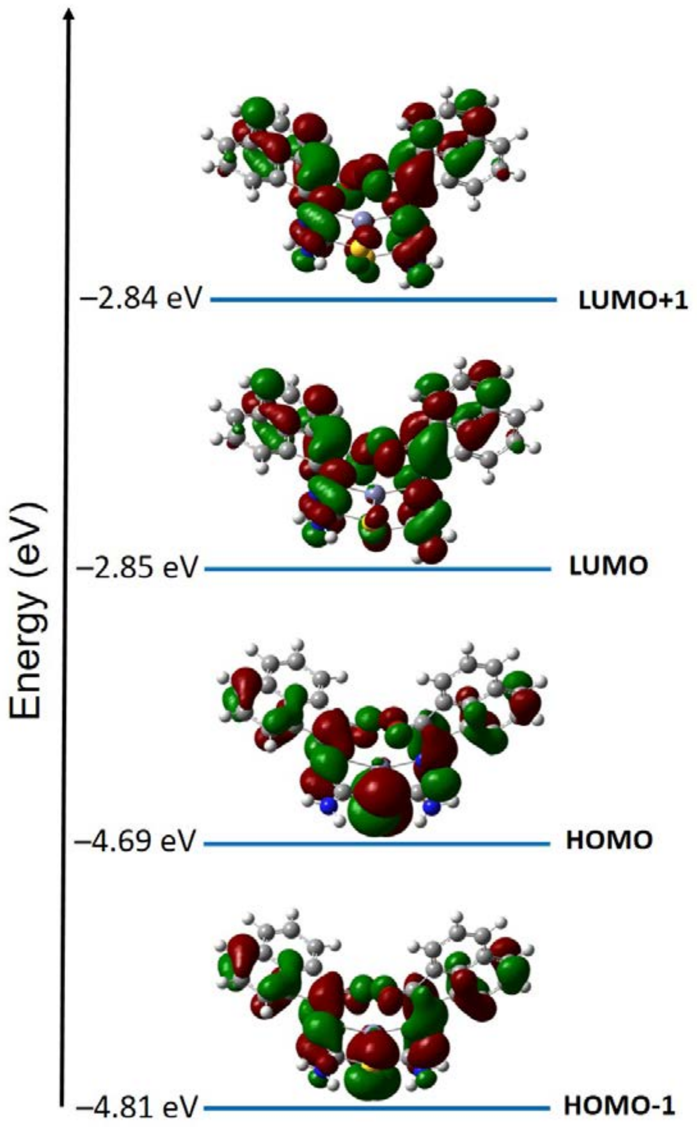

Figure S91 DFT calculated relevant energy levels likely involved in UV-Vis/Fluorescence spectroscopy. Structure was optimised using the PBEPBE exchange correlation functional and aug-cc-PVTZ-pp/631G** basis set.

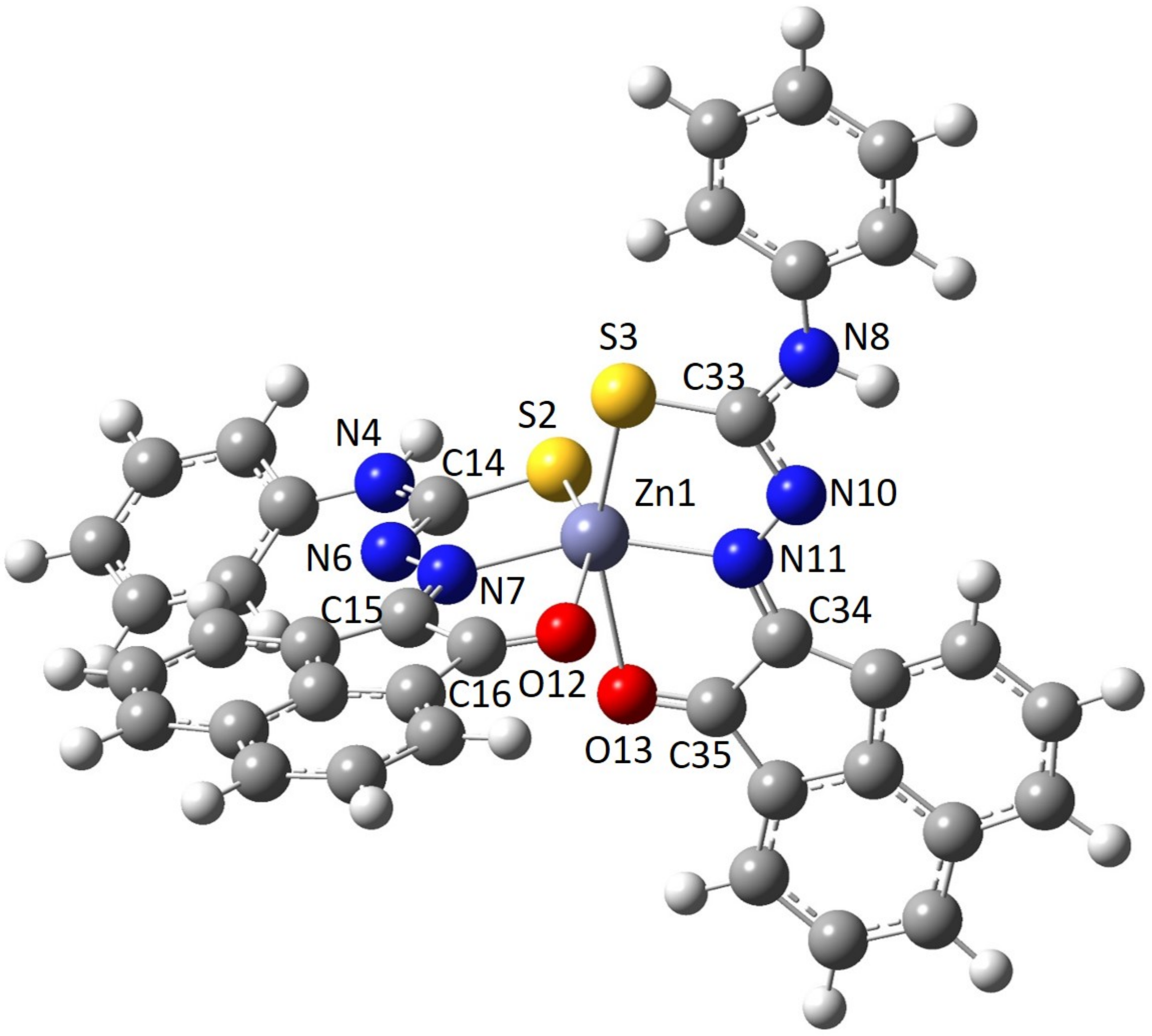

Figure S92 Relaxed structure of the model Zn(AN-Ph)$_2$ thiosemicarbazone. Structure was optimised using the PBEPBE exchange correlation functional and aug-cc-PVTZ-pp/631G** basis set.

.

Table S16 Selected bond lengths and angles for the optimized structure of the complex $Zn(AN\text{-}Ph)_2$

Bond	Bond length (Å)	Angle	Bond angles (°)
Zn1-S2	2.401	S2-Zn1-S3	103.82
Zn1–S3	2.431	S2-Zn1-N7	82.09
Zn1–N7	2.102	S3-Zn1-N11	81.18
Zn1-N11	2.098	N11-Zn1-O13	76.47
Zn1-O12	2.475	N7-Zn1-O12	75.20
Zn1-O13	2.405	Zn1-N7-N6	122.29
N6-N7	1.345	O12-Zn1-O13	82.81
N10-N11	1.330	Zn1-S3-C33	94.25
N6-C14	1.357	Zn1-S2-C14	93.74
N7-C15	1.310	Zn1-O12-C16	104.46
N11-C34	1.322	Zn1-O13-C35	105.23
C15-C16	1.490		
C35-C34	1.487		
C16-O12	1.244		
C35-O13	1.248		

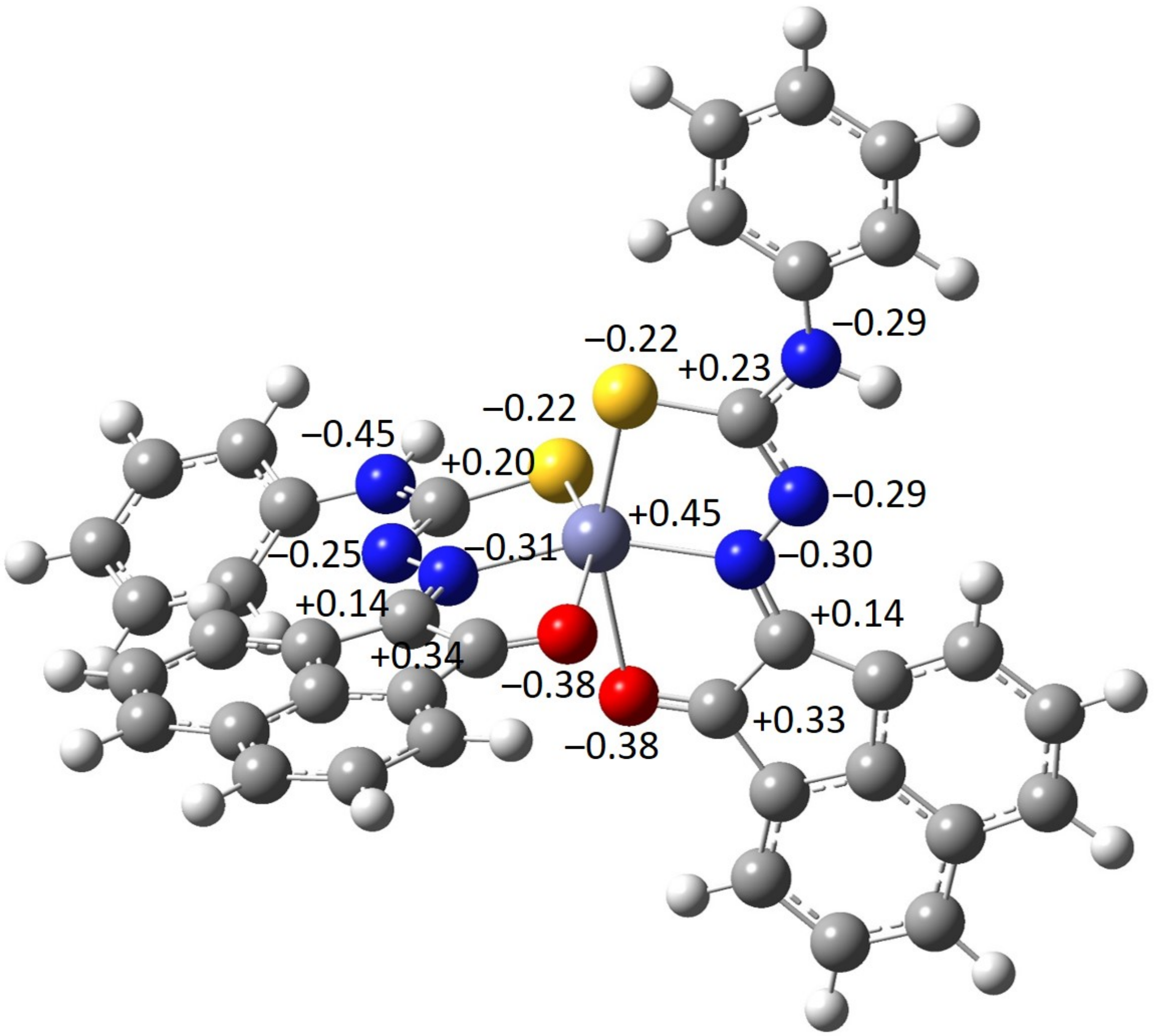

Figure S93 Mulliken charges calculated for the optimised thiosemicarbazone complex $Zn(AN\text{-}Ph)_2$

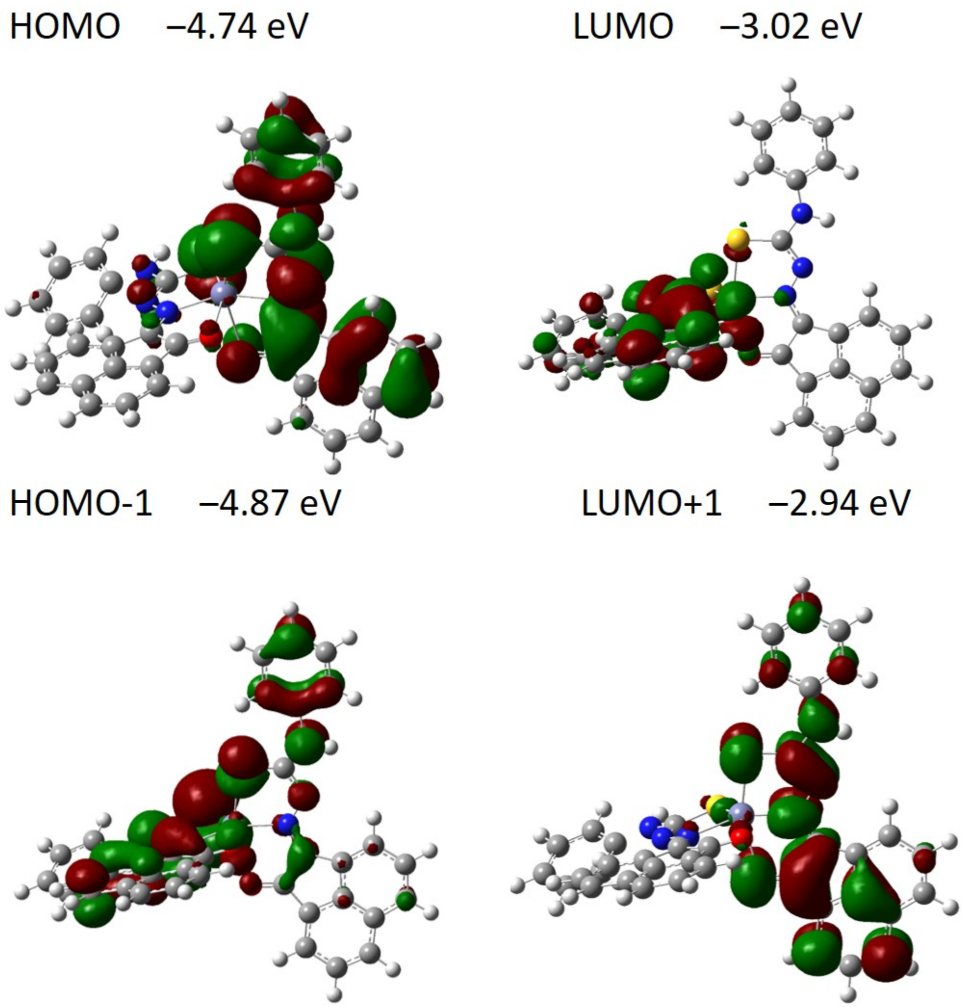

Figure S94 Shapes and energies of frontier molecular orbitals (HOMO and LUMO) for Zn(AN-Ph)$_2$ model complex, optimised using the PBEPBE exchange correlation functional and aug-cc-PVTZ-pp/631G** basis set.

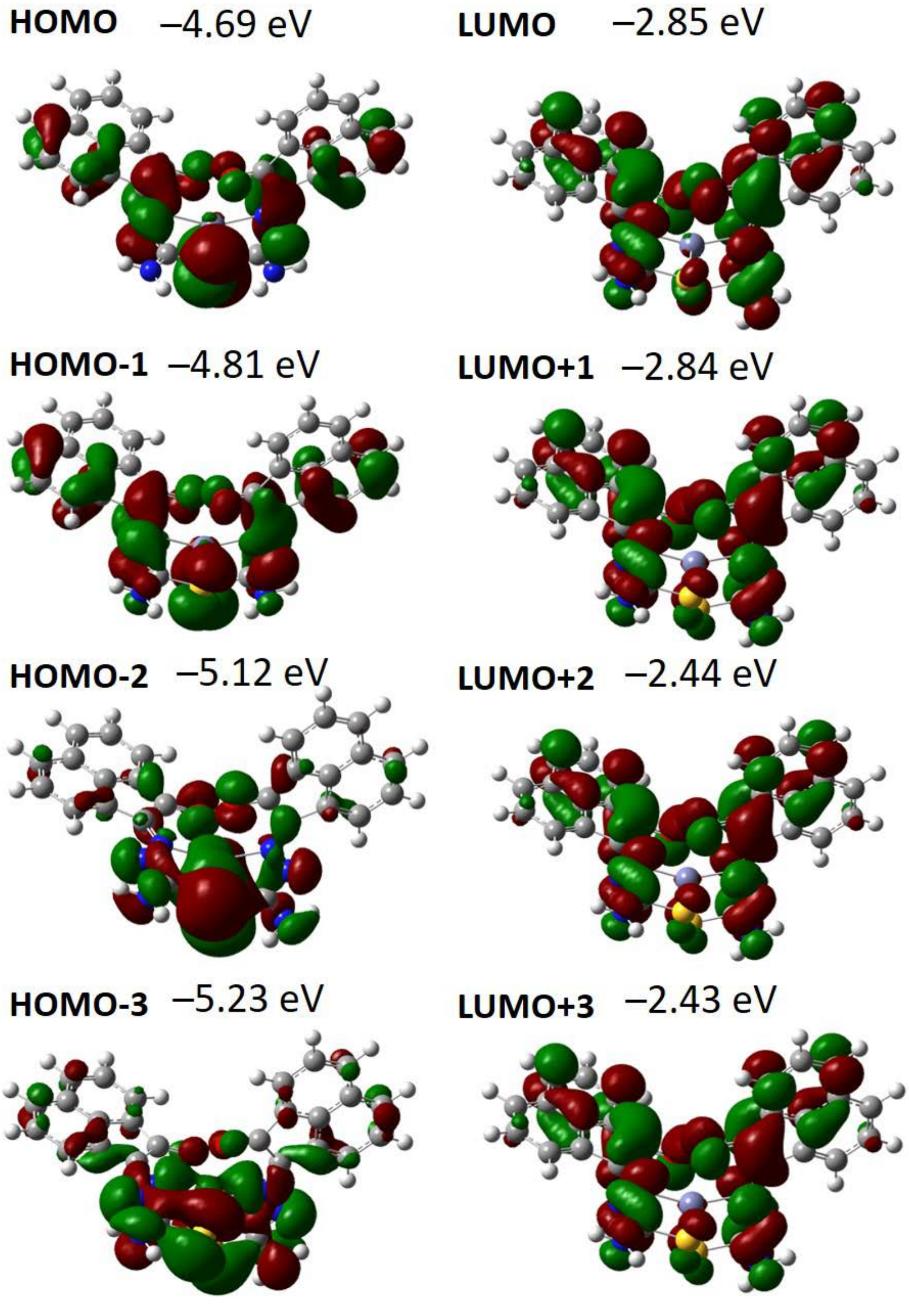

Figure S95 DFT calculated shapes and energies of frontier orbitals for $Zn(AN\text{-}H)_2$ modle complex optimised using the PBEPBE exchange correlation functional and aug-cc-PVTZ-pp/631G** basis set.

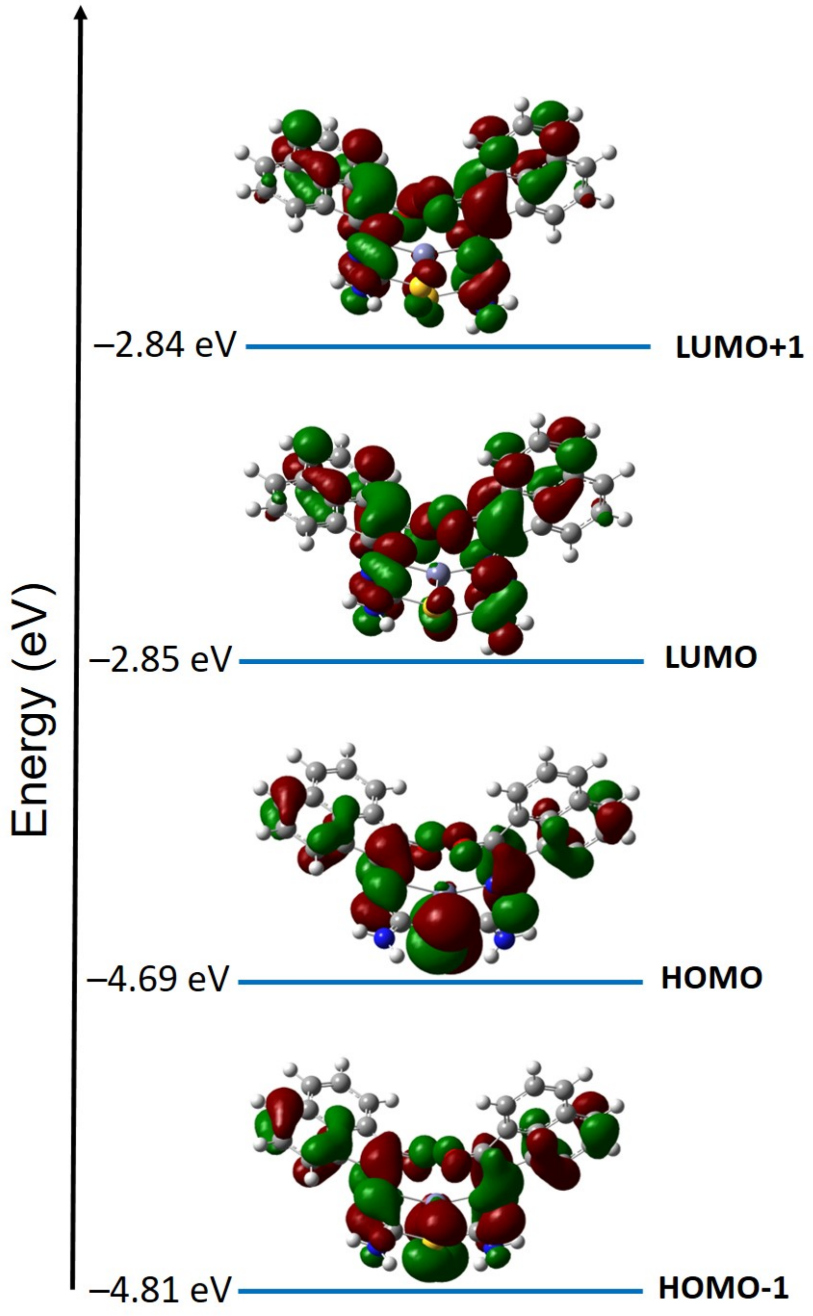

Figure S96 DFT calculated relevant energy levels likely involved in the model UV-Vis/Fluorescence spectroscopy of Zn(AN-H)$_2$. Structures optimised using the PBEPBE exchange correlation functional and aug-cc-PVTZ-pp/631G** basis set.

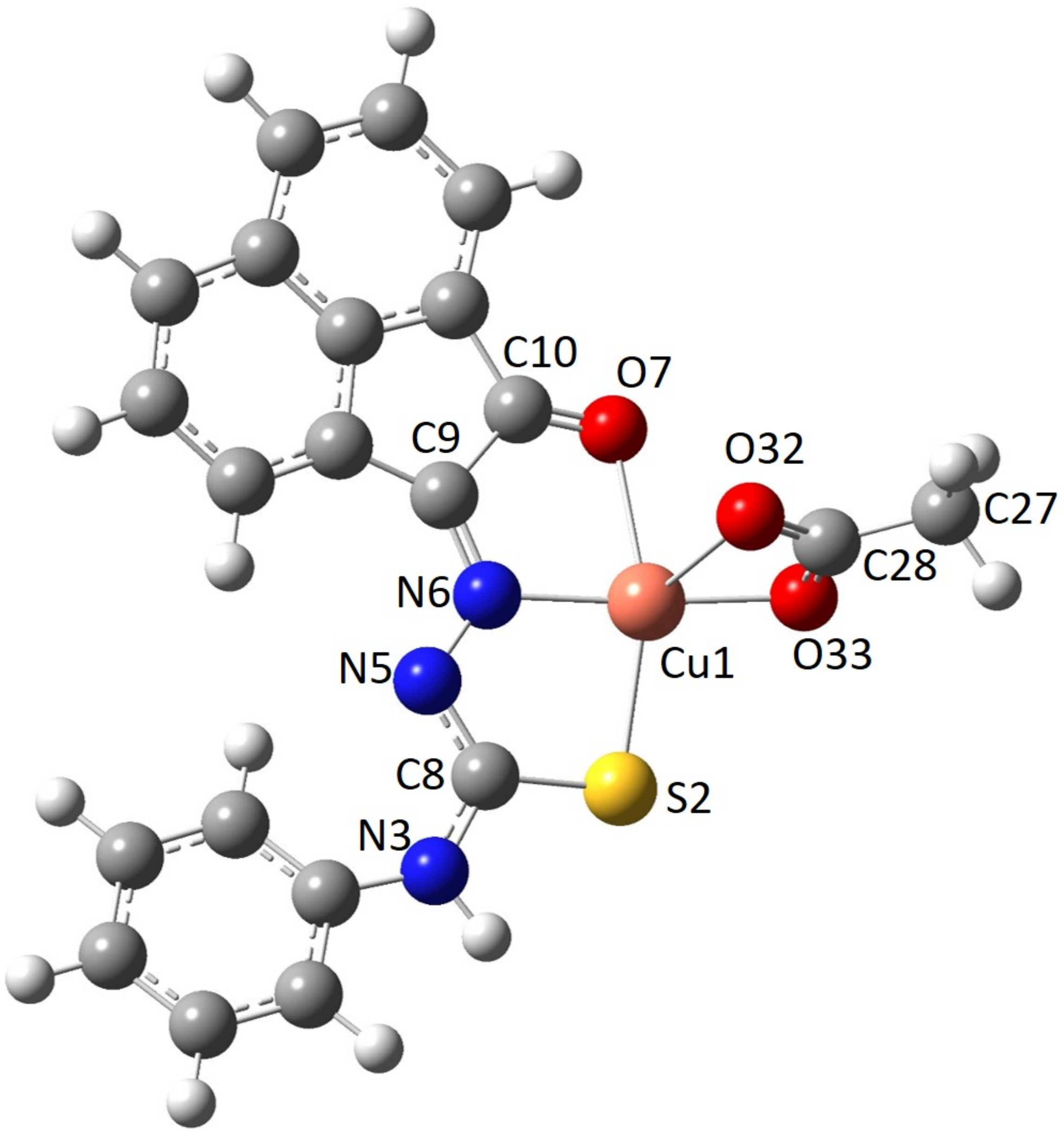

Figure S97 DFT calculated ‘relaxed’ structure of the postulated complex [Cu(AN-Ph)(OAc)]

Table S17 DFT-calculated, selected bond lengths and angles for the optimised complex of the postulated [Cu(AN-Ph)(OAc)] complex.

Bond	Bond length (Å)	Angle	Bond angles (°)
Cu1-S2	2.292	N6-Cu1-O7	80.87
Cu1–N6	1.965	N6-Cu1-S2	84.05
Cu1–O7	2.245	N6-Cu1-O32	175.83
Cu1-O32	2.256	N6-Cu1-O33	114.04
Cu1-O33	1.944	O7-Cu1-O32	95.43
S2-C8	1.744	O7-Cu1-S2	156.07
C8-N3	1.368	O32-Cu1-O33	63.40
C8-N5	1.354	O32-C28-O33	119.89
N5-N6	1.333	Cu1-N6-N5	123.91
N6-C9	1.322	Cu1-N6-C9	114.58
C9-C10	1.486	Cu1-O7-C10	103.99
C10-O7	1.252		
C28-O32	1.302		
C28-O33	1.265		

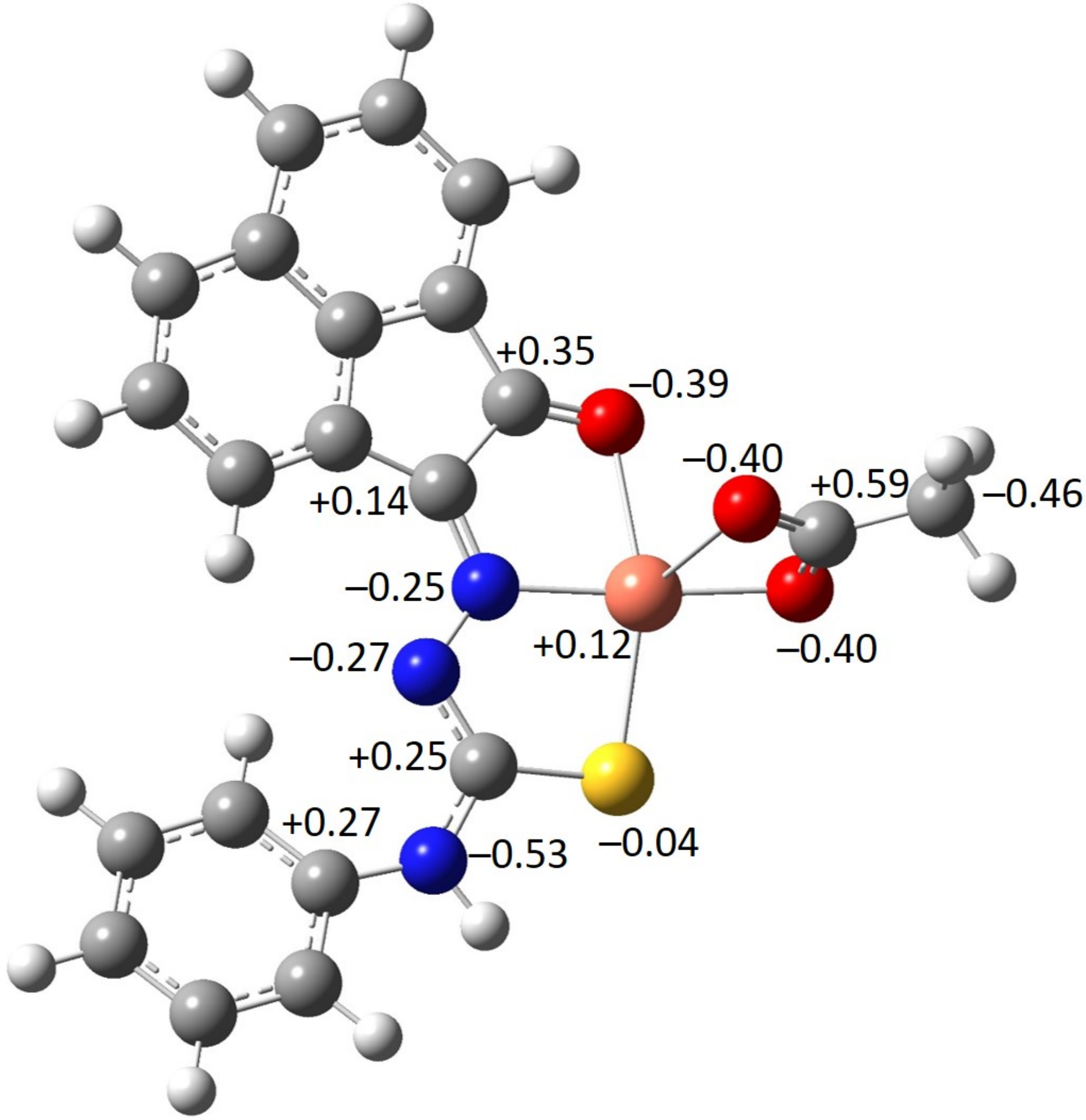

Figure S98. Mulliken charges in the postulated complex [Cu(AN-Ph)(OAc)]

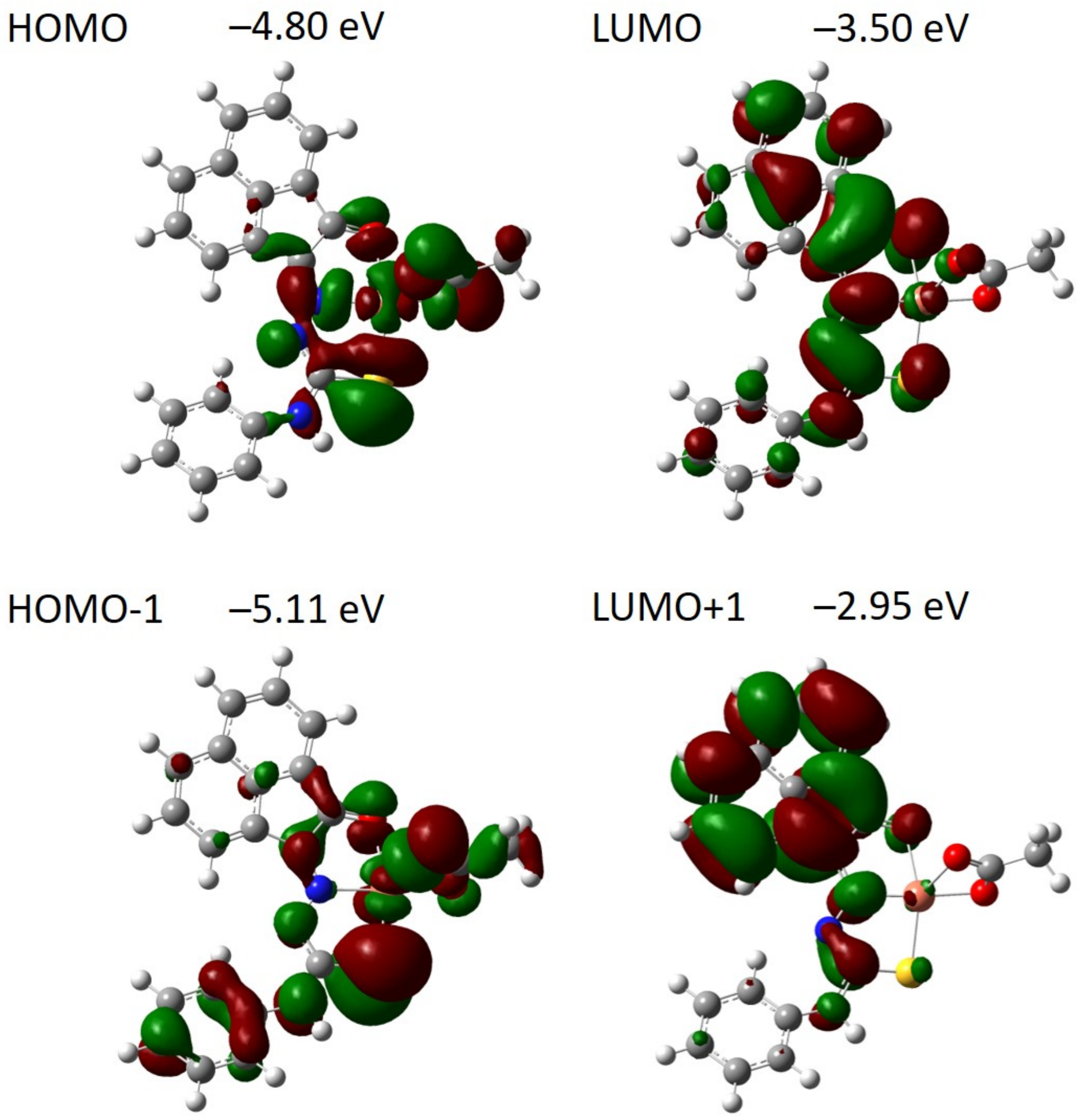

Figure S99 Shapes and energies of frontier orbitals (HOMO and LUMO) for the postulated complex [Cu(AN-Ph)(OAc)]

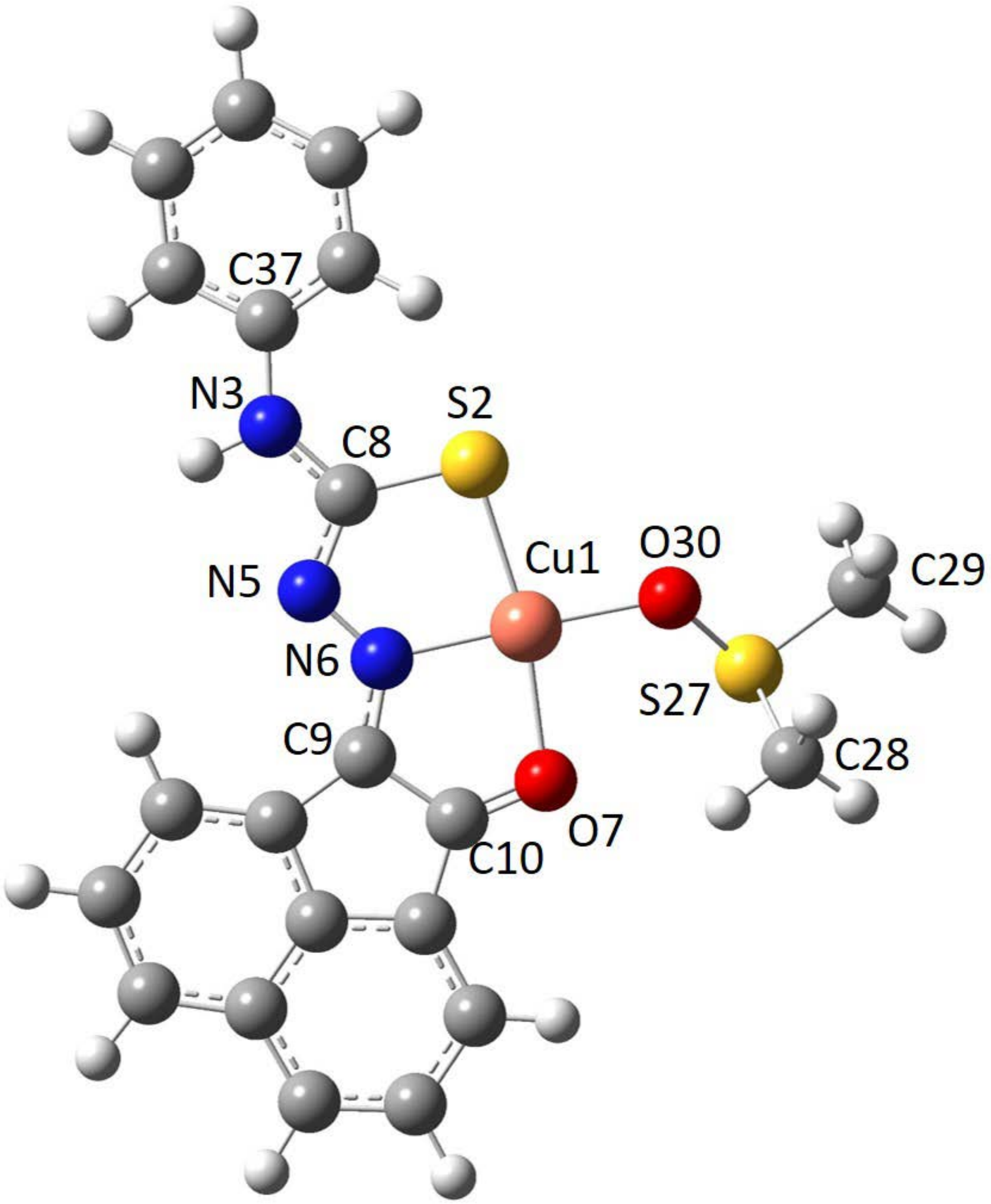

Figure S100 Relaxed structure of for the postulated complex $[Cu(AN\text{-}Ph)(DMSO)]^{+}$

Table S18. Selected bond lengths and angles for the optimised structure of the postulated complex $[Cu(AN\text{-}Ph)(DMSO)]^{+}$

Bond	Bond length (Å)	Angle	Bond angles (°)
Cu1-N6	1.951	N6-Cu1-S2	85.09
Cu1–S2	2.264	N6-Cu1-O7	84.58
Cu1–O7	2.065	N6-Cu1-O30	177.44
Cu1-O30	1.938	O7-Cu1-O30	94.40
N5-N6	1.317	S2-Cu1-O30	95.93
N5-C8	1.370	O7-Cu1-S2	169.67
C8-N3	1.355	C8-S2-Cu1	94.07
C8-S2	1.743	Cu1-O30-S27	119.99
N6-C9	1.329	N6-N5-C8	112.18
C9-C10	1.472	C8-N3-C37	134.09
S27-O30	1.586	C10-O7-Cu1	106.44
S27-C28	1.814		
S27-C29	1.815		

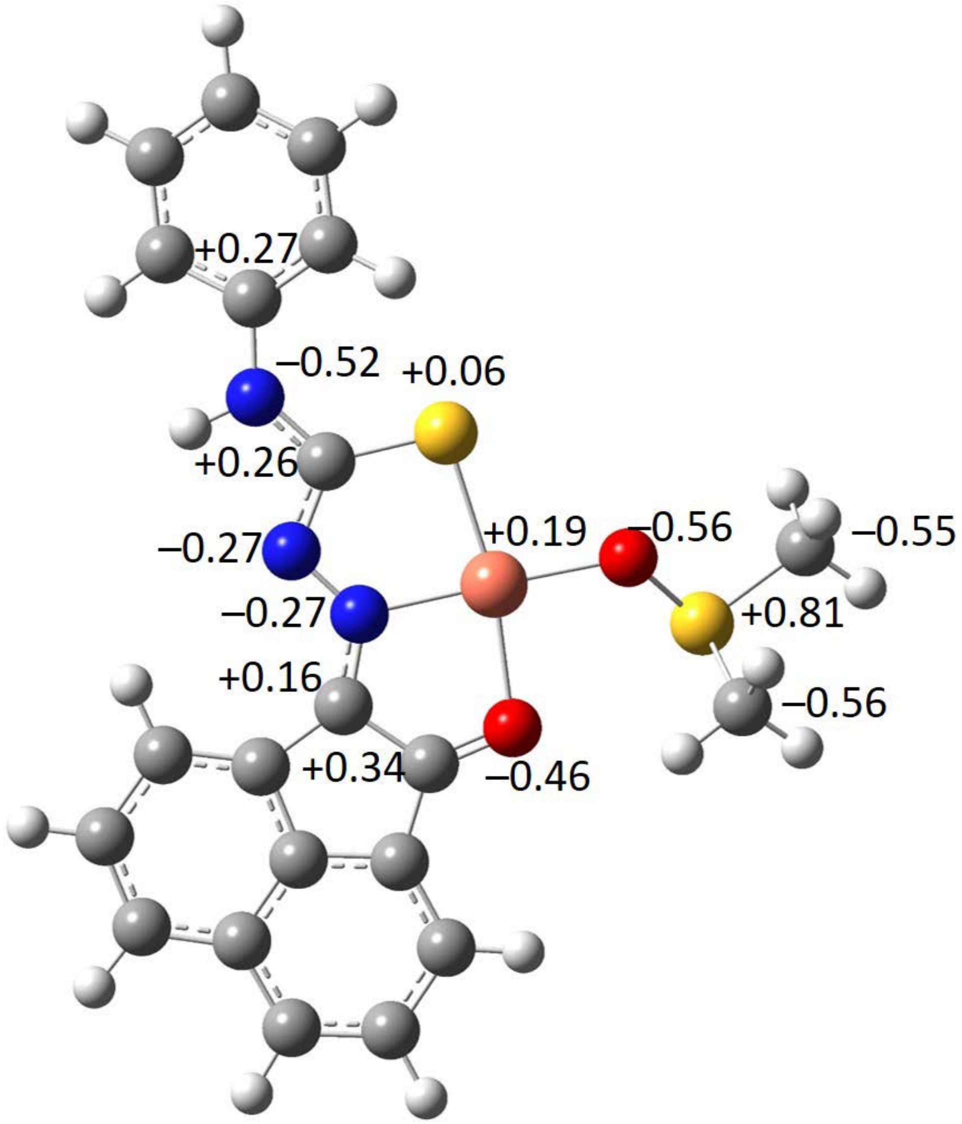

Figure S101 Mulliken charges for the optimised structure of the postulated complex [Cu(AN-Ph)(DMSO)]$^+$

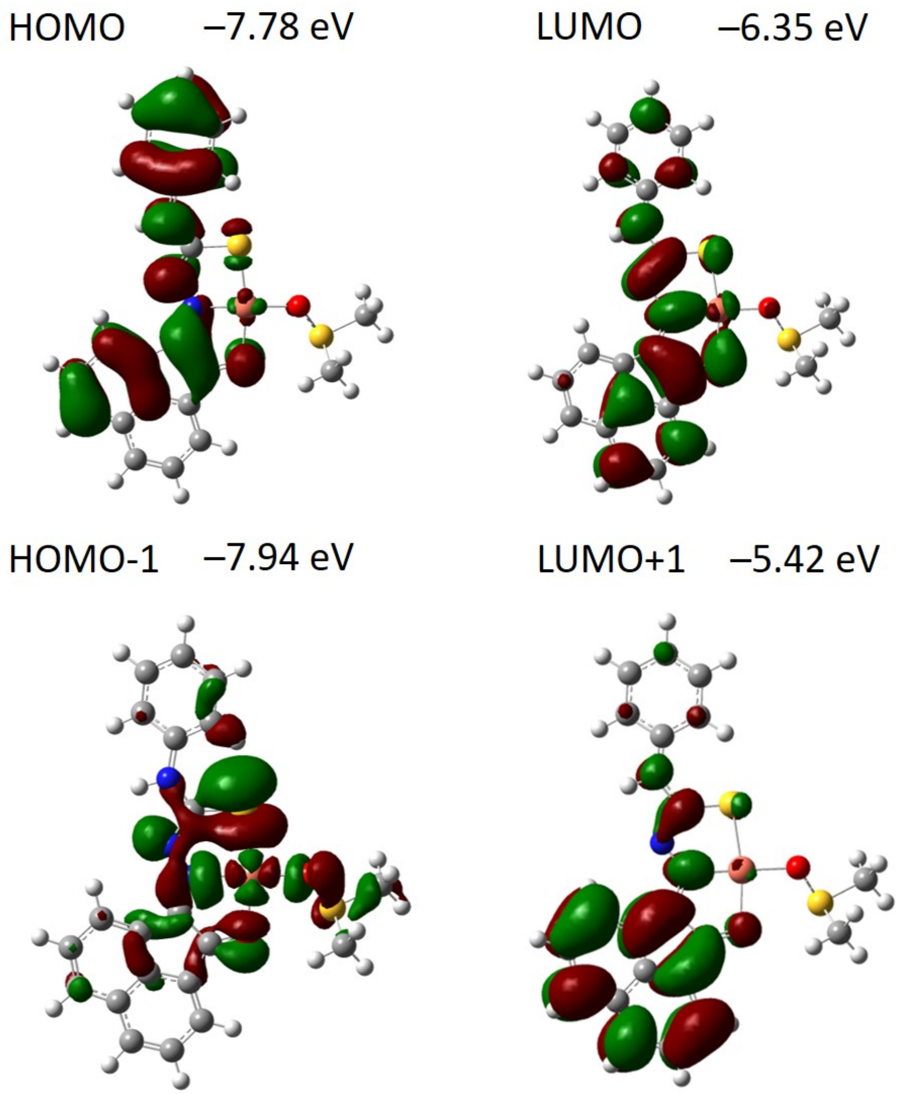

Figure S102 Shapes and energies of frontier orbitals (HOMO and LUMO) for the optimized structure of the postulated complex $[Cu(AN\text{-}Ph)(DMSO)]^+$

Table S19. Total energies calculated for all ligands and metal complexes considered in this study. HL: AN-H free base ligand; HL(NHPh) is the AN-Ph free base ligand (PBEPBE exchange correlation functional and aug-cc-PVTZ-pp/631G** basis set).

Ligand or complex	Energy		
	Hartree	eV	kJmol^{-1}
HL (AN-H)	–1137.44	–30949.74	–2986195.18
HL (AN-Ph)	–1368.20	–37228.72	–3592024.50
AcO^{-}	–228.24	–6210.41	–599213.32
AcOH	–228.83	–6226.46	–600761.91
DMSO	–552.82	–15042.23	–1451354.19
Zn $(AN\text{-}H)_2$	–2500.88	–68048.94	–6565722.9
Zn $(AN\text{-}Ph)_2$	–2962.39	–80606.63	–7777355.48
$[Zn(AN\text{-}H)(DMSO)]^{+}$	–1916.66	–52152.32	–5031932.63
$[Zn(AN\text{-}Ph)(DMSO)]^{+}$	–2147.42	–58431.29	–5637760.98
[Zn(AN-H)(OAc)]	–1592.28	–43325.94	–4180316.64
[Zn(AN-Ph)(OAc)]	–1823.03	–49604.65	–4786119.91
$Cu(AN\text{-}H)_2$	–2471.09	–67238.36	–6487513.84
$Cu(AN\text{-}Ph)_2$	–2932.60	–79796.05	–7699146.42
$[Cu(AN\text{-}H)(DMSO)]^{+}$	–1886.89	–51342.27	–4953774.71
$[Cu(AN\text{-}Ph)(DMSO)]^{+}$	–2117.65	–57621.26	–5559604.99
[Cu(AN-H)(OAc)]	–1562.49	–42515.35	–4102106.62
[Cu(AN-Ph)OAc)]	–1793.25	–48794.33	–4707935.93

Table S20. Reaction energies calculated for proposed reaction schemes involved at the interconversion of the Cu(II) species for the AN-H ligand (deprotonated L- or free base ligand HL). The reaction energies provided in the parentheses are those calculated for the AN-Ph (deprotonated L- or free base ligand HL)

Reaction	Reaction energy (kJmol^{-1})	
	M = Zn	M=Cu
$M(OAc)_2 + 2\ HL \rightarrow ML_2 + 2\ AcOH$	25.09 (56.93)	51.14 (79.12)
$M(OAc)_2 + HL \rightarrow ML(OAc) + AcOH$	1.93 (26.05)	28.95 (27.02)
$2\ ML(OAc) \rightarrow ML_2 + M(OAc)_2$	27.02 (0.96)	–0.96 (25.09)
$ML(OAc) + HL \rightarrow ML_2 + AcOH$	28.95 (27.02)	27.98 (52.01)
$ML(OAc) + DMSO \rightarrow [ML(DMSO)]^{+} + OAc^{-}$	554.79 (499.79)	472.78 (471.81)
$[ML(DMSO)]^{+} + HL \rightarrow ML_2 + DMSO + H^{+}$	1050.72 (1066.16)	1092.21 (1119.23)

11 X-Ray Crystallography: CCDC Numbers

The cif files for the X-ray structures of Compounds listed below are available free of charge from CCDC (https://www.ccdc.cam.ac.uk/), with the CCDC Deposition Numbers given below and correspnding cifs are also uploaded as separate Supplementary Information files.

1. CCDC 2218631 (s15sip1) C24H30N4O3S1 **AN-12**
2. CCDC 2218612 (Zn-mono_H)2(C26H16N6O2S2Zn1), 2(C26H16N6O2S2Zn1) - **Zn(AN-H)$_2$** (co-crystalised with a large number of disordered DMSO molecules)
3. CCDC 2218629 (FCT090) 2 (C20H22N4O4) H2O – **urea derivative byproduct** *(H2O adduct, traces from synthesis of AN-11 only)*
4. CCDC 2218628 (anthra) C18H13N3O1S1 – **AA-Me**
5. CCDC 2218626 (k08sip3) C20H15N3O1S1 – **AA-Allyl**
6. CCDC 2218624 (s18sip4) C23H15N3O1S1,C2H3N1 **AA-Ph** (CH_3CN adduct)
7. CCDC 2218623 (k07sip1) C18H15N3O1S1 -**PH-Allyl**
8. CCDC 2218622 (ox5458) C17N15N3O1S1 - **PH-Ethyl**
9. CCDC 2218769 (s18sip3) C21H15N3O1S1 -**PH-Ph**
10. CCDC 2218621 (s15sip8) C23H15N3O1S1 -**PY-Ph**
11. CCDC 2218620 (e15sip5) C23H15N3O1S1, C2H6O1S1 **PY-Ph (**DMSO adduct)
12. CCDC 2218619 (e15sip2) C20H15N3O1S1 - **PY-Allyl**
13. CCDC 2218617 (e15sip3) C19H15N3O1S1 – **PY-Et**
14. CCDC 2218616 (e15sip4) C18H13N3O1S1 -**PY-Me**
15. CCDC 2218615 (e18sip1) C38H28N6O2S2Zn1 2(C2H6O1S1) **Zn(PY-Ethyl)$_2$** (2 DMSO adduct)
16. CCDC 2218614 (ox5472) C34H28N6O2S2Zn1 2(C4H8O1) **Zn(PH-Ethyl)$_2$** (2THF adduct)
17. CCDC 2218613 (h07sip1) C36H28N6O2S2Zn1, 2(C4H8O1) **Zn(PH-Allyl)$_2$** (2THF adduct)

The CCDC 2218630 deposition corresponds to the structure of **AN-Ph (DMSO adduct)**, C19H13N3O1S1.C2H6O1S1. This single crystal was isolated from the sample resulting from the microwave irradiation reaction as reported hereby. Data was measured and structure refined to show that this compound has the same molecular structure as the previously reported polymorphs (deposition numbers CCDC 2130508 and 2130508) which were previously isolated from reactions carried under conventional heating methods. [2]

12 References

1. Alam, I. S.; Arrowsmith, R. L.; Cortezon-Tamarit F.; Twyman, F.; Kociok-Köhn G.; Botchway, S. W.; Dilworth, J. R.; Carroll, L.; Aboagye, E. O. and Pascu, S. I. Microwave Gallium-68 radiochemistry for kinetically stable bis(thiosemicarbazone) complexes: Structural investigations and cellular uptake under hypoxia, *Dalton Trans.***,** **2016**, *45*, 144.

2. Sarpaki, S.; Cortezon-Tamarit, F.; Exner, R. M.; Song, K.; de Aguiar, S. R. M. M.; Ge, H.; Pourzand, C.; Paisey, S. J.; Kociok-Köhn, G.; Dilworth, J. R.; Carroll, L. and Pascu, S. I. Functional, Aromatic, and Fluorinated Monothiosemicarbazones: Investigations into Their Structures and Activity toward the Gallium-68 Incorporation by Microwave Irradiation *ACS Omega* **2022**, *7(16)*, 13750.

3. Sarpaki, S.; Cortezon-Tamarit, F.; de Aguiar, S. R. M. M.; Exner, R. M; Divall, D.; Arrowsmith, R.L.; Ge, H.; Palomares, F.J.; Carroll, L.; Calatayud, D. G.; Paisey, S. J.; Aboagye, E. O. and Pascu, S. I. Radio- and nano-chemistry of aqueous Ga(III) ions anchored onto graphene oxide-modified complexes, *Nanoscale*, **2020**, *12*, 6603.

4. Lledos, M.; Mirabello, V.; Sarpaki, S.; Ge, H.; Smugowski, H. J.; Carroll, L.; Aboagye, E. O.; Aigbirhio, F. I.; Botchway, S.W.; Dilworth, J. R.; Calatayud, D. G.; Plucinski, P. K.; Price, G. J.; Pascu, S.I. Synthesis, Radiolabelling and In Vitro Imaging of Multifunctional Nanoceramics *ChemNanoMat* **2018**, *4*, 361.

5. Pascu, S. I.; Waghorn, P. A.; Conry, T. D.; Lin, B.; Betts, H. M.; Dilworth, J. R.; Sim, R. B.; Churchill, G. C.; Aigbirhio, F. I.; Warren, J. E. Cellular confocal fluorescence studies and cytotoxic activity of new Zn(ii) bis(thiosemicarbazonato) complexes, *Dalton Trans*, **2008**, *(16)*, 2107.

6. Pascu, S. I.; Waghorn, P. A.; Kennedy, B. W. C.; Arrowsmith, R. L.; Bayly, S. R.; Dilworth, J. R.; Christlieb, M.; Tyrrell, R.M.; Zhong, J.; Kowalczyk, R. M.; Collison, D.; Aley, P. K.; Churchill, G. C.; Aigbirhio, F. I. Fluorescent Copper(II) Bis(thiosemicarbazonates): Synthesis, Structures, Electron Paramagnetic Resonance, Radiolabeling, In Vitro Cytotoxicity and Confocal Fluorescence Microscopy Studies, *Chem Asian J.* **2010**, *5(3)*, 506.

7. Arrowsmith, R.L.; Waghorn, P.A.; Jones, M.W.; Bauman, A., Brayshaw, S.K.; Hu, Z.; Kociok-Köhn, G.; Mindt, T.L.; Tyrrell, R.M.; Botchway S.W.; Dilworth, J.R.; Pascu, S.I. Fluorescent gallium and indium bis(thiosemicarbazonates) and their radiolabelled analogues: synthesis, structures and cellular confocal fluorescence imaging investigations, *Dalton Trans.* **2011**, *40(23)*, 6238.

8. Rory Arrowsmith, PhD Thesis, University of Bath, 2013, https://purehost.bath.ac.uk/ws/portalfiles/portal/187954555/Arrowsmith_RL_Thesis_2013.pdf
